# Identification by Synthesis: Imidacins, Urocanate-Derived Alkaloids from the Myxobacterium *Stigmatella aurantiaca*

**DOI:** 10.1021/acs.orglett.4c02036

**Published:** 2024-07-22

**Authors:** Michael Kostka, Daniel Krug, Jennifer Herrmann, Jeroen S. Dickschat, Julia Meyer, Rolf Müller, Stefan Schulz

**Affiliations:** † Institute of Organic Chemistry, Technische Universität Braunschweig, Hagenring 30, 38106 Braunschweig, Germany; ‡ Helmholtz Institute for Pharmaceutical Research Saarland (HIPS), Department of Microbial Natural Products, Helmholtz Centre for Infection Research (HZI) and Department of Pharmaceutical Biotechnology, Universität des Saarlandes, Campus E8.1, 66123 Saarbrücken, Germany; § German Centre for Infection Research (DZIF), Partner Site Hannover−Braunschweig, 38124 Braunschweig, Germany; ∥ Kekulé Institute of Organic Chemistry and Biochemistry, University of Bonn, Gerhard-Domagk-Straße 1, 53121 Bonn, Germany

## Abstract

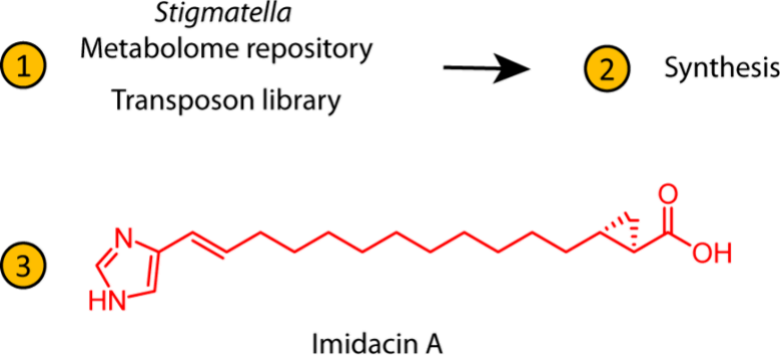

Innovative discovery approaches such as genome-mining and metabolomics-inspired methods have reshaped the natural product research field, complementing traditional bioactivity-based screens and allowing hitherto unseen compounds to be uncovered from previously investigated producers. In line with these trends, we report here imidacins, a novel class of secondary metabolites specific to the myxobacterial genus *Stigmatella*. A combination of secondary metabolome analysis, genome-mining techniques, spectroscopic analysis, and finally total synthesis was used to allow structure elucidation. Imidacins are urocanate-derived aliphatic acids with an adjacent cyclopropane moiety, structural features unprecedented in natural products to date.

In the search for new natural compounds from microorganisms, we have seen tremendous progress in bioinformatic tools to predict biosynthetic pathways from microbial genome sequences.^1^ Workflows that couple mass spectrometric comparative profiling with gene inactivation have been implemented to allow gene-to-compound correlation to arrive at new structures.^2^ A complementary approach uses bioactivity assays, structure elucidation, and “retro-biosynthetic” models to correlate with genome-encoded pathways. The combined application of this powerful analytical toolset for exploring microbial secondary metabolomes can facilitate the discovery of novel compounds even from previously scrutinized producers.^3^ Employing a metabolome-mining approach with a taxonomically diverse collection of potential producers, a high likelihood of chemical novelty for uncovered compounds is accompanied by clade-specific occurrence patterns observed for many known natural products.^4^ However, low production titers for newly annotated compounds often hinder unambiguous identification, as the detection sensitivity of MS platforms used for discovery is disparately above the level NMR offers for full structural characterization. Thus, structure confirmation by synthesis can effectively aid in the identification process of novel natural products.

We describe here the identification and synthesis of a novel class of secondary metabolites, the imidacins, which could be isolated only in minute quantities from the myxobacterial strain *S. aurantiaca* Sga15.^5^ Myxobacteria are a well-established source of natural products exhibiting diverse structures and biological activities.^6^ The genus *Stigmatella* contains genes encoding the pathways for at least ten structurally diverse compounds.^7−9^ Nevertheless, the genomes of *S. aurantiaca* DW4/3–1 and Sga15 indicated a significant additional capacity for the production of yet uncharacterized secondary metabolites, as judged by the numbers of predicted biosynthetic gene clusters.^10^ The imidacins presented here belong to this reservoir of “hidden” compounds and were ultimately revealed by a combined metabolome- and genome-mining approach.

Briefly, the procedure leading to the discovery of imidacins (Figure S1) comprised the analysis of high-resolution HPLC/MS data covering 12 known myxobacterial genera, whereby clustering of signals as previously described yielded a shortlist of 132 features predominantly appearing within the genus *Stigmatella*.^4,5^ These filtered mass spectral features–pairs of retention time and *m*/*z* values–were subsequently connected with HPLC/MS data from a library of clones generated by random transposon mutagenesis of *S. aurantiaca* Sga15. This step served to identify mutants lacking any feature from the filtered candidate list.^5,9^ Through this combined analysis a feature with *m*/*z* 347 (**c347**, C_21_H_34_N_2_O_2_, [M + H]^+^*m*/*z* 347.2693, calc. 347.2699) was highlighted, occurring in 29 *Stigmatella* strains. At the same time, this feature was absent from one transposon-inserted clone, together with a related feature with 319 *m*/*z* (**c319**, C_19_H_30_N_2_O_2_, [M + H]^+^*m*/*z* 319.2374, calc. 319.2386). The transposon insertion site has been located inside a gene encoding typical biosynthetic domains, later revealed as an apparent operon comprising a relatively short type-I polyketide synthase (PKS) assembly line. This mapping was achieved by comparing the recovered sequence from the transposon experiment with a draft genome of the *S. aurantiaca* Sga15 wildtype strain.^5^ However, the prediction of product structures was not feasible through bioinformatic analysis based on available sequence information. Nevertheless, mass spectral data suggested that the two candidate features likely constituted novel myxobacterial natural products.

Initial attempts to isolate the candidate compounds were not met with success due to the very low yield obtained from Sga15 even after culture optimization. Therefore, a mutant strain was constructed in which the biosynthetic operon was placed under the control of a constitutive promotor, utilizing a previously established single-crossover plasmid insertion approach.^5,7^ This genetic manipulation helped to increase production approximately 5-fold, enabling the isolation of small amounts of pure compounds for which the yield was estimated in the range of only 10–20 μg/L culture.^5^ The isolated materials were used for direct-infusion ESI-MS^n^ analysis, GC/MS analysis following derivatization with *N*-methyl-*N*-(trimethylsilyl)-trifluoracetamide, and ^1^H NMR spectroscopy (^5^ and Figure S2). The MS^n^ spectra revealed the loss of a terminal carboxylic acid group and several neutral fragments along an alkyl chain to leave a charged end group containing two nitrogens.^11^ EI mass spectra also indicated the presence of alkyl chains in both derivatives differing in length by two methylene units (Figure S3). The 40 Da (C_3_H_4_) distance between distinct fragments (*m*/*z* 291/331 and 319/359) hinted at the presence of a cyclopropane group. This structural detail was further substantiated by ^1^H NMR data (Figure S2), showing the expected signals for protons located at a disubstituted cyclopropane ring below 1 ppm. Nevertheless, due to signal overlap with impurities, no clear assignment of the configuration or substitution of the cyclopropane unit was possible. NMR analysis also disclosed the nitrogen-containing substructure as an (*E*)-vinylimidazole moiety (signals at 7.7, 6.9. 6.7, and 6.3 ppm and *J* = 16.0 Hz) connected to an alkyl chain, whereas the exact structure of this chain could not be inferred due to signal overlaps. These data indicate the compounds–named imidacins–exhibit novel structures as shown in Figure 1, although the available analytical data initially left the position of the cyclopropane ring unclear, as well as its *cis-* or *trans-*configuration. This structural issue was resolved by total synthesis.

**Figure 1:**
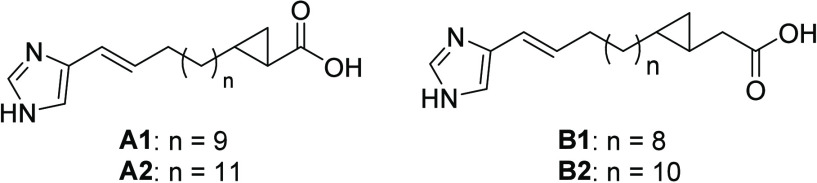
Structural proposals for imidacins **c319** (A1/B1) and **c347** (A2/B2).

Proposal **B1** was synthesized using two consecutive Wittig-Schlosser reactions^12^ and a final Simmons-Smith cyclopropanation as key steps (Schemes S1 and S2). Trityl-protected imidazol-2-carbaldehyde^13^ (**8**) reacted smoothly with the Wittig reagent obtained from 11-bromoundecan-1-ol. After Swern oxidation, the resulting aldehyde was subjected to a second Wittig-Schlosser reaction using 3-bromopropan-1-ol as a building block. Replacing this Wittig reaction with the respective Julia-Kocienski reaction led to an increased yield.

Final cyclopropane formation according to the Furukawa modification^14^ proved to be regio-unselective, furnishing a mixture of cyclopropanation products. Nevertheless, pure imidacin B1 could be obtained after the final oxidation of the terminal alcohol to the acid. Unfortunately, the ^1^H NMR spectra of the natural compound and imidacin B1 differed. The details of the synthesis can be found in the SI, Section 3.

Therefore, we turned our attention to proposal A1. Because of the problems encountered in the previous synthesis, the sequence of steps was changed, performing cyclopropanation earlier, after the formation of the first double bond (Scheme 1). Starting from 1,12-dodecanediol (**1**), 12-bromododecanal (**2**) was prepared by standard methods. A Horner-Wadsworth–Emmons reaction delivered alcohol **3** that was converted into *trans*-cyclopropane alcohol **4** in excellent yield. The formation of the Wittig salt was straightforward if NaI was used as an additive. The following Wittig-Schlosser reaction exclusively produced the *E-*configured alcohol **6**, indicated by ^3^*J* = 15.8 Hz found for the vinylic protons. The best procedure to convert this alcohol to acid **7** turned out to be a two-step sequence with initial MnO_2_ oxidation to the aldehyde, followed by Pinnick oxidation.^15^ Finally, the trityl protecting group was cleaved with acetic acid to furnish pure **9**. This material proved to be identical to the natural material **c319**, presenting an *E*-configured double bond and a *trans*-configured cyclopropane ring. Similarly, the bis-homologue of **9**, imidacin A2, was synthesized and proved to be identical to **c347**. For comparison, we also synthesized *cis*-imidacin A1, exhibiting a *cis-*configured cyclopropane unit. While the overall sequence was similar to that shown in Scheme 1, instead of **3** its *Z*-isomer (*Z*)-14-bromotetradec-2-en-1-ol was needed. This alcohol was synthesized from **2** by the Still-Gennari variation of the Horner-Wadsworth-Emmons reaction,^16^ followed by DIBAL reduction (Scheme S3).

**Scheme 1:**
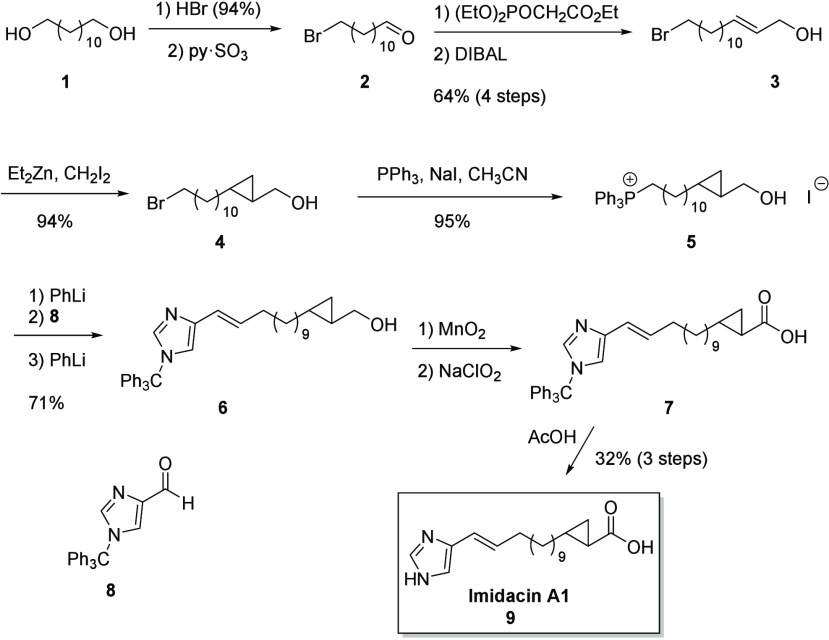
Synthesis of Racemic Imidacin A1 (**9**)

While the relative configuration was successfully elucidated, we turned our attention to the absolute configuration of the imidacins. The natural material of imidacin A1 showed an [α]_D_^22^ of −18 (*c* 0.05, MeOH). We therefore modified the synthetic route to synthesize both enantiomers of **9** enantioselectively by replacing the Simmons-Smith cyclopropanation with the Charette methodology^17^ (Scheme 2).

**Scheme 2:**
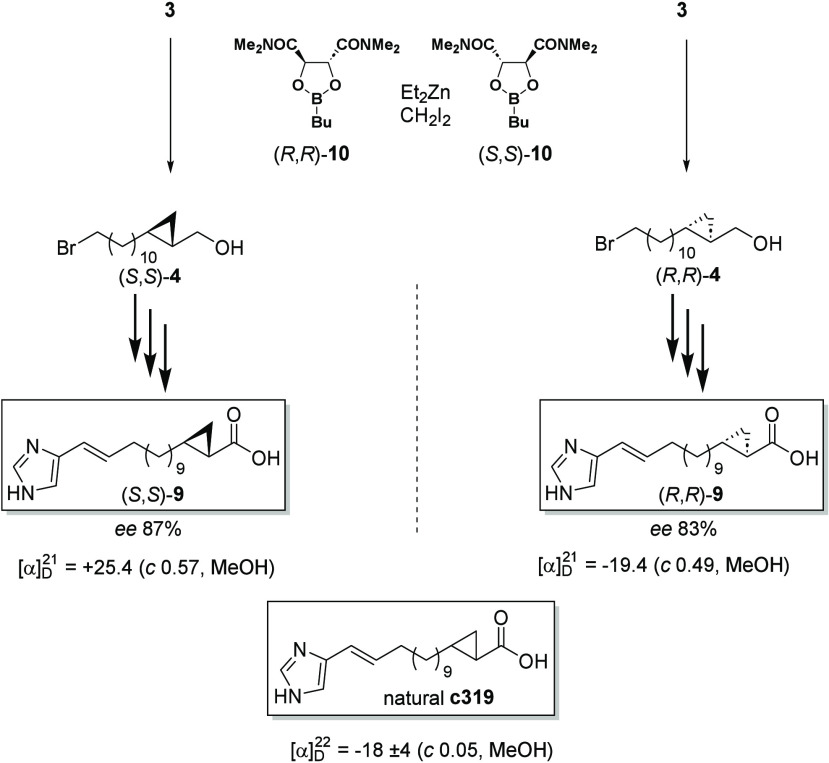
Enantioselective Synthesis of the Two Enantiomers of Imidacin A1 and Their Optical Rotation Values Compared to That of the Natural Compound The (*R*,*R*)-configuration of the latter is shown.

Starting from alcohol **3**, the two enantiomers of **4** were obtained in excellent yield with moderate *ee* > 80% (Scheme 1). It is well documented that ligand (*S*,*S*)-**10** leads to cyclopropylcarbinol (*R*,*R*)- **4**, readily converted into (*R*,*R*)-**9**. The specific optical rotation of this compound had the same sense and a similar value as the natural sample (Scheme 2). Therefore, the natural imidacins preferentially occur with (*R*,*R*)-configuration.

The establishment of the imidacin structures allowed additional investigations of their biosynthesis by *S. aurantiaca* Sga15. The terminal imidazole ring is a striking feature not previously reported from any PKS-derived metabolites, although related bulbimidazoles have been reported, derived from fatty acid biosynthesis.^18^ In the latter case, the imidazole moiety is likely introduced late in biosynthesis because a saturated alkyl chain and a typical bacterial methyl-branched fatty acids signature (*n*, *iso*, and *anteiso*) is present (see Scheme S5).^18^ The imidacins contain no alkyl end and the location of the imidazole ring opposite to the acid indicated an imidazole-containing biosynthetic starter unit.

Feeding (^15^N_3_)histidine to Sga15 cultures has indicated that the imidazole moiety stems from histidine with a loss of one nitrogen as revealed by HPLC/MS analysis.^5^ Furthermore, an enzyme capable of carrying out deamination of l-His, histidine ammonium-lyase (HAL), is known from many bacterial species and has also been identified from *S. aurantiaca* Sga15.^5^ HAL_Sa_ has been shown to catalyze the elimination of ammonia leading to the formation of urocanic acid, as it is also known as the first step of a general bacterial degradation pathway from l-His to l-Glu. Using recombinant HAL_Sa,_ commercially available (^13^C_6_)His has been converted *in vitro* into (^13^C_6_)urocanic acid, which was subsequently used for a feeding study.^5^ The observed mass shift of +6 Da in that experiment indicated that all carbon atoms from urocanic acid are incorporated into imidacins. Consequently, these data indicate a biosynthesis route in which a urocanate starter unit (**12**) is elongated by multiple malonate units to arrive at unsaturated acid **13** or its bishomolog, the likely precursors of the final cyclopropanation (Scheme 3). Furthermore, it has been reasoned that the cyclopropane moiety should result from the methylenation by a SAM-dependent cyclopropane synthase, as concluded from the clear incorporation of two deuterium labels after feeding of (*methyl*-^2^H_3_)-l-methionine.^5^

**Scheme 3:**
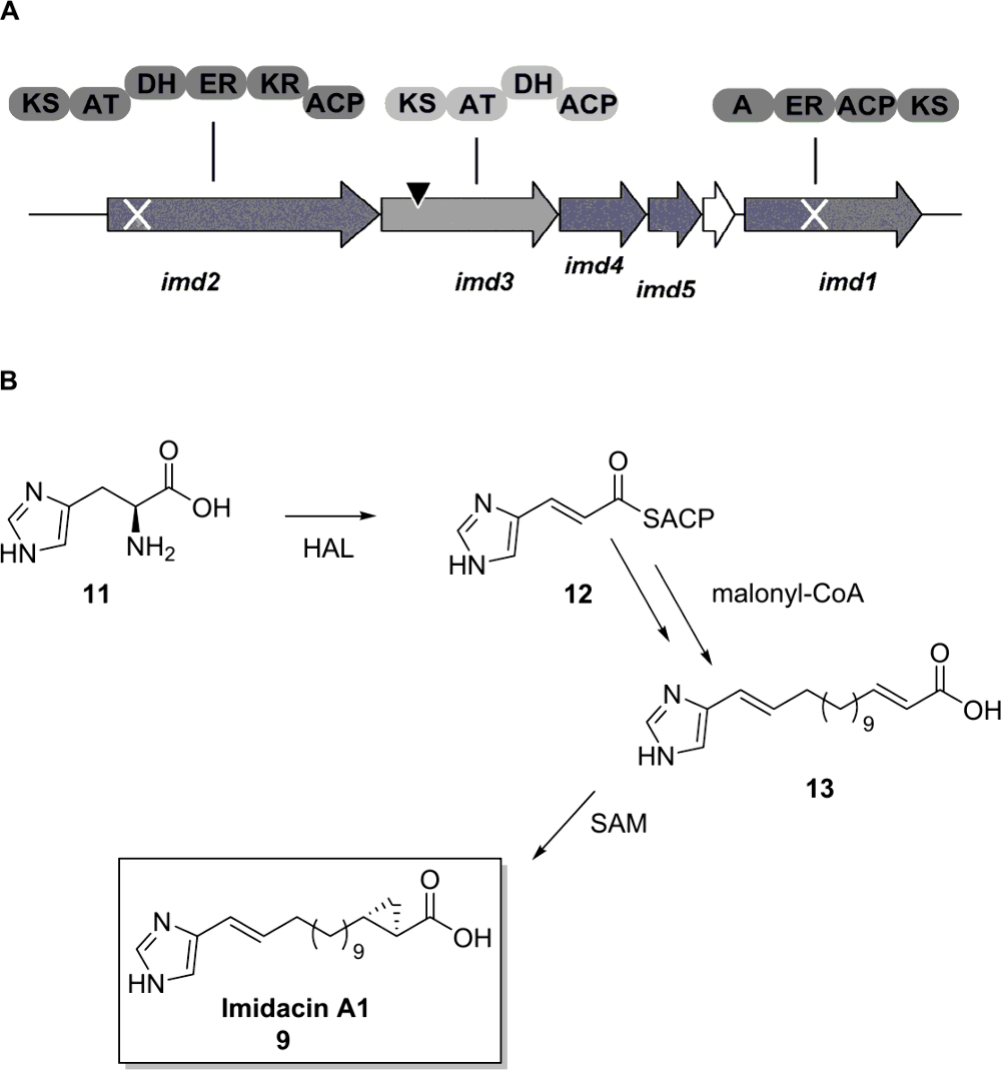
Overview of the *imd* Biosynthetic Gene Cluster in *S. aurantiaca* Sg a15 The proposed biosynthesis recruits urocanate supplied by histidine ammonia lyase (HAL) encoded distantly in the genome. It then proceeds with chain extension according to PKS-type biosynthetic logic, encoded by *imd1*, *imd2* and *imd3*, whereas the reductive loop represented by *imd2* is likely used iteratively. Domains: A, adenylation domain; KS, ketosynthase; AT, acyltransferase; DH, dehydratase; ER, enoylreductase; KR, ketoreductase; ACP, acyl carrier protein. White cross, targeted gene inactivation experiment; black triangle, transposon insertion site. Adapted from ref (5).

However, an antimicrobial bioassay containing various bacteria (*rac*)-imidacins A1 showed no activity. The pure enantiomers of imidacin A1 were slightly (*S*,*S*) to moderately (*R*,*R*) active against two TolC-deficient *E. coli* strains, presumably because of their higher concentration. The unnatural *cis*-imidacin A1 was moderately active against the two TolC-deficient *E. coli* strains and slightly active against *Staphylococcus aureus*, *Mucor hiemalis*, and *Bacillus subtilis* (SI). No activity was observed against *Pseudomonas*, in contrast to the initial results obtained with the enriched material obtained from culture extracts. Therefore, the initially observed activity might originate from other components of the extract fractions, probably present in even lower concentrations than the imidacins.

In conclusion, a comprehensive discovery approach integrating genomic and mass spectral data and total synthesis allowed the identification of unprecedented urocanoic acid-derived natural products, the imidacins A1 and A2 from the known myxobacterial secondary metabolite producer *S. aurantiaca*. Due to their low abundance, their structures and absolute configuration could only be resolved by an enantioselective total synthesis. Our data are a strong indication that total synthesis will remain of prime importance in the future for the structure determination of novel microbial natural products and material delivery for biological testing.

## Data Availability

The data underlying this study are available in the published article and its Supporting Information

## Supporting Information Available

Experimental details, materials, and methods (PDF)
^1^H and ^13^C NMR spectra for all compounds (PDF)

## References

[ref1] aZiemert N.; Alanjary M.; Weber T. The evolution of genome mining in microbes - a review. Nat. Prod. Rep. 2016, 33, 988–1005. 10.1039/C6NP00025H. 27272205

[ref2] Cortina N. S.; Krug D.; Plaza A.; Revermann O.; Müller R. Myxoprincomide: a natural product from *Myxococcus xanthus* discovered by comprehensive analysis of the secondary metabolome. Angew. Chem., Int. Ed. Engl. 2012, 51, 811–816. 10.1002/anie.201106305. 22162209

[ref3] Hoffmann T.; Krug D.; Hüttel S.; Müller R. Improving natural products identification through targeted LC-MS/MS in an untargeted secondary metabolomics workflow. Anal. Chem. 2014, 86, 10780–10788. 10.1021/ac502805w. 25280058

[ref4] Hoffmann T.; Krug D.; Bozkurt N.; Duddela S.; Jansen R.; Garcia R.; Gerth K.; Steinmetz H.; Müller R. Correlating chemical diversity with taxonomic distance for discovery of natural products in myxobacteria. Nat. Commun. 2018, 9, 803 10.1038/s41467-018-03184-1. 29476047 PMC5824889

[ref5] Krug D. Natural product biosynthesis in myxobacteria: Studies on enzymatic versatility and secondary metabolite diversity. Dissertation, Universität des Saarlandes, Saarbrücken, German, 2009. 10.22028/D291-22581.

[ref6] aWeissman K. J.; Müller R. Myxobacterial secondary metabolites: bioactivities and modes-of-action. Nat. Prod. Rep. 2010, 27, 1276–1295. 10.1039/c001260m. 20520915

[ref7] Beyer S.; Kunze B.; Silakowski B.; Müller R. Metabolic diversity in myxobacteria: identification of the myxalamid and the stigmatellin biosynthetic gene cluster of *Stigmatella aurantiaca* Sg a15 and a combined polyketide-(poly)peptide gene cluster from the epothilone producing strain *Sorangium cellulosum* So ce90. Biochim. Biophys. Acta 1999, 1445, 185–195. 10.1016/S0167-4781(99)00041-X. 10320771

[ref8] aSilakowski B.; Nordsiek G.; Kunze B.; Blöcker H.; Müller R. Novel features in a combined polyketide synthase/non-ribosomal peptide synthetase: the myxalamid biosynthetic gene cluster of the myxobacterium *Stigmatella aurantiaca* Sga15. Chem. Biol. 2001, 8, 59–69. 10.1016/S1074-5521(00)00056-9. 11182319

[ref9] Sandmann A.; Dickschat J.; Jenke-Kodama H.; Kunze B.; Dittmann E.; Müller R. A type II polyketide synthase from the gram-negative bacterium *Stigmatella aurantiaca* is involved in aurachin alkaloid biosynthesis. Angew. Chem., Int. Ed. Engl. 2007, 46, 2712–2716. 10.1002/anie.200603513. 17335090

[ref10] Ronning C. M.; Nierman W. C. The genomes of *Myxococcus xanthus* and *Stigmatella aurantiaca*. In Myxobacteria: Multicellularity and differentiation; Whitworth D., Ed.; ASM Press, 2007; pp 285–298.

[ref11] Denekamp C.; van den Heuvel H.; Voinov V. G.; Claeys M.; Seto C.; Grossert J. S.; Waddell D. S.; Curtis J. M.; Boyd R. K. Charge-remote fragmentation characteristics of functionalized alkanes in high-energy collision-induced dissociation. Rapid Commun. Mass Spectrom. 2000, 14, 1035–1043. 10.1002/1097-0231(20000630)14:12<1035::AID-RCM986>3.0.CO;2-4. 10861984

[ref12] Schlosser M.; Tuong H. B.; Schaub B. The betaine-ylid route to trans-alkenols. Tetrahedron Lett. 1985, 26, 311–314. 10.1016/S0040-4039(01)80805-4.

[ref13] aBertinaria M.; Di Stilo A.; Tosco P.; Sorba G.; Poli E.; Pozzoli C.; Coruzzi G.; Fruttero R.; Gasco A. [3-(1H-Imidazol-4-yl)propyl]guanidines containing furoxan moieties. Bioorg. Med. Chem. 2003, 11, 1197–1205. 10.1016/S0968-0896(02)00651-X. 12628647

[ref14] Furukawa J.; Kawabata N.; Nishimura J. Synthesis of cyclopropanes by the reaction of olefins with dialkylzinc and methylene iodide. Tetrahedron 1968, 24, 53–58. 10.1016/0040-4020(68)89007-6.

[ref15] Beckmann C.; Rattke J.; Sperling P.; Heinz E.; Boland W. Stereochemistry of a bifunctional dihydroceramide D4-desaturase/hydroxylase from *Candida albicans*; a key enzyme of sphingolipid metabolism. Org. Biomol. Chem. 2003, 1, 2448–2454. 10.1039/B303939K. 12956060

[ref16] Mergott D. J.; Frank S. A.; Roush W. R. Application of the Intramolecular Vinylogous Morita-Baylis-Hillman Reaction toward the Synthesis of the Spinosyn A Tricyclic Nucleus. Org. Lett. 2002, 4, 3157–3160. 10.1021/ol026540d. 12201741

[ref17] Charette A. B.; Juteau H.; Lebel H.; Molinaro C. Enantioselective Cyclopropanation of Allylic Alcohols with Dioxaborolane Ligands: Scope and Synthetic Applications. J. Am. Chem. Soc. 1998, 120, 11943–11952. 10.1021/ja982055v.

[ref18] Karim M. R. U.; Harunari E.; Oku N.; Akasaka K.; Igarashi Y. Bulbimidazoles A-C, Antimicrobial and Cytotoxic Alkanoyl Imidazoles from a Marine Gammaproteobacterium *Microbulbifer* Species. J. Nat. Prod. 2020, 83, 1295–1299. 10.1021/acs.jnatprod.0c00082. 32191468

