# Supplementary material for: Identification by Synthesis: Imidacins, Urocanate-Derived Alkaloids from the Myxobacterium Stigmatella aurantiaca
Source: Org Lett. 2024 Jul 22;26(30):6359–63. doi: 10.1021/acs.orglett.4c02036 (PMC11301661; doi:10.1021/acs.orglett.4c02036)
Supplement: Supplementary file 1 — ol4c02036_si_001.pdf [file ol4c02036_si_001.pdf]

Supporting information

Identification by Synthesis: Imidacins, Urocanate Derived Alkaloids from the Myxobacterium *Stigmatella aurantiaca*

Michael Kostka[1‡], Daniel Krug[2,3‡], Jennifer Herrmann[2], Jeroen S. Dickschat[2,4†], Julia Meyer[1], Rolf Müller[2,3], Stefan Schulz[1]*

1 Institute of Organic Chemistry, Technische Universität Braunschweig, Hagenring 30, 38106 Braunschweig, Germany.

2 Helmholtz Institute for Pharmaceutical Research Saarland (HIPS), Department of Microbial Natural Products, Helmholtz Centre for Infection Research (HZI) and Department of Pharmaceutical Biotechnology, Universität des Saarlandes, Campus E8.1, 66123 Saarbrücken, Germany.

3 German Centre for Infection Research (DZIF), Partner Site Hannover–Braunschweig, Germany.

4 Kekulé Institute of Organic Chemistry and Biochemistry, University of Bonn, Gerhard-Domagk-Straße 1, 53121 Bonn, Germany.

Content

1 Analytical workflow 2

2 Spectra of natural Imidacins 3

3 Synthesis of imidacin B1 and *cis*-imidacin A1 6

4 Proposed biosynthese of bulbimidazols 10

5 Experimental part 11

- 5.1 General methods 11
- 5.2 Bioactivity of imidacins 11
- 5.3 General procedures 13
- 5.4 Synthesis of imidacin A1 15
- 5.5 Synthesis of imidacin *cis*-A1 21
- 5.6 Enantioselective syntheses of imidacin A1 27
- 5.7 Synthesis of imidacin A2 35
- 5.8 Synthesis of imidacin B1 42
- 5.9 Methods for cultivation, manipulation and analysis of *S. aurantiaca* 52

6 References 55

1 Analytical workflow

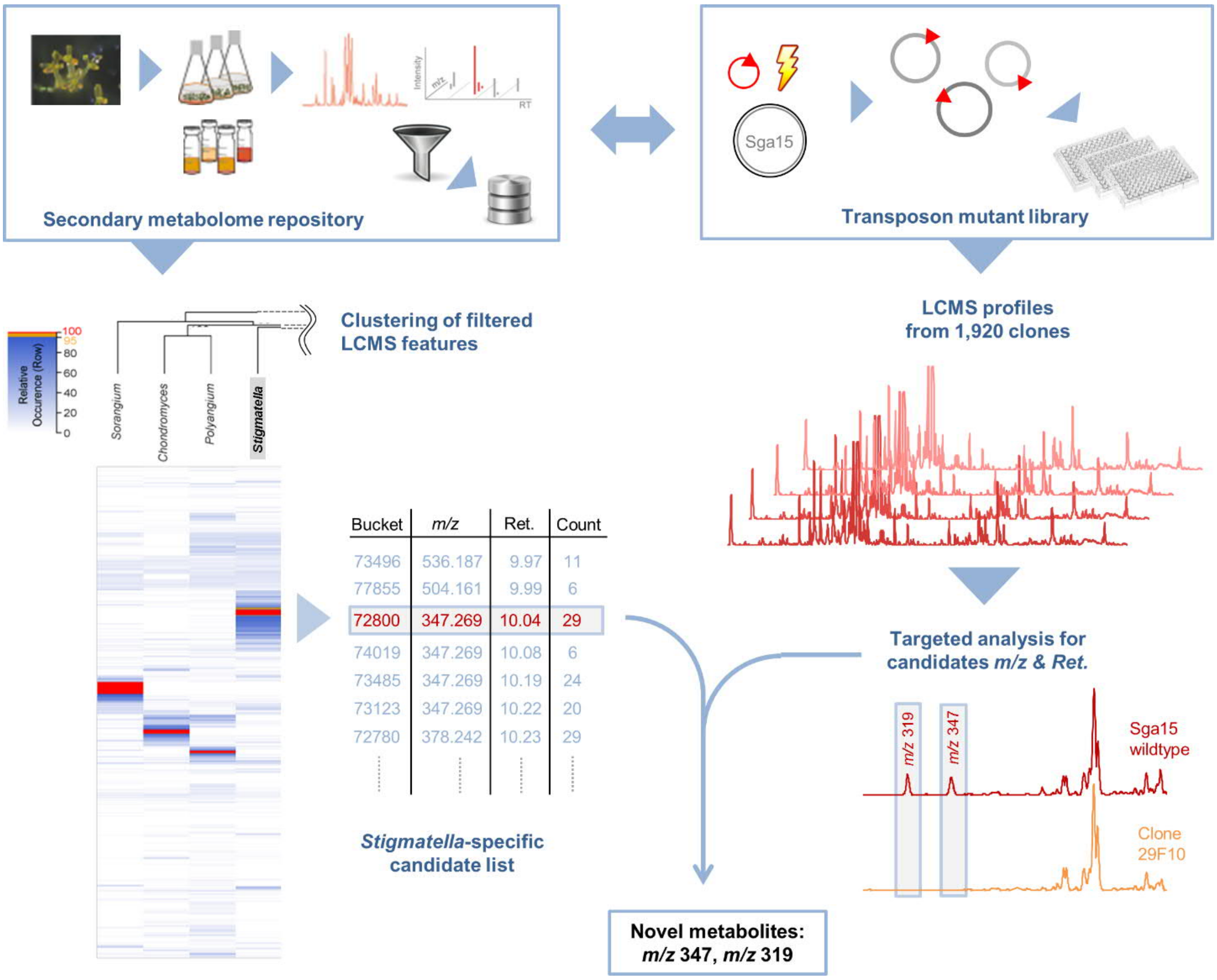

Figure S1. Schematic description of the analysis resulting in the detection of analytes **c319** and **c347** (ESI$^+$, [M+H]$^+$). A previously established secondary metabolome repository of myxobacterial strains was analyzed by LC/MS and their features were filtered for occurrence within specific taxonomic clades.[S1] The analysis of a transposon mutant library of *S. aurantiaca* showed mutants lacking the target analytes. This allowed assignment of the biosynthetic gene cluster by comparison of mutated loci to the *S. aurantiaca* genome sequence (see section 5.9).

2 Spectra of natural imidacins

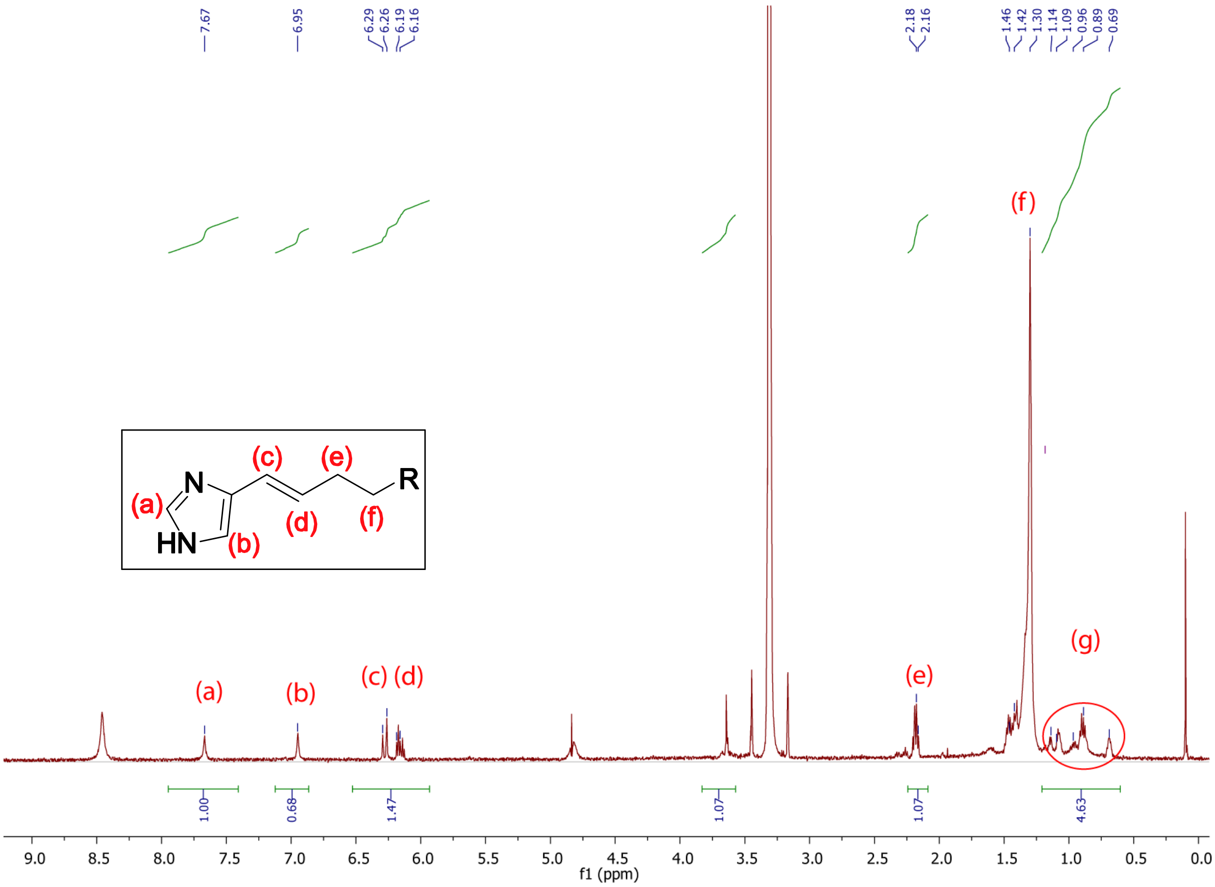

Figure S2. ^{1}H NMR (600 MHz) spectrum of isolated **c319**, imidacin A1. (g): protons of the cyclopropane ring.

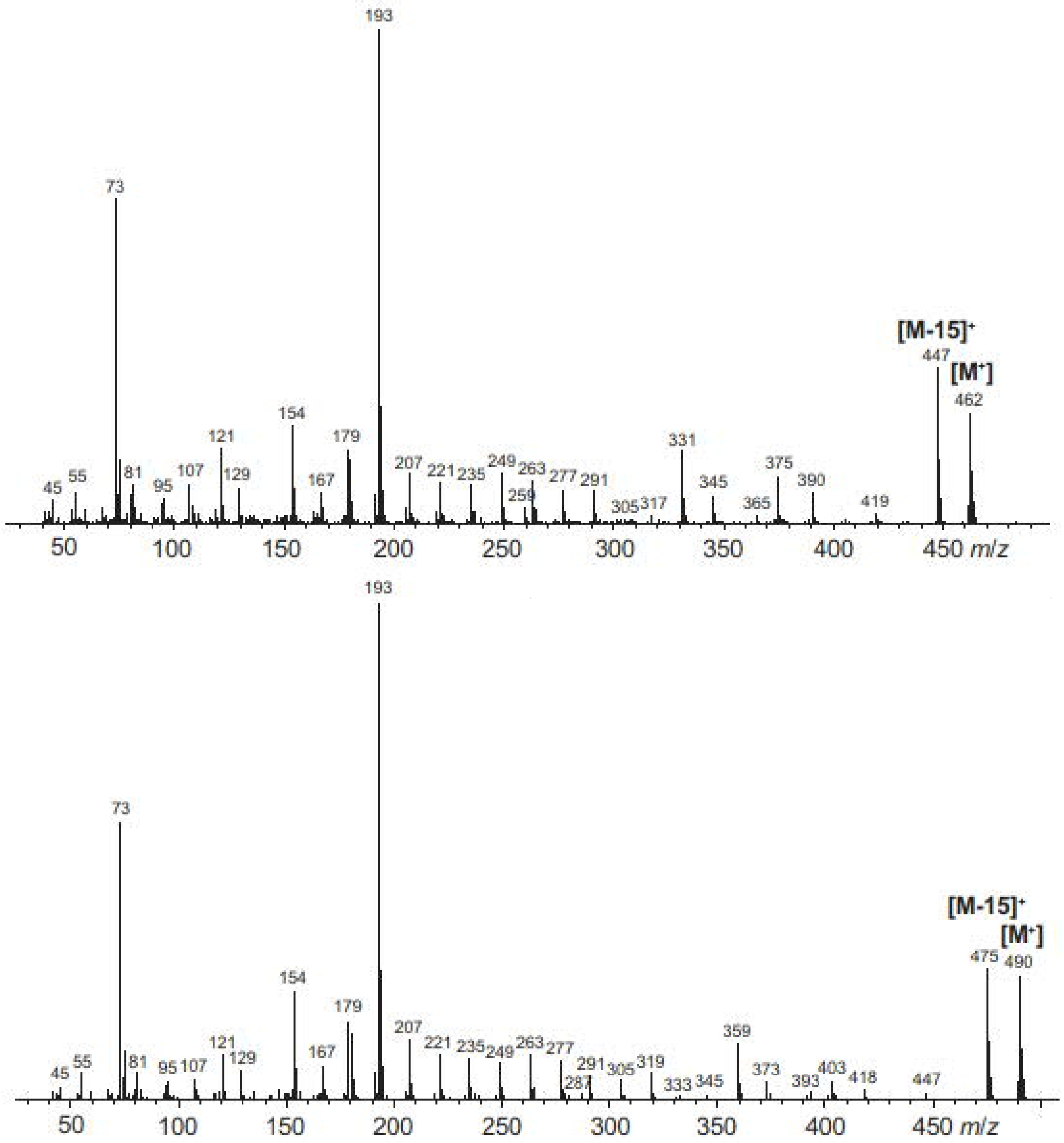

Figure S3. EI mass spectra of imidacins isolated from myxobacterial extracts (**c319**, top and **c347**, bottom) derivatized with *N*-methyl-*N*-(trimethylsilyl)-trifluoroacetamide.

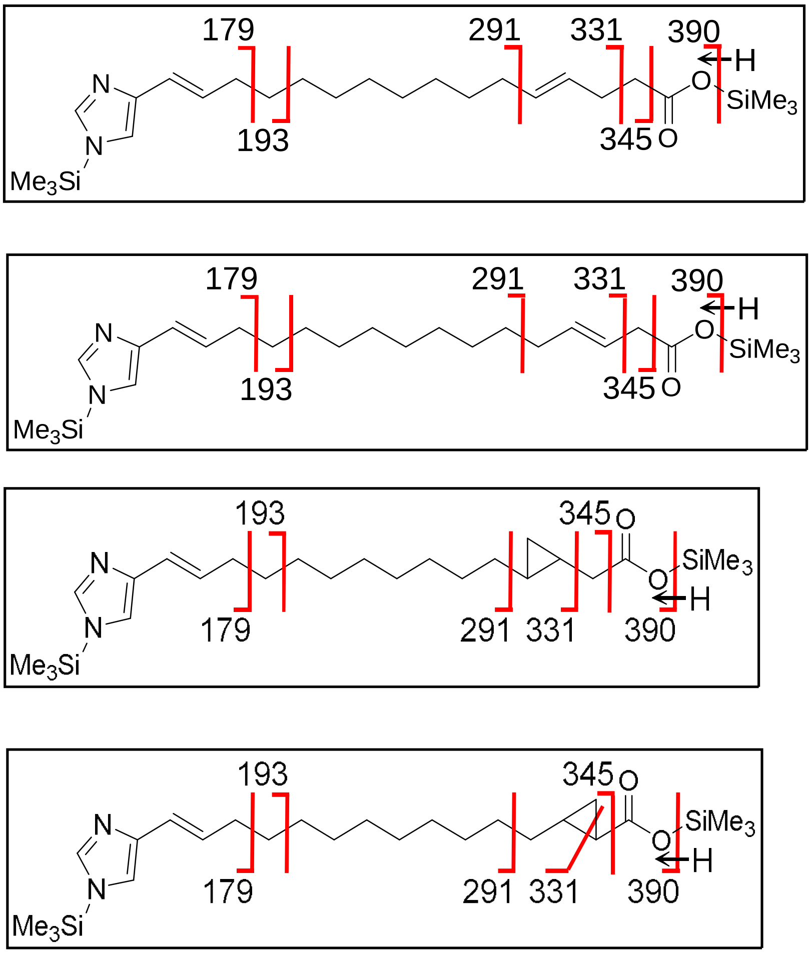

Figure S4. Candidate structures and possible mass spectrometric fragmentation of trimethylsilylated **c319**.

3 Synthesis of imidacin B1 and *cis*-Imidacin A1

The synthesis of imidacin B1 (**31**) needed a homoallyl alcohol to allow construction of its cyclopropylethanol structural fragment (Scheme S1). Therefore, bromoalcohol **14** was synthesized starting from undecane-1,11-diol and converted into the Wittig-reagent **15**. The following Wittig-Schlosser reaction with trityl-protected imidazolcarbaldehyde **8**[S2,S3] furnished alcohol **16**, which was cleanly converted into the respective aldehyde **17** via Swern oxidation. The establishment of the (*E*)-homoallyl alcohol fragment proved to be difficult. By use of again Wittig-Schlosser conditions the required alcohol **19** was obtained in low yield, together with the phenyl adduct **20**. Optimization of the reaction conditions did not furnish a higher yield of **19**. Therefore, an alternative Julia-Kocienski-Olefination using KHMDS as base to ensure (*E*)-configuration was performed, using sulfone **18**, derived form 3-bromopropan-1-ol. The required deprotection lowered the yield, but the procedure furnished better yields and a cleaner product compared to the Wittig-Schlosser approach. Final cyclopropane formation according to the Furukawa modification[S4] proved to be regio-unselective, furnishing a mixture of cyclopropanation products **21** and **22** in roughly equal amounts. Nevertheless, pure imidacin B2 (**24**) was obtained after final oxidation of the terminal alcohol into the acid **23** and deprotection, but the mass spectrum after trimethylsilylation differed from that of the natural **c319**.

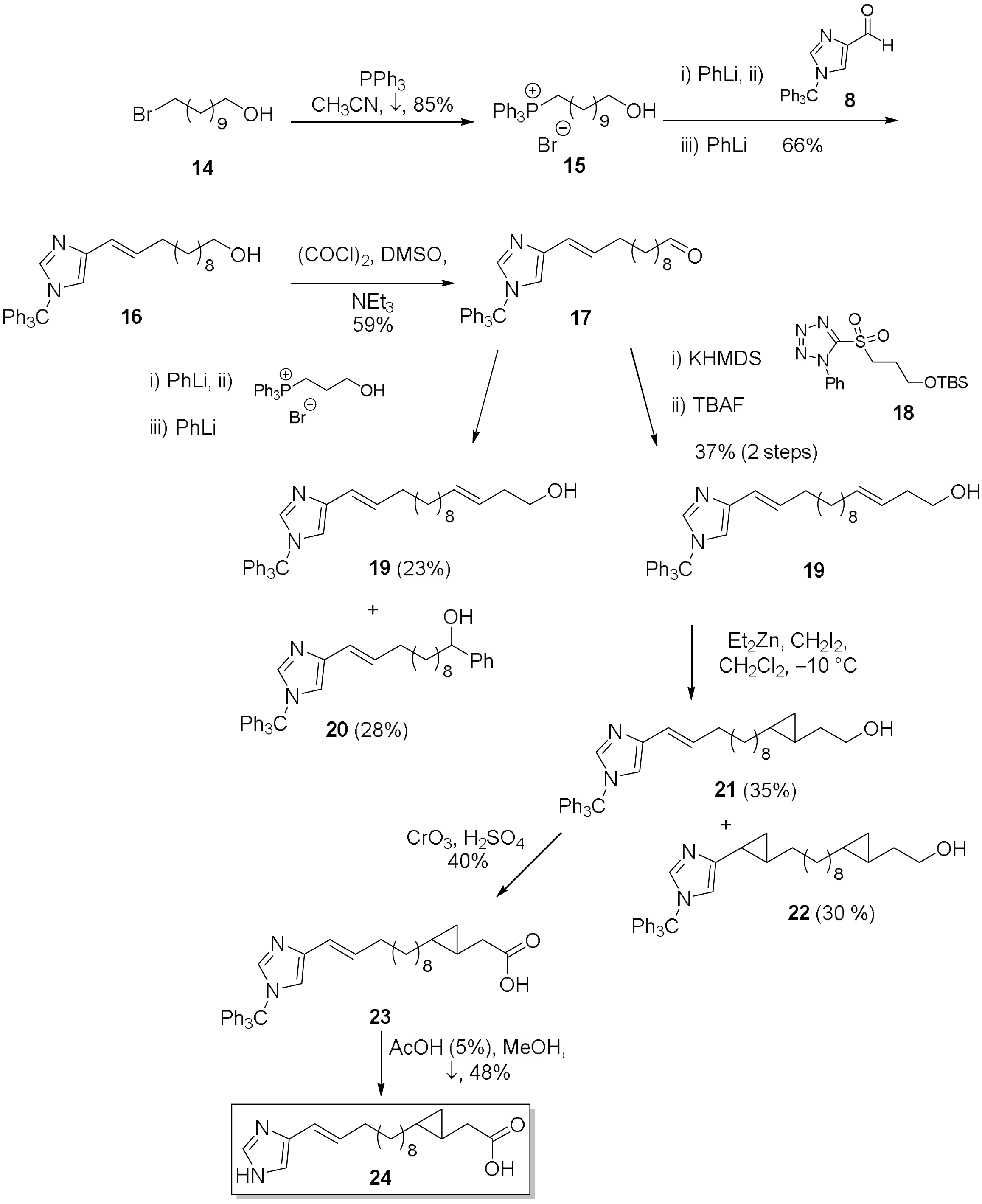

Scheme S1. First synthesis of imidacin B1 (**24**), not yet discovered as natural product.

Therefore, the synthetic strategy was revised, starting with an early introduction of the cyclopropane ring into a Wittig reagent and final construction of the urocanate moiety, as described in the main text for imidacin A1. Parikh-Doering oxidation delivered aldehyde **25** that was used in a following Julia-Kocienski coupling with **18**. The (*E*)-alcohol **26** was obtained cleanly, but in moderate yield, due to a side reaction leading to the respective fluoride **27** by nucleophilic substitution. Cyclopropanation was uneventful, as was the Wittig salt preparation. Nevertheless, NaI is needed for a high yield. Construction for the carbon framework is

concluded by a Wittig-Schlosser reaction with trityl-protected imidazolcarbaldehyde **8** leading to (*E*)-configured imidazol **21** in moderate yield. While Pinnick-oxidation only gave low yields, Jones-oxidation allowed formation of the protected acid **23**. Finally, removal of the trityl group delivered imidacin B_1 (**24**). Purification of **24** and other imidacins proved to be difficult due to losses during final column chromatography.

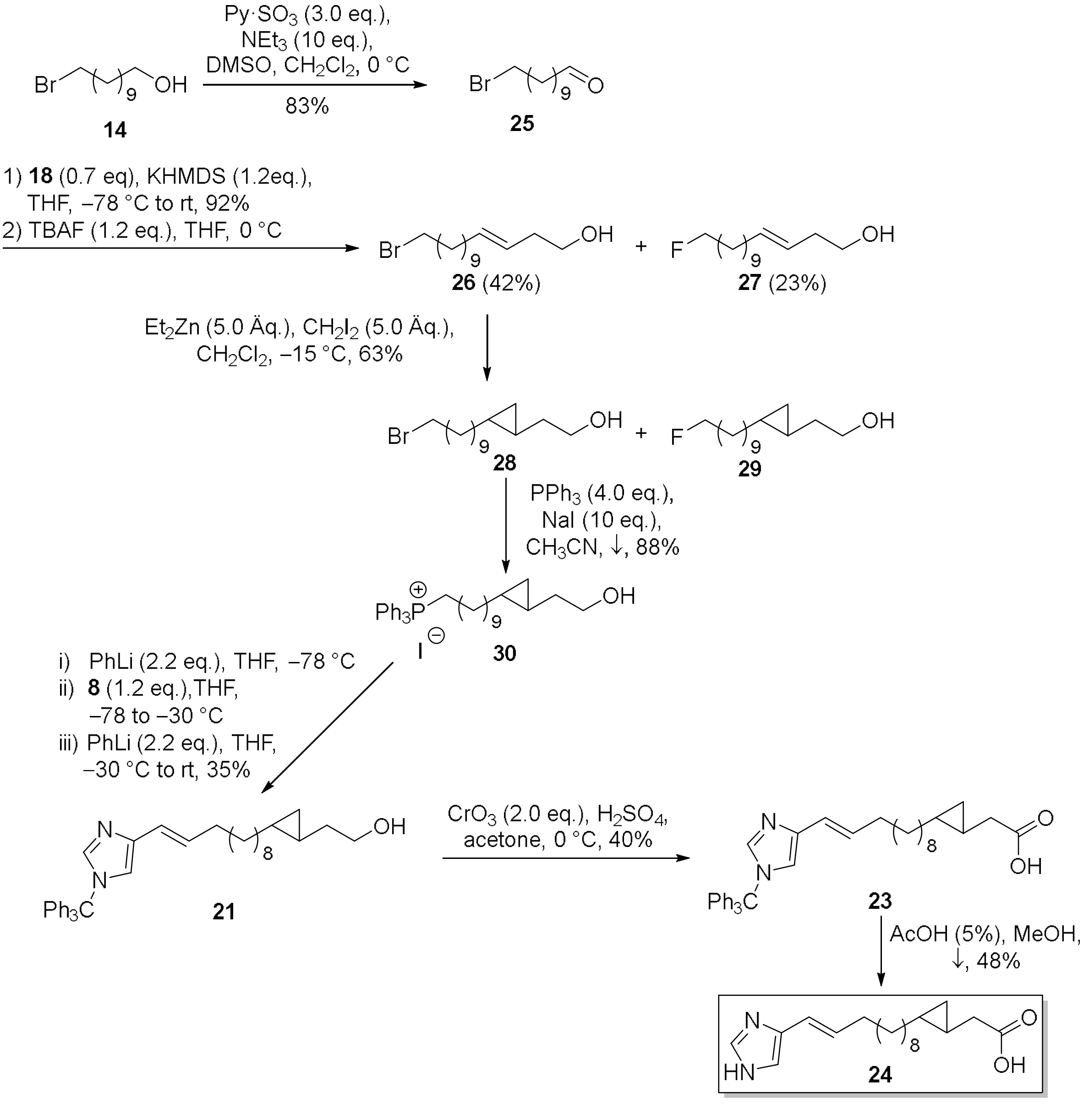

Scheme S2. Second, improved synthesis of imidacin B1 (**24**), not yet discovered as natural product.

cis-Imidacin A1 (**38**) was synthesized in a similar manner as imidacin A1, but using (*Z*)-14-bromotetradec-2-enoate (**33**) as precursor of the cyclopropantion, thus ensuring a *cis*-configured cyclopropane unit in **38** (Scheme S3). The *cis*-configured double bond was introduced by the Still-Gennari variant of the Horner-Wadsworth-Emmons reaction. As can be seen in the accompanying NMR spectra, the *cis*- and *trans*-imidacins can be clearly

differentiated using to the cyclopropane signals below 1 ppm. Natural imidacins are indeed *trans*-configured.

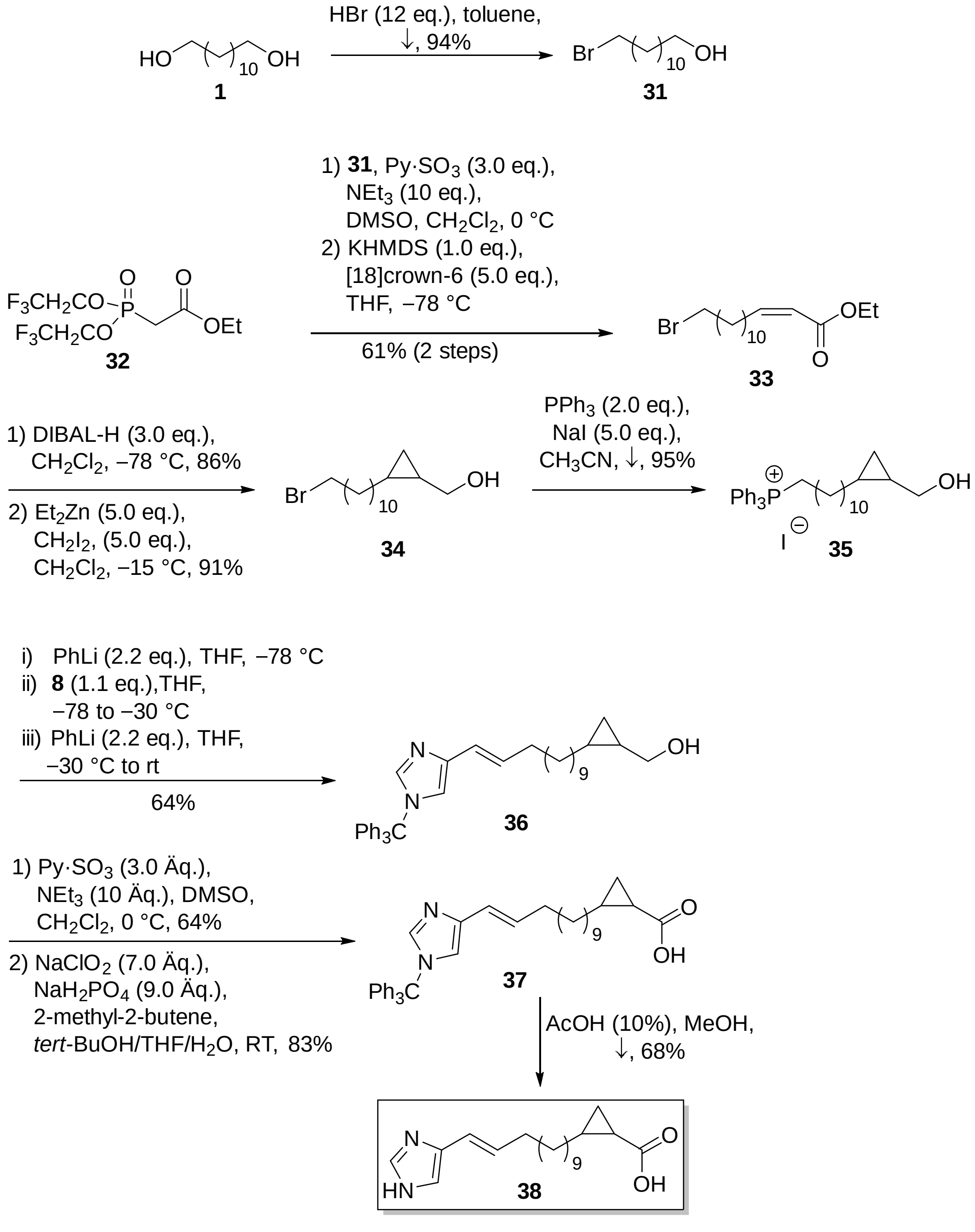

Scheme S3. The synthesis of imidacin *cis*-A1 (**38**). The synthesis is analogous to that of imidacin A1 described in the main text, using a *Z*-configured double bond for cyclopropanation.

Imidacin A2 was synthesized as described for imidacin A1, starting from 14-bromotetradecan-1-ol instead of 12-bromododecan-1-ol (Scheme S4).

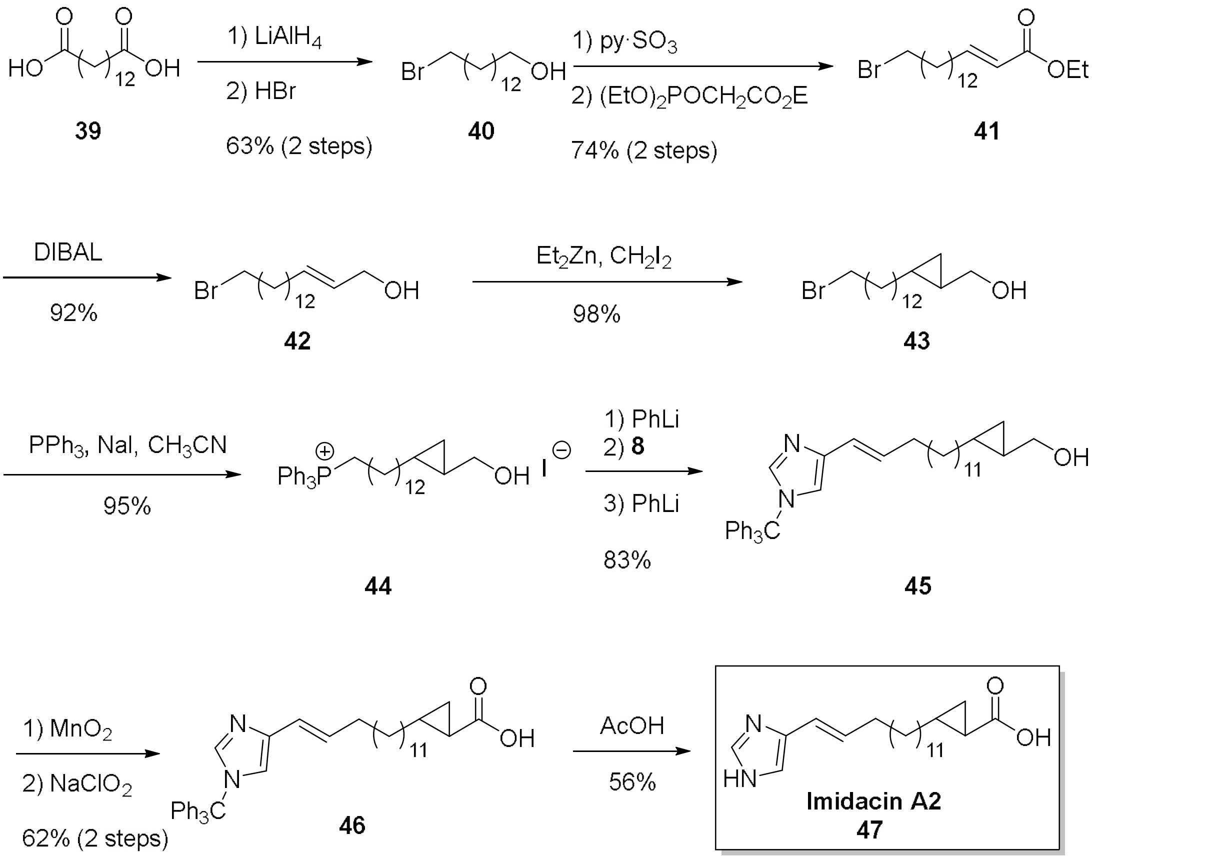

Scheme S4. The synthesis of imidacin A2 (**47**). The synthesis is analogous to that of imidacin A1 described in the main text.

4 Proposed biosynthesis of bulbimidazols

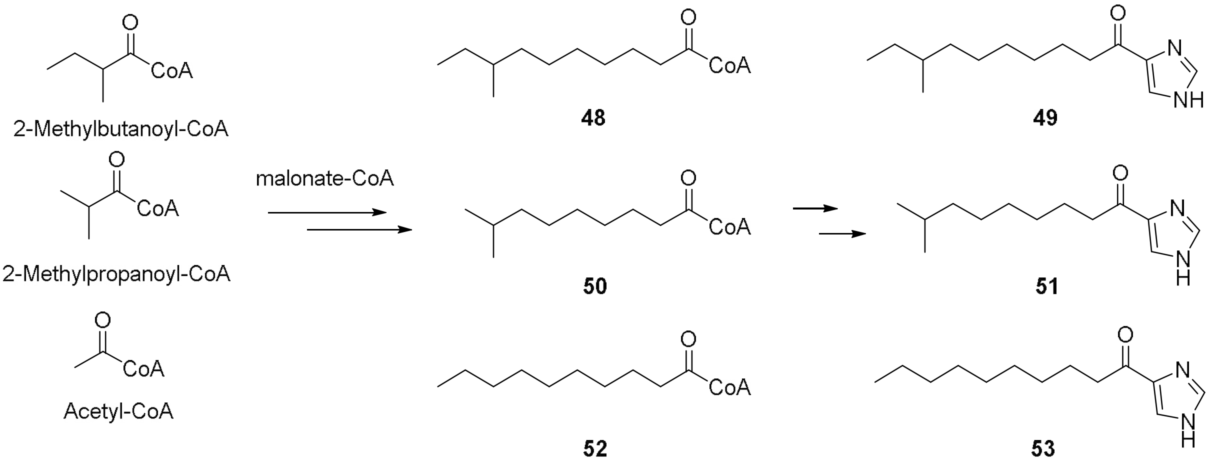

Scheme S5: Most likely biosynthetic pathway to bulbimidazoles **49**, **51**, and **53** according to Ul Karim et al.[S5] The typical bacterial fatty acid biosynthetic starter units 2-methylbutanoyl-CoA, 2-methylpropanoyl-CoA, and acetyl-CoA are elongated to the necessary chain length by malonyl-CoA to arrive at the acyl-CoA compounds **48**, **50**, and **52**. These CoA esters are the

most likely precursors that are connected via an unknown mechanism to an imidazole precurosr of are further modified to form the bulbimidazoles. The difference to the imidacin biosynthesis is that the imidazoles are introduced at the end of the pathway, in contrast to the imidacin biosynthesis, in which urocanoate serves as starter unit.

5 Experimental Part

5.1 General methods

The reactions were performed with dry solvents. All reactions involving water-sensitive chemicals were carried out in overnight oven dried (100°C) clean glass equipment which was further dried with a heat gun before the start of the reaction under a nitrogen atmosphere and magnetic stirring. TLC was performed on 0.2 mm pre-coated plastic sheets of Polygram® SIL G/UV254 plates. Detection of compounds was performed by immersion in 10% ethanol solution of phosphomolybdic acid, followed by gentle heating with a heat gun. Flash chromatography was performed on silica gel M60 (0.04-0.063 mm, 230-400 mesh ASTM) under pressure with the eluent mentioned in the respective procedures. GC/MS analyses were performed on a HP 6890 gas chromatograph coupled to an MSD 5973 (EI 70 eV) (Hewlett Packard). Separation was performed on a HP5-MS (30 m x 0.25 mm I.D. x 0.25 µm). Instrument parameters were adjusted as follows: inlet pressure 77.1 kPa; He 23.3 ml/min; injector 250 °C; electron energy 70eV. ESI-MS and HR-ESI spectra were obtained with an LTQ Orbitrap Velos spectrometer by direct infusion with a Proxeon nanospray ion source. A Exactive GC orbitrap mass spectrometer (ThermoScientific, Bremen, Germany) was used for GC/HR-MS. The resolution was set to 60,000 (FWHM; instrument setting at 200 u). Mass range was 50–650 u and 2 micro scans were averaged per data scan. Automated gain control (AGC target) was set to 1×10^6 and maximum inject time was set to “auto.” Auxiliary temperatures were set to 270 °C for both transfer lines 1 and 2 and the temperature of the EI source was set to 220 °C. EI was performed at 70 eV energy in positive mode. Helium (carrier gas) and nitrogen (supply for the C-Trap) were equipped with gas purification cartridges to trap moisture and organic impurities of the gases (Thermo Scientific, Bremen, Germany). The column bleed ion at 207.03235 u was used as lock mass for internal mass calibration of the data. For chemical ionization in positive mode (CIP), methane (99.995%) was used as CI-gas at a flow rate of 1.5 mL/min. IR spectra were recorded with Bruker Tensor 27 spectrometer with diamante-ATR. IR bands are reported in cm^{-1}. 1D and 2D NMR spectra were obtained with Bruker DRX-400 (^{1}H 400 MHz, ^{13}C 101 MHz) and Bruker AV II-600 (^{1}H 600.1 MHz, ^{13}C 150.9 MHz) instruments. Chemical shifts are reported in ppm relative to tetramethylsilane as an internal standard (δ = 0). All compounds were dissolved in the respective deuterated solvents mentioned. Optical rotations were obtained with a Dr. Kernchen Propol Digital automatic polarimeter. UV/VIS spectra were recorded with a Varian Cary 100 Bio spectrometer.

5.2 Bioactivity of imidacins

Imidacins *rac*-A1, *cis*-A1, (*S*,*S*)-A1, (*R*,*R*)-A1, and A2 were dissolved in DMSO and their minimum inhibitory concentrations (MIC) were tested in standard microbroth dilution assays as described earlier.[S6] Growth inhibition was tested against the following strains: *Bacillus subtilis* DSM-10, *Chromobacterium violaceum* DSM-30191, *Escherichia coli* DSM-1116, TolC-deficient *E. coli, E. coli* DSM-26863 (TolC3 deficient),[S7] *Micrococcus luteus* DSM-1790, *Mycobacterium smegmatis* mc^{2}155, *Pseudomonas aeruginosa* PA14, *Staphylococcus aureus* Newman, *Candida albicans* DSM-1665, *Mucor hiemalis* DSM-2656, and *Pichia anomala* DSM-

6766. Serial dilutions of samples were prepared from DMSO stock solutions in sterile 96-well plates, the cell suspension was added, and microorganisms were grown for 16–48 h at their optimal growth temperature at either 30 °C or 37 °C. The reported MIC values are the lowest concentration without visible growth. All values higher than 128 μg/mL were considered inactive.

Imidacin *rac*-A1 and A2 were inactive against all tested strains. Imidacin *cis*-A1 showed low activity with a MIC of 64 μg/mL against *M. luteus* and *B. subtilis*, and moderated activity of 16 μg/mL against the two TolC deficient *E. coli* strains. Imidacin (*S*,*S*)-A1 showed low activity of 64 μg/mL against these *E. coli* strains, while the activity of imidacin (*R*,*R*)-A1 was slightly higher with 16 μg/mL.

5.3 General procedures

General procedure 1: Synthesis of bromoalcohols

Following the reported procedure,[S8] HBr (12 eq., 48% in H_2O) was added to a solution of the respective diol (1.0 eq.) in toluene at 0 °C and the resulting mixture was heated at reflux until complete consumption of the starting material (TLC monitoring). The solution was allowed to cool down to r.t., followed by addition of sat. NaCl solution. The layers were separated and the aqueous layer was extracted with *n*-pentane (3x). The combined organic layers were dried with $MgSO_4$, filtered and the solvent was removed under reduced pressure. The crude product was purified by flash chromatography on silica gel.

General procedure 2: Parikh-Doering oxidation

According to a reported procedure,[S9] Py·SO_3 (3.0 eq.) was dissolved in dry DMSO and stirred at r.t. for 20 min. This solution was added to a solution of the respective bromo alcohol (1.0 eq.) in dry CH_2Cl_2 and triethylamine (10 eq.) at 0 °C. After complete consumption of alcohol (TLC monitoring) the reaction was quenched with water and the layers separated. The aqueous layer was extracted with CH_2Cl_2 (3x) and the combined organic layers were washed with sat. NaCl solution. After drying with $MgSO_4$, filtration and evaporation of the solvent under reduced pressure, the residue was taken up in *n*-pentane and filtered again. Removal of the solvent under reduced pressure gave crude aldehyde, which was used in the next step without further purification.

General procedure 3: Horner-Wadsworth-Emmons reaction

Based on a reported procedure,[S10] sodium hydride (1.5 eq., 60% in mineral oil) was suspended in Et_2O and cooled down to 0 °C. Triethyl phosphonoacetate (2.5 eq.) was slowly added and the reaction mixture was stirred for 0.5 h at r.t. The solution was cooled down to 0 °C, a solution of freshly prepared crude aldehyde in Et_2O was added and the mixture was stirred for 1 h at r.t. After addition of water, the layers were separated and the aqueous layer was extracted with Et_2O (3x). The combined organic layers were dried with $MgSO_4$ and the solvent was removed under reduced pressure. The residue was purified by flash chromatography on silica gel. Only the desired (*E*)-isomer was obtained.

General procedure 4: DIBAL-*H* reduction of esters to alcohols

Referring to reported a procedure,[S11] DIBAL-*H* solution (3.0 eq., 1.0 M in CH_2Cl_2) was added dropwise to a stirred solution of the respective ester (1.0 eq.) in dry CH_2Cl_2 at −78 °C and the solution slowly returned to r.t. After complete consumption of the ester, methanol was slowly added at −5 °C, followed by 2 M HCl solution. The layers were separated and the aqueous layer was extracted with CH_2Cl_2 (3x). The combined organic layers were dried with $MgSO_4$ and the solvent was removed under reduced pressure. The residue was subjected to flash chromatography on silica gel.

General procedure 5: Simmons-Smith cyclopropanation

According to the literature,[S12] diethylzinc solution (5.0 eq., 1.0 M in *n*-hexane) followed by methylene iodide (5.0 eq.) was added to a solution of an allyl/homoallyl alcohol (1.0 eq.) in dry CH_2Cl_2 at −15 °C. The mixture then was allowed to return to r.t. over a 3-4 h period. The sat. NH_4Cl solution was added, the layers were separated and the aqueous layer was extracted

with CH_2Cl_2 (3x). The combined organic layers were washed with sat. $NaHCO_3$ solution, sat. NaCl solution and water followed by drying with $MgSO_4$. After removal of the solvent under reduced pressure, the residue was purified by flash chromatography on silica gel.

General procedure 6: Preparation of phosphonium salts
Similar to a reported procedure,[S13,S14] a solution of an alkyl bromide (1.0 eq.) in CH_3CN was treated with triphenylphosphane (2.0 eq.) and sodium iodide (5.0 eq.). After heating at reflux for 17-23 h, the mixture was cooled down to r.t. and the solvent was removed under reduced pressure. The residue was extracted several times with CH_2Cl_2 and filtered until the combined organic layers were again concentrated under reduced pressure. Pure phosphonium salt was obtained after purification by flash chromatography on silica gel.

General-procedure 7: Wittig-Schlosser reaction
Basing on a reported procedure,[S15] a phosphonium salt (1.0 -1.1 eq.) was dissolved in dry THF, cooled down to −78 °C and treated with phenyllithium solution (2.0 -2.2 eq., 1.8 M in Bu_2O). The resulting mixture was allowed to return to r.t. and after 2 h again cooled down to −78 °C. A solution of an aldehyde (1.0-1.2 eq.) in dry THF was added slowly, the mixture was allowed to reach −30 °C and after additional 30 min at −30 °C again phenyllithium solution (2.2 eq., 1.8 M in Bu_2O) was added. The reaction mixture was slowly returned to r.t. overnight followed by quenching with water and separating the layers. The aqueous layer was extracted with EtOAc (3x), the combined organic phases dried with $MgSO_4$, concentrated under reduced pressure and the residue was purified by flash chromatography on silica gel. Only desired (*E*)-alkene was isolated, nevertheless minor amounts of the (*Z*)-alkene were also formed.

General procedure 8: Pinnick oxidation
Basing on reported procedures,[S16] a freshly prepared solution of $NaClO_2$ (7.0 eq.) and NaH_2PO_4 (9.0 eq.) in water was slowly added at r.t. to a solution of an aldehyde (1.0 eq.) in *tert*-BuOH/2-methyl-2-butene (3:1). The reaction mixture was stirred at r.t. for 0.5-6.5 h (TLC monitoring) until the organic layer was separated and the aqueous layer was extracted with CH_2Cl_2 (3x). The combined organic layers were washed with sat. NaCl solution and dried with $MgSO_4$. The solvent was removed under reduced pressure and the residue purified by flash chromatography on silica gel.

General procedure 9: Trityl deprotection
According to a literature procedure,[S17] starting material (1.0 eq.) was heated to reflux in 10% acetic acid in methanol for 2.5-6 h (TLC monitoring). The solvent was removed under reduced pressure and the residue subjected to flash chromatography on silica gel.

5.4 Synthesis of Imidacin A1

1-Trityl-1*H*-imidazole-4-carbaldehyde (8)

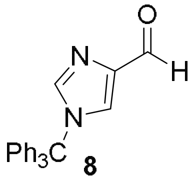

8

A solution of 1*H*-Imidazole-4-carbaldehyde (6.91 g, 71.9 mmol, 1.0 eq.) in dry CH_2Cl_2 (140 mL) at r.t. was treated with triethylamine (10.0 mL, 71.9 mmol, 1.0 eq.) and triphenylmethyl chloride (20.1 g, 72.1 mmol, 1.0 eq.) and the resulting mixture stirred at r.t. for 24 h. The precipitate was filtered off and consecutively washed with EtOAc and water. The layers were separated, the organic layer combined with the reaction solution, and washed with sat. NaCl solution and water. After removal of the solvent under reduced pressure, aldehyde **8** (19.1 g, 56.4 mmol, 78%) was obtained as a colorless solid.[S3]

TLC [silica gel, *n*-pentane/EtOAc (2:1)]: R_f = 0.28.

^{1}H-NMR (300 MHz, $CDCl_3$): δ = 9.88 (s, 1H, C*H*O), 7.62 (d, ^4J = 1.3 Hz, 1H, NC*H*N), 7.54 (d, ^4J = 1.3 Hz, 1H, NCC*H*N), 7.40-7.33 (m, 9H, *m*-/*p*-Ph-C*H*), 7.15-7.09 (m, 6H, *o*-Ph-C*H*).

^{13}C-NMR (75 MHz, $CDCl_3$): δ = 186.5 (*C*HO), 141.5 (3C, *ipso*-Ph-*C*), 140.8 (N*C*CHN), 140.6 (N*C*HN), 129.6 (6C, *o*-Ph-*C*H), 128.5 (3C, *p*-Ph-*C*H), 128.3 (6C, *m*-Ph-*C*H), 126.8 (NC*C*HN), 76.3 (*C*Ph_3).

IR (ATR): $\tilde{\nu}$ = 3136 (w), 3055 (w), 2816 (w), 2754 (w), 1691 (s), 1535 (m), 1490 (m), 1443 (m), 136 (w), 127 (w), 1227 (w), 1183 (w), 1154 (w), 1119 (m), 109 (m), 1036 (w), 1000 (w), 978 (w), 907 (w), 866 (w), 844 (w), 750 (s), 698 (s), 673 (m), 659 (m), 637 (m), 556 (w).

UV/VIS (CH_2Cl_2): λ_{max} (log ε) = 260 (4.17), 227 (3.99), 222 (3.89).

MS (ESI+): *m/z* (%) = 699.27 (100) $[2M+Na]^+$, 361.13 (29) $[M+Na]^+$, 243.12 (79) $[CPh_3]^+$.

HRMS (ESI) *m/z*: $[M + Na]^+$ Calcd for $C_{23}H_{18}N_2NaO$ 361.1311; Found 361.1314

12-Bromododecan-1-ol

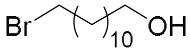

This alcohol was prepared following general procedure 1:
Dodecane-1,12-diol (**1**, 6.07 g, 30.0 mmol, 1.0 eq.) in toluene (100 mL).
HBr (42.0 mL, 360 mmol, 12 eq., 48% in H_2O).
Flash chromatography [silica gel, *n*-pentane/EtOAc (10:1) to (2:1)].
12-Bromododecan-1-ol (7.49 g, 28.2 mmol, 95%), colorless solid.
The spectroscopic data matched those reported earlier.[S18]

TLC (silica gel, *n*-pentane/EtOAc = 10:1): R_f = 0.23.

^{1}H-NMR (400 MHz, $CDCl_3$): δ = 3.63 (t, ^3J = 6.7 Hz, 2H, C*H*$_2$OH), 3.41 (t, ^3J = 6.9 Hz, 2H, C*H*$_2$Br), 1.90-1.81 (m, 2H, C*H*$_2$CH$_2$Br), 1.67-1.52 (m, 3H, C*H*$_2$CH$_2$O*H*), 1.47-1.23 (m, 16H, (C*H*$_2$)$_8$).

^{13}C-NMR (100 MHz, $CDCl_3$): δ = 63.0 (*C*H$_2$OH), 34.0 (*C*H$_2$Br), 32.8 (*C*H$_2$CH$_2$Br), 32.7 (*C*H$_2$CH$_2$OH), 29.5 (3C, *C*H$_2$), 29.4 (2C, *C*H$_2$), 28.7 (*C*H$_2$), 28.1 (*C*H$_2$), 25.7 (*C*H$_2$).

IR (ATR): $\tilde{\nu}$ = 3391 (m), 3323 (w), 2918 (s), 2849 (s), 1463 (m), 1381 (w), 1263 (w), 1235 (w), 1209 (w), 1137 (w), 1052 (s), 1027 (m), 997 (m), 970 (m), 758 (w), 727 (m), 643 (s).

UV/VIS (CH_2Cl_2): λ_{max} (log ε) = 228 (2.01).

MS (EI): *m/z* (%) = 264/266 (missing) [M^+], 162/164 (8/8), 148/150 (16/16), 135/137 (7/8), 111 (8), 109 (5), 97 (35), 6 (5), 84 (6), 83 (42), 82 (17), 81 (9), 70 (13), 69 (62), 68 (26), 67 (15), 57 (19), 56 (23), 55 (100), 54 (12), 53 (7).

Ethyl (*E*)-14-bromotetradec-2-enoate

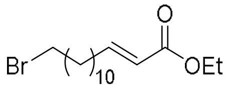

This Ester was prepared following general procedures 2 and 3:
12-Bromododecan-1-ol (5.01 g, 18.9 mmol, 1.0 eq.) in dry CH_2Cl_2 (100 mL).
Triethylamine (26.3 mL, 189 mmol, 10 eq.).
Py·SO_3 (9.02 g, 56.7 mmol, 3.0 Eq.) in dry DMSO (50 mL).
The aldehyde was used directly in the next step in dry Et_2O (60 mL).

NaH (1.13 g, 28.3 mmol, 1.5 eq., 60% in mineral oil) in dry Et_2O (80 mL).
Triethyl phosphonoacetate (9.40 mL, 47.4 mmol, 2.5 eq.).
Flash chromatography [silica gel, *n*-pentane/Et_2O (50:1) to (20:1)].
Ethyl (*E*)-14-bromotetradec-2-enoate (4.56 g, 13.7 mmol, 72% over 2 steps), colorless oil.

TLC (silica gel, *n*-pentane/Et_2O = 50:1): R_f = 0.48.

^{1}H-NMR (400 MHz, $CDCl_3$): δ = 6.7 (td, ^3J = 7.0 Hz, 15.6 Hz, 1H, C*H*CHCO$_2$Et), 5.81 (td, ^4J = 1.6 Hz, ^3J = 15.6 Hz, 1H, C*H*CO$_2$Et), 4.18 (q, ^3J = 7.1 Hz, 2H, OC*H*$_2$CH$_3$), 3.41 (t, ^3J = 6.9 Hz, 2H, C*H*$_2$Br), 2.23-2.15 (m, 2H, C*H*$_2$CHCHCO$_2$Et), 1.89-1.81 (m, 2H, C*H*$_2$CH$_2$Br), 1.50-1.23 (m, 19H, (C*H*$_2$)$_8$, OCH$_2$C*H*$_3$).

^{13}C-NMR (100 MHz, $CDCl_3$): δ = 166.7 (*C*O$_2$Et), 149.4 (*C*HCHCO$_2$Et), 121.2 (*C*HCO$_2$Et), 60.1 (O*C*H$_2$CH$_3$), 34.0 (*C*H$_2$Br), 32.8 (*C*H$_2$CH$_2$Br), 32.2 (*C*H$_2$CHCHCO$_2$Et), 29.4 (3C, *C*H$_2$), 29.3 (*C*H$_2$), 29.1 (*C*H$_2$), 28.7 (*C*H$_2$), 28.1 (*C*H$_2$), 28.0 (*C*H$_2$), 14.3 (OCH$_2$*C*H$_3$).

IR (ATR): $\tilde{\nu}$ = 235 (m), 294 (m), 1719 (s), 1654 (m), 1463 (w), 1367 (w), 1308 (m), 1265 (m), 1177 (m), 1129 (w), 107 (w), 1042 (m), 980 (m), 98 (w), 721 (w), 646 (w), 563 (w).

UV/VIS (CH_2Cl_2): λ_{max} (log ε) = 229 (3.41).

MS (EI): *m/z* (%) = 332/334 (<1) [M^+], 287/289 (9/10), 244/246 (6/5), 123 (9), 115 (5), 114 (5), 110 (5), 109 (17), 107 (9), 101 (21), 99 (18), 98 (6), 97 (12), 7 (13), 6 (24), 4 (6), 88 (12), 86 (9), 9 (6), 84 (9), 83 (14), 82 (12), 81 (43), 80 (5), 79 (11), 73 (16), 71 (5), 70 (7), 69 (28), 68 (30), 67 (33), 65 (5), 57 (20), 56 (12), 55 (100), 54 (16), 53 (25), 45 (12), 43 (49), 42 (21), 41 (90), 40 (10), 39 (34).

HRMS (CI) *m/z*: [M + H]$^+$ Calcd for $C_{16}H_{30}BrO_2$ 333.1429; Found 333.1427

(*E*)-14-Bromotetradec-2-en-1-ol (3)

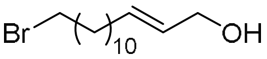

3

Allyl alcohol **3** was prepared following general procedure 4:
Ethyl (*E*)-14-bromotetradec-2-enoate (9.76 g, 29.3 mmol, 1.0 eq.) in dry CH_2Cl_2 (300 mL).
DIBAL-*H* (87.9 mL, 87.9 mmol, 3.0 eq., 1.0 M in CH_2Cl_2).
Flash chromatography [silica gel, CH_2Cl_2].
Alcohol **3** (7.90 g, 27.1 mmol, 94%) as a colorless oil.

TLC (silica gel, CH_2Cl_2 100%): R_f = 0.34.

^{1}H-NMR (400 MHz, $CDCl_3$): δ = 5.74-5.59 (m, 2H, C*HCH*CH_2OH), 4.08 (d, ^3J = 5.5 Hz, 2H, C*H*$_2$OH), 3.41 (t, ^3J = 6.9 Hz, 2H, C*H*$_2$Br), 2.08-2.00 (m, 2H, C*H*$_2$CHCHCH$_2$OH), 1.90-1.81 (m, 2H, C*H*$_2$CH$_2$Br), 1.48-1.22 (m, 16H, (C*H*$_2$)$_8$).

^{13}C-NMR (100 MHz, $CDCl_3$): δ = 133.5 (*C*HCHCH$_2$OH), 128.8 (*C*HCH$_2$OH), 63.8 (*C*H$_2$OH), 34.0 (*C*H$_2$Br), 32.8 (*C*H$_2$CH$_2$Br), 32.2 (*C*H$_2$CHCHCH$_2$OH), 29.5 (2C, *C*H$_2$), 29.4 (2C, *C*H$_2$), 29.1 (2C, *C*H$_2$), 28.7 (*C*H$_2$), 28.1 (*C*H$_2$).

IR (ATR): $\tilde{\nu}$ = 3322 (br), 232 (s), 292 (s), 1670 (w), 1462 (m), 1251 (w), 1089 (m), 1000 (m), 78 (s), 722 (m), 646 (m), 561 (m).

UV/VIS (CH_2Cl_2): λ_{max} (log ε) = 228 (2.10), 221 (1.74).

MS (EI): *m/z* (%) = 290/292 (missing) [M^+], 272/274 (5/5), 123 (5), 109 (22), 107 (13), 97 (8), 7 (19), 6 (31), 4 (11), 91 (11), 83 (14), 82 (36), 81 (65), 80 (17), 79 (57), 78 (6), 77 (23), 70 (6), 69 (26), 68 (57), 67 (100), 66 (16), 65 (24), 57 (26), 56 (12), 55 (89), 54 (55), 53 (36), 52 (8), 51 (9).

HRMS (CI) *m/z*: [M-18-H + H]$^+$ Calcd for $C_{14}H_{24}Br$ 271.1061; Found 271.1055

((1*RS*,2*RS*)-2-(11-Bromoundecyl)cyclopropyl)methanol (4)

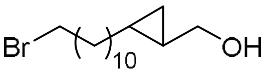

4

Alcohol **4** was prepared following general procedure 5:
Allyl alcohol **3** (1.60 g, 5.49 mmol, 1.0 eq.) in dry CH_2Cl_2 (60 mL).
Diethylzinc solution (27.5 mL, 27.5 mmol, 5.0 eq., 1.0 M in *n*-hexane).
Methylene iodide (2.20 mL, 4.7 mmol, 5.0 eq.).
Flash chromatography [silica gel, CH_2Cl_2].
Alcohol **4** (1.57 g, 5.14 mmol, 95%) as colorless oil.

TLC (silica gel, CH_2Cl_2 100%): R_f = 0.29.

^{1}H-NMR (400 MHz, $CDCl_3$): δ = 3.49-3.38 (m, 4H, C*H*$_2$OH, C*H*$_2$Br), 1.90-1.81 (m, 2H, C*H*$_2$CH$_2$Br), 1.47-1.18 (m, 18H, (C*H*$_2$)$_9$), 0.89-0.78 (m, 1H, C*H*CHCH$_2$OH), 0.64-0.54 (m, 1H, C*H*CH$_2$OH), 0.39-0.27 (m, 2H, CHC*H*$_2$CH).

^{13}C-NMR (100 MHz, $CDCl_3$): δ = 67.2 (*C*H$_2$OH), 34.0 (*C*H$_2$Br), 33.5 (*C*H$_2$CHCHCH$_2$OH), 32.8 (*C*H$_2$CH$_2$Br), 29.6 (2C, *C*H$_2$), 29.5 (2C, *C*H$_2$), 29.4 (2C, *C*H$_2$), 28.7 (*C*H$_2$), 28.1 (*C*H$_2$), 21.1 (*C*HCHCH$_2$OH), 17.1 (*C*HCH$_2$OH), 9.9 (CH*C*H$_2$CH).

IR (ATR): $\tilde{\nu}$ = 3373 (w), 3270 (w), 3067 (w), 297 (w), 2916 (s), 2849 (s), 1466 (m), 1441 (m), 1375 (w), 1235 (w), 1210 (w), 1154 (w), 1020 (s), 905 (w), 874 (w), 95 (w), 807 (w), 778 (w), 759 (w), 721 (m), 644 (m), 612 (m).

UV/VIS (CH_2Cl_2): λ_{max} (log ε) = 226 (1.81), 222 (1.58).

MS (EI): *m/z* (%) = 304/306 (missing) [M^+], 286/288 (2/2), 263/265 (7/6), 189/191 (9/8), 175/177 (6/7), 165 (8), 164 (7), 163 (6), 162 (7), 151 (5), 150 (12), 149 (6), 148 (12), 137 (11), 135 (8), 123 (21), 111 (14), 110 (10), 109 (44), 107 (7), 98 (7), 97 (63), 7 (42), 6 (57), 4 (6), 9 (8), 84 (11), 83 (84), 82 (63), 81 (77), 80 (8), 79 (20), 77 (5), 71 (21), 70 (15), 69 (77), 68 (58), 67 (71), 66 (6), 65 (5), 58 (5), 57 (57), 56 (17), 55 (100), 54 (35), 53 (13), 44 (11), 43 (30), 42 (10), 41 (66), 39 (14).

HRMS (CI) *m/z*: $[M - H]^+$ Calcd for $C_{15}H_{28}BrO$ 303.1324; Found 331.1319

(11-((1*RS*,2*RS*)-2-(Hydroxymethyl)cyclopropyl)undecyl)triphenylphosphonium iodide (5)

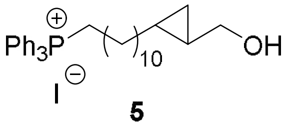

5

Phosphonium salt **5** was prepared following general procedure 6:
Bromoalcohol **4** (1.86 g, 6.09 mmol, 1.0 eq.) in CH_3CN (80 mL).
Triphenylphosphane (3.20 g, 12.2 mmol, 2.0 eq.).
Sodium iodide (4.57 g, 30.5 mmol, 5.0 eq.).
Flash chromatography [silica gel, CH_2Cl_2, then CH_2Cl_2/MeOH (5:1)].
Phosphonium salt **5** (3.57 g, 5.81 mmol, 96%) as colorless oil.

TLC (silica gel, CH_2Cl_2/MeOH = 20:1): R_f = 0.23.

^{1}H-NMR (400 MHz, $CDCl_3$): δ = 7.86-7.77 (m, 9H, *m*-/*p*-Ph-C*H*), 7.77-7.70 (m, 6H, *o*-Ph-C*H*), 3.65-3.52 (m, 2H, PCH_2), 3.49-3.36 (m, 2H, CH_2OH), 1.71 (s, br, 1H, O*H*), 1.68-1.58 (m, 4H, $PCH_2CH_2CH_2$), 1.39-1.13 (m, 16H, $(CH_2)_8$), 0.86-0.77 (m, 1H, C*H*CHCH_2OH), 0.63-0.54 (m, 1H, C*H*CH_2OH), 0.38-0.24 (m, 2H, CHCH_2CH).

^{13}C-NMR (100 MHz, $CDCl_3$): δ = 135.0 (3C, *p*-Ph-CH), 133.5 (6C, d, *J* = 10.0 Hz, *o*-Ph-*C*H), 130.5 (6C, d, *J* = 12.4 Hz, *m*-Ph-*C*H), 117.9 (3C, d, *J* = 86.0 Hz, *ipso*-Ph-*C*), 66.9 (*C*H_2OH), 33.4 (*C*H_2CHCHCH_2OH), 30.3 (d, *J* = 15.6 Hz, $PCH_2CH_2CH_2$), 29.4 (2C, *C*H_2), 29.3 (2C, *C*H_2), 29.1 (*C*H_2), 29.0 (2C, *C*H_2), 22.9 (d, *J* = 50.0 Hz, P*C*H_2), 22.4 (d, *J* = 4.5 Hz, PCH_2CH_2), 21.0 (*C*HCHCH_2OH), 17.0 (*C*HCH_2OH), 9.8 (CH*C*H_2CH).

IR (ATR): $\tilde{\nu}$ = 3369 (br), 3055 (w), 24 (w), 231 (m), 291 (m), 1587 (w), 1484 (w), 1462 (w), 1437 (m), 1317 (w), 1189 (w), 1111 (m), 1028 (m), 97 (m), 789 (w), 722 (s), 688 (s), 532 (s).

UV/VIS (MeOH): λ_{max} (log ε) = 274 (3.38), 267 (3.46), 222 (4.55), 203 (4.65).

MS (ESI+): *m/z* (%) = 487.3 (100) $[M{-}I]^+$.

HRMS (ESI) *m/z*: $[M - I]^+$ Calcd for $C_{33}H_{44}OP$ 487.3124; Found 487.3126

((1*RS*,2*RS*)-2-((*E*)-12-(1-Trityl-1*H*-imidazol-4-yl)dodec-11-en-1-yl)cyclopropyl)-methanol (6)

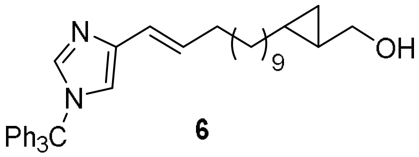

6

Alcohol **6** was prepared following general procedure 7:
Phosphonium salt **5** (0.97, 1.58 mmol, 1.0 eq.) in dry THF (40 mL).
Phenyllithium solution (1.90 mL, 3.42 mmol, 2.2 eq., 1.8 M in Bu_2O).
Aldehyde **8** (580 mg, 1.71 mmol, 1.1 eq.) in dry THF (20 mL).
Phenyllithium solution (1.90 mL, 3.42 mmol, 2.2 eq., 1.8 M in Bu_2O).
Flash chromatography [silica gel, CH_2Cl_2/MeOH (100:1)].
Alcohol **6** (610 mg, 1.12 mmol, 71%) as yellowish solid.

TLC (silica gel, CH_2Cl_2/MeOH = 40:1): R_f = 0.27.

^{1}H-NMR (400 MHz, $CDCl_3$): δ = 7.36 (d, ^4J = 1.2 Hz, 1H, NC*H*N), 7.34-7.29 (m, 9H, *m*-/*p*-Ph-C*H*), 7.17-7.12 (m, 6H, *o*-Ph-C*H*), 6.66 (d, ^4J = 1.3 Hz, 1H, NCC*H*N), 6.34 (td, ^3J = 6.8 Hz, 15.7 Hz, 1H, NCCHC*H*), 6.20 (d, ^3J = 15.8 Hz, 1H, NCC*H*CH), 3.50-3.36 (m, 2H, CH_2OH), 2.18-2.10 (m, 2H, NCCHCHCH_2), 2.04 (s, br, 1H, O*H*), 1.48-1.17 (m, 18H, $(CH_2)_9$), 0.87-0.77 (m, 1H, C*H*CHCH$_2$OH), 0.62-0.53 (m, 1H, C*H*CH$_2$OH), 0.38-0.25 (m, 2H, CHCH_2CH).

^{13}C-NMR (100 MHz, $CDCl_3$): δ = 142.4 (3C, *ipso*-Ph-*C*), 139.5 (N*C*CHN), 138.9 (N*C*HN), 129.8 (NCCH*C*H), 129.7 (6C, *o*-Ph-*C*H), 128.0 (9C, *m*-/*p*-Ph-*C*H), 121.3 (NC*C*HCH), 118.0 (NC*C*HN), 75.2 (*C*Ph$_3$), 67.0 (*C*H$_2$OH), 33.5 (*C*H$_2$CHCHCH$_2$OH), 32.8 (NCCHCH*C*H$_2$), 29.5 (5C, *C*H$_2$), 29.3 (2C, *C*H$_2$), 29.2 (*C*H$_2$), 21.2 (*C*HCHCH$_2$OH), 17.1 (*C*HCH$_2$OH), 9.9 (CH*C*H$_2$CH).

IR (ATR): $\tilde{\nu}$ = 3250 (br), 3061 (w), 2976 (w), 2919 (m), 290 (m), 156 (w), 1490 (m), 1470 (m), 1445 (m), 1291 (w), 1229 (w), 1184 (w), 1158 (w), 1131 (m), 1084 (w), 1032 (m), 970 (m), 906 (w), 869 (w), 832 (w), 748 (s), 700 (s), 657 (m), 638 (m).

UV/VIS (MeOH): λ_{max} (log ε) = 240 (4.22), 203 (4.72).

MS (ESI+): *m/z* (%) = 1115.7 (7) $[2M+Na]^+$, 569.4 (56) $[M+Na]^+$, 547.4 (100) $[M+H]^+$, 243.1 (54) $[CPh_3]^+$.

HRMS (ESI) *m/z*: $[M + Na]^+$ Calcd for $C_{38}H_{46}N_2NaO$ 569.3502; Found 569.3502

(1*RS*,2*RS*)-2-((*E*)-12-(1-Trityl-1*H*-imidazol-4-yl)dodec-11-en-1-yl)cyclopropane-1-carbaldehyde

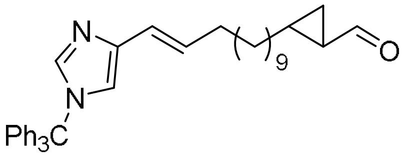

The title aldehyde was prepared following general procedure 2:
Alcohol **6** (361 mg, 0.66 mmol, 1.0 eq.) in dry CH_2Cl_2 (30 mL).
Triethylamine (0.3 mL, 6.62 mmol, 10 eq.).
Py·SO_3 (315 mg, 1.98 mmol, 3.0 eq.) in dry DMSO (10 mL).
Flash chromatography [silica gel, CH_2Cl_2/MeOH (400:1) to (200:1)].

Aldehyde **7** (188 mg, 0.35 mmol, 52%) as yellowish oily solid.

TLC (silica gel, $CHCl_3$/MeOH = 40:1): R_f = 0.40.

^{1}H-NMR (400 MHz, $CDCl_3$): δ = 8.99 (d, ^3J = 5.6 Hz, 1H, C*H*O), 7.37-7.35 (m, 1H, NC*H*N), 7.35-7.30 (m, 9H, *m*-/*p*-Ph-C*H*), 7.18-7.12 (m, 6H, *o*-Ph-C*H*), 6.66 (d, ^4J = 1.3 Hz, 1H, NCC*H*N), 6.34 (td, ^3J = 6.8 Hz, 15.6 Hz, 1H, NCCHC*H*), 6.21 (d, ^3J = 15.7 Hz, 1H, NCC*H*CH), 2.19-2.10 (m, 2H, NCCHCHCH_2), 1.65-1.58 (m, 1H, C*H*CHO), 1.51-1.19 (m, 20H, $(CH_2)_9$, C*H*CHCHO, CHC[*H*]$_2$CH), 0.6-0.90 (m, 1H, CHC[*H*]$_2$CH).

^{13}C-NMR (100 MHz, $CDCl_3$): δ = 201.1 (*C*HO), 142.4 (3C, *ipso*-Ph-*C*), 139.6 (N*C*CHN), 139.0 (N*C*HN), 129.8 (6C, *o*-Ph-*C*H), 129.7 (NCCH*C*H), 128.0 (9C, *m*-/*p*-Ph-*C*H), 121.4 (NC*C*HCH), 118.0 (NC*C*HN), 75.2 (*C*Ph_3), 32.8 (NCCHCH*C*H_2), 32.6 (*C*H_2CHCHCHO), 30.5 (*C*HCHO), 29.5 (4C, *C*H_2), 29.4 (*C*H_2), 29.2 (2C, *C*H_2), 29.1 (*C*H_2), 22.7 (*C*HCHCHO), 14.9 (CH*C*H_2CH).

IR (ATR): $\tilde{\nu}$ = 3059 (w), 3003 (w), 234 (m), 291 (m), 167 (m), 157 (w), 1491 (m), 1466 (w), 1445 (m), 1406 (w), 1297 (w), 1217 (w), 1183 (w), 1157 (w), 1129 (w), 109 (w), 1035 (w), 1001 (w), 973 (m), 905 (w), 868 (w), 826 (w), 749 (s), 699 (s), 656 (m), 638 (m).

UV/VIS (MeOH): λ_{max} (log ε) = 245 (4.10), 205 (4.70).

MS (ESI+): *m/z* (%) = 561.3 (13) $[M+Na]^+$, 545.4 (49) $[M+H]^+$, 243.1 (100) $[CPh_3]^+$.

HRMS (ESI) *m/z*: $[M + H]^+$ Calcd for $C_{38}H_{45}N_2O$ 545.3526, Found. 545.3525

(1*RS*,2*RS*)-2-((*E*)-12-(1*H*-Imidazol-4-yl)dodec-11-en-1-yl)cyclopropane-1-carboxylic acid (9)

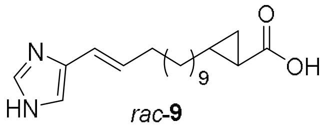

rac-**9**

Acid **9** was prepared following general procedure 8 and 9:
The corresponding aldehyde (160 mg, 0.29 mmol, 1.0 eq.) in *tert*-BuOH (18 mL) and 2-methyl-2-butene (6 mL).
$NaClO_2$ (186 mg, 2.06 mmol, 7.0 eq.) and NaH_2PO_4 (365 mg, 2.65 mmol, 9.0 eq.) in water (5 mL).
Acetic acid (2.00 mL) in MeOH (20 mL).
Flash chromatography [silica gel, $CHCl_3$/MeOH/NH_3 (40:10:1)].
Acid *rac*-**7** (56 mg, 0.18 mmol, 62% over 2 steps) as colorless solid.

TLC (silica gel, $CHCl_3$/MeOH/NH_3 = 40:10:1): R_f = 0.10.

^{1}H-NMR (600 MHz, CD_3OD): δ = 7.72 (s, 1H, NC*H*N), 6.97 (s, 1H, NCC*H*N), 6.27 (d, ^3J = 16.0 Hz, 1H, NCC*H*CH), 6.17 (td, ^3J = 6.9 Hz, 15.9 Hz, 1H, NCCHC*H*), 2.20-2.15 (m, 2H, NCCHCHCH_2), 1.50-1.23 (m, 20H, $(CH_2)_9$, C*H*C*H*CO_2H), 1.09-1.05 (m, 1H, CHC[*H*]$_2$CH), 0.70-0.66 (m, 1H, CHC[*H*]$_2$CH).

^{13}C-NMR (150 MHz, CD_3OD): δ = 179.2 (*C*O_2H), 136.3 (N*C*HN), 136.0 (N*C*CHN), 131.2 (NCCH*C*H), 120.0 (NC*C*HCH), 119.1 (NC*C*HN), 34.2 (*C*H_2CHCHCO_2H), 34.0 (NCCHCH*C*H_2), 30.7 (3C, *C*H_2), 30.6 (*C*H_2), 30.5 (*C*H_2), 30.4 (*C*H_2), 30.3 (2C, *C*H_2), 23.8 (*C*HCHCO_2H), 21.4 (*C*HCO_2H), 15.9 (CH*C*H_2CH).

IR (ATR): $\tilde{\nu}$ = 3143 (w), 3010 (w), 2915 (s), 2848 (s), 1649 (m), 1467 (w), 1404 (w), 1361 (w), 1253 (w), 1171 (w), 1101 (m), 1081 (m), 1037 (m), 78 (s), 879 (m), 824 (m), 746 (m), 721 (m), 635 (s).

UV/VIS (MeOH): λ_{max} (log ε) = 250 (4.08).

MS (ESI+): *m/z* (%) = 681.4 (16) $[2M-H+2Na]^+$, 637.5 (18) $[2M+H]^+$, 363.2 (28) $[M-H+2Na]^+$, 341.2 (58) $[M+Na]^+$, 319.2 (100) $[M+H]^+$, 301.2 (33) $[M-OH]^+$.

HRMS (ESI) *m/z*: $[M + H]^+$ Calcd for $C_{19}H_{31}N_2O_2$ 319.2380; Found 319.2382

5.5 Synthesis of Imidacin *cis*-A1

Ethyl (*Z*)-14-bromotetradec-2-enoate (33)

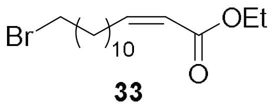

33

12-Bromododecanal was prepared following general procedure 2:
12-Bromododecan-1-ol (**31**, 984 mg, 3.71 mmol, 1.0 eq.) in dry CH_2Cl_2 (20 mL).
Triethylamine (5.16 mL, 37.1 mmol, 10 eq.).
Py·SO_3 (1.77 g, 11.1 mmol, 3.0 eq.) in dry DMSO (12 mL).
The crude aldehyde was directly used in the following Still-Gennari modification of the Horner-Wadsworth-Emmons olefination:
According to a reported procedure,[S15] 18-crown-6 (4.90 g, 18.5 mmol, 5.0 eq.) and phosphonate **32** (0.88 mL, 3.72 mmol, 1.0 eq.) were dissolved in dry THF (30 mL) and treated dropwise at −78 °C with KHMDS solution (7.42 mL, 3.71 mmol, 1.0 eq., 0.5 M in toluene). After stirring at −78 °C for additional 1 h, a solution of the freshly prepared aldehyde in dry THF (15 mL) was added and stirred for 10 min until the cooling bath was removed. The reaction mixture was slowly allowed to reach r.t. overnight (19 h). Saturated NH_4Cl solution (40 mL) was added and the layers were separated. The organic phase was washed with water and the aqueous phase extracted with Et_2O. The combined organic phases were dried with $MgSO_4$ and the solvent was removed under reduced pressure. The residue was purified by flash chromatography [silica gel, *n*-pentane/Et_2O (200:1 to 50:1)] yielding ester **33** (760 mg, 2.28 mmol, 61% over 2 steps) as a colorless oil.

TLC (silica gel, *n*-pentane/Et_2O = 100:1): R_f = 0.31.

^{1}H-NMR (400 MHz, $CDCl_3$): δ = 6.21 (td, ^3J = 7.5 Hz, 11.5 Hz, 1H, C*H*CHCO$_2$Et), 5.75 (td, ^4J = 1.7 Hz, ^3J = 11.5 Hz, 1H, C*H*CO$_2$Et), 4.17 (q, ^3J = 7.1 Hz, 2H, OC*H*$_2$CH$_3$), 3.40 (t, ^3J = 6.9 Hz, 2H, C*H*$_2$Br), 2.64 (ddd, ^4J = 1.7 Hz, ^3J = 7.5 Hz, 15.0 Hz, 2H, C*H*$_2$CHCHCO$_2$Et), 1.89-1.81 (m, 2H, C*H*$_2$CH$_2$Br), 1.47-1.24 (m, 19H, (C*H*$_2$)$_8$, OCH$_2$C*H*$_3$).

^{13}C-NMR (100 MHz, $CDCl_3$): δ = 166.5 (*C*O$_2$Et), 150.6 (*C*HCHCO$_2$Et), 119.6 (*C*HCO$_2$Et), 59.7 (O*C*H$_2$CH$_3$), 34.0 (*C*H$_2$Br), 32.8 (*C*H$_2$CH$_2$Br), 29.5 (2C, *C*H$_2$), 29.4 (2C, *C*H$_2$), 29.3 (*C*H$_2$), 29.0 (2C, *C*H$_2$), 28.7 (*C*H$_2$), 28.2 (*C*H$_2$), 14.3 (OCH$_2$*C*H$_3$).

IR (ATR): $\tilde{\nu}$ = 234 (m), 294 (m), 1719 (s), 1643 (w), 1462 (w), 1415 (w), 1387 (w), 1178 (s), 1034 (m), 820 (m), 722 (w), 645 (w), 564 (w).

UV/VIS (MeOH): λ_{max} (log ε) = 210 (4.11).

MS (EI): *m/z* (%) = 332/334 (2/2) [M^+], 287/289 (12/13), 244/246 (6/5), 235/237 (9/9), 155 (5), 141 (5), 137 (6), 135 (5), 128 (12), 127 (76), 123 (12), 121 (6), 115 (5), 114 (21), 113 (5), 111 (5), 110 (6), 109 (24), 107 (12), 101 (23), 100 (7), 99 (84), 98 (8), 97 (22), 7 (17), 6 (36), 5 (7), 4 (8), 89 (7), 88 (30), 86 (24), 9 (6), 84 (12), 83 (19), 82 (18), 81 (58), 80 (6), 79 (15), 77 (5), 73 (21), 71 (11), 70 (11), 69 (36), 68 (32), 67 (42), 65 (5), 57 (19), 56 (12), 55 (100), 54 (17), 53 (27), 45 (7), 43 (49), 42 (16), 41 (83), 40 (8), 39 (26).

HRMS (CI) *m/z*: $[M + H]^+$ Calcd for $C_{16}H_{30}BrO_2$ 333.1429; Found 333.1423

(*Z*)-14-Bromotetradec-2-en-1-ol

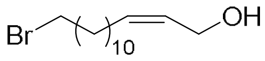

This allyl alcohol was prepared following general procedure 4:
Ester **33** (729 mg, 2.19 mmol, 1.0 eq.) in dry CH_2Cl_2 (30 mL).
DIBAL-*H* solution (6.56 mL, 6.56 mmol, 3.0 eq., 1.0 M in CH_2Cl_2).
Flash chromatography [silica gel, CH_2Cl_2].
(*Z*)-14-Bromotetradec-2-en-1-ol (550 mg, 1.89 mmol, 86%) as colorless oil.

TLC [silica gel, CH_2Cl_2]: R_f = 0.31.

^{1}H-NMR (400 MHz, $CDCl_3$): δ = 5.64-5.50 (m, 2H, C*H*C*H*CH_2OH), 4.22-4.17 (m, 2H, CH_2OH), 3.41 (t, ^3J = 6.9 Hz, 2H, CH_2Br), 2.11-2.02 (m, 2H, CH_2CHCHCH_2OH), 1.90-1.81 (m, 2H, CH_2CH_2Br), 1.47-1.24 (m, 16H, (CH_2)$_8$).

^{13}C-NMR (100 MHz, $CDCl_3$): δ = 133.3 (*C*HCHCH_2OH), 128.3 (*C*HCH_2OH), 58.6 (*C*H_2OH), 34.0 (*C*H_2Br), 32.8 (*C*H_2CH_2Br), 29.6 (*C*H_2), 29.5 (2C, *C*H_2), 29.4 (2C, *C*H_2), 29.2 (*C*H_2), 28.7 (*C*H_2), 28.2 (*C*H_2), 27.4 (*C*H_2CHCHCH_2OH).

IR (ATR): $\tilde{\nu}$ = 3329 (br), 3013 (w), 233 (s), 292 (m), 1463 (w), 1252 (w), 1009 (m), 721 (m), 645 (w), 563 (w).

UV/VIS (MeOH): λ_{max} (log ε) = 202 (3.20).

MS (EI): *m/z* (%) = 290/23 (missing) [M^+], 272/274 (3/3), 123 (6), 111 (5), 110 (5), 109 (22), 107 (8), 97 (13), 7 (24), 6 (33), 4 (6), 91 (5), 83 (25), 82 (40), 81 (46), 80 (9), 79 (26), 77 (10), 71 (7), 70 (11), 69 (33), 68 (39), 67 (66), 66 (9), 65 (8), 57 (54), 56 (12), 55 (80), 54 (48), 53 (20), 43 (31), 42 (17), 41 (100), 40 (6), 39 (32).

HRMS (CI) *m/z*: $[M-18 - H]^+$ Calcd for $C_{14}H_{24}Br$ 271.1061; Found 271.1057

((1*RS*,2*SR*)-2-(11-Bromoundecyl)cyclopropyl)methanol (34)

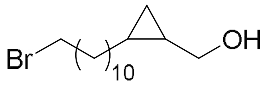

34

Alcohol **34** was prepared following general procedure 5:
(*Z*)-14-Bromotetradec-2-en-1-ol (527 mg, 1.81 mmol, 1.0 eq.) in dry CH_2Cl_2 (20 mL).

Diethylzinc solution (9.05 mL, 9.05 mmol, 5.0 eq., 1.0 M in hexane).
Methylene iodide (0.73 mL, 9.05 mmol, 5.0 eq.).
Flash chromatography [silica gel, CH_2Cl_2].
Alcohol **34** (503 mg, 1.65 mmol, 91%) as colorless solid.

TLC [silica gel, CH_2Cl_2]: R_f = 0.38.

^{1}H-NMR (400 MHz, $CDCl_3$): δ = 3.68-3.55 (m, 2H, C*H*$_2$OH), 3.41 (t, ^3J = 6.9 Hz, 2H, C*H*$_2$Br), 1.90-1.81 (m, 2H, C*H*$_2$CH$_2$Br), 1.50-1.19 (m, 18H, (C*H*$_2$)$_9$), 1.15-1.05 (m, 1H, C*H*CH$_2$OH), 0.3-0.81 (m, 1H, C*H*CHCH$_2$OH), 0.70 (dt, ^3J = 4.6 Hz, 8.3 Hz, 1H, CHC[*H*]$_2$CH), −(0.01-0.06) (m, 1H, CHC[*H*]$_2$CH).

^{13}C-NMR (100 MHz, $CDCl_3$): δ = 63.4 (*C*H$_2$OH), 34.0 (*C*H$_2$Br), 32.8 (*C*H$_2$CH$_2$Br), 30.2 (*C*H$_2$CHCHCH$_2$OH), 29.6 (2C, *C*H$_2$), 29.5 (2C, *C*H$_2$), 29.4 (*C*H$_2$), 28.8 (*C*H$_2$), 28.6 (*C*H$_2$), 28.2 (*C*H$_2$), 18.2 (*C*HCH$_2$OH), 16.2 (*C*HCHCH$_2$OH), 9.5 (CH*C*H$_2$CH).

IR (ATR): $\tilde{\nu}$ = 3339 (br), 3063 (w), 26 (w), 232 (s), 292 (m), 1463 (w), 1251 (w), 1029 (m), 823 (w), 721 (w), 645 (w), 563 (w).

MS (EI): *m/z* (%) = 304/306 (missing) [M$^+$], 189/191 (5/4), 123 (9), 109 (24), 107 (11), 97 (17), 7 (12), 6 (22), 4 (8), 91 (11), 83 (21), 82 (19), 81 (45), 80 (10), 79 (41), 77 (16), 70 (5), 69 (27), 68 (26), 67 (55), 66 (8), 65 (12), 57 (18), 56 (11), 55 (76), 54 (24), 53 (23), 51 (5), 44 (5), 43 (30), 42 (17), 41 (100), 40 (6), 39 (35).

HRMS (CI) *m/z*: [M - H]$^+$ Calcd for $C_{15}H_{28}BrO$ 303.1324; Found 331.1317

(11-((1*RS*,2*SR*)-2-(Hydroxymethyl)cyclopropyl)undecyl)triphenylphosphonium iodide (35)

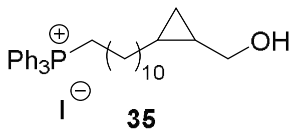

35

Phosphonium salt **35**was prepared following general procedure 5:
34 (484 mg, 1.59 mmol, 1.0 eq.) in CH_3CN (25 mL).
Triphenylphosphane (832 mg, 3.17 mmol, 2.0 eq.).
Sodium iodide (1.19 g, 7.5 mmol, 5.0 eq.).
Flash chromatography [silica gel, CH_2Cl_2, then CH_2Cl_2/MeOH (20:1)].
35 (40 mg, 1.51 mmol, 6%) as colorless oil.

TLC [silica gel, CH_2Cl_2/MeOH (20:1)]: R_f = 0.25.

^{1}H-NMR (400 MHz, $CDCl_3$): δ = 7.9-7.77 (m, 9H, *m*-/*p*-Ph-C*H*), 7.76-7.70 (m, 6H, *o*-Ph-C*H*), 3.67-3.53 (m, 4H, PC*H*$_2$, C*H*$_2$OH), 1.88 (s, br, 1H, O*H*), 1.71-1.57 (m, 4H, PCH$_2$C*H*$_2$C*H*$_2$), 1.47-1.16 (m, 16H, (C*H*$_2$)$_8$), 1.15-1.03 (m, 1H, C*H*CH$_2$OH), 0.90-0.78 (m, 1H, C*H*CHCH$_2$OH), 0.72-0.64 (m, 1H, CHC[*H*]$_2$CH), −(0.02-0.08) (m, 1H, CHC[*H*]$_2$CH).

^{13}C-NMR (100 MHz, $CDCl_3$): δ = 135.0 (3C, d, J = 2.9 Hz, *p*-Ph-*C*H), 133.5 (6C, d, J = 10.0 Hz, *o*-Ph-*C*H), 130.5 (6C, d, J = 12.5 Hz, *m*-Ph-*C*H), 118.0 (3C, d, J = 9.9 Hz, *ipso*-Ph-*C*), 63.0 (*C*H$_2$OH), 30.3 (d, J = 15.5 Hz, PCH$_2$CH$_2$*C*H$_2$), 30.0 (*C*H$_2$CHCHCH$_2$OH), 29.4 (*C*H$_2$), 29.3 (3C, *C*H$_2$), 29.0 (2C, *C*H$_2$), 28.4 (*C*H$_2$), 23.0 (d, J = 49.9 Hz, P*C*H$_2$), 22.5 (d, J = 4.7 Hz, PCH$_2$*C*H$_2$), 18.0 (*C*HCH$_2$OH), 16.0 (*C*HCHCH$_2$OH), 9.4 (CH*C*H$_2$CH).

IR (ATR): $\tilde{\nu}$ = 3381 (br), 3057 (w), 2991 (w), 233 (m), 292 (m), 2189 (w), 1587 (w), 1484 (w), 1463 (w), 1437 (m), 1237 (w), 1112 (m), 1029 (m), 97 (w), 917 (w), 745 (s), 722 (s), 688 (s), 661 (m), 641 (w), 532 (m).

UV/VIS (MeOH): λ_{max} (log ε) = 274 (3.33), 267 (3.41), 222 (4.49), 203 (4.64).

MS (ESI+): *m/z* (%) = 487.3 (100) $[M-I]^+$.

HRMS (ESI) *m/z*: $[M - I]^+$ Calcd for $C_{33}H_{44}OP$ 487.3124; Found 487.3117

((1*RS*,2*SR*)-2-((*E*)-12-(1-Trityl-1*H*-imidazol-4-yl)dodec-11-en-1-yl)cyclopropyl)-methanol (36)

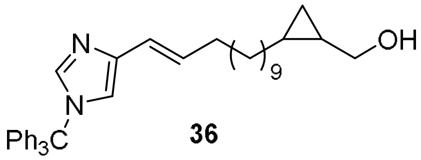

36

Alcohol **36** was prepared following general procedure 7:
Phosphonium salt **35** (870 mg, 1.42 mmol, 1.0 eq.) in dry THF (20 mL).
Phenyllithium solution (1.71 mL, 3.08 mmol, 2.2 eq., 1.8 M in Bu_2O).
Aldehyde **8** (520 mg, 1.54 mmol, 1.1 eq.) in dry THF (15 mL).
Phenyllithium solution (1.71 mL, 3.08 mmol, 2.2 eq., 1.8 M in Bu_2O).
Flash chromatography [silica gel, *n*-pentane/EtOAc (3:1) to (1:1)].
35 (47 mg, 0.91 mmol, 64%) as yellowish solid.

TLC [silica gel, *n*-pentane /EtOAc (2:1)]: R_f = 0.30.

^{1}H-NMR (400 MHz, $CDCl_3$): δ = 7.39 (d, ^4J = 1.0 Hz, 1H, NC*H*N), 7.35-7.30 (m, 9H, *m*-/*p*-Ph-C*H*), 7.17-7.12 (m, 6H, *o*-Ph-C*H*), 6.66 (d, ^4J = 1.2 Hz, 1H, NCC*H*N), 6.36 (td, ^3J = 6.8 Hz, 15.6 Hz, 1H, NCCHC*H*), 6.20 (d, ^3J = 15.7 Hz, 1H, NCC*H*CH), 3.67-3.55 (m, 2H, CH_2OH), 2.18-2.11 (m, 2H, NCCHCHCH_2), 1.48-1.18 (m, 18H, $(CH_2)_9$), 1.14-1.03 (m, 1H, C*H*CH_2OH), 0.91-0.79 (m, 1H, C*H*CHCH_2OH), 0.69 (dt, *J* = 4.6 Hz, 8.3 Hz, 1H, CHC[*H*]$_2$CH), −0.04 (dd, *J* = 5.4 Hz, 10.2 Hz, 1H, CHC[*H*]$_2$CH).

^{13}C-NMR (100 MHz, $CDCl_3$): δ = 142.3 (3C, *ipso*-Ph-*C*), 139.4 (N*C*CHN), 138.8 (N*C*HN), 130.0 (NCCH*C*H), 129.8 (6C, *o*-Ph-*C*H), 128.0 (9C, *m*-/*p*-Ph-*C*H), 121.2 (NC*C*HCH), 118.0 (NC*C*HN), 75.3 (*C*Ph_3), 63.2 (*C*H_2OH), 32.8 (NCCHCH*C*H_2), 30.1 (*C*H_2), 29.5 (5C, *C*H_2), 29.3 (*C*H_2), 29.2 (*C*H_2), 28.5 (*C*H_2), 18.2 (*C*HCH_2OH), 16.1 (*C*HCHCH_2OH), 9.5 (CH*C*H_2CH).

IR (ATR): $\tilde{\nu}$ = 3261 (br), 3058 (w), 2987 (w), 230 (m), 290 (m), 156 (w), 1490 (w), 1470 (w), 1445 (m), 1291 (w), 1228 (w), 1184 (w), 1157 (w), 1131 (w), 109 (w), 1033 (m), 79 (m), 905 (w), 869 (w), 830 (w), 749 (s), 700 (s), 656 (m), 638 (m).

UV/VIS (MeOH): λ_{max} (log ε) = 241 (4.27), 204 (4.71).

MS (ESI+): *m/z* (%) = 1115.7 (82) $[2M+Na]^+$, 569.4 (64) $[M+Na]^+$, 547.4 (61) $[M+H]^+$, 243.1 (100) $[CPh_3]^+$.

HRMS (ESI) *m/z*: $[M + Na]^+$ Calcd for $C_{38}H_{46}N_2NaO$ 569.3502; Found 569.3501

(1*RS*,2*SR*)-2-((*E*)-12-(1-Trityl-1*H*-imidazol-4-yl)dodec-11-en-1-yl)cyclopropane-1-carbaldehyde

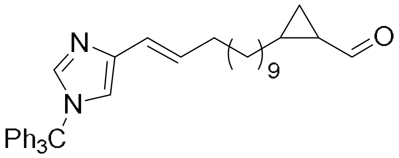

This aldehyde was prepared following general procedure 2:
Alcohol **35** (468 mg, 0.86 mmol, 1.0 eq.) in dry CH_2Cl_2 (20 mL).
Triethylamine (1.19 mL, 8.65 mmol, 10 eq.).
Py·SO_3 (409 mg, 2.57 mmol, 3.0 eq.) in dry DMSO (10 mL).
Flash chromatography [silica gel, *n*-pentane/EtOAc (8:1) to (3:1)].
The aldehyde was obtained (300 mg, 0.55 mmol, 64%) as colorless oil.

TLC [silica gel, *n*-pentane/EtOAc (5:1)]: R_f = 0.24.

^{1}H-NMR (400 MHz, $CDCl_3$): δ = 9.34 (d, ^3J = 5.6 Hz, 1H, C*H*O), 7.38 (d, ^4J = 1.2 Hz, 1H, NC*H*N), 7.35-7.30 (m, 9H, *m*-/*p*-Ph-C*H*), 7.17-7.12 (m, 6H, *o*-Ph-C*H*), 6.67 (d, ^4J = 1.3 Hz, 1H, NCC*H*N), 6.35 (td, ^3J = 6.8 Hz, 15.6 Hz, 1H, NCCHC*H*), 6.21 (d, ^3J = 15.7 Hz, 1H, NCC*H*CH), 2.18-2.11 (m, 2H, NCCHCHCH_2), 1.9 (tt, J = 5.4 Hz, 8.1 Hz, C*H*CHO), 1.65-1.15 (m, 21H, $(CH_2)_9$, C*H*CH_2CHCHO).

^{13}C-NMR (100 MHz, $CDCl_3$): δ = 201.8 (*C*HO), 142.3 (3C, *ipso*-Ph-*C*), 139.4 (N*C*CHN), 138.9 (N*C*HN), 129.9 (NCCH*C*H), 129.8 (6C, *o*-Ph-*C*H), 128.0 (9C, *m*-/*p*-Ph-*C*H), 121.2 (NC*C*HCH), 118.0 (NC*C*HN), 75.2 (*C*Ph_3), 32.8 (NCCHCH*C*H_2), 29.9 (*C*H_2), 29.5 (4C, *C*H_2), 29.3 (*C*H_2), 29.2 (2C, *C*H_2), 28.2 (*C*H_2), 27.8 (*C*HCHO), 24.7 (*C*HCHCHO), 14.7 (CH*C*H_2CH).

IR (ATR): $\tilde{\nu}$ = 3032 (w), 230 (m), 290 (m), 2730 (w), 1700 (m), 157 (w), 1490 (m), 1468 (w), 1444 (m), 1360 (w), 1288 (w), 1227 (w), 1182 (w), 1157 (w), 1128 (w), 109 (w), 1035 (w), 972 (m), 907 (w), 868 (w), 827 (w), 749 (s), 700 (s), 655 (m), 638 (m).

UV/VIS (CH_2Cl_2): λ_{max} (log ε) = 247 (4.23), 228 (4.27).

MS (ESI+): *m/z* (%) = 1111.7 (76) $[2M+Na]^+$, 567.3 (67) $[M+Na]^+$, 545.4 (97) $[M+H]^+$, 243.1 (100) $[CPh_3]^+$.

HRMS (ESI) *m/z*: $[M + H]^+$ Calcd for $C_{38}H_{45}N_2O$ 545.3526; Found 545.3524

(1*RS*,2*SR*)-2-((*E*)-12-(1-Trityl-1*H*-imidazol-4-yl)dodec-11-en-1-yl)cyclopropane-1-carboxylic acid (37)

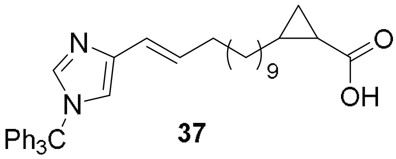

37

Acid **36** was prepared following general procedure 8:
The corresponding aldehyde (266 mg, 0.49 mmol, 1.0 eq.) in *tert*-BuOH (18 mL)/THF (15 mL) and 2-methyl-2-butene (3 mL).
$NaClO_2$ (309 mg, 3.42 mmol, 7.0 eq.), NaH_2PO_4 (606 mg, 4.39 mmol, 9.0 eq.) in water (5 mL).
Flash chromatography [silica gel, $CHCl_3$/MeOH/NH_3 (90:10:1)].
37 (226 mg, 0.40 mmol, 83%) as colorless oil.

TLC [silica gel, $CHCl_3$/MeOH/NH_3 (90:10:1)]: R_f = 0.19.

^{1}H-NMR (400 MHz, $CDCl_3$): δ = 7.49 (d, ^4J = 1.3 Hz, 1H, NC*H*N), 7.36-7.30 (m, 9H, *m*-/*p*-Ph-C*H*), 7.16-7.10 (m, 6H, *o*-Ph-C*H*), 6.65 (d, ^4J = 1.3 Hz, 1H, NCC*H*N), 6.30 (td, ^3J = 6.7 Hz, 15.8 Hz, 1H, NCCHC*H*), 6.20 (d, ^3J = 15.9 Hz, 1H, NCC*H*CH), 2.18-2.10 (m, 2H, NCCHCHCH_2), 1.69-1.17 (m, 20H, $(CH_2)_9$, C*H*C*H*CO_2H), 1.02-0.89 (m, 2H, CHCH_2CH).

^{13}C-NMR (100 MHz, $CDCl_3$): δ = 177.0 (CO_2H), 142.1 (3C, *ipso*-Ph-*C*), 139.0 (N*C*CHN), 138.7 (N*C*HN), 130.7 (NCCH*C*H), 129.8 (6C, *o*-Ph-*C*H), 128.1 (9C, *m*-/*p*-Ph-*C*H), 120.8 (NC*C*HCH), 117.9 (NC*C*HN), 75.5 (*C*Ph_3), 32.7 (NCCHCH*C*H_2), 29.5 (*C*H_2), 29.2 (*C*H_2), 29.0 (4C, *C*H_2), 28.9 (*C*H_2), 28.6 (*C*H_2), 27.1 (*C*H_2), 22.3 (*C*HCHCO_2H), 18.3 (*C*HCO_2H), 13.6 (CH*C*H_2CH).

IR (ATR): $\tilde{\nu}$ = 3060 (w), 233 (m), 292 (w), 164 (m), 143 (w), 1446 (w), 119 (m), 1157 (m), 1129 (w), 1087 (w), 1037 (w), 1001 (w), 77 (w), 908 (w), 869 (w), 746 (m), 699 (s), 659 (m), 638 (m).

UV/VIS (MeOH): λ_{max} (log ε) = 299 (3.03), 250 (3.87), 205 (4.63).

MS (ESI+): *m/z* (%) = 561.3 (100) $[M+H]^+$.

HRMS (ESI) *m/z*: $[M + H]^+$ Calcd for $C_{38}H_{45}N_2O_2$ 561.3476; Found 561.3480

(1*RS*,2*SR*)-2-((*E*)-12-(1*H*-Imidazol-4-yl)dodec-11-en-1-yl)cyclopropane-1-carboxylic acid (38)

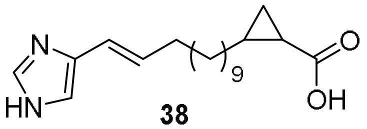

38

Acid **38** was prepared following general procedure 9:
Acid **37** (199 mg, 0.35 mmol, 1.0 eq.).
Acetic acid (2.00 mL) in methanol (20 mL).
Flash chromatography [silica gel, $CHCl_3$/MeOH/NH_3 (40:10:1)].
38 (77 mg, 0.24 mmol, 68%) as colorless solid.

TLC [silica gel, $CHCl_3$/MeOH/NH_3 (40:10:1)]: R_f = 0.24.

^{1}H-NMR (600 MHz, CD_3OD): δ = 7.60 (s, 1H, NC*H*N), 6.3 (s, 1H, NCC*H*N), 6.27 (d, ^3J = 15.9 Hz, 1H, NCC*H*CH), 6.14 (td, ^3J = 6.9 Hz, 15.9 Hz, 1H, NCCHC*H*), 2.20-2.14 (m, 2H, NCCHCHCH_2), 1.65-1.59 (m, 1H, C*H*CO_2H), 1.57-1.22 (m, 18H, $(CH_2)_9$,), 1.21-1.13 (m, 1H, C*H*CHCO_2H), 0.3-0.88 (m, 1H, CHC[*H*]$_2$CH), 0.80-0.76 (m, 1H, CHC[*H*]$_2$CH).

^{13}C-NMR (150 MHz, CD_3OD): δ = 178.7 (*C*O_2H), 136.4 (N*C*HN), 136.2 (N*C*CHN), 130.6 (NCCH*C*H), 120.5 (NC*C*HCH), 119.6 (NC*C*HN), 34.0 (NCCHCH*C*H_2), 30.8 (4C, *C*H_2), 30.6 (3C, *C*H_2), 30.3 (*C*H_2), 28.6 (*C*H_2), 22.1 (*C*HCHCO_2H), 20.3 (*C*HCO_2H), 13.4 (CH*C*H_2CH).

IR (ATR): $\tilde{\nu}$ = 3149 (w), 3002 (w), 2919 (s), 2849 (m), 256 (br), 243 (br), 138 (br), 1698 (w), 1675 (w), 1552 (m), 1467 (m), 1413 (m), 1289 (m), 1211 (w), 1166 (w), 1098 (m), 1043 (w), 1020 (w), 72 (m), 31 (w), 878 (m), 811 (m), 771 (w), 717 (m), 626 (m).

UV/VIS (MeOH): λ_{max} (log ε) = 250 (4.09), 201 (3.82).

MS (ESI+): *m/z* (%) = 637.5 (100) $[2M+H]^+$, 319.2 (40) $[M+H]^+$.

HRMS (ESI) *m/z*: $[M + H]^+$ Calcd for $C_{19}H_{31}N_2O_2$ 319.2380; Found 319.2381

5.6 Enantioselective syntheses of imidacin A1

2-Butyl-1,3,6,2-dioxazaborocane

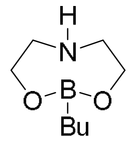

A solution of butylboronic acid (5.05 g, 49.5 mmol, 1.0 eq.) in dry Et_2O (70 mL) and dry CH_2Cl_2 (60 mL) with molecular sieve 3 Å was treated at r.t. with diethanolamine (4.75 mL, 49.5 mmol, 1.0 eq.) by stirring at r.t. for 3.5 h. The reaction mixture was filtered and the solvent was removed under reduced pressure. The residue was taken up in CH_2Cl_2 (10 mL) and heated to reflux. Then *tert*.-butyl methyl ether (TBME, 50 mL) was added and the mixture was slowly allowed to cool down to r.t. and later on cooled in fridge to 5°C. The product was filtered off, washed with Et_2O. After drying under reduced pressure, the borane (8.08 g, 47.2 mmol, 6%) was obtained as colorless solid. The spectroscopic data matched those reported.[S19]

^{1}H-NMR (300 MHz, $CDCl_3$): δ = 6.31 (s, br, 1H, N*H*), 3.99-3.76 (m, 4H, OCH_2), 3.35-3.19 (m, 2H, NCH_2), 2.79-2.66 (m, 2H, NCH_2), 1.39-1.17 (m, 4H, $BCH_2CH_2CH_2$), 0.87 (t, ^{3}J = 7.0 Hz, 3H, CH_3), 0.48-0.37 (m, 2H, BCH_2).

^{13}C-NMR (75 MHz, $CDCl_3$): δ = 62.5 (2C, OCH_2), 51.5 (2C, NCH_2), 28.1 (BCH_2CH_2), 26.5 ($BCH_2CH_2CH_2$), 18.4 (BCH_2), 14.2 (CH_3).

IR (ATR): $\tilde{\nu}$ = 3186 (w), 3118 (w), 266 (w), 2914 (m), 2863 (m), 2711 (w), 1461 (w), 1421 (w), 1368 (w), 1348 (w), 1310 (w), 1271 (w), 1232 (m), 1186 (w), 1137 (w), 1101 (m), 1050 (s), 998 (m), 63 (m), 908 (m), 874 (m), 827 (w), 784 (m), 744 (m), 64 (w), 657 (w), 610 (w).

UV/VIS (CH_2Cl_2): λ_{max} (log ε) = 227 (1.48).

(4*R*,5*R*)-2-Butyl-*N*⁴,*N*⁴,*N*⁵,*N*⁵-tetramethyl-1,3,2-dioxaborolane-4,5-dicarboxamide ((*R*,*R*)-10)

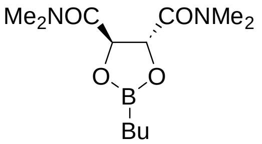

(*R*,*R*)-**10**

A solution of 2-butyl-1,3,6,2-dioxazaborocane (1.26 g, 7.37 mmol, 1.0 eq.) and tetramethyl-L-tartaric acid diamide (1.7 g, 9.60 mmol, 1.3 eq.) in CH_2Cl_2 (40 mL) and sat. NaCl solution (12 mL) was stirred at r.t. for 1 h. The layers were separated and the aqueous layer was extracted with CH_2Cl_2 (3 x 20 mL). The combined organic layers were washed with sat. NaCl solution (50 mL) and dried with $MgSO_4$. The solvent was removed under reduced pressure and (*R*,*R*)-**10** (1.47 g, 5.44 mmol, 74%) was obtained as colorless oil. The spectroscopic data matched those reported.[S19]

^{1}H-NMR (400 MHz, $CDCl_3$): δ = 5.53 (s, 2H, C*H*CON), 3.20 (s, 6H, NCH_3), 2.99 (s, 6H, NCH_3), 1.45-1.25 (m, 4H, $BCH_2CH_2CH_2$), 0.3-0.83 (m, 5H, BC$H_2(CH_2)_2CH_3$).

^{13}C-NMR (100 MHz, $CDCl_3$): δ = 168.4 (2C, *C*ON), 75.7 (2C, *C*HCON), 37.0 (2C, NCH_3), 35.9 (2C, NCH_3), 25.8 (CH_2), 25.1 (CH_2), 13.7 ($B(CH_2)_3CH_3$), 9.9 (br, BCH_2).

IR (ATR): $\tilde{\nu}$ = 3397 (br), 264 (w), 238 (w), 2871 (w), 1638 (s), 1504 (w), 1466 (w), 1377 (m), 1255 (w), 1155 (w), 1060 (m), 1026 (m), 972 (w), 9 (w), 825 (w), 757 (w), 722 (w), 676 (w), 638 (w), 575 (w).

UV/VIS (CH_2Cl_2): λ_{max} (log ε) = 228 (3.21).

MS (EI): *m/z* (%) = 270 (<5) [M^+], 213 (13), 199 (17), 198 (100) $[M-CONMe_2]^+$, 197 (35), 169 (11), 168 (7), 153 (17), 152 (5), 142 (6), 114 (7), 98 (5), 72 (68).

(4*S*,5*S*)-2-Butyl-*N*^{4},*N*^{4},*N*^{5},*N*^{5}-tetramethyl-1,3,2-dioxaborolane-4,5-dicarboxamide ((*S*,*S*)-10)

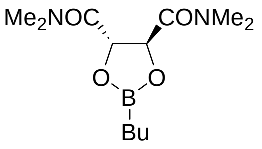

(*S*,*S*)-**10**

A solution of 2-butyl-1,3,6,2-dioxazaborocane (1.26 g, 7.37 mmol, 1.0 eq.) and tetramethyl-D-tartaric acid diamide (1.7 g, 9.60 mmol, 1.3 eq.) in CH_2Cl_2 (40 mL) and sat. NaCl solution (12 mL) was stirred at r.t. for 3 h. The layers were separated and the aqueous layer was extracted with CH_2Cl_2 (3 x 20 mL). The combined organic layers were washed with sat. NaCl solution (50 mL) and dried with $MgSO_4$. The solvent was removed under reduced pressure and (*S*,*S*)-**10** (1.65 g, 6.11 mmol, 83%) was obtained as colorless oil. The spectroscopic data matched those reported.[S19]

^{1}H-NMR (400 MHz, $CDCl_3$): δ = 5.53 (s, 2H, C*H*CON), 3.20 (s, 6H, NCH_3), 2.99 (s, 6H, NCH_3), 1.45-1.27 (m, 4H, $BCH_2CH_2CH_2$), 0.3-0.82 (m, 5H, BCH_2(CH_2)$_2$CH_3).

^{13}C-NMR (100 MHz, $CDCl_3$): δ = 168.4 (2C, *C*ON), 75.7 (2C, *C*HCON), 37.1 (2C, NCH_3), 36.0 (2C, NCH_3), 25.8 (CH_2), 25.2 (CH_2), 13.8 (B(CH_2)$_3$$CH_3$), 10.0 (br, B$CH_2$).

IR (ATR): $\tilde{\nu}$ = 3410 (br), 264 (w), 240 (w), 2871 (w), 1641 (s), 1503 (w), 1463 (w), 1378 (m), 1254 (w), 1154 (m), 1058 (m), 79 (w), 84 (w), 814 (w), 721 (w), 678 (w), 638 (w), 561 (w).

UV/VIS (CH_2Cl_2): λ_{max} (log ε) = 229 (3.25).

MS (EI): *m/z* (%) = 270 (<5) [M^+], 213 (14), 199 (19), 198 (100) [M−$CONMe_2$]$^+$, 197 (38), 169 (13), 168 (8), 153 (20), 152 (5), 142 (7), 140 (5), 114 (8), 98 (5), 73 (5), 72 (69), 70 (5), 42 (5).

((1*S*,2*S*)-2-(11-Bromundecyl)cyclopropyl)methanol ((*S*,*S*)-4)

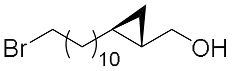

(*S*,*S*)-**4**

Allyl alcohol **3** (1.04 g, 3.57 mmol, 1.0 eq.) was added to a solution of (*R*,*R*)-**10** (1.06 g, 3.3 mmol, 1.1 eq.) in dry CH_2Cl_2 (50 mL) and the mixture cooled down to −20 °C. Then diethylzinc solution (17.9 mL, 17.9 mmol, 5.0 eq., 1.0 M in *n*-hexane) was slowly added followed by methylene iodide (1.44 mL, 17.8 mmol, 5.0 eq.). The reaction mixture was allowed to warm to r.t. over 4 h and sat. NH_4Cl solution (50 mL) was added. The layers were separated and the aqueous layer was extracted with CH_2Cl_2 (3 x 30 mL). The combined organic layers were washed with sat. $NaHCO_3$ solution (50 mL), sat. NaCl solution (50 mL), water (50 mL) and dried with $MgSO_4$. The solvent was removed under reduced pressure and the residue was purified by flash chromatography [silica gel, CH_2Cl_2]. (*S*,*S*)-**4** (1.07 g, 3.50 mmol, 98%, 82% *ee*) was obtained as colorless solid.[S12,S19]

TLC [silica gel, CH_2Cl_2]: R_f = 0.31.

^{1}H-NMR (400 MHz, $CDCl_3$): δ = 3.49-3.38 (m, 4H, CH_2OH, CH_2Br), 1.90-1.81 (m, 2H, C$H_2$$CH_2$Br), 1.48-1.15 (m, 18H, (CH_2)$_9$), 0.88-0.78 (m, 1H, C*H*CHCH_2OH), 0.64-0.54 (m, 1H, C*H*CH_2OH), 0.39-0.27 (m, 2H, CHCH_2CH).

^{13}C-NMR (100 MHz, $CDCl_3$): δ = 67.2 (CH_2OH), 34.0 (CH_2Br), 33.6 (CH_2CHCHCH_2OH), 32.8 ($CH_2$$CH_2$Br), 29.6 (3C, CH_2), 29.5 (CH_2), 29.4 (2C, CH_2), 28.8 (CH_2), 28.2 (CH_2), 21.2 (*C*HCHCH_2OH), 17.2 (*C*HCH_2OH), 9.9 (CHCH_2CH).

$[\alpha]_D^{20.7}$ = +11.1 (*c* = 2.43, $CDCl_3$).

((1*R*,2*R*)-2-(11-Bromundecyl)cyclopropyl)methanol ((*R*,*R*)-4)

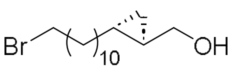

(*R*,*R*)-**4**

Diethylzinc solution (22.8 mL, 22.8 mmol, 5.0 eq., 1.0 M in *n*-hexane) followed by methylene iodide (1.84 mL, 22.8 mmol, 5.0 eq.) were slowly added to a solution of allyl alcohol **3** (1.33 g, 4.57 mmol, 1.0 eq.) and (*S*,*S*)-**10** (1.42 g, 5.26 mmol, 1.2 eq.) in dry CH_2Cl_2 (80 mL) at −20 °C. The reaction mixture was allowed to warm to r.t. over 3 h until sat. NH_4Cl solution (60 mL) was added. The layers were separated and the aqueous layer was extracted with CH_2Cl_2 (3 x 30 mL). The combined organic layers were washed with sat. $NaHCO_3$ solution (50 mL), sat. NaCl solution (50 mL), water (50 mL) and dried with $MgSO_4$. The solvent was removed under reduced pressure and the residue was purified by flash chromatography [silica gel, CH_2Cl_2]. (*R*,*R*)-**4** (1.30 g, 4.26 mmol, 4%, 71% *ee*) was obtained as colorless solid.[S12,S19]

TLC [silica gel, CH_2Cl_2]: R_f = 0.27.

^{1}H-NMR (400 MHz, $CDCl_3$): δ = 3.48-3.38 (m, 4H, C*H*$_2$OH, C*H*$_2$Br), 1.90-1.81 (m, 2H, C*H*$_2$CH$_2$Br), 1.47-1.16 (m, 18H, (C*H*$_2$)$_9$), 0.88-0.78 (m, 1H, C*H*CHCH$_2$OH), 0.64-0.54 (m, 1H, C*H*CH$_2$OH), 0.40-0.27 (m, 2H, CHC*H*$_2$CH).

^{13}C-NMR (100 MHz, $CDCl_3$): δ = 67.2 (*C*H$_2$OH), 34.0 (*C*H$_2$Br), 33.6 (*C*H$_2$CHCHCH$_2$OH), 32.8 (*C*H$_2$CH$_2$Br), 29.6 (3C, *C*H$_2$), 29.5 (*C*H$_2$), 29.4 (2C, *C*H$_2$), 28.7 (*C*H$_2$), 28.2 (*C*H$_2$), 21.2 (*C*HCHCH$_2$OH), 17.2 (*C*HCH$_2$OH), 9.9 (CH*C*H$_2$CH).

$[\alpha]_D^{20.6}$ = −11.2 (*c* = 3.75, $CDCl_3$).

(11-((1*S*,2*S*)-2-(Hydroxymethyl)cyclopropyl)undecyl)triphenylphosphonium iodide ((*S*,*S*)-5)

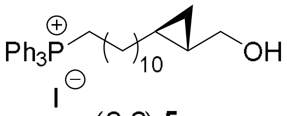

(*S*,*S*)-**5**

Phosphonium salt (*S*,*S*)-**5** was prepared following general procedure 6:
(*S*,*S*)-**4** (1.02 g, 3.34 mmol, 1.0 eq.) in CH_3CN (50 mL).
Triphenylphosphane (1.75 g, 6.67 mmol, 2.0 eq.).
Sodium iodide (2.50 g, 16.7 mmol, 5.0 eq.).
Flash chromatography [silica gel, CH_2Cl_2, then CH_2Cl_2/MeOH (20:1) to (10:1)].
(*S*,*S*)-**5** (2.05 g, 3.34 mmol, 99%) as yellowish oil.

TLC [silica gel, CH_2Cl_2/MeOH (20:1)]: R_f = 0.24.

^{1}H-NMR (400 MHz, $CDCl_3$): δ = 7.86-7.78 (m, 9H, *m*-/*p*-Ph-C*H*), 7.76-7.68 (m, 6H, *o*-Ph-C*H*), 3.70-3.57 (m, 2H, PC*H*$_2$), 3.49-3.37 (m, 2H, C*H*$_2$OH), 1.78 (s, br, 1H, O*H*), 1.70-1.58 (m, 4H, PCH$_2$C*H*$_2$C*H*$_2$), 1.40-1.13 (m, 16H, (C*H*$_2$)$_8$), 0.87-0.77 (m, 1H, C*H*CHCH$_2$OH), 0.63-0.53 (m, 1H, C*H*CH$_2$OH), 0.39-0.25 (m, 2H, CHC*H*$_2$CH).

^{13}C-NMR (100 MHz, $CDCl_3$): δ = 135.1 (3C, d, *J* = 3.0 Hz *p*-Ph-*C*H), 133.6 (6C, d, *J* = 10.0 Hz, *o*-Ph-*C*H), 130.5 (6C, d, *J* = 12.5 Hz, *m*-Ph-*C*H), 118.1 (3C, d, *J* = 86.0 Hz, *ipso*-Ph-*C*), 67.0 (*C*H$_2$OH), 33.5 (*C*H$_2$CHCHCH$_2$OH), 30.4 (d, *J* = 15.6 Hz, PCH$_2$CH$_2$*C*H$_2$), 29.5 (2C, *C*H$_2$), 29.4 (2C, *C*H$_2$), 29.2 (*C*H$_2$), 29.1 (2C, *C*H$_2$), 23.1 (d, *J* = 49.9 Hz, P*C*H$_2$), 22.6 (d, *J* = 4.7 Hz, PCH$_2$*C*H$_2$), 21.1 (*C*HCHCH$_2$OH), 17.1 (*C*HCH$_2$OH), 9.9 (CH*C*H$_2$CH).

HRMS (ESI) *m/z*: $[M- I]^+$ Calcd for $C_{33}H_{44}OP$ 487.3124; Found 487.3133

$[\alpha]_D^{21.8}$ = +5.27 (*c* = 2.37, $CDCl_3$).

(11-((1*R*,2*R*)-2-(Hydroxymethyl)cyclopropyl)undecyl)triphenylphosphonium iodide ((*R*,*R*)-5)

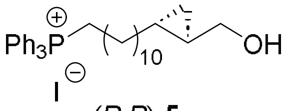

(*R*,*R*)-**5**

Phosphonium salt (*R*,*R*)-**5** was prepared following general procedure 6:
(*R*,*R*)-**4** (1.24 g, 4.06 mmol, 1.0 eq.) in CH_3CN (60 mL).
Triphenylphosphane (2.13 g, 8.12 mmol, 2.0 eq.).
Sodium iodide (3.04 g, 20.3 mmol, 5.0 eq.).
Flash chromatography [silica gel, CH_2Cl_2, then CH_2Cl_2/MeOH (20:1) bis (10:1)].
(*R*,*R*)-**5** (2.42 g, 3.5 mmol, 97%) as yellowish oil.

TLC [silica gel, CH_2Cl_2/MeOH (20:1)]: R_f = 0.17.

^{1}H-NMR (400 MHz, $CDCl_3$): δ = 7.87-7.77 (m, 9H, *m-/p*-Ph-C*H*), 7.76-7.68 (m, 6H, *o*-Ph-C*H*), 3.71-3.59 (m, 2H, PC*H*$_2$), 3.49-3.38 (m, 2H, C*H*$_2$OH), 1.75 (s, br, 1H, O*H*), 1.70-1.57 (m, 4H, PCH$_2$C*H*$_2$C*H*$_2$), 1.40-1.12 (m, 16H, (C*H*$_2$)$_8$), 0.87-0.77 (m, 1H, C*H*CHCH$_2$OH), 0.63-0.54 (m, 1H, C*H*CH$_2$OH), 0.39-0.25 (m, 2H, CHC*H*$_2$CH).

^{13}C-NMR (100 MHz, $CDCl_3$): δ = 135.1 (3C, d, *J* = 3.0 Hz, *p*-Ph-*C*H), 133.6 (6C, d, *J* = 10.0 Hz, *o*-Ph-*C*H), 130.5 (6C, d, *J* = 12.5 Hz, *m*-Ph-*C*H), 118.2 (3C, d, *J* = 9.9 Hz, *ipso*-Ph-*C*), 67.1 (*C*H$_2$OH), 33.5 (*C*H$_2$CHCHCH$_2$OH), 30.4 (d, *J* = 15.7 Hz, PCH$_2$CH$_2$*C*H$_2$), 29.5 (2C, *C*H$_2$), 29.4 (2C, *C*H$_2$), 29.3 (*C*H$_2$), 29.1 (2C, *C*H$_2$), 23.1 (d, *J* = 49.9 Hz, P*C*H$_2$), 22.6 (d, *J* = 4.5 Hz, PCH$_2$*C*H$_2$), 21.2 (*C*HCHCH$_2$OH), 17.1 (*C*HCH$_2$OH), 9.9 (CH*C*H$_2$CH).

HRMS (ESI) *m/z*: $[M- I]^+$ Calcd for $C_{33}H_{44}OP$ 487.3124; Found 487.3131

$[\alpha]_D^{21.0}$ = −4.44 (*c* = 2.70, $CDCl_3$).

((1*S*,2*S*)-2-((*E*)-12-(1-Trityl-1*H*-imidazol-4-yl)dodec-11-en-1-yl)cyclopropyl)-methanol ((*S*,*S*)-6)

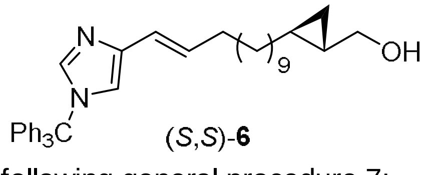

(*S*,*S*)-**6**

Alcohol (*S*,*S*)-**6** was prepared following general procedure 7:
Phosphonium salt (*S*,*S*)-**5** (1.99 g, 3.24 mmol, 1.0 eq.) in dry THF (100 mL).
Phenyllithium solution (3.90 mL, 7.02 mmol, 2.2 eq., 1.8 M in Bu_2O).
8 (1.19 g, 3.52 mmol, 1.1 eq.) in dry THF (30 mL).
Phenyllithium solution (3.90 mL, 7.02 mmol, 2.2 eq., 1.8 M in Bu_2O).
Flash chromatography [silica gel, *n*-pentane/EtOAc (3:1) to (1:1)].
(*S*,*S*)-**6** (1.04 g, 1.90 mmol, 59%) as yellowish solid.

TLC [silica gel, *n*-pentane /EtOAc (3:1)]: R_f = 0.10.

^{1}**H-NMR** (400 MHz, $CDCl_3$): δ = 7.38 (s, 1H, NC*H*N), 7.35-7.30 (m, 9H, *m*-/*p*-Ph-C*H*), 7.18-7.12 (m, 6H, *o*-Ph-C*H*), 6.66 (d, ^{4}J = 1.3 Hz, 1H, NCC*H*N), 6.35 (td, ^{3}J = 6.8 Hz, 15.6 Hz, 1H, NCCHC*H*), 6.20 (d, ^{3}J = 15.7 Hz, 1H, NCC*H*CH), 3.50-3.37 (m, 2H, C*H*$_2$OH), 2.18-2.11 (m, 2H, NCCHCHC*H*$_2$), 1.48-1.14 (m, 18H, (C*H*$_2$)$_9$), 0.87-0.78 (m, 1H, C*H*CHCH$_2$OH), 0.63-0.54 (m, 1H, C*H*CH$_2$OH), 0.39-0.26 (m, 2H, CHC*H*$_2$CH).

^{13}**C-NMR** (100 MHz, $CDCl_3$): δ = 142.3 (3C, *ipso*-Ph-*C*), 139.4 (N*C*CHN), 138.8 (N*C*HN), 130.1 (NCCH*C*H), 129.8 (6C, *o*-Ph-*C*H), 128.0 (9C, *m*-/*p*-Ph-*C*H), 121.2 (NC*C*HCH), 118.0 (NC*C*HN), 75.3 (*C*Ph$_3$), 67.1 (*C*H$_2$OH), 33.6 (*C*H$_2$CHCHCH$_2$OH), 32.8 (NCCHCH*C*H$_2$), 29.6 (3C, *C*H$_2$), 29.5 (2C, *C*H$_2$), 29.3 (2C, *C*H$_2$), 29.2 (*C*H$_2$), 21.2 (*C*HCHCH$_2$OH), 17.2 (*C*HCH$_2$OH), 9.9 (CH*C*H$_2$CH).

HRMS (ESI) *m/z*: $[M + H]^+$ Calcd for $C_{38}H_{47}N_2O$ 547.3688; Found 547.3686

$[\alpha]_D^{21.0}$ = +6.61 (*c* = 3.10, $CDCl_3$).

((1*R*,2*R*)-2-((*E*)-12-(1-Trityl-1*H*-imidazol-4-yl)dodec-11-en-1-yl)cyclopropyl)-methanol ((*R*,*R*)-6)

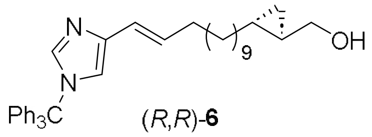

(*R*,*R*)-**6**

Alcohol (*R*,*R*)-**6** was prepared following general procedure 7:
Phosphonium salt (*R*,*R*)-**5** (2.36 g, 3.84 mmol, 1.0 eq.) in dry THF (120 mL).
Phenyllithium solution (4.62 mL, 8.32 mmol, 2.2 eq., 1.8 M in Bu_2O).
8 (1.41 g, 4.17 mmol, 1.1 eq.) in dry THF (40 mL).
Phenyllithium solution (4.62 mL, 8.32 mmol, 2.2 eq., 1.8 M in Bu_2O).
Flash chromatography [silica gel, *n*-pentane/EtOAc (3:1) to (1:1)]
(*R*,*R*)-**6** (648 mg, 1.19 mmol, 31%) as colorless solid.

TLC [silica gel, *n*-pentane/EtOAc (1:1)]: R_f = 0.38.

^{1}**H-NMR** (400 MHz, $CDCl_3$): δ = 7.38 (s, 1H, NC*H*N), 7.35-7.30 (m, 9H, *m*-/*p*-Ph-C*H*), 7.18-7.11 (m, 6H, *o*-Ph-C*H*), 6.66 (d, ^{4}J = 1.3 Hz, 1H, NCC*H*N), 6.35 (td, ^{3}J = 6.8 Hz, 15.6 Hz, 1H, NCCHC*H*), 6.20 (d, ^{3}J = 15.7 Hz, 1H, NCC*H*CH), 3.50-3.36 (m, 2H, C*H*$_2$OH), 2.18-2.11 (m, 2H, NCCHCHC*H*$_2$), 1.48-1.15 (m, 18H, (C*H*$_2$)$_9$), 0.87-0.77 (m, 1H, C*H*CHCH$_2$OH), 0.63-0.54 (m, 1H, C*H*CH$_2$OH), 0.39-0.26 (m, 2H, CHC*H*$_2$CH).

^{13}**C-NMR** (100 MHz, $CDCl_3$): δ = 142.3 (3C, *ipso*-Ph-*C*), 139.4 (N*C*CHN), 138.8 (N*C*HN), 130.1 (NCCH*C*H), 129.8 (6C, *o*-Ph-*C*H), 128.0 (9C, *m*-/*p*-Ph-*C*H), 121.2 (NC*C*HCH), 118.0 (NC*C*HN), 75.3 (*C*Ph$_3$), 67.2 (*C*H$_2$OH), 33.6 (*C*H$_2$CHCHCH$_2$OH), 32.9 (NCCHCH*C*H$_2$), 29.6 (3C, *C*H$_2$), 29.5 (2C, *C*H$_2$), 29.3 (2C, *C*H$_2$), 29.2 (*C*H$_2$), 21.2 (*C*HCHCH$_2$OH), 17.2 (*C*HCH$_2$OH), 9.9 (CH*C*H$_2$CH).

HRMS (ESI) *m/z*: $[M + H]^+$ Calcd for $C_{38}H_{47}N_2O$ 547.3688; Found 547.3685

$[\alpha]_D^{20.1}$ = −5.56 (*c* = 2.25, $CDCl_3$).

(1*S*,2*S*)-2-((*E*)-12-(1-Trityl-1*H*-imidazol-4-yl)dodec-11-en-1-yl)cyclopropane-1-carbaldehyde

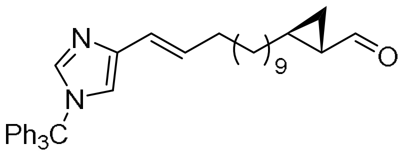

This aldehyde was prepared following general procedure 2:
Alcohol (*S*,*S*)-**6** (991 mg, 1.81 mmol, 1.0 eq.) in dry CH_2Cl_2 (25 mL).
Triethylamine (2.52 mL, 18.1 mmol, 10 eq.).
Py·SO_3 (865 mg, 5.43 mmol, 3.0 eq.) in dry DMSO (10 mL).
Flash chromatography [silica gel, *n*-pentane/EtOAc (5:1)].
(1*S*,2*S*)-2-((*E*)-12-(1-Trityl-1*H*-imidazol-4-yl)dodec-11-en-1-yl)cyclopropane-1-carbaldehyde (736 mg, 1.35 mmol, 75%) as colorless solid.

TLC [silica gel, *n*-pentane/EtOAc (3:1)]: R_f = 0.31.

^{1}H-NMR (400 MHz, $CDCl_3$): δ = 8.99 (d, ^3J = 5.6 Hz, 1H, C*H*O), 7.38 (s, 1H, NC*H*N), 7.35-7.30 (m, 9H, *m*-/*p*-Ph-C*H*), 7.18-7.12 (m, 6H, *o*-Ph-C*H*), 6.67 (d, ^4J = 1.3 Hz, 1H, NCC*H*N), 6.40-6.31 (m, 1H, NCCHC*H*), 6.21 (d, ^3J = 15.7 Hz, 1H, NCC*H*CH), 2.19-2.11 (m, 2H, NCCHCHCH_2), 1.65-1.58 (m, 1H, C*H*CHO), 1.51-1.20 (m, 20H, $(CH_2)_9$, C*H*CHCHO, CHC[*H*]$_2$CH), 0.6-0.89 (m, 1H, CHC[*H*]$_2$CH).

^{13}C-NMR (100 MHz, $CDCl_3$): δ = 201.1 (*C*HO), 142.4 (3C, *ipso*-Ph-*C*), 139.5 (N*C*CHN), 138.9 (N*C*HN), 129.9 (NCCH*C*H), 129.8 (6C, *o*-Ph-*C*H), 128.0 (9C, *m*-/*p*-Ph-*C*H), 121.3 (NC*C*HCH), 118.0 (NC*C*HN), 75.2 (*C*Ph_3), 32.8 (NCCHCH*C*H_2), 32.6 (*C*H_2CHCHCHO), 30.5 (*C*HCHO), 29.5 (4C, *C*H_2), 29.3 (*C*H_2), 29.2 (2C, *C*H_2), 29.1 (*C*H_2), 22.7 (*C*HCHCHO), 14.9 (CH*C*H_2CH).

HRMS (ESI) *m/z*: $[M + H]^+$ Calcd for $C_{38}H_{45}N_2O$ 545.3526; Found 545.3529

$[\alpha]_D^{20.2}$ = +12.5 (*c* = 2.19, $CDCl_3$).

(1*R*,2*R*)-2-((*E*)-12-(1-Trityl-1*H*-imidazol-4-yl)dodec-11-en-1-yl)cyclopropane-1-carbaldehyde

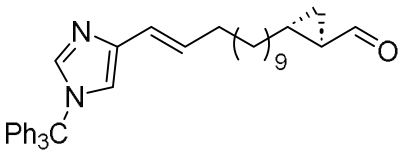

This aldehyde was prepared following general procedure 2:
Alcohol (*R*,*R*)-**6** (624 mg, 1.14 mmol, 1.0 eq.) in dry CH_2Cl_2 (20 mL).
Triethylamine (1.59 mL, 11.4 mmol, 10 eq.).
Py·SO_3 (545 mg, 3.42 mmol, 3.0 Eq..) in dry DMSO (7 mL).
Flash chromatography [silica gel, *n*-pentane/EtOAc (5:1) to (3:1)].
(1*R*,2*R*)-2-((*E*)-12-(1-Trityl-1*H*-imidazol-4-yl)dodec-11-en-1-yl)cyclopropane-1-carbaldehyde (420 mg, 0.77 mmol, 68%) as colorless solid.

TLC [silica gel, *n*-pentane/EtOAc (5:1)]: R_f = 0.18.

^{1}H-NMR (400 MHz, $CDCl_3$): δ = 8.99 (d, ^3J = 5.6 Hz, 1H, C*H*O), 7.38 (s, 1H, NC*H*N), 7.36-7.30 (m, 9H, *m*-/*p*-Ph-C*H*), 7.18-7.12 (m, 6H, *o*-Ph-C*H*), 6.67 (d, ^4J = 1.3 Hz, 1H, NCC*H*N), 6.35 (td, ^3J = 7.0 Hz, 15.6 Hz, 1H, NCCHC*H*), 6.21 (d, ^3J = 15.8 Hz, 1H, NCC*H*CH), 2.19-2.11 (m, 2H,

NCCHCHCH_2), 1.65-1.58 (m, 1H, C*H*CHO), 1.51-1.19 (m, 20H, (C*H*$_2$)$_9$, C*H*CHCHO, CHC[*H*]$_2$CH), 0.7-0.89 (m, 1H, CHC[*H*]$_2$CH).

^{13}C-NMR (100 MHz, $CDCl_3$): δ = 201.1 (*C*HO), 142.4 (3C, *ipso*-Ph-*C*), 139.5 (N*C*CHN), 138.9 (N*C*HN), 129.9 (NCCH*C*H), 129.8 (6C, *o*-Ph-*C*H), 128.0 (9C, *m*-/*p*-Ph-*C*H), 121.3 (NC*C*HCH), 118.0 (NC*C*HN), 75.2 (*C*Ph_3), 32.8 (NCCHCH*C*H_2), 32.6 (*C*H_2CHCHCHO), 30.5 (*C*HCHO), 29.5 (4C, *C*H_2), 29.3 (*C*H_2), 29.2 (2C, *C*H_2), 29.1 (*C*H_2), 22.7 (*C*HCHCHO), 14.9 (CH*C*H_2CH).

HRMS (ESI) *m/z*: $[M + H]^+$ Calcd for $C_{38}H_{45}N_2O$ 545.3526; Found 545.3528

$[\alpha]_D^{20.2}$ = −10.8 (*c* = 2.13, $CDCl_3$).

(1*S*,2*S*)-2-((*E*)-12-(1-Trityl-1*H*-imidazol-4-yl)dodec-11-en-1-yl)cyclopropane-1-carboxylic acid ((*S*,*S*)-7)

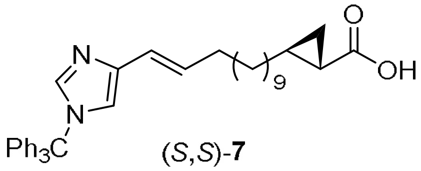

(*S*,*S*)-**7**

Acid (*S*,*S*)- **7** was prepared following general procedure 8:
(1*S*,2*S*)-2-((*E*)-12-(1-Trityl-1*H*-imidazol-4-yl)dodec-11-en-1-yl)cyclopropane-1-carbaldehyde (678 mg, 1.24 mmol, 1.0 eq.) in *tert*-BuOH (30 mL)/THF (25 mL) and 2-methyl-2-butene (5 mL).
$NaClO_2$ (788 mg, 8.71 mmol, 7.0 eq.), NaH_2PO_4 (1.55 g, 11.2 mmol, 9.0 eq.) in water (10 mL).
Flash chromatography [silica gel, $CHCl_3$/MeOH/NH_3 (90:10:1)].
(*S*,*S*)-**7** (527 mg, 0.5 mmol, 76%) as colorless oil.

TLC [silica gel, $CHCl_3$/MeOH/NH_3 (90:10:1)]: R_f = 0.20.

^{1}H-NMR (400 MHz, $CDCl_3$): δ = 7.51 (d, ^4J = 1.2 Hz, 1H, NC*H*N), 7.35-7.30 (m, 9H, *m*-/*p*-Ph-C*H*), 7.17-7.10 (m, 6H, *o*-Ph-C*H*), 6.64 (d, ^4J = 1.3 Hz, 1H, NCC*H*N), 6.34 (td, ^3J = 6.9 Hz, 15.7 Hz, 1H, NCCHC*H*), 6.18 (d, ^3J = 15.8 Hz, 1H, NCC*H*CH), 2.19-2.10 (m, 2H, NCCHCHC*H*$_2$), 1.49-1.11 (m, 21H, (C*H*$_2$)$_9$, C*H*C[*H*]$_2$C*H*), 0.71-0.63 (m, 1H, CHC[*H*]$_2$CH).

^{13}C-NMR (100 MHz, $CDCl_3$): δ = 178.6 (*C*O_2H), 142.1 (3C, *ipso*-Ph-*C*), 138.9 (N*C*CHN), 138.7 (N*C*HN), 130.8 (NCCH*C*H), 129.8 (6C, *o*-Ph-*C*H), 128.1 (9C, *m*-/*p*-Ph-*C*H), 120.6 (NC*C*HCH), 118.0 (NC*C*HN), 75.5 (*C*Ph_3), 32.8 (*C*H_2CHCHCO_2H), 32.7 (NCCHCH*C*H_2), 29.2 (*C*H_2), 29.1 (3C, *C*H_2), 29.0 (*C*H_2), 28.9 (*C*H_2), 28.8 (*C*H_2), 28.6 (*C*H_2), 23.3 (*C*HCHCO_2H), 20.3 (*C*HCO_2H), 15.8 (CH*C*H_2CH).

$[\alpha]_D^{19.6}$ = +17.0 (*c* = 2.79, $CDCl_3$).

(1*R*,2*R*)-2-((*E*)-12-(1-Trityl-1*H*-imidazol-4-yl)dodec-11-en-1-yl)cyclopropane-1-carboxylic acid ((*R*,*R*)-7)

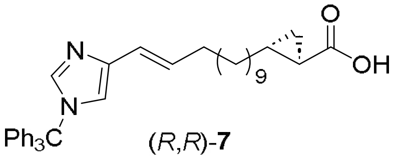

(*R*,*R*)-**7**

Acid (*R*,*R*)- **7** was prepared following general procedure 8:
(1*R*,2*R*)-2-((*E*)-12-(1-Trityl-1*H*-imidazol-4-yl)dodec-11-en-1-yl)cyclopropane-1-carbaldehyde (400 mg, 0.73 mmol, 1.0 eq.) in *tert*-BuOH (18 mL)/THF (15 mL) and 2-methyl-2-butene (3 mL).

$NaClO_2$ (465 mg, 5.14 mmol, 7.0 eq.), NaH_2PO_4 (912 mg, 6.61 mmol, 9.0 eq.) in water (6 mL).
Flash chromatography [silica gel, $CHCl_3$/MeOH/NH_3 (90:10:1)].
(*R*,*R*)-**97** (281 mg, 0.50 mmol, 68%) as yellowish oil.

TLC [silica gel, $CHCl_3$/MeOH/NH_3 (90:10:1)]: R_f = 0.20.

^{1}H-NMR (400 MHz, $CDCl_3$): δ = 7.52 (s, 1H, NC*H*N), 7.35-7.30 (m, 9H, *m*-/*p*-Ph-C*H*), 7.17-7.09 (m, 6H, *o*-Ph-C*H*), 6.63 (d, ^4J = 1.3 Hz, 1H, NCC*H*N), 6.35 (td, ^3J = 6.9 Hz, 15.6 Hz, 1H, NCCHC*H*), 6.18 (d, ^3J = 15.8 Hz, 1H, NCC*H*CH), 2.19-2.10 (m, 2H, NCCHCHC*H*$_2$), 1.53-1.06 (m, 21H, (C*H*$_2$)$_9$, C*H*C[*H*]$_2$C*H*), 0.70-0.63 (m, 1H, CHC[*H*]$_2$CH).

^{13}C-NMR (100 MHz, $CDCl_3$): δ = 178.3 (*C*O$_2$H), 142.1 (3C, *ipso*-Ph-*C*), 138.8 (N*C*CHN), 138.7 (N*C*HN), 131.0 (NCCH*C*H), 129.8 (6C, *o*-Ph-*C*H), 128.1 (9C, *m*-/*p*-Ph-*C*H), 120.4 (NC*C*HCH), 118.0 (NC*C*HN), 75.6 (*C*Ph$_3$), 32.8 (*C*H$_2$CHCHCO$_2$H), 32.7 (NCCHCH*C*H$_2$), 29.1 (2C, *C*H$_2$), 29.0 (2C, *C*H$_2$), 28.9 (2C, *C*H$_2$), 28.6 (*C*H$_2$), 28.4 (*C*H$_2$), 23.0 (*C*HCHCO$_2$H), 20.5 (*C*HCO$_2$H), 15.5 (CH*C*H$_2$CH).

$[\alpha]_D^{19.6}$ = −12.9 (*c* = 1.82, $CDCl_3$).

(1*S*,2*S*)-2-((*E*)-12-(1*H*-Imidazol-4-yl)dodec-11-en-1-yl)cyclopropane-1-carboxylic acid ((*S*,*S*)-9)

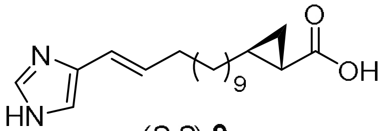

(*S*,*S*)-**9**

Acid (*S*,*S*)-**9** was prepared following general procedure 9:
(*S*,*S*)-**7** (43 mg, 0.88 mmol, 1.0 eq.).
Acetic acid (4.00 mL) in methanol (40 mL).
Flash chromatography [silica gel, $CHCl_3$/MeOH/NH_3 (70:10:1) to (40:10:1)].
(*S*,*S*)-**9** (208 mg, 0.65 mmol, 74%) as colorless solid.

TLC [silica gel, $CHCl_3$/MeOH/NH_3 (40:10:1)]: R_f = 0.10.

^{1}H-NMR (600 MHz, CD_3OD + 4 µL NH_3): δ = 7.56 (s, 1H, NC*H*N), 6.90 (s, 1H, NCC*H*N), 6.26 (d, ^3J = 15.9 Hz, 1H, NCC*H*CH), 6.13 (td, ^3J = 6.9 Hz, 15.9 Hz, 1H, NCCHC*H*), 2.19-2.14 (m, 2H, NCCHCHC*H*$_2$), 1.49-1.14 (m, 20H, (C*H*$_2$)$_9$, C*H*C*H*CO$_2$H), 0.97-0.4 (m, 1H, CHC[*H*]$_2$CH), 0.46-0.42 (m, 1H, CHC[*H*]$_2$CH).

^{13}C-NMR (150 MHz CD_3OD + 4 µL NH_3): δ = 183.4 (*C*O$_2$H), 136.4 (N*C*HN), 136.1 (N*C*CHN), 130.3 (NCCH*C*H), 120.6 (NC*C*HCH), 119.7 (NC*C*HN), 34.7 (*C*H$_2$CHCHCO$_2$H), 33.9 (NCCHCH*C*H$_2$), 30.8 (*C*H$_2$), 30.7 (2C, *C*H$_2$), 30.6 (3C, *C*H$_2$), 30.5 (*C*H$_2$), 30.3 (2C, *C*H$_2$), 24.3 (*C*HCHCO$_2$H), 22.2 (*C*HCO$_2$H), 14.9 (CH*C*H$_2$CH).

HRMS (ESI) *m/z*: $[M + H]^+$ Calcd for $C_{19}H_{31}N_2O_2$ 319.2380; Found 319.2377

$[\alpha]_D^{21.3}$ = +25.4 (*c* = 0.57, 1% NH_3 in MeOH).

(1*R*,2*R*)-2-((*E*)-12-(1*H*-Imidazol-4-yl)dodec-11-en-1-yl)cyclopropane-1-carboxylic acid ((*R*,*R*)-9)

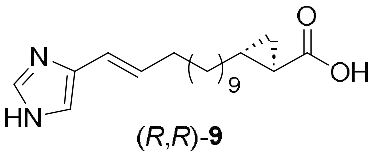

(*R*,*R*)-**9**

Acid (*R*,*R*)-**9** was prepared following general procedure 9:
(*R*,*R*)-**7** (253 mg, 0.45 mmol, 1.0 eq.).
Acetic acid (2.50 mL) in methanol (25 mL).
Flash chromatography [silica gel, $CHCl_3$/MeOH/NH_3 (70:10:1) to (40:10:1)].
(*R*,*R*)-**9** (72 mg, 0.23 mmol, 50%) as colorless solid.

TLC [silica gel, $CHCl_3$/MeOH/NH_3 (40:10:1)]: R_f = 0.10.

^{1}H-NMR (600 MHz, CD_3OD + 4 µL NH_3): δ = 7.56 (s, 1H, NC*H*N), 6.90 (s, 1H, NCC*H*N), 6.26 (d, ^3J = 15.9 Hz, 1H, NCC*H*CH), 6.13 (td, ^3J = 6.9 Hz, 15.9 Hz, 1H, NCCHC*H*), 2.19-2.14 (m, 2H, NCCHCHC*H*$_2$), 1.49-1.13 (m, 20H, (C*H*$_2$)$_9$, C*H*C*H*CO$_2$H), 0.97-0.4 (m, 1H, CHC[*H*]$_2$CH), 0.47-0.43 (m, 1H, CHC[*H*]$_2$CH).

^{13}C-NMR (150 MHz, CD_3OD + 4 µL NH_3): δ = 183.4 (*C*O$_2$H), 136.5 (N*C*HN), 136.2 (N*C*CHN), 130.3 (NCCH*C*H), 120.6 (NC*C*HCH), 119.7 (NC*C*HN), 34.7 (*C*H$_2$CHCHCO$_2$H), 34.0 (NCCHCH*C*H$_2$), 30.8 (*C*H$_2$), 30.7 (2C, *C*H$_2$), 30.6 (3C, *C*H$_2$), 30.5 (*C*H$_2$), 30.3 (2C, *C*H$_2$), 24.3 (*C*HCHCO$_2$H), 22.2 (*C*HCO$_2$H), 14.9 (CH*C*H$_2$CH).

HRMS (ESI) *m/z*: $[M + H]^+$ Calcd for $C_{19}H_{31}N_2O_2$ 319.2380; Found 319.2375

$[\boldsymbol{\alpha}]_{\boldsymbol{D}}^{\boldsymbol{21.4}}$ = −19.4 (*c* = 0.49, 1% NH_3 in MeOH).

5.7 Synthesis of imidacin A2

14-Bromotetradecan-1-ol (40)

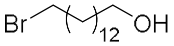

40

According to the literature,[S20] $LiAlH_4$ (3.00 g, 79.1 mmol, 3.5 eq.) was suspended in dry THF (100 mL) at 0 °C followed by slow addition of a solution of 1,14-tetradecanedioic acid (5.84 g, 22.6 mmol, 1.0 eq.) in dry THF (150 mL). Ice water was added after stirring for 19 h at r.t and the formed precipitate was dissolved by adding 10% H_2SO_4. The layers were separated and the organic layer was washed with sat. NaCl solution (3 x 50 mL). After drying with $MgSO_4$ and removal of the solvent under reduced pressure, 1,14-tetradecandiol was obtained and directly used without further purification for the preparation of 14-bromotetradecan-1-ol following general procedure 1:
HBr (31.0 mL, 272 mmol, 12 eq., 48% in H_2O).
Flash chromatography [silica gel, *n*-pentane/EtOAc (10:1) to (5:1)].
40 (4.20 g, 14.3 mmol, 63% over 2 steps) as colorless solid.
The spectroscopic data matched those reported earlier.[S20]

TLC [silica gel, *n*-pentane/EtOAc (10:1)]: R_f = 0.23.

^{1}**H-NMR** (400 MHz, $CDCl_3$): δ = 3.64 (t, ^3J = 6.6 Hz, 2H, CH_2OH), 3.41 (t, ^3J = 6.9 Hz, 2H, CH_2Br), 1.89-1.81 (m, 2H, C$H_2$$CH_2$Br), 1.61-1.52 (m, 2H, C$H_2$$CH_2$OH), 1.47-1.22 (m, 20H, (CH_2)$_{10}$).

^{13}**C-NMR** (100 MHz, $CDCl_3$): δ = 63.1 (*C*H_2OH), 34.0 (*C*H_2Br), 32.8 (2C, *C*$H_2$$CH_2$Br, *C*$H_2$$CH_2$OH), 29.6 (4C, *C*$H_2$), 29.5 (*C*$H_2$), 29.4 (2C, *C*$H_2$), 28.8 (*C*$H_2$), 28.2 (*C*$H_2$), 25.7 (*C*$H_2$).

IR (ATR): $\tilde{\nu}$ = 3271 (br), 2917 (s), 2849 (s), 1733 (w), 1463 (m), 1413 (w), 1349 (w), 1272 (w), 1245 (w), 1224 (w), 1202 (w), 1122 (w), 1069 (m), 1040 (m), 1005 (w), 67 (w), 30 (w), 879 (w), 719 (m), 649 (m).

MS (EI): *m/z* (%) = 292/294 (missing) [M^+], 162/164 (21/20), 151 (6), 149 (5), 148/150 (34/35), 135/137 (13/15), 123/125 (9/10), 111 (24), 109 (12), 98 (10), 97 (67), 7 (11), 6 (13), 9 (6), 84 (15), 83 (86), 82 (39), 81 (18), 71 (13), 70 (25), 69 (5), 68 (38), 67 (24), 57 (31), 56 (26), 55 (100), 54 (11), 53 (6), 43 (31), 42 (13), 41 (52), 39 (8).

HRMS (CI) *m/z*: $[M - H]^+$ Calcd for $C_{14}H_{28}BrO$ 291.1324; Found 291.1317

Ethyl (*E*)-16-bromehexadec-2-enoate (41)

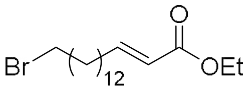

41

This ester was prepared in 2 steps following general procedure 2 and 3:
14-Bromotetradecan-1-ol (4.11 g, 14.0 mmol, 1.0 eq.) in dry CH_2Cl_2 (80 mL).
Triethylamine (19.5 mL, 140 mmol, 10 eq.).
Py·SO_3 (6.69 g, 42.0 mmol, 3.0 eq.) in dry DMSO (40 mL).
The resulting aldehyde 14-bromotetradecanal was used directly in the next step in dry Et_2O (50 mL).

NaH (841 mg, 21.0 mmol, 1.5 eq., 60% in mineral oil) in dry Et_2O (50 mL).
Triethyl phosphonoacetate (7.00 mL, 35.0 mmol, 2.5 eq.).
Flash chromatography [silica gel, *n*-pentane/TBME (100:1) to (50:1)].
41 (3.77 g, 10.4 mmol, 74% over 2 steps) as colorless oil.

TLC [silica gel, *n*-pentane/Et_2O (50:1)]: R_f = 0.31.

^{1}**H-NMR** (400 MHz, $CDCl_3$): δ = 6.7 (td, ^3J = 7.0 Hz, 15.6 Hz, 1H, C*H*CHCO$_2$Et), 5.81 (td, ^4J = 1.6 Hz, ^3J = 15.6 Hz, 1H, C*H*CO$_2$Et), 4.18 (q, ^3J = 7.1 Hz, 2H, OCH_2CH$_3$), 3.41 (t, ^3J = 6.9 Hz, 2H, CH_2Br), 2.23-2.15 (m, 2H, CH_2CHCHCO$_2$Et), 1.90-1.81 (m, 2H, CH_2CH$_2$Br), 1.50-1.21 (m, 23H, (CH_2)$_{10}$, OCH$_2$CH_3).

^{13}**C-NMR** (100 MHz, $CDCl_3$): δ = 166.8 (*C*O$_2$Et), 149.4 (*C*HCHCO$_2$Et), 121.2 (*C*HCO$_2$Et), 60.1 (O*C*H$_2$CH$_3$), 34.0 (*C*H$_2$Br), 32.8 (*C*H$_2$CH$_2$Br), 32.2 (*C*H$_2$CHCHCO$_2$Et), 29.6 (2C, *C*H$_2$), 29.5 (2C, *C*H$_2$), 29.4 (2C, *C*H$_2$), 29.1 (*C*H$_2$), 28.7 (*C*H$_2$), 28.2 (*C*H$_2$), 28.0 (*C*H$_2$), 14.3 (OCH$_2$*C*H$_3$).

IR (ATR): $\tilde{\nu}$ = 234 (s), 293 (m), 1719 (s), 1654 (m), 1463 (w), 1367 (w), 1308 (w), 1264 (m), 1178 (m), 1128 (w), 107 (w), 1043 (m), 979 (m), 96 (w), 721 (w), 645 (w), 562 (w).

UV/VIS (CH_2Cl_2): λ_{max} (log ε) = 229 (3.42).

MS (EI): *m/z* (%) = 360/362 (1/1) [M^+], 317 (22), 316 (11), 315 (21), 314 (8), 281 (8), 272/274 (18/15), 235 (11), 234 (9), 232 (6), 230 (7), 155 (21), 152 (5), 151 (9), 150 (8), 148 (5), 142 (8),

141 (16), 138 (7), 137 (16), 135 (9), 129 (5), 128 (11), 127 (34), 125 (7),124 (8), 123 (25), 121 (8), 115 (26), 114 (17), 113 (7), 112 (5), 111 (15), 110 (19), 109 (33), 108 (5), 107 (9), 102 (7), 101 (100), 100 (5), 99 (45), 98 (29), 97 (43), 7 (49), 6 (48), 5 (8), 4 (7), 89 (9), 88 (52), 87 (6), 86 (19), 9 (10), 84 (36), 83 (41), 82 (27), 81 (61), 80 (7), 79 (11), 73 (41), 71 (11), 70 (17), 69 (52), 68 (29), 67 (35), 61 (5), 57 (23), 56 (11), 55 (91), 54 (13), 53 (12), 43 (30), 42 (9), 41 (44), 39 (9).

HRMS (CI) *m/z*: $[M + H]^+$ Calcd for $C_{18}H_{34}BrO_2$ 361.1742; Found 361.1737

(*E*)-16-Bromohexadec-2-en-1-ol (42)

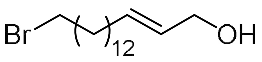

42

(*E*)-16-Bromohexadec-2-en-1-ol (**42**) was prepared following general procedure 4:
Ethyl (*E*)-16-bromehexadec-2-enoate (1.89 g, 5.23 mmol, 1.0 eq.) in CH_2Cl_2 (50 mL).
DIBAL-*H* solution (15.7 mL, 15.7 mmol, 3.0 eq., 1.0 M in CH_2Cl_2).
Flash chromatography [silica gel, CH_2Cl_2].
42 (1.54 g, 4.82 mmol, 92%) as colorless solid.

TLC [silica gel, CH_2Cl_2]: R_f = 0.24.

^{1}H-NMR (400 MHz, $CDCl_3$): δ = 5.74-5.58 (m, 2H, C*H*C*H*CH_2OH), 4.10-4.07 (m, 2H, C*H*$_2$OH), 3.41 (t, ^3J = 6.9 Hz, 2H, C*H*$_2$Br), 2.07-2.00 (m, 2H, C*H*$_2$CHCHCH_2OH), 1.89-1.81 (m, 2H, C*H*$_2$CH_2Br), 1.47-1.23 (m, 20H, (C*H*$_2$)$_{10}$).

^{13}C-NMR (100 MHz, $CDCl_3$): δ = 133.5 (*C*HCHCH_2OH), 128.8 (*C*HCH_2OH), 63.8 (*C*H_2OH), 34.0 (*C*H_2Br), 32.8 (*C*H_2CH_2Br), 32.2 (*C*H_2CHCHCH_2OH), 29.6 (3C, *C*H_2), 29.5 (2C, *C*H_2), 29.4 (*C*H_2), 29.2 (*C*H_2), 29.1 (*C*H_2), 28.7 (*C*H_2), 28.1 (*C*H_2).

IR (ATR): $\tilde{\nu}$ = 3359 (br), 3280 (br), 3003 (w), 2916 (s), 2848 (m), 1674 (w), 1466 (m), 1359 (w), 1305 (w), 1271 (w), 1226 (w), 1202 (w), 1087 (m), 991 (m), 972 (s), 720 (m), 675 (m), 648 (s), 539 (w).

MS (EI): *m/z* (%) = 318/320 (<1) [M^+], 162/164 (5/4), 148/150 (8/8), 137 (11), 135 (7), 124 (7), 123 (16), 111 (10), 110 (15), 109 (33), 107 (6), 98 /5), 97 (36), 7 (61), 6 (54), 5 (5), 4 (5), 9 (8), 84 (8), 83 (55), 82 (100), 81 (62), 80 (8), 79 (14), 72 (8), 71 (24), 70 (18), 69 (55), 68 (57), 67 (61), 66 (8), 58 (7), 57 (98), 56 (15), 55 (80), 54 (28), 53 (9), 44 (9), 43 (33), 42 (10), 41 (54), 39 (10).

HRMS (CI) *m/z*: $[M - H]^+$ Calcd for $C_{16}H_{30}BrO$ 317.1480; Found 317.1474

((1*RS*,2*RS*)-2-(13-Bromotridecyl)cyclopropyl)methanol (43)

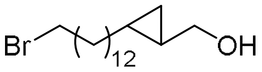

43

43 was prepared following general procedure 5:
42 (1.50 g, 4.70 mmol, 1.0 eq.) in dry CH_2Cl_2 (80 mL).
Diethylzinc solution (23.5 mL, 23.5 mmol, 5.0 eq., 1.0 M in *n*-hexane).

Diiodomethane (1.90 mL, 4.7 mmol, 5.0 eq.).
Flash chromatography [silica gel, *n*-pentane/EtOAc (10:1)].
43 (1.53 g, 4.59 mmol, 98%) as colorless solid.

TLC [silica gel, *n*-pentane/EtOAc (10:1)]: R_f = 0.36.

^{1}H-NMR (400 MHz, $CDCl_3$): δ = 3.49-3.38 (m, 4H, C*H*$_2$OH, C*H*$_2$Br), 1.90-1.81 (m, 2H, C*H*$_2$CH$_2$Br), 1.48-1.15 (m, 22H, (C*H*$_2$)$_{11}$), 0.88-0.78 (m, 1H, C*H*CHCH$_2$OH), 0.64-0.54 (m, 1H, C*H*CH$_2$OH), 0.39-0.27 (m, 2H, CHC*H*$_2$CH).

^{13}C-NMR (100 MHz, $CDCl_3$): δ = 67.2 (*C*H$_2$OH), 34.0 (*C*H$_2$Br), 33.6 (*C*H$_2$CHCHCH$_2$OH), 32.8 (*C*H$_2$CH$_2$Br), 29.6 (5C, *C*H$_2$), 29.5 (*C*H$_2$), 29.4 (2C, *C*H$_2$), 28.7 (*C*H$_2$), 28.2 (*C*H$_2$), 21.1 (*C*HCHCH$_2$OH), 17.2 (*C*HCH$_2$OH), 9.9 (CH*C*H$_2$CH).

IR (ATR): $\tilde{\nu}$ = 3334 (w), 3253 (w), 3074 (w), 3001 (w), 2916 (s), 2848 (s), 1465 (m), 1447 (m), 1372 (w), 1321 (w), 1274 (w), 1247 (w), 1226 (w), 1204 (w), 1155 (w), 1070 (m), 1051 (m), 1029 (m), 906 (w), 92 (w), 791 (w), 723 (m), 647 (s).

UV/VIS (CH_2Cl_2): λ_{max} (log ε) = 227 (1.87).

MS (EI): *m/z* (%) = 332/334 (missing) [M$^+$], 314/316 (3/3), 291/24 (8/8), 203 (5), 191 (5), 177 (5), 164 (7), 163 (5), 162 (7), 151 (7), 150 (12), 149 (5), 148 (12), 137 (21), 135 (8), 125 (11), 124 (7), 123 (25), 121 (5), 111 (36), 110 (14), 109 (40), 107 (8), 98 (9), 97 (79), 7 (54), 6 (60), 5 (5), 4 (7), 9 (10), 84 (11), 83 (81), 82 (72), 81 (81), 80 (10), 79 (23), 77 (6), 71 (24), 70 (16), 69 (79), 68 (65), 67 (75), 66 (7), 65 (5), 58 (5), 57 (55), 56 (17), 55 (100), 54 (32), 53 (13), 44 (11), 43 (33), 42 (11), 41 (65), 39 (13).

HRMS (CI) *m/z*: [M - H]$^+$ Calcd for $C_{17}H_{32}BrO$ 331.1637; Found 331.1631

(13-((1*RS*,2*RS*)-2-(Hydroxymethyl)cyclopropyl)tridecyl)triphenylphosphonium iodide (44)

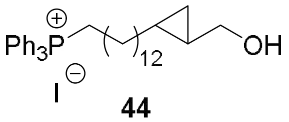

44

44 was prepared following general procedure 6:
43 (1.49 g, 4.47 mmol, 1.0 eq.) in CH_3CN (80 mL).
Triphenylphosphane (2.34 g, 8.3 mmol, 2.0 eq.).
Sodium iodide (4.57 g, 22.3 mmol, 5.0 eq.).
Flash chromatography [silica gel, CH_2Cl_2, then CH_2Cl_2/MeOH (20:1)].
44 (2.72 g, 4.23 mmol, 95%) as colorless oil.

TLC [silica gel, CH_2Cl_2/MeOH (20:1)]: R_f = 0.18.

^{1}H-NMR (400 MHz, $CDCl_3$): δ = 7.87-7.78 (m, 9H, *m*-/*p*-Ph-C*H*), 7.77-7.70 (m, 6H, *o*-Ph-C*H*), 3.65-3.52 (m, 2H, PC*H*$_2$), 3.50-3.36 (m, 2H, C*H*$_2$OH), 1.72 (s, br, 1H, O*H*), 1.69-1.58 (m, 4H, PCH$_2$C*H*$_2$C*H*$_2$), 1.42-1.15 (m, 20H, (C*H*$_2$)$_{10}$), 0.87-0.78 (m, 1H, C*H*CHCH$_2$OH), 0.63-0.54 (m, 1H, C*H*CH$_2$OH), 0.39-0.25 (m, 2H, CHC*H*$_2$CH).

^{13}C-NMR (100 MHz, $CDCl_3$): δ = 135.0 (3C, d, *J* = 3.0 Hz, *p*-Ph-*C*H), 133.4 (6C, d, *J* = 10.0 Hz, *o*-Ph-*C*H), 130.4 (6C, d, *J* = 12.5 Hz, *m*-Ph-*C*H), 117.9 (3C, d, *J* = 86.0 Hz, *ipso*-Ph-*C*), 66.8 (*C*H$_2$OH), 33.4 (*C*H$_2$CHCHCH$_2$OH), 30.3 (d, *J* = 15.5 Hz, PCH$_2$CH$_2$*C*H$_2$), 29.4 (*C*H$_2$), 29.3 (4C,

CH_2), 29.2 (*C*H_2), 29.1 (*C*H_2), 29.0 (*C*H_2), 28.9 (*C*H_2), 22.9 (d, J = 50.1 Hz, P*C*H_2), 22.4 (d, J = 4.5 Hz, PCH_2*C*H_2), 20.9 (*C*HCHCH_2OH), 16.9 (*C*HCH_2OH), 9.8 (CH*C*H_2CH).

IR (ATR): $\tilde{\nu}$ = 3372 (br), 3055 (w), 24 (w), 231 (m), 291 (m), 1587 (w), 1484 (w), 1462 (w), 1437 (m), 1317 (w), 1269 (w), 1189 (w), 1111 (m), 1029 (m), 97 (m), 919 (w), 789 (w), 723 (s), 689 (s).

UV/VIS (MeOH): λ_{max} (log ε) = 274 (3.39), 267 (3.47), 222 (4.54), 203 (4.67).

MS (ESI+): *m/z* (%) = 515.3 (100) $[M-I]^+$.

HRMS (ESI) *m/z*: $[M + I]^+$ Calcd for $C_{35}H_{48}OP$ 515.3437; Found 515.3416

((1*RS*,2*RS*)-2-((*E*)-14-(1-Trityl-1*H*-imidazol-4-yl)tetradec-13-en-1-yl)cyclopropyl)-methanol (45)

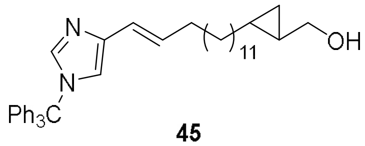

45

45 was prepared following general procedure 7:
44 (2.72 g, 4.23 mmol, 1.0 eq.) in dry THF (100 mL).
Phenyllithium solution (5.10 mL, 9.18 mmol, 2.2 eq., 1.8 M in Bu_2O).
Aldehyde **8** (1.55 g, 4.58 mmol, 1.1 eq.) in dry THF (40 mL).
Phenyllithium solution (5.10 mL, 9.18 mmol, 2.2 eq., 1.8 M in Bu_2O).
Flash chromatography [silica gel, CH_2Cl_2, then CH_2Cl_2/MeOH (100:1) to (50:1)].
45 (2.01 g, 3.50 mmol, 83%) as yellow oil.

TLC [silica gel, CH_2Cl_2/MeOH (50:1)]: R_f = 0.25.

^{1}H-NMR (400 MHz, $CDCl_3$): δ = 7.37 (d, ^4J = 1.2 Hz, 1H, NC*H*N), 7.34-7.29 (m, 9H, *m*-/*p*-Ph-C*H*), 7.17-7.11 (m, 6H, *o*-Ph-C*H*), 6.66 (d, ^4J = 1.3 Hz, 1H, NCC*H*N), 6.34 (td, ^3J = 6.8 Hz, 15.7 Hz, 1H, NCCHC*H*), 6.20 (d, ^3J = 15.8 Hz, 1H, NCC*H*CH), 3.49-3.37 (m, 2H, C*H*$_2$OH), 2.18-2.10 (m, 2H, NCCHCHC*H*$_2$), 1.99 (s, br, 1H, O*H*), 1.48-1.17 (m, 22H, $(CH_2)_{11}$), 0.87-0.77 (m, 1H, C*H*CHCH_2OH), 0.63-0.53 (m, 1H, C*H*CH_2OH), 0.38-0.25 (m, 2H, CHC*H*$_2$CH).

^{13}C-NMR (100 MHz, $CDCl_3$): δ = 142.4 (3C, *ipso*-Ph-*C*), 139.5 (N*C*CHN), 138.9 (N*C*HN), 129.8 (NCCH*C*H), 129.7 (6C, *o*-Ph-*C*H), 128.0 (9C, *m*-/*p*-Ph-*C*H), 121.3 (NC*C*HCH), 118.0 (NC*C*HN), 75.2 (*C*Ph_3), 67.0 (*C*H_2OH), 33.6 (*C*H_2CHCHCH_2OH), 32.8 (NCCHCH*C*H_2), 29.6 (3C, *C*H_2), 29.5 (4C, *C*H_2), 29.4 (*C*H_2), 29.3 (*C*H_2), 29.2 (*C*H_2), 21.2 (*C*HCHCH_2OH), 17.1 (*C*HCH_2OH), 9.9 (CH*C*H_2CH).

IR (ATR): $\tilde{\nu}$ = 327 (br), 3057 (w), 2989 (w), 232 (m), 290 (m), 163 (w), 156 (w), 1490 (m), 1468 (m), 1444 (m), 125 (w), 1224 (w), 1184 (w), 1157 (w), 1128 (m), 1080 (w), 1034 (m), 79 (m), 904 (w), 869 (w), 826 (w), 751 (s), 700 (s), 656 (m), 637 (m), 546 (w).

UV/VIS (MeOH): λ_{max} (log ε) = 235 (4.27), 203 (4.79).

MS (ESI+): *m/z* (%) = 1149.8 (31) $[2M+H]^+$, 575.4 (100) $[M+H]^+$, 243.1 (100) $[CPh_3]^+$.

HRMS (ESI) *m/z*: $[M + H]^+$ Calcd for $C_{40}H_{51}N_2O$ 575.4001; Found 575.4000

(1*RS*,2*RS*)-2-((*E*)-14-(1-Trityl-1*H*-imidazol-4-yl)tetradec-13-en-1-yl)cyclopropane-1-carbaldehyde

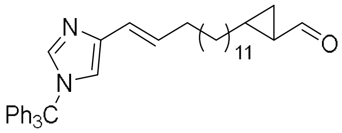

(1*RS*,2*RS*)-2-((*E*)-14-(1-Trityl-1*H*-imidazol-4-yl)tetradec-13-en-1-yl)cyclopropane-1-carbaldehyde was prepared following general procedure 2:

45 (316 mg, 0.55 mmol, 1.0 eq.) in dry CH_2Cl_2 (20 mL).
Triethylamine (0.77 mL, 5.50 mmol, 10 eq.).
Py·SO_3 (262 mg, 1.65 mmol, 3.0 eq.) in dry DMSO (5 mL).
Flash chromatography [Silica gel, CH_2Cl_2/MeOH (200:1)].
(1*RS*,2*RS*)-2-((*E*)-14-(1-Trityl-1*H*-imidazol-4-yl)tetradec-13-en-1-yl)cyclopropane-1-carbaldehyde (217 mg, 0.38 mmol, 69%) as yellowish oil.

TLC [silica gel, CH_2Cl_2/MeOH (100:1)]: R_f = 0.27.

^{1}H-NMR (400 MHz, $CDCl_3$): δ = 8.99 (d, ^3J = 5.7 Hz, 1H, C*H*O), 7.37 (s, 1H, NC*H*N), 7.35-7.30 (m, 9H, *m*-/*p*-Ph-C*H*), 7.18-7.12 (m, 6H, *o*-Ph-C*H*), 6.67 (s, 1H, NCC*H*N), 6.35 (td, ^3J = 6.8 Hz, 15.6 Hz, 1H, NCCHC*H*), 6.21 (d, ^3J = 15.7 Hz, 1H, NCC*H*CH), 2.18-2.11 (m, 2H, NCCHCHCH_2), 1.65-1.58 (m, 1H, C*H*CHO), 1.51-1.20 (m, 24H, $(CH_2)_{11}$, C*H*CHCHO, CHC[*H*]$_2$CH), 0.6-0.89 (m, 1H, CHC[*H*]$_2$CH).

^{13}C-NMR (100 MHz, $CDCl_3$): δ = 201.1 (*C*HO), 142.4 (3C, *ipso*-Ph-*C*), 139.5 (N*C*CHN), 138.9 (N*C*HN), 129.8 (7C, *o*-Ph-*C*H, NCCH*C*H), 128.0 (9C, *m*-/*p*-Ph-*C*H), 121.3 (NC*C*HCH), 118.0 (NC*C*HN), 75.2 (*C*Ph_3), 32.8 (NCCHCH*C*H_2), 32.6 (*C*H_2CHCHCHO), 30.5 (*C*HCHO), 29.6 (3C, *C*H_2), 29.5 (3C, *C*H_2), 29.3 (2C, *C*H_2), 29.2 (*C*H_2), 29.1 (*C*H_2), 22.7 (*C*HCHCHO), 14.9 (CH*C*H_2CH).

(1*RS*,2*RS*)-2-((*E*)-14-(1-Trityl-1*H*-imidazol-4-yl)tetradec-13-en-1-yl)cyclopropane-1-carboxylic acid (46)

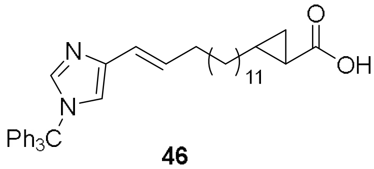

46

46 was prepared following general procedure 8:
(1*RS*,2*RS*)-2-((*E*)-14-(1-Trityl-1*H*-imidazol-4-yl)tetradec-13-en-1-yl)cyclopropane-1-carbaldehyde (183 mg, 0.32 mmol, 1.0 eq.) in *tert*-BuOH (18 mL) and 2-methyl-2-butene 6 mL).
$NaClO_2$ (203 mg, 2.24 mmol, 7.0 eq.), NaH_2PO_4 (397 mg, 2.88 mmol, 9.0 eq.) in water (5 mL).
Flash chromatography [silica gel, CH_2Cl_2, then CH_2Cl_2/MeOH (40:1)].
46 (170 mg, 0.29 mmol, 90%) as colorless oil.

TLC [silica gel, $CHCl_3$/MeOH/NH_3 (70:10:1)]: R_f = 0.16.

^{1}H-NMR (400 MHz, $CDCl_3$): δ = 8.07 (s, br, 1H, CO_2*H*), 7.48 (s, 1H, NC*H*N), 7.35-7.28 (m, 9H, *m*-/*p*-Ph-C*H*), 7.18-7.10 (m, 6H, *o*-Ph-C*H*), 6.65 (s, 1H, NCC*H*N), 6.32 (td, ^3J = 6.8 Hz, 15.5 Hz, 1H, NCCHC*H*), 6.19 (d, ^3J = 15.9 Hz, 1H, NCC*H*CH), 2.18-2.10 (m, 2H, NCCHCHCH_2), 1.48-1.12 (m, 25H, $(CH_2)_{11}$, C*H*C[*H*]$_2$C*H*), 0.72-0.64 (m, 1H, CHC[*H*]$_2$CH).

^{13}C-NMR (100 MHz, $CDCl_3$): δ = 179.0 (*C*O_2H), 142.2 (3C, *ipso*-Ph-*C*), 139.0 (N*C*CHN), 138.8 (N*C*HN), 130.6 (NCCH*C*H), 129.7 (6C, *o*-Ph-*C*H), 128.1 (9C, *m-/p*-Ph-*C*H), 120.7 (NC*C*HCH), 118.0 (NC*C*HN), 75.4 (*C*Ph_3), 33.0 (*C*H_2CHCHCO_2H), 32.9 (NCCHCH*C*H_2), 29.4 (4C, *C*H_2), 29.3 (2C, *C*H_2), 29.2 (*C*H_2), 29.1 (2C, *C*H_2), 29.0 (*C*H_2), 23.3 (*C*HCHCO_2H), 20.3 (*C*HCO_2H), 15.8 (CH*C*H_2CH).

IR (ATR): $\tilde{\nu}$ = 232 (m), 292 (w), 1717 (w), 165 (w), 144 (w), 1446 (w), 1159 (w), 1129 (w), 1084 (w), 1036 (w), 869 (w), 747 (m), 700 (s), 659 (m), 638 (m).

UV/VIS (MeOH): λ_{max} (log ε) = 254 (3.5), 204 (4.64).

MS (ESI+): *m/z* (%) = 589.4 (35) $[M+H]^+$, 243.1 (100) $[CPh_3]^+$.

HRMS (ESI) *m/z*: $[M + H]^+$ Calcd for $C_{40}H_{49}N_2O_2$ 589.3789; Found 589.3791

(1*RS*,2*RS*)-2-((*E*)-14-(1*H*-Imidazol-4-yl)tetradec-13-en-1-yl)cyclopropane-1-carboxylic acid (imidacin A2, 47)

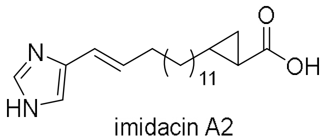

imidacin A2
47

rac-imidacin A2 (**47**) was prepared following general procedure 9:
46 (162 mg, 0.28 mmol, 1.0 eq.)
Acetic acid (2.00 mL) in methanol (20 mL)
Flash chromatography [silica gel, $CHCl_3$/MeOH/NH_3 (40:10:1)]
rac-imidacin A2 (53 mg, 0.15 mmol, 56%) as colorless solid.

TLC [silica gel, $CHCl_3$/MeOH/NH_3 (40:10:1)]: R_f = 0.26.

^{1}H-NMR (600 MHz, CD_3OD + 4 µL NH_3): δ = 7.56 (s, 1H, NC*H*N), 6.90 (s, 1H, NCC*H*N), 6.26 (d, ^3J = 15.9 Hz, 1H, NCC*H*CH), 6.13 (td, ^3J = 6.9 Hz, 15.9 Hz, 1H, NCCHC*H*), 2.20-2.14 (m, 2H, NCCHCHC*H*$_2$), 1.49-1.14 (m, 24H, (C*H*$_2$)$_{11}$, C*H*C*H*CO_2H), 0.97-0.4 (m, 1H, CHC[*H*]$_2$CH), 0.46-0.41 (m, 1H, CHC[*H*]$_2$CH).

^{13}C-NMR (150 MHz, CD_3OD + 4 µL NH_3): δ = 183.6 (*C*O_2H), 136.5 (N*C*HN), 130.3 (NCCH*C*H), 120.6 (NC*C*HCH), 34.7 (*C*H_2CHCHCO_2H), 34.0 (NCCHCH*C*H_2), 30.8 (4C, *C*H_2), 30.7 (*C*H_2), 30.6 (4C, *C*H_2), 30.3 (*C*H_2), 24.4 (*C*HCHCO_2H), 22.1 (*C*HCO_2H), 14.9 (CH*C*H_2CH).

IR (ATR): $\tilde{\nu}$ = 3146 (w), 3010 (w), 2916 (s), 2848 (s), 2615 (w), 1668 (m), 1466 (w), 1407 (w), 1352 (w), 1176 (w), 1103 (m), 1081 (w), 79 (m), 811 (m), 747 (m), 721 (m), 665 (w), 634 (m).

UV/VIS (MeOH): λ_{max} (log ε) = 250 (4.12).

MS (ESI+): *m/z* (%) = 64.5 (12) $[2M+H]^+$, 391.2 (9) $[M-H+2Na]^+$, 369.3 (42) $[M+Na]^+$, 347.3 (100) $[M+H]^+$, 329.3 (27) $[M-OH]^+$, 301.3 (13) $[M-CO_2H]^+$.

HRMS (ESI) *m/z*: $[M + H]^+$ Calcd for $C_{21}H_{35}N_2O_2$ 347.2640; Found 347.2661

5.8 Synthesis of imidacin B1

(11-Hydroxyundecyl)triphenylphosphonium bromide (15)

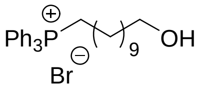

15

Alcohol **14** (7.54 g, 30.0 mmol, 1.0 eq.), obtained from dodecane-1,12-diol according to procedure 1, and triphenylphosphane (9.45 g, 36.0 mmol, 1.2 eq.) were dissolved in CH_3CN (120 mL) and heated at reflux for 19.5 h. The solvent was removed under reduced pressure and the residue purified by flash chromatography (silica gel, EtOAc, then CH_2Cl_2/MeOH 20:1 to 10:1). Phosphonium salt **15** (13.1 g, 25.5 mmol, 85%) was obtained as colorless solid. [S14]

TLC [silica gel, CH_2Cl_2/MeOH (20:1)]: $R_f = 0.22$.

^{1}H-NMR (400 MHz, $CDCl_3$): δ = 7.87-7.78 (m, 9H, *m*-/*p*-Ph-C*H*), 7.76-7.69 (m, 6H, *o*-Ph-C*H*), 3.72-3.62 (m, 2H, PC*H*$_2$), 3.60 (t, ^3J = 6.7 Hz, 2H, C*H*$_2$OH), 2.36 (s, br, 1H, O*H*), 1.69-1.57 (m, 4H, $PCH_2CH_2CH_2$), 1.57-1.48 (m, 2H, C*H*$_2$CH$_2$OH), 1.35-1.13 (m, 12H, (C*H*$_2$)$_6$).

^{13}C-NMR (100 MHz, $CDCl_3$): δ = 134.9 (3C, d, J = 2.9 Hz, *p*-Ph-*C*H), 133.5 (6C, d, J = 9.9 Hz, *o*-Ph-*C*H), 130.4 (6C, d, J = 12.5 Hz, *m*-Ph-*C*H), 118.1 (3C, d, J = 9.8 Hz, *ipso*-Ph-*C*), 62.5 (*C*H$_2$OH), 32.5 (*C*H$_2$CH$_2$OH), 30.2 (d, J = 15.5 Hz, PCH$_2$CH$_2$*C*H$_2$), 29.2 (*C*H$_2$), 29.1 (2C, *C*H$_2$), 28.9 (2C, *C*H$_2$), 25.5 (*C*H$_2$CH$_2$CH$_2$OH), 22.6 (d, J = 49.8 Hz, P*C*H$_2$), 22.4 (d, J = 4.5 Hz, PCH$_2$*C*H$_2$).

IR (ATR): $\tilde{\nu}$ = 3268 (m), 3046 (w), 3015 (w), 232 (m), 291 (m), 1586 (w), 1482 (w), 1466 (w), 1436 (m), 1317 (w), 1182 (w), 1111 (m), 1059 (m), 6 (m), 44 (w), 910 (w), 748 (s), 725 (s), 690 (s), 628 (m), 531 (s).

UV/VIS (MeOH): λ_{max} (log ε) = 274 (3.36), 267 (3.44), 225 (4.36), 204 (4.59).

MS (ESI+): *m/z* (%) = 433.27 (100) $[M-Br]^+$.

HRMS (ESI) *m/z*: $[M - Br]^+$ Calcd for $C_{29}H_{38}OP$ 433.2655; Found 433.2659

(*E*)-12-(1-Trityl-1*H*-imidazol-4-yl)dodec-11-en-1-ol (16)

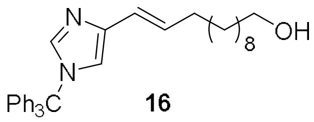

16

Alcohol **16** was prepared following general procedure 7:
Phosphonium salt **15** (5.14 g, 10.0 mmol, 1.1 eq.) in dry THF (100 mL).
Phenyllithium solution (11.2 mL, 20.0 mmol, 2.2 eq., 1.8 M in Bu_2O).
Aldehyde **52** (3.05 g, 9.01 mmol, 1.0 eq.) in dry THF (50 mL).
Phenyllithium solution (11.2 mL, 20.0 mmol, 2.2 eq., 1.8 M in Bu_2O).
Flash chromatography [silica gel, CH_2Cl_2/MeOH (100:1)].
Alcohol **16** (2.4 g, 5.6 mmol, 66%) as yellow solid.

TLC [silica gel, CH_2Cl_2/MeOH (50:1)]: $R_f = 0.20$.

^{1}H-NMR (400 MHz, $CDCl_3$): δ = 7.37 (d, ^4J = 1.3 Hz, 1H, NC*H*N), 7.35-7.30 (m, 9H, *m*-/*p*-Ph-C*H*), 7.17-7.12 (m, 6H, *o*-Ph-C*H*), 6.66 (d, ^4J = 1.3 Hz, 1H, NCC*H*N), 6.34 (td, ^3J = 6.8 Hz, 15.7 Hz, 1H, NCCHC*H*), 6.20 (d, ^3J = 15.7 Hz, 1H, NCC*H*CH), 3.62 (t, ^3J = 6.7 Hz, 2H, C*H*$_2$OH), 2.18-2.11 (m, 2H, NCCHCHC*H*$_2$), 1.59-1.51 (m, 2H, C*H*$_2$CH$_2$OH), 1.47-1.38 (m, 2H, CHCH$_2$C*H*$_2$), 1.37-1.24 (m, 12H, (C*H*$_2$)$_6$).

^{13}**C-NMR** (100 MHz, $CDCl_3$): δ = 142.4 (3C, *ipso*-Ph-*C*), 139.5 (N*C*CHN), 138.9 (N*C*HN), 129.8 (NCCHCH), 129.7 (6C, *o*-Ph-*C*H), 128.0 (9C, *m*-/*p*-Ph-*C*H), 121.3 (NC*C*HCH), 118.0 (NC*C*HN), 75.2 (CPh_3), 62.8 (CH_2OH), 32.8 ($NCCHCHCH_2$), 29.5 (CH_2), 29.4 (2C, CH_2), 29.3 (2C, CH_2), 29.1 (CH_2), 25.7 (CH_2).

IR (ATR): $\tilde{\nu}$ = 3338 (br), 3060 (w), 231 (m), 291 (m), 1735 (w), 156 (w), 1490 (m), 1469 (m), 1444 (m), 1221 (w), 1183 (w), 1158 (w), 1128 (m), 1083 (w), 1036 (m), 1000 (w), 971 (m), 905 (w), 869 (w), 824 (w), 749 (s), 700 (s), 655 (m), 637 (m).

UV/VIS (MeOH): λ_{max} (log ε) = 241 (4.28), 203 (4.79).

MS (ESI+): *m/z* (%) = 1500.9 (31) $[3M+Na]^+$, 1007.6 (89) $[2M+Na]^+$, 515.3 (60) $[M+Na]^+$, 243.1 (100) $[CPh_3]^+$.

HRMS (ESI) *m/z*: $[M + Na]^+$ Calcd for $C_{34}H_{40}N_2NaO$ 515.3033; Found 515.3033

(*E*)-12-(1-Trityl-1*H*-imidazol-4-yl)dodec-11-enal (17)

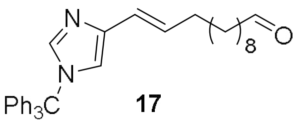

17

A solution of dry DMSO (0.37 mL, 5.21 mmol, 3.0 eq.) in dry CH_2Cl_2 (5 mL) was added at −78 °C to a solution of oxalyl chloride (0.22 mL, 2.56 mmol, 1.5 eq.) in dry CH_2Cl_2 (5 mL). After stirring at −78 °C for 20 min, a solution of **16** (94 mg, 1.73 mmol, 1.0 eq.) in dry CH_2Cl_2 (20 mL) was slowly added and stirred further for 30 min at −78 °C. Triethylamine (1.81 mL, 13.0 mmol, 7.5 eq.) was added and after 5 min the reaction mixture was allowed to warm to room temperature. After quenching with 10% NH_4Cl solution (20 mL), layers were separated and the aqueous layer was extracted with CH_2Cl_2 (3 x 20 mL). The combined organic phases were washed with sat. NaCl solution (40 mL) and water (40 mL) and dried with $MgSO_4$. The solvent was removed under reduced pressure and the residue was purified by flash chromatography [silica gel, CH_2Cl_2/MeOH (200:1)]. Aldehyde **17** (506 mg, 1.03 mmol, 59%) was obtained as colorless solid. [S9]

TLC [silica gel, CH_2Cl_2/MeOH (100:1)]: R_f = 0.23.

^{1}**H-NMR** (400 MHz, $CDCl_3$): δ = 9.76 (t, ^3J = 1.9 Hz, 1H, C*H*O), 7.37 (d, ^4J = 1.0 Hz, 1H, NC*H*N), 7.35-7.30 (m, 9H, *m*-/*p*-Ph-C*H*), 7.17-7.12 (m, 6H, *o*-Ph-C*H*), 6.67 (d, ^4J = 1.1 Hz, 1H, NCC*H*N), 6.34 (td, ^3J = 6.8 Hz, 15.6 Hz, 1H, NCCHC*H*), 6.20 (d, ^3J = 15.8 Hz, 1H, NCC*H*CH), 2.41 (dt, ^3J = 1.9 Hz, 7.4 Hz, 2H, CH_2CHO), 2.18-2.11 (m, 2H, $NCCHCHCH_2$), 1.67-1.57 (m, 2H, CH_2CH_2CHO), 1.48-1.23 (m, 12H, $(CH_2)_6$).

^{13}**C-NMR** (100 MHz, $CDCl_3$): δ = 203.0 (*C*HO), 142.4 (3C, *ipso*-Ph-*C*), 139.5 (N*C*CH), 138.9 (N*C*HN), 129.8 (7C, *o*-Ph-*C*H, NCCH*C*H), 128.0 (9C, *m*-/*p*-Ph-*C*H), 121.3 (NC*C*HCH), 118.0 (NC*C*HN), 75.2 (CPh_3), 43.9 (CH_2CHO), 32.8 ($NCCHCHCH_2$), 29.4 (CH_2), 29.3 (3C, CH_2), 29.2 (CH_2), 29.1 (CH_2), 22.1 (CH_2CH_2CHO).

IR (ATR): $\tilde{\nu}$ = 3178 (br), 3058 (w), 231 (m), 291 (m), 1724 (w), 1491 (m), 1469 (m), 1444 (m), 1220 (w), 1124 (m), 1075 (m), 1036 (m), 78 (m), 46 (m), 869 (m), 827 (m), 748 (s), 699 (s), 655 (m), 638 (m), 574 (w).

UV/VIS (MeOH): λ_{max} (log ε) = 241 (4.25), 204 (4.71).

MS (EI): *m/z* (%) = 490.3 (<5) $[M^+]$, 243.1 (100) $[CPh_3]^+$, 165.1 (22).

HREIMS (ESI) *m/z*: $[M]^+$ Calcd for $C_{34}H_{38}N_2O$ 490.2979; Found 490.2971

(3-Hydroxypropyl)triphenylphosphonium bromide

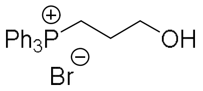

3-Bromopropan-1-ol (4.17 g, 30.0 mmol, 1.0 eq.) and triphenylphosphane (9.45 g, 36.0 mmol, 1.2 eq.) were dissolved in CH_3CN (120 mL) and heated at reflux for 23.5 h. The solvent was removed under reduced pressure and the residue was purified by flash chromatography [silica gel, CH_2Cl_2/MeOH (10:1)]. The phosphonium salt (8.57 g, 21.4 mmol, 71%) was obtained as colorless solid. [S14]

TLC [silica gel, CH_2Cl_2/MeOH (10:1)]: R_f = 0.30.

^{1}H-NMR (400 MHz, $CDCl_3$): δ = 7.84-7.75 (m, 9H, *m-/p*-Ph-C*H*), 7.74-7.67 (m, 6H, *o*-Ph-C*H*), 3.89-3.74 (m, 4H, CH_2OH, PCH_2), 1.91-1.80 (m, 2H, CH_2CH$_2$OH).

^{13}C-NMR (100 MHz, $CDCl_3$): δ = 135.0 (3C, d, J = 3.0 Hz, *p*-Ph-*C*H), 133.5 (6C, d, J = 9.9 Hz, *o*-Ph-*C*H), 130.5 (6C, d, J = 12.5 Hz, *m*-Ph-*C*H), 118.4 (3C, d, J = 86.1 Hz, *ipso*-Ph-*C*), 60.3 (d, J = 16.6 Hz, *C*H$_2$OH), 25.9 (d, J = 4.4 Hz, *C*H$_2$CH$_2$OH), 20.2 (d, J = 52.5 Hz, P*C*H$_2$).

IR (ATR): $\tilde{\nu}$ = 3318 (br), 3077 (w), 3009 (w), 253 (w), 2889 (w), 2862 (w), 2791 (w), 1586 (w), 149 (w), 1435 (m), 1387 (w), 1319 (w), 1211 (w), 1161 (w), 1111 (s), 1048 (m), 1025 (m), 97 (m), 899 (m), 877 (m), 790 (w), 741 (s), 721 (s), 691 (s), 622 (m), 598 (m), 532 (s).

UV/VIS (MeOH): λ_{max} (log ε) = 274 (3.44), 267 (3.52), 225 (4.41), 204 (4.61).

MS (ESI+): *m/z* (%) = 321.1 (100) $[M-Br]^+$.

HRMS (ESI) *m/z*: $[M - Br]^+$ Calcd for $C_{21}H_{22}OP$ 321.1403; Found 321.1400

3-((1-Phenyl-1*H*-tetrazol-5-yl)thio)propan-1-ol

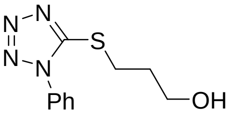

Potassium carbonate (K_2CO_3, 1.98 g, 23.6 mmol, 2.4 eq.) was added to a solution of 1-phenyl-1*H*-tetrazole-5-thiol (1.78 g, 9.99 mmol, 1.0 eq.) and 3-bromopropan-1-ol (1.39 g, 10.0 mmol, 1.0 eq.) in acetone (20 mL) at r.t. and the resulting mixture was heated to 60 °C for 3.5 h. After addition of water (100 mL) and CH_2Cl_2 (20 mL), the layers were separated and the aqueous layer was extracted with CH_2Cl_2 (3 x 30 mL). The combined organic phases were washed with sat. NaCl solution (50 mL) and water (50 mL) and dried with $MgSO_4$. The solvent was removed under reduced pressure and the title alcohol (2.30 g, 9.74 mmol, 98%) was obtained as colorless solid.[S21]

TLC [silica gel, *n*-pentane/EtOAc (1:1)]: R_f = 0.30.

^{1}H-NMR (400 MHz, $CDCl_3$): δ = 7.61-7.52 (m, 5H, Ph-C*H*), 3.82-3.75 (m, 2H, CH_2OH), 3.54 (t, ^3J = 6.7 Hz, 2H, SCH_2), 3.08 (s, br, 1H, O*H*), 2.12-2.05 (m, 2H,CH_2CH$_2$OH).

^{13}C-NMR (100 MHz, $CDCl_3$): δ = 155.0 (N*C*S), 133.5 (*ipso*-Ph-*C*H), 130.2 (*p*-Ph-*C*H), 129.8 (2C, *m*-Ph-*C*H), 123.8 (2C, *o*-Ph-*C*H), 59.6 (*C*H$_2$OH), 32.5 (*C*H$_2$CH$_2$OH), 29.9 (S*C*H$_2$).

IR (ATR): $\tilde{\nu}$ = 3386 (m), 242 (m), 2847 (w), 154 (m), 144 (m), 1415 (m), 1390 (s), 1353 (w), 1317 (m), 1271 (m), 1248 (m), 1211 (w), 1172 (w), 103 (m), 1071 (s), 1011 (m), 980 (m), 908 (m), 758 (s), 735 (m), 69 (s), 633 (m), 552 (m).

UV/VIS (MeOH): λ_{max} (log ε) = 226 (3.87), 203 (4.25).

MS (ESI+): *m/z* (%) = 46.1 (63) $[2M+Na]^+$, 259.1 (100) $[M+Na]^+$, 237.1 (27) $[M+H]^+$.

HRMS (ESI) *m/z*: $[M + Na]^+$ Calcd for $C_{10}H_{12}N_4NaOS$ 259.0624; Found 259.0621

3-((1-Phenyl-1*H*-tetrazol-5-yl)sulfonyl)propan-1-ol

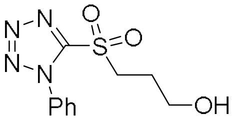

An ice cold solution of ammonium heptamolybdate (1.90 g, 1.54 mmol, 0.16 eq.) in H_2O_2 (6 mL, 30% in H_2O) was slowly added to a solution of alcohol 3-((1-phenyl-1*H*-tetrazol-5-yl)thio)propan-1-ol (2.27 g, 9.61 mmol, 1.0 eq.) in EtOH (25 mL) and THF (20 mL) at 5 °C. Three more batches with identical composition were added in intervals of 1 h. The mixture was stirred for 1 h at r.t., water (120 mL) was added and the phases were separated. The aqueous layer was extracted with CH_2Cl_2 (4 x 40 mL) and the combined organic phases were washed with sat. NaCl solution (40 mL) and water (40 mL). After drying with MgSO4, the solvent was removed under reduced pressure and the residue was purified by flash chromatography [silica gel, *n*-pentane/EtOAc (1:1)]. The sulfone (2.51 g, 9.36 mmol, 97%) was obtained as colorless oil. [S21] The spectroscopic data matched those reported earlier. [S22]

TLC [silica gel, *n*-pentane/EtOAc (1:1)]: $R_f = 0.36$.

^{1}H-NMR (400 MHz, $CDCl_3$): δ = 7.72-7.57 (m, 5H, Ph-C*H*), 3.4-3.88 (m, 2H, SCH_2), 3.82 (t, $^3J = 5.9$ Hz, 2H, CH_2OH), 2.27-2.19 (m, 2H, CH_2CH$_2$OH), 1.5 (s, br, 1H, O*H*).

^{13}C-NMR (100 MHz, $CDCl_3$): δ = 153.5 (N*C*S), 133.0 (*ipso*-Ph-*C*H), 131.5 (*p*-Ph-*C*H), 129.7 (2C, *m*-Ph-*C*H), 125.1 (2C, *o*-Ph-*C*H), 59.9 (*C*H$_2$OH), 53.2 (S*C*H$_2$), 25.3 (*C*H$_2$CH$_2$OH).

5-((3-((*tert*-Butyldimethylsilyl)oxy)propyl)sulfonyl)-1-phenyl-1 *H*-tetrazole (18)

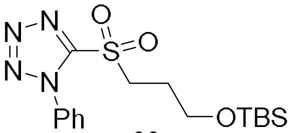

69

Imidazole (784 mg, 11.5 mmol, 3.0 eq.) and *tert.*-butyldimethylsilyl chloride (TBSCl, 754 mg, 5.00 mmol, 1.3 eq.) were added to a solution of 3-((1-phenyl-1*H*-tetrazol-5-yl)sulfonyl)propan-1-ol (1.03 g, 3.84 mmol, 1.0 eq.) in dry THF (40 mL) and the resulting mixture was stirred for 5 h at r.t. The solvent was removed under reduced pressure, the residue was taken up in water and extracted with CH_2Cl_2 (3 x 40 mL). The combined organic layers were washed with sat. NaCl solution (40 mL) and dried with $MgSO_4$. The solvent was removed under reduced pressure and the residue purified by flash chromatography [silica gel, *n*-pentane/EtOAc (5:1)]. Compound **18** (1.33 g, 3.48 mmol, 91%) was obtained as colorless solid. [S23]

TLC [silica gel, *n*-pentane/EtOAc (20:1)]: $R_f = 0.34$.

^{1}H-NMR (400 MHz, $CDCl_3$): δ = 7.72-7.57 (m, 5H, Ph-C*H*), 3.87-3.82 (m, 2H, SCH_2), 3.78 (t, $^3J = 5.7$ Hz, 2H, CH_2OSi), 2.21-2.12 (m, 2H, CH_2CH$_2$OSi), 0.90 (s, 9H, C(CH_3)$_3$), 0.07 (s, 6H, Si(CH_3)$_2$).

^{13}C-NMR (100 MHz, $CDCl_3$): δ = 153.5 (N*C*S), 133.1 (*ipso*-Ph-*C*H), 131.4 (*p*-Ph-*C*H), 129.7 (2C, *m*-Ph-*C*H), 125.1 (2C, *o*-Ph-*C*H), 60.3 (*C*H$_2$OSi), 53.4 (S*C*H$_2$), 25.8 (3C, C(*C*H$_3$)$_3$), 25.5 (*C*H$_2$CH$_2$OSi), 18.2 (*C*(CH$_3$)$_3$), −5.4 (2C, Si(*C*H$_3$)$_2$).

MS (ESI+): *m/z* (%) = 787.3 (100) $[2M+Na]^+$, 405.1 (57) $[M+Na]^+$, 383.2 (83) $[M+H]^+$.

HRMS (ESI) *m/z*: $[M + H]^+$ Calcd for $C_{16}H_{27}N_4O_3SSi$ 383.1568; Found 383.1567

4-((1*E*,12*E*)-15-((*tert*-Butyldimethylsilyl)oxy)pentadeca-1,12-dien-1-yl)-1-trityl-1*H*-imidazole

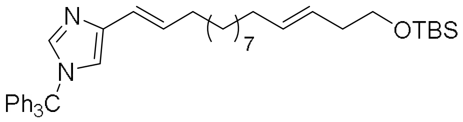

A KHMDS solution (0.48 mL, 0.24 mmol, 1.2 eq., 0.5 M in toluene) was slowly added to a solution of sulfone **18** (77 mg, 0.20 mmol, 1.0 eq.) and aldehyde **17** (108 mg, 0.22 mmol, 1.1 eq.) in dry THF (3.5 mL) at −78 °C. The mixture was stirred for 30 min at −78 °C, allowed to warm to r.t. over 1.5 h and further stirred for 40 min at r.t. Sat. NH_4Cl solution (5 mL) was added and the layers were separated. The aqueous layer was extracted with Et_2O (3 x 15 mL) and EtOAc (1 x 15 mL), the combined organic phases were washed with sat. NaCl solution (20 mL) and dried with $MgSO_4$. The solvent was removed under reduced pressure and the residue was purified by flash chromatography [silica gel, *n*-pentane/EtOAc (10:1)]. 4-((1*E*,12*E*)-15-((*tert*-butyldimethylsilyl)oxy)pentadeca-1,12-dien-1-yl)-1-trityl-1*H*-imidazole (65 mg, 0.10 mmol, 50%) was obtained as colorless oil.[S24]

TLC [silica gel, *n*-pentane/EtOAc (10:1)]: R_f = 0.43.

^{1}H-NMR (300 MHz, $CDCl_3$): δ = 7.37 (d, ^4J = 1.4 Hz, 1H, NC*H*N), 7.35-7.29 (m, 9H, *m*-/*p*-Ph-C*H*), 7.18-7.12 (m, 6H, *o*-Ph-C*H*), 6.66 (d, ^4J = 1.4 Hz, 1H, NCC*H*N), 6.35 (td, ^3J = 6.7 Hz, 15.7 Hz, 1H, NCCHC*H*), 6.20 (d, ^3J = 15.7 Hz, 1H, NCC*H*CH), 5.53-5.31 (m, 2H, C*H*C*H*(CH_2)$_2$OH), 3.61 (t, ^3J = 6.9 Hz, 2H, C*H*$_2$OSi), 2.26-2.09 (m, 4H, NCCHCHC*H*$_2$, C*H*$_2$CH$_2$OH), 2.03-1.91 (m, 2H, C*H*$_2$CHCH(CH_2)$_2$OH), 1.49-1.21 (m, 14H, (C*H*$_2$)$_7$), 0.89 (s, 9H, C(C*H*$_3$)$_3$), 0.05 (s, 6H, Si(C*H*$_3$)$_2$).

^{13}C-NMR (75 MHz, $CDCl_3$): δ = 142.4 (3C, *ipso*-Ph-*C*), 139.6 (N*C*CH), 138.9 (N*C*HN), 132.6 (*C*HCH(CH_2)$_2$OH), 129.8 (7C, *o*-Ph-*C*H, NCCH*C*H), 128.0 (9C, *m*-/*p*-Ph-*C*H), 126.2 (*C*H(CH_2)$_2$OH), 121.3 (NC*C*HCH), 118.0 (NC*C*HN), 75.2 (*C*Ph$_3$), 63.4 (*C*H$_2$OSi), 36.3 (*C*H$_2$CH$_2$OH), 32.8 (NCCHCH*C*H$_2$), 32.7 (*C*H$_2$CHCH(CH_2)$_2$OH), 29.5 (4C, *C*H$_2$), 29.3 (2C, *C*H$_2$), 29.2 (*C*H$_2$), 25.9 (3C, C(*C*H$_3$)$_3$), 18.3 (*C*(CH$_3$)$_3$), −5.2 (2C, Si(*C*H$_3$)$_2$).

(3*E*,14*E*)-15-(1-Trityl-1*H*-imidazol-4-yl)pentadeca-3,14-dien-1-ol (19)

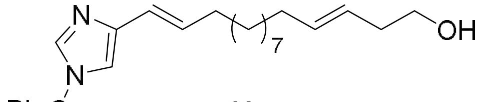

19

Method A: Wittig-Schlosser reaction

Alcohol **19** was prepared following general procedure 7:
(3-Hydroxypropyl)triphenylphosphonium bromide (161 mg, 0.40 mmol, 1.0 eq.) in dry THF (10 mL)
Phenyllithium solution (0.45 mL, 0.81 mmol, 2.0 eq., 1.8 M in Bu_2O)
Aldehyde **17** (197 mg, 0.40 mmol, 1.0 eq.) in dry THF (10 mL)
Phenyllithium solution (0.45 mL, 0.81 mmol, 2.0 eq., 1.8 M in Bu_2O)
Flash chromatography [silica gel, *n*-pentane/EtOAc (3:1) to (1:1)]
Alcohol **19** (50 mg, 0.09 mmol, 23%) as yellowish oil

Method B: TBAF deprotection of 4-((1*E*,12*E*)-15-((*tert*-butyldimethylsilyl)oxy)pentadeca-1,12-dien-1-yl)-1-trityl-1*H*-imidazole

To a solution of 4-((1*E*,12*E*)-15-((*tert*-butyldimethylsilyl)oxy)pentadeca-1,12-dien-1-yl)-1-trityl-1*H*-imidazole (63 mg, 1.47 mmol, 1.0 eq.) in dry THF (50 mL) at 0 °C was added TBAF solution (1.80 mL, 1.80 mmol, 1.2 eq., 1.0 M in THF) and the resulting mixture was stirred for 3.5 h at r.t. After addition of sat. NH_4Cl solution (40 mL), the layers were separated and the aqueous layer was extracted with CH_2Cl_2 (4 x 40 mL). The combined organic layers were washed with sat. NaCl solution (50 mL), dried with $MgSO_4$ and the solvent was removed under reduced pressure. The residue was purified by flash chromatography [silica gel, $CHCl_3$/MeOH (100:1)] yielding **19** (584 mg, 1.10 mmol, 74%) as yellowish oil. [S25]

TLC [silica gel, CH_2Cl_2/MeOH (50:1)]: R_f = 0.25.

^{1}H-NMR (300 MHz, $CDCl_3$): δ = 7.36 (d, ^4J = 1.3 Hz, 1H, NC*H*N), 7.35-7.29 (m, 9H, *m*-/*p*-Ph-C*H*), 7.18-7.11 (m, 6H, *o*-Ph-C*H*), 6.66 (d, ^4J = 1.3 Hz, 1H, NCC*H*N), 6.34 (td, ^3J = 6.6 Hz, 15.7 Hz, 1H, NCCHC*H*), 6.20 (d, ^3J = 15.8 Hz, 1H, NCC*H*CH), 5.60-5.48 (m, 1H, C*H*CH(CH_2)$_2$OH), 5.43-5.30 (m, 1H, C*H*(CH_2)$_2$OH), 3.62 (t, ^3J = 6.3 Hz, 2H, C*H*$_2$OH), 2.30-2.20 (m, 2H, C*H*$_2$$CH_2$OH), 2.19-2.09 (m, 2H, NCCHCHC*H*$_2$), 2.05-1.82 (m, 3H, C*H*$_2$CHCH(CH_2)$_2$O*H*), 1.49-1.20 (m, 14H, (C*H*$_2$)$_7$).

^{13}C-NMR (75 MHz, $CDCl_3$): δ = 142.4 (3C, *ipso*-Ph-*C*), 139.5 (N*C*CH), 138.9 (N*C*HN), 134.2 (*C*HCH(CH_2)$_2$OH), 129.8 (7C, *o*-Ph-*C*H, NCCH*C*H), 128.0 (9C, *m*-/*p*-Ph-*C*H), 125.7 (*C*H(CH_2)$_2$OH), 121.3 (NC*C*HCH), 118.0 (NC*C*HN), 75.2 (*C*Ph_3), 62.0 (*C*H_2OH), 36.0 (*C*$H_2$$CH_2$OH), 32.8 (NCCHCH*C*$H_2$), 32.6 (*C*$H_2$CHCH($CH_2$)$_2$OH), 29.5 (2C, *C*$H_2$), 29.4 (2C, *C*$H_2$), 29.3 (*C*$H_2$), 29.2 (*C*$H_2$), 29.1 (*C*$H_2$).

IR (ATR): $\tilde{\nu}$ = 3300 (br), 3059 (w), 3031 (w), 231 (m), 291 (m), 156 (w), 1490 (m), 1469 (w), 1444 (m), 1223 (w), 1184 (w), 1157 (w), 1129 (m), 109 (w), 1051 (m), 1035 (m), 76 (m), 905 (w), 869 (w), 825 (w), 749 (s), 700 (s), 656 (m), 637 (m).

UV/VIS (MeOH): λ_{max} (log ε) = 241 (4.25), 203 (4.77).

MS (ESI+): *m/z* (%) = 1065.7 (18) $[2M+H]^+$, 555.3 (8) $[M+Na]^+$, 533.4 (80) $[M+H]^+$, 291.2 (25), 243.1 (100) $[CPh_3]^+$, 165.1 (37).

HRMS (ESI) *m/z*: $[M + H]^+$ Calcd for $C_{37}H_{45}N_2O$ 533.3526; Found 533.3528

11-Bromoundecanal (25)

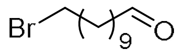

25

Aldehyde **25** was prepared following general procedure 2:
Alcohol **14** (5.03 g, 20.0 mmol, 1.0 eq.) in dry CH_2Cl_2 (40 mL).
Triethylamine (27.8 mL, 200 mmol, 10 eq.).
Py·SO_3 (9.55 g, 60.0 mmol, 3.0 eq.) in dry DMSO (40 mL).
Flash chromatography [Silica gel, *n*-pentane/EtOAc (20:1)].
25 (4.12 g, 16.5 mmol, 83%) as colorless oil.
The spectroscopic data matched those reported earlier. [S26]

TLC [silica gel, *n*-pentane/EtOAc (10:1)]: R_f = 0.71.

^{1}H-NMR (300 MHz, $CDCl_3$): δ = 9.77 (t, ^3J = 1.9 Hz, 1H, C*H*O), 3.41 (t, ^3J = 6.9 Hz, 2H, C*H*$_2$Br), 2.42 (dt, ^3J = 1.9 Hz, 7.3 Hz, 2H, C*H*$_2$CHO), 1.91-1.80 (m, 2H, C*H*$_2$$CH_2$Br), 1.69-1.56 (m, 2H, C*H*$_2$$CH_2$CHO), 1.49-1.24 (m, 12H, (C*H*$_2$)$_6$).

^{13}**C-NMR** (75 MHz, $CDCl_3$): δ = 202.9 (*C*HO), 43.9 (*C*H_2CHO), 34.0 (*C*H_2Br), 32.8 (*C*H_2CH_2Br), 29.3 (3C, *C*H_2), 29.1 (*C*H_2), 28.7 (*C*H_2), 28.1 (*C*H_2), 22.0 (*C*H_2CH_2CHO).

(*E*)-((14-Bromotetradec-3-en-1-yl)oxy)(*tert*-butyl)dimethylsilane

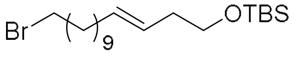

Sulfone **18** (4.25 g, 11.1 mmol, 1.0 eq.) and aldehyde **25** (3.87 g, 15.5 mmol, 1.4 eq.) were placed in dry THF (120 mL) and cooled down to −78 °C. A solution of KHMDS (13.3 mL, 13.3 mmol, 1.2 eq., 1 M in THF) was slowly added over a period of 10 min and the reaction mixture was allowed to warm to r.t. overnight (16 h). The reaction was quenched by addition of sat. NH_4Cl solution (100 mL). The layers were separated and the aqueous layer was extracted with *n*-pentane (3 x 50 mL). The combined organic layers were washed with sat. NaCl solution (100 mL), dried with $MgSO_4$ and the solvent was removed in vacuo. The residue was purified by flash chromatography [silica gel, *n*-pentane/EtOAc (1:0) to (100:1)], c (4.14 g, 10.2 mmol, 92%) obtained as yellowish oil. [15]

TLC [silica gel, *n*-pentane]: R_f = 0.36.

^{1}**H-NMR** (400 MHz, $CDCl_3$): δ = 5.52-5.32 (m, 2H, C*H*C*H*), 3.61 (t, ^3J = 6.9 Hz, 2H, C*H*$_2$OSi), 3.40 (t, ^3J = 6.9 Hz, 2H, C*H*$_2$Br), 2.24-2.16 (m, 2H, C*H*$_2$CH_2OSi), 2.01-1.93 (m, 2H, C*H*$_2$CHCH(CH_2)$_2$OSi), 1.90-1.81 (m, 2H, C*H*$_2$CH_2Br), 1.46-1.23 (m, 14H, (C*H*$_2$)$_7$), 0.89 (s, 9H, C(C*H*$_3$)$_3$), 0.05 (s, 6H, Si(C*H*$_3$)$_2$).

^{13}**C-NMR** (100 MHz, $CDCl_3$): δ = 132.6 (*C*HCH(CH_2)$_2$OSi), 126.3 (*C*H(CH_2)$_2$OSi), 63.4 (*C*H_2OSi), 36.3 (*C*H_2CH_2OSi), 34.0 (*C*H_2Br), 32.8 (*C*H_2CH_2Br), 32.7 (*C*H_2CHCH(CH_2)$_2$OSi), 29.5 (3C, *C*H_2), 29.4 (*C*H_2), 29.1 (*C*H_2), 28.8 (*C*H_2), 28.2 (*C*H_2), 26.0 (3C, C(*C*H_3)$_3$), 18.4 (*C*(CH_3)$_3$), −5.2 (2C, Si(*C*H_3)$_2$).

MS (EI): *m/z* (%) = 404/406 (missing) [M^+], 347/349 (<1) [M−^{t}Bu]$^+$, 267 (3), 193 (3), 167/169 (25/26), 151 (5), 139 (12), 137 (19), 123 (26), 115 (6), 111 (17), 109 (59), 101 (12), 97 (31), 95 (100), 89 (47), 83 (33), 81 (84), 75 (83), 73 (70), 69 (52), 67 (57), 59 (22), 57 (22), 55 (62).

(*E*)-14-Bromotetradec-3-en-1-ol (26)

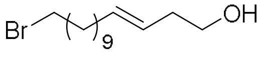

26

A solution of (*E*)-((14-bromotetradec-3-en-1-yl)oxy)(tert-butyl)dimethylsilane (5.00 g, 12.3 mmol, 1.0 eq.) in dry THF (80 mL) was treated at 0 °C with TBAF solution (14.8 mL, 14.8 mmol, 1.2 eq., 1.0 M in THF) and stirred 5 h at r.t. Sat. NH_4Cl-Lösung (80 mL) was added, the layers were separated and the aqueous layer was extracted with CH_2Cl_2 (3 x 50 mL). The combined organic layers were washed with sat. NaCl solution (100 mL), dried with $MgSO_4$ and the solvent was removed in vacuo. The residue was purified by flash chromatography [silica gel, *n*-pentane/EtOAc (10:1) bis (5:1)] and alcohol **26** (1.52 g, 5.21 mmol, 42%) was obtained as yellowish oil. [16]

TLC [silica gel, *n*-pentane/EtOAc (10:1)]: R_f = 0.31.

^{1}**H-NMR** (400 MHz, $CDCl_3$): δ = 5.60-5.51 (m, 1H, C*H*CH(CH_2)$_2$OH), 5.42-5.33 (m, 1H, C*H*(CH_2)$_2$OH), 3.62 (t, ^3J = 6.3 Hz, 2H, C*H*$_2$OH), 3.53 (t, ^3J = 6.8 Hz, 2H, C*H*$_2$Br), 2.29-2.22 (m, 2H, C*H*$_2$CH_2OH), 2.05-1.97 (m, 2H, C*H*$_2$CHCH(CH_2)$_2$OH), 1.81-1.54 (m, 3H, C*H*$_2$CH_2Br, O*H*), 1.47-1.22 (m, 14H, (C*H*$_2$)$_7$).

^{13}**C-NMR** (100 MHz, $CDCl_3$): δ = 134.3 (*C*HCH(CH_2)$_2$OH), 125.7 (*C*H(CH_2)$_2$OH), 62.0 (*C*H_2OH), 45.1 (*C*H_2Br), 36.0 (*C*H_2CH_2OH), 32.6 (2C, *C*H_2CH_2Br, *C*H_2CHCH(CH_2)$_2$OH), 29.4 (4C, *C*H_2), 29.1 (*C*H_2), 28.8 (*C*H_2), 26.8 (*C*H_2).

IR (ATR): $\tilde{\nu}$ = 3341 (br), 2923 (s), 2853 (s), 1463 (m), 1046 (s), 968 (s), 723 (m), 652 (m).

UV/VIS (MeOH): λ_{max} (log ε) = 228 (2.45), 202 (3.28).

HRMS (CI) *m/z*: $[M\text{-}18 - H]^+$ Calcd for $C_{14}H_{24}Br$ 271.1061; Found 271.1057

2-((1*RS*,2*SR*)-(2-(10-bromodecyl)cyclopropyl)ethan-1-ol (28)

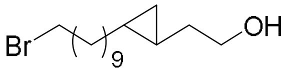

28

Alcohol **28** was prepared following general procedure 5:
Alcohol **26** (2.08 g, 7.14 mmol, 1.0 eq.) in dry CH_2Cl_2 (100 mL).
Diethylzinc solution (35.7 mL, 35.7 mmol, 5.0 eq., 1.0 M in *n*-hexane).
Methylene iodide (2.88 mL, 35.7 mmol, 5.0 eq.).
Flash chromatography [silica gel, CH_2Cl_2].
28 (1.37 g, 4.49 mmol, 63%) as yellowish oil.

TLC [silica gel, CH_2Cl_2]: R_f = 0.25.

^{1}H-NMR (400 MHz, $CDCl_3$): δ = 3.69 (t, ^3J = 6.6 Hz, 2H, CH_2OH), 3.53 (t, ^3J = 6.8 Hz, 2H, CH_2Br), 1.81-1.61 (m, 2H, CH_2CH$_2$Br), 1.56 (s, br, 1H, O*H*), 1.51-1.09 (m, 18H, (CH_2)$_9$), 0.50-0.39 (m, 2H, C*H*C*H*(CH$_2$)$_2$OH), 0.27-0.18 (m, 2H, CHCH_2CH).

^{13}C-NMR (100 MHz, $CDCl_3$): δ = 63.2 (*C*H$_2$OH), 45.1 (*C*H$_2$Br), 37.2 (*C*H$_2$CH$_2$OH), 34.1 (*C*H$_2$CHCH(CH$_2$)$_2$OH), 32.6 (*C*H$_2$CH$_2$Br), 29.6 (2C, *C*H$_2$), 29.5 (2C, *C*H$_2$), 29.4 (*C*H$_2$), 28.9 (*C*H$_2$), 26.9 (*C*H$_2$), 18.4 (*C*HCH(CH$_2$)$_2$OH), 15.1 (*C*H(CH$_2$)$_2$OH), 11.3 (CH*C*H$_2$CH).

IR (ATR): $\tilde{\nu}$ = 3336 (br), 3060 (w), 2991 (w), 2922 (s), 2853 (s), 1462 (m), 1308 (w), 1043 (m), 1017 (m), 892 (w), 724 (m), 652 (m).

UV/VIS (MeOH): λ_{max} (log ε) = 224 (1.93), 202 (2.25).

HRMS (CI) *m/z*: $[M - H]^+$ Calcd for $C_{15}H_{28}BrO$ 303.1324; Found 303.1315

(10-((1*RS*,2*SR*)- 2-(2-Hydroxyethyl)cyclopropyl)decyl)triphenylphosphonium iodide (30)

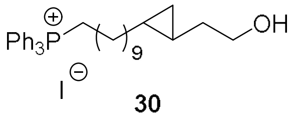

30

Triphenylphosphane (3.10 g, 11.8 mmol, 2.0 eq.) and sodium iodide (4.42 g, 29.5 mmol, 5.0 eq.) were added to a solution of **28** (1.80 g, 5.90 mmol, 1.0 eq.) in CH_3CN (100 mL) and the resulting suspension was heated to reflux for 67 h. Monitoring of the reaction showed still presence of starting material. Triphenylphosphane (3.10 g, 11.8 mmol, 2.0 eq.) and sodium iodide (4.42 g, 29.5 mmol, 5.0 eq.) were added again and the reaction mixture was heated to reflux for an additional 50 h. The solvent was removed under reduced pressure and the residue was extracted with CH_2Cl_2 several times. The extracts were combined, the solvent was removed under reduced pressure and the residue was purified by flash chromatography [silica gel, $CHCl_3$, then $CHCl_3$/MeOH (10:1)]. Phosphonium salt **30** (3.20 g, 5.21 mmol, 88%) was obtained as yellowish oil.[6],[7]

TLC [silica gel, $CHCl_3$/MeOH (10:1)]: R_f = 0.35.

^{1}H-NMR (400 MHz, $CDCl_3$): δ = 7.85-7.78 (m, 9H, *m*-/*p*-Ph-C*H*), 7.76-7.69 (m, 6H, *o*-Ph-C*H*), 3.68 (t, ^3J = 6.7 Hz, 2H, CH_2OH), 3.66-3.56 (m, 2H, PCH_2), 1.81 (s, br, 1H, O*H*), 1.69-1.58 (m,

4H, $PCH_2CH_2CH_2$), 1.51-1.44 (m, 2H, CH_2CH_2OH), 1.38-1.03 (m, 14H, $(CH_2)_7$), 0.48-0.38 (m, 2H, $CHCH(CH_2)_2OH$), 0.25-0.16 (m, 2H, $CHCH_2CH$).

^{13}C-NMR (100 MHz, $CDCl_3$): δ = 135.1 (3C, d, J = 3.0 Hz, *p*-Ph-*C*H), 133.6 (6C, d, J = 10.0 Hz, *o*-Ph-*C*H), 130.5 (6C, d, J = 12.5 Hz, *m*-Ph-*C*H), 118.1 (3C, d, J = 85.9 Hz, *ipso*-Ph-*C*), 62.9 (CH_2OH), 37.2 (CH_2CH_2OH), 34.0 ($CH_2CHCH(CH_2)_2OH$), 30.4 (d, J = 15.5 Hz, $PCH_2CH_2CH_2$), 29.4 (2C, CH_2), 29.3 (CH_2), 29.2 (CH_2), 29.0 (2C, CH_2), 23.1 (d, J = 50.1 Hz, PCH_2), 22.5 (d, J = 4.4 Hz, PCH_2CH_2), 18.3 ($CHCH(CH_2)_2OH$), 15.1 ($CH(CH_2)_2OH$), 11.3 ($CHCH_2CH$).

IR (ATR): $\tilde{\nu}$ = 3373 (br), 3054 (w), 2988 (w), 2921 (m), 2851 (m), 1587 (w), 1484 (w), 1462 (w), 1437 (m), 1316 (w), 1187 (w), 1163 (w), 1111 (s), 1042 (m), 996 (m), 789 (w), 746 (m), 721 (s), 688 (s), 531 (s).

UV/VIS (MeOH): λ_{max} (log ε) = 274 (3.36), 267 (3.45), 222 (4.56), 203 (4.70).

MS (ESI+): *m/z* (%) = 487.3 (100) $[M-I]^+$.

HRMS (ESI) *m/z*: $[M - I]^+$ Calcd for $C_{33}H_{44}OP$ 487.3124; Found 487.3123

2-((1*RS*,2*SR*)-2-((*E*)-11-(1-Trityl-1*H*-imidazol-4-yl)undec-10-en-1-yl)cyclopropyl)-ethan-1-ol (21)

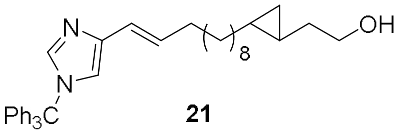

21

Alcohol **21** was prepared following general procedure 5:
Alcohol **19** (50 mg, 112 µmol, 1.0 eq.) in dry CH_2Cl_2 (5 mL).
Diethylzinc solution (558 µL, 558 µmol, 5.0 eq., 1.0 M in *n*-hexane).
Methylene iodide (26.8 mg, 558 µmol, 5.0 eq.).
Flash chromatography [silica gel, CH_2Cl_2].
Alcohol **21** (20 mg, 43 µmol, 35 %) as yellowish oil.

TLC [silica gel, CH_2Cl_2/MeOH (100:1)]: R_f = 0.29.

^{1}H-NMR (400 MHz, $CDCl_3$): δ = 7.36 (d, ^4J = 1.3 Hz, 1H, NC*H*N), 7.34-7.30 (m, 9H, *m*-/*p*-Ph-C*H*), 7.17-7.12 (m, 6H, *o*-Ph-C*H*), 6.66 (d, ^4J = 1.3 Hz, 1H, NCC*H*N), 6.34 (td, ^3J = 6.8 Hz, 15.7 Hz, 1H, NCCHC*H*), 6.20 (d, ^3J = 15.7 Hz, 1H, NCC*H*CH), 3.68 (t, ^3J = 6.7 Hz, 2H, CH_2OH), 2.18-2.06 (m, 2H, $NCCHCHCH_2$), 1.51-1.06 (m, 18H, $(CH_2)_9$), 0.49-0.38 (m, 2H, $CHCH(CH_2)_2OH$), 0.25-0.17 (m, 2H, $CHCH_2CH$).

^{13}C-NMR (100 MHz, $CDCl_3$): δ = 142.4 (3C, *ipso*-Ph-*C*), 139.5 (N*C*CHN), 138.9 (N*C*HN), 129.8 (7C, *o*-Ph-*C*H, NCCH*C*H), 128.0 (9C, *m*-/*p*-Ph-*C*H), 121.3 (NC*C*HCH), 118.0 (NC*C*HN), 75.2 (CPh_3), 63.0 (CH_2OH), 37.3 (CH_2), 34.1 (CH_2), 32.8 ($NCCHCHCH_2$), 29.5 (2C, CH_2), 29.4 (3C, CH_2), 29.3 (CH_2), 29.2 (CH_2), 18.4 ($CHCH(CH_2)_2OH$), 15.2 ($CH(CH_2)_2OH$), 11.3 ($CHCH_2CH$).

IR (ATR): $\tilde{\nu}$ = 3322 (br), 3059 (w), 2991 (w), 232 (m), 290 (m), 157 (w), 1490 (m), 1468 (m), 1444 (m), 1220 (w), 1184 (w), 1157 (w), 1128 (m), 1083 (w), 1035 (m), 78 (m), 904 (w), 869 (w), 824 (w), 748 (s), 700 (s), 656 (m), 638 (m).

UV/VIS (MeOH): λ_{max} (log ε) = 240 (4.22), 204 (4.74).

MS (ESI+): *m/z* (%) = 104.7 (23) $[2M+H]^+$, 569.4 (9) $[M+Na]^+$, 547.4 (65) $[M+H]^+$, 243.1 (100) $[CPh_3]^+$, 165.1 (67).

HRMS (ESI) *m/z*: $[M + H]^+$ Calcd for $C_{38}H_{47}N_2O$ 547.3683; Found 547.3687

2-((1*RS*,2*SR*)-2-((*E*)-11-(1-Trityl-1*H*-imidazol-4-yl)undec-10-en-1-yl)cyclopropyl)-acetic acid (23)

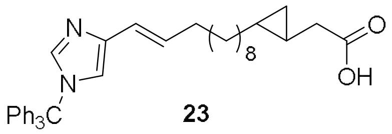

23

Chromium trioxide (388 mg, 3.88 mmol, 2.0 eq.) was placed in a mixture of conc. sulfuric acid (0.36 ml) and water (2.16 mL) at 0 °C, followed by dropwise addition of a solution of alcohol **21** (1.06 g, 1.5 mmol, 1.0 eq.) in acetone (40 mL).[S27] The resulting reaction mixture was allowed to warm to r.t. and stirred for 4.5 h at ambient temperature. The reaction was quenched by addition of isopropanol (40 mL). After addition of EtOAc (50 mL) and sat. NaCl solution (50 mL), the layers were separated and the aqueous layer was extracted with EtOAc (3 x 40 mL). The combined organic layers were washed with sat. NaCl solution (3 x 40 mL), dried with $MgSO_4$ and the solvent was removed under reduced pressure. The residue was purified by flash chromatography [silica gel, $CHCl_3$/MeOH (20:1)] and acid **23** (434 mg, 0.77 mmol, 40%) was obtained as yellow oil.

TLC [silica gel, $CHCl_3$/MeOH (20:1)]: $R_f = 0.34$.

^{1}H-NMR (400 MHz, $CDCl_3$): δ = 8.34 (s, br, 1H, CO_2H), 7.53 (d, ^4J = 1.1 Hz, 1H, NC*H*N), 7.36-7.29 (m, 9H, *m*-/*p*-Ph-C*H*), 7.17-7.10 (m, 6H, *o*-Ph-C*H*), 6.65 (d, ^4J = 1.1 Hz, 1H, NCC*H*N), 6.33 (td, ^3J = 6.8 Hz, 15.7 Hz, 1H, NCCHC*H*), 6.20 (d, ^3J = 15.9 Hz, 1H, NCC*H*CH), 2.36-2.10 (m, 4H, NCCHCHCH_2, CH_2CO$_2$H), 1.48-1.10 (m, 16H, (CH_2)$_8$), 0.84-0.74 (m, 1H, C*H*CH$_2$CO$_2$H), 0.59-0.49 (m, 1H, C*H*CHCH$_2$CO$_2$H), 0.33-0.26 (m, 2H, CHCH_2CH).

^{13}C-NMR (100 MHz, $CDCl_3$): δ = 142.1 (3C, *ipso*-Ph-*C*H), 138.9 (N*C*CHN), 138.8 (N*C*HN), 130.8 (NCCH*C*H), 129.7 (6C, *o*-Ph-*C*H), 128.1 (9C, *m*-/*p*-Ph-*C*H), 120.6 (NC*C*HCH), 118.0 (NC*C*HN), 75.5 (*C*Ph$_3$), 39.4 (*C*H$_2$CO$_2$H) 33.7 (*C*H$_2$CHCHCH$_2$CO$_2$H), 32.8 (NCCHCH*C*H$_2$), 29.0 (5C, *C*H$_2$), 28.7 (2C, *C*H$_2$), 18.6 (*C*HCHCH$_2$CO$_2$H), 14.4 (*C*HCH$_2$CO$_2$H), 11.6 (CH*C*H$_2$CH).

IR (ATR): $\tilde{\nu}$ = 3060 (w), 232 (m), 291 (m), 1711 (m), 1491 (w), 1445 (m), 1300 (w), 119 (w), 1157 (w), 1128 (w), 1084 (w), 1035 (w), 77 (w), 907 (w), 869 (w), 745 (m), 699 (s), 658 (m), 638 (m), 536 (w).

UV/VIS (MeOH): λ_{max} (log ε) = 250 (3.97), 204 (4.69).

MS (ESI+): *m/z* (%) = 1121.7 (21) $[2M+H]^+$, 583.3 (6) $[M+Na]^+$, 561.3 (64) $[M+H]^+$, 243.1 (100) $[CPh_3]^+$, 165.1 (36).

HRMS (ESI) *m/z*: $[M + H]^+$ Calcd for $C_{38}H_{45}N_2O_2$ 561.3476; Found 561.3480

2-((1*RS*,2*SR*)-2-((*E*)-11-(1*H*-imidazol-4-yl)undec-10-en-1-yl)cyclopropyl)acetic acid (24)

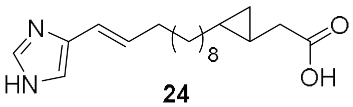

24

Acid **23** (391 mg, 0.70 mmol, 1.0 eq.) was heated at reflux temperature for 7 h in 5% acetic acid (1.00 mL) in methanol (20 mL). [S17] The solvent was removed under reduced pressure and the residue purified by flash chromatography [silica gel, $CHCl_3$/MeOH/NH_3 (40:10:1)]. Acid **24** (107 mg, 0.34 mmol, 48%) was obtained as colorless solid.

TLC [silica gel, $CHCl_3$/MeOH/NH_3 (40:10:1)]: R_f = 0.13.

^{1}H-NMR (600 MHz, CD_3OD): δ = 7.78 (s, 1H, NC*H*N), 6.99 (s, 1H, NCC*H*N), 6.28 (d, ^3J = 16.0 Hz, 1H, NCC*H*CH), 6.18 (dt, ^3J = 16.0 Hz, 6.9 Hz, 1H, NCCHC*H*), 2.21-2.10 (m, 4H, NCCHCHCH2, CH2CO2H), 1.50-1.43 (m, 2H, NCCHCHCH2CH2), 1.43-1.16 (m, 14H, (CH2)7), 0.77-0.70 (m, 1H, CHCH2CO2H), 0.56-0.50 (m, 1H, C*H*CHCH$_2$CO$_2$H), 0.29 (tdd, ^3J = 4.7 Hz, 8.2 Hz, 19.1 Hz, 2H, CHC*H*$_2$CH).

^{13}C-NMR (150 MHz, CD_3OD): δ = 178.2 (*C*O$_2$H), 136.2 (N*C*HN), 135.8 (N*C*CHN), 131.6 (NCCH*C*H), 119.8 (NC*C*HCH), 119.0 (NC*C*HN), 40.5 (*C*H$_2$CO$_2$H), 35.2 (*C*H$_2$CHCHCH$_2$CO$_2$H), 34.0 (NCCHCH*C*H$_2$), 30.8 (*C*H$_2$), 30.7 (*C*H$_2$), 30.6 (3C, *C*H$_2$), 30.5 (NCCHCHCH$_2$*C*H$_2$), 30.3 (*C*H$_2$), 19.7 (*C*HCHCH$_2$CO$_2$H), 15.8 (*C*HCH$_2$CO$_2$H), 12.3 (CH*C*H$_2$CH).

IR (ATR): $\tilde{\nu}$ = 315 (w), 3140 (w), 2990 (w), 2915 (s), 2847 (s), 1675 (m), 1565 (w), 1466 (w), 1437 (w), 1401 (w), 1361 (w), 1246 (w), 1213 (w), 1191 (w), 1106 (m), 1024 (m), 77 (s), 811 (s), 721 (m), 639 (s).

UV/VIS (MeOH): λ_{max} (log ε) = 250 (4.14).

MS (ESI+): *m/z* (%) = 637.5 (20) $[2M+H]^+$, 341.2 (8) $[M+Na]^+$, 319.2 (100) $[M+H]^+$, 273.2 (28).

HRMS (ESI) *m/z*: $[M + H]^+$ Calcd for $C_{19}H_{31}N_2O_2$ 319.2380; Found 319.2379.

5.9 Methods for cultivation, manipulation and analysis of *Stigmatella aurantiaca*

Fermentation and isolation conditions – Stigmatella aurantiaca Sg a15 and its decendants were grown in liquid tryptone medium at 30 °C and 200 rpm and maintained on TS (tryptone-soy) agar plates, containing kanamycin when required for selection. For liquid cultivation, a 50 mL shaking flask culture of *S. aurantiaca* Sg a15 was typically grown for 72–84 hours, clarified by centrifugation, and the supernatant was extracted with excess ethyl acetate. Following evaporation of the solvent until complete dryness, the solid residue was re-dissolved in 1 mL of methanol. Upscaling was done by cultivation in 5 L shaking flasks (1.5 L filling volume) using a 15 mL starter culture per flask and adjusting solvent amounts accordingly.

HPLC and MS analysis - For production control, a 5 µL aliquot was used for injection into an Agilent 1100 series HPLC-DAD system coupled to a HCTplus iontrap MS device (Bruker Daltonik, Bremen, Germany). Chromatographic separation was performed on a Luna RP-C18 column (100 x 2 mm, 2.5 µm particle size; Phenomenex, Germany) equipped with a 4 x 2 mm C18 guard cartridge. The mobile phase system consisted of water (A) and acetonitrile (B),

each containing 0.1 % formic acid. Linear gradients from 5 %B to 95 %B were routinely employed at a flow rate of 0.4 mL/min, and the column temperature was held at 32 °C. MS analysis was carried out in ESI positive ionization mode. High-resolution MS measurements were performed as described by Hoffmann *et al.* [S1] using a BEH-C18 column (100 x 2 mm, 1.7 µm particle size; Waters, USA) for LC separation coupled to a maXis time-of-flight mass spectrometer (Bruker Daltonik, Germany).

Screening of a library of Sg a15 mutants and plasmid recovery – The construction of a library of *S. aurantiaca* mutants harboring random insertional gene inactivations was reported previously.[S28] Briefly, *S. aurantiaca* Sg a15 was transformed with the *mariner*-based transposon plasmid pMycoMar,[S29] and viable clones were subsequently selected on TS agar containing kanamycin and transferred to 150 µL tryptone medium (with kanamycin added) in 96-well plates. After growth for 5 days at 30 °C and 700 rpm on a wellplate incubator (Infors), 50 µL aliquots from each well were added to 100 µL methanol, the suspension was clarified by centrifugation and 15 µL aliquots from the supernatant were subjected to HPLC-MS analysis. For plasmid recovery from Imidacin-negative mutant clone Sga15-29F10, chromosomal DNA was digested with *Sma*I, re-ligated using T4 DNA ligase and used for the transformation of *E. coli* DH5αλpir. The obtained plasmid p29F10 was used to determine the cloned sequence flanking the transposable element, using primers K388 and K390.[S29] Treatment of p29F10 with *Sma*I followed by ligation to the *Sma*I-digestested pBluescriptSK vector backbone gave plasmid p29F10Sma, which was used for sequencing into the region in between the former chromosomally-encoded *Sma*I restriction sites, utilizing standard M13 primers.

Insertion of an upstream npt *promotor upstream to the imidacin gene cluster* – The *npt* (neomycin phosphotransferase) promotor sequence was PCR-amplified from the plasmid pCR2.1Topo (Invitrogen) using Phusion polymerase (Finnzymes) and primers Mch71 and Mch72,[S30] also adding a RBS sequence. The amplified fragment 157 bp in size was gel-purified and used in a subsequent overlap-extension PCR step, in combination with primers Mch71 and Sga15con4b and a 1073-bp fragment previously amplified from a cosmid containing the imidacin gene cluster.[S31] Following the addition of single A-overhangs to the resulting PCR product by Taq polymerase, the gel-purified fragment 1209 bp in size was cloned into the pCR2.1Topo vector to give plasmid pTOPO-Sga15act. The correct fusion of the *npt* sequence to the *orf1*-homologous region was verified by sequencing, and the plasmid was used for the transformation of *S. aurantiaca* Sg a15, yielding strain Sga15-npt.[S31] This strain showing increased imidacin production was cultivated as described above and used for compound isolation.

Targeted gene inactivation in S. aurantiaca *Sg a15* – An internal fragment (500–800 bp in size) of the gene to be inactivated was PCR-amplified from genomic DNA using Taq polymerase and sequence-specific primers (listed in [S31]), and cloned into the pCR2.1Topo vector. The resulting inactivation plasmid was used for electroporation of *S. aurantiaca* Sg a15 as previously reported.[S32] Viable colonies were obtained on TS agar supplemented with kanamycin, and chromosomal DNA was pepared from liquid cultures using the Puregene Kit (Gentra). Integration of the plasmid into the correct genomic locus was routinely verified by PCR, using control primers which are located outside the amplified region, in combination with vector-specific primers pTOPOin and pTOPOout.[S31] This approach was used to construct a mutant with inactivated imidacin biosynthetic gene cluster, and another mutant deficient in histidine ammonia lyase activity as a consequence of inactivation of the HAL-encoding gene.[S31]

Feeding of urocanate - As uniformly ^{13}C-labeled urocanic acid was not commercially available, recombinant histidine ammonia lyase (HAL) was used for the *in-vitro* conversion of $^{13}C_6$-L-histidine to $^{13}C_6$-urocanic acid.[S31] The Sga15 HAL-inactivated mutant of Sg a15 was chosen for the subsequent feeding experiment, since it conveniently allowed for the incorporation of $^{13}C_6$-urocanate without intrinsic background that would otherwise result from the HAL-catalyzed conversion of non-labelled histidine inside the wildtype strain. Extracts from a cultivation of HAL-inactivated Sg a15 in the presence of ~1 mM $^{13}C_6$-urocanic acid were analyzed by HPLC-coupled MS. Signals with 325 *m/z* and 353 *m/z* were detected at the same retention times that were previously observed for the non-labelled compounds with 319 *m/z* and 347 *m/z*, respectively, and the corresponding ions were subjected to multiple stages of collisionally-induced dissociation MS.[S33] This analysis revealed unambigously that all carbon atoms from urocanic acid were incorporated into the imidacins, due to the observed mass shifts of +6 Da.

Bioinformatic tools – Sequence searches were performed using the NCBI BLAST utility as part of the Geneious Prime software, which was also used for sequence comparison and detailed analysis. AntiSMASH version 5 was used for the initial annotation of the *imd* biosynthetic gene cluster.

6 References

(S1) Hoffmann, T.; Krug, D.; Bozkurt, N.; Duddela, S.; Jansen, R.; Garcia, R.; Gerth, K.; Steinmetz, H.; Müller, R., Correlating chemical diversity with taxonomic distance for discovery of natural products in myxobacteria. *Nat. Commun.* **2018**, 9, 803.

(S2) Bertinaria, M.; Di Stilo, A.; Tosco, P.; Sorba, G.; Poli, E.; Pozzoli, C.; Coruzzi, G.; Fruttero, R.; Gasco, A., [3-(1H-Imidazol-4-yl)propyl]guanidines containing furoxan moieties. *Bioorg. Med. Chem.* **2003**, 11, 1197–1205.

(S3) Fujimoto, T.; Tobisu, M.; Konishi, N.; Kawamura, M.; Tada, N.; Takagi, T.; Kubo, K., Synthesis and biological evaluation of the metabolites of 2-(1-{3-[(6-chloronaphthalen-2-yl)sulfonyl]propanoyl}piperidin-4-yl)-5-methyl-1,2-dihydro-3 *H*-imidazo[1,5-c]imidazol-3-one. *Bioorg. Med. Chem.* **2009**, 17, 7993–8002.

(S4) Furukawa, J.; Kawabata, N.; Nishimura, J., Synthesis of cyclopropanes by the reaction of olefins with dialkylzinc and methylene iodide. *Tetrahedron* **1968**, 24, 53–58.

(S5) Karim, M. R. U.; Harunari, E.; Oku, N.; Akasaka, K.; Igarashi, Y., Bulbimidazoles A-C, Antimicrobial and Cytotoxic Alkanoyl Imidazoles from a Marine Gammaproteobacterium *Microbulbifer* Species. *J. Nat. Prod.* **2020**, 83, 1295–1299.

(S6) Montes Vidal, D.; Rymon-Lipinski, A.-L. von; Ravella, S.; Groenhagen, U.; Herrmann, J.; Zaburannyi, N.; Zarbin, P. H. G.; Varadarajan, A. R.; Ahrens, C. H.; Weisskopf, L.; Müller, R.; Schulz, S., Long-Chain Alkyl Cyanides: Unprecedented Volatile Compounds Released by *Pseudomonas* and *Micromonospora* Bacteria. *Angew. Chem. Int. Ed.* **2017**, 56, 4342–4346.

(S7) Donner, J.; Reck, M.; Bunk, B.; Jarek, M.; App, C. B.; Meier-Kolthoff, J. P.; Overmann, J.; Müller, R.; Kirschning, A.; Wagner-Döbler, I., The Biofilm Inhibitor Carolacton Enters Gram-Negative Cells: Studies Using a TolC-Deficient Strain of *Escherichia coli*. *mSphere* **2017**, 2.

(S8) Abad, J.-L.; Fabriàs, G.; Camps, F., Synthesis of deuterated fatty acids to investigate the biosynthetic pathway of disparlure, the sex pheromone of the Gypsy Moth, *Lymantria dispar*. *Lipids* **2004**, 39, 397–401.

(S9) Tojo, G. *Oxidation of alcohols to aldehydes and ketones*: A guide to current common practice; Springer, New York, 2006.

(S10) Brandau, S.; Hoppe, D., Asymmetric synthesis of (2-carbamoyloxy-1-alkenyl)cyclopropanes by intramolecular cycloalkylation. *Tetrahedron* **2005**, 61, 12244–12255.

(S11) Tilley, S. D.; Francis, M. B., Tyrosine-selective protein alkylation using pi-allylpalladium complexes. *J. Am. Chem. Soc.* **2006**, 128, 1080–1081.

(S12) Li, J. J. *Name Reactions for Carbocyclic Ring Formations;* John Wiley & Sons, Inc., Hoboken, New Jersey, 2010.

(S13) Nilewski, C.; Geisser, R. W.; Carreira, E. M., Total synthesis of a chlorosulpholipid cytotoxin associated with seafood poisoning. *Nature* **2009**, 457, 573–577.

(S14) Li, J.-M.; Yong, J.-P.; Aisa, H. A., Synthesis of (*Z/E*)-11-tetradecen-1-ol, a component of *Ostrinia nubilalis* sex pheromone. *Chem. Nat. Compd.* **2008**, 44, 224–226.

(S15) Schlosser, M.; Tuong, H. B.; Schaub, B., The betaine-ylid route to trans-alkenols. *Tetrahedron Lett.* **1985**, 26, 311–314.

(S16) a) Maltais, F.; Jung, Y. C.; Chen, M.; Tanoury, J.; Perni, R. B.; Mani, N.; Laitinen, L.; Huang, H.; Liao, S.; Gao, H.; Tsao, H.; Block, E.; Ma, C.; Shawgo, R. S.; Town, C.; Brummel, C. L.; Howe, D.; Pazhanisamy, S.; Raybuck, S.; Namchuk, M.; Bennani, Y. L., In vitro and in vivo isotope effects with hepatitis C protease inhibitors: enhanced plasma exposure of deuterated telaprevir versus telaprevir in rats. *J. Med. Chem.* **2009**, 52, 7993–

8001; b) Beckmann, C.; Rattke, J.; Sperling, P.; Heinz, E.; Boland, W., Stereochemistry of a bifunctional dihydroceramide Δ 4 -desaturase/hydroxylase from Candida albicans; a key enzyme of sphingolipid metabolism. *Org. Biomol. Chem.* **2003**, 1, 2448–2454;

(S17) Kumar, S.; Jaller, D.; Patel, B.; LaLonde, J. M.; DuHadaway, J. B.; Malachowski, W. P.; Prendergast, G. C.; Muller, A. J., Structure based development of phenylimidazole-derived inhibitors of indoleamine 2,3-dioxygenase. *J. Med. Chem.* **2008**, 51, 4968–4977.

(S18) Chinta, S. P.; Goller, S.; Uhl, G.; Schulz, S., Identification and Synthesis of Branched Wax-type Esters, Novel Surface Lipids from the Spider *Argyrodes elevatus* (Araneae: Theridiidae). *Chem. Biodivers.* **2016**, 13, 1202–1220.

(S19) Charette, A. B.; Juteau, H.; Lebel, H.; Molinaro, C., Enantioselective Cyclopropanation of Allylic Alcohols with Dioxaborolane Ligands: Scope and Synthetic Applications. *J. Am. Chem. Soc.* **1998**, 120, 11943–11952.

(S20) Yue, H.; Waldeck, D. H.; Schrock, K.; Kirby, D.; Knorr, K.; Switzer, S.; Rosmus, J.; Clark, R. A., Multiple Sites for Electron Tunneling between Cytochrome c and Mixed Self-Assembled Monolayers. *J. Phys. Chem. C* **2008**, 112, 2514–2521.

(S21) Al Dulayymi, J. R.; Baird, M. S.; Roberts, E.; Deysel, M.; Verschoor, J., The first syntheses of single enantiomers of the major methoxymycolic acid of *Mycobacterium tuberculosis*. *Tetrahedron* **2007**, 63, 2571–2592.

(S22) Kang, S. H.; Kang, S. Y.; Choi, H.; Kim, C. M.; Jun, H.-S.; Youn, J.-H., Stereoselective Total Synthesis of the Natural (+)-Lasonolide A. *Synthesis* **2004**, 1102–1114.

(S23) Trapella, C.; Fischetti, C.; Pela', M.; Lazzari, I.; Guerrini, R.; Calo', G.; Rizzi, A.; Camarda, V.; Lambert, D. G.; McDonald, J.; Regoli, D.; Salvadori, S., Structure–activity studies on the nociceptin/orphanin FQ receptor antagonist 1-benzyl-*N*-{3-[spiroisobenzofuran-1(3*H*),4′-piperidin-1-yl]propyl} pyrrolidine-2-carboxamide. *Bioorg. Med. Chem.* **2009**, 17, 5080–5095.

(S24) Pospísil, J.; Markó, I. E., Efficient and stereoselective synthesis of allylic ethers and alcohols. *Org. Lett.* **2006**, 8, 5983–5986.

(S25) Liang, B.; Negishi, E.-I., Highly efficient asymmetric synthesis of fluvirucinine A1 via Zr-catalyzed asymmetric carboalumination of alkenes (ZACA)-lipase-catalyzed acetylation tandem process. *Org. Lett.* **2008**, 10, 193–195.

(S26) Houghton, S. R.; Furst, L.; Boddy, C. N., Biomimetic transannular oxa-conjugate addition approach to the 2,6-disubstituted dihydropyran of laulimalide yields an unprecedented transannular oxetane. *J. Org. Chem.* **2009**, 74, 1454–1463.

(S27) a) Tojo, G.; Fernandez, M. *Oxidation of Primary Alcohols to Carboxylic Acids;* Springer, New York, NY, 2007; b) Smith, S. M.; Takacs, J. M., Amide-directed catalytic asymmetric hydroboration of trisubstituted alkenes. *J. Am. Chem. Soc.* **2010**, 132, 1740–1741;

(S28) Sandmann, A.; Dickschat, J.; Jenke-Kodama, H.; Kunze, B.; Dittmann, E.; Müller, R., A type II polyketide synthase from the gram-negative bacterium *Stigmatella aurantiaca* is involved in aurachin alkaloid biosynthesis. *Angew. Chem. Int. Ed. Engl.* **2007**, 46, 2712–2716.

(S29) Sandmann, A.; Sasse, F.; Müller, R., Identification and analysis of the core biosynthetic machinery of tubulysin, a potent cytotoxin with potential anticancer activity. *Chem. Biol.* **2004**, 11, 1071–1079.

(S30) Meiser, P.; Müller, R., Two functionally redundant Sfp-type 4'-phosphopantetheinyl transferases differentially activate biosynthetic pathways in *Myxococcus xanthus*. *ChemBioChem* **2008**, 9, 1549–1553.

(S31) Krug, D., Natural product biosynthesis in myxobacteria: Studies on enzymatic versatility and secondary metabolite diversity. Dissertation, Universität des Saarlandes, Germany, Saarbrücken, 2009. doi: 10.22028/D291-22581.

(S32) Beyer, S.; Kunze, B.; Silakowski, B.; Müller, R., Metabolic diversity in myxobacteria: identification of the myxalamid and the stigmatellin biosynthetic gene cluster of *Stigmatella aurantiaca* Sg a15 and a combined polyketide-(poly)peptide gene cluster from the epothilone producing strain *Sorangium cellulosum* So ce90. *Biochim. Biophys. Acta* **1999**, 1445, 185–195.

(S33) Krug, D.; Müller, R., Discovery of additional members of the tyrosine aminomutase enzyme family and the mutational analysis of CmdF. *ChemBioChem* **2009**, 10, 741–750.
