# Supplementary material for: Identification by Synthesis: Imidacins, Urocanate-Derived Alkaloids from the Myxobacterium Stigmatella aurantiaca
Source: Org Lett. 2024 Jul 22;26(30):6359–63. doi: 10.1021/acs.orglett.4c02036 (PMC11301661; doi:10.1021/acs.orglett.4c02036)
Supplement: Supplementary file 2 — ol4c02036_si_002.pdf [file ol4c02036_si_002.pdf]

6 NMR Spectra of synthetic imidacins

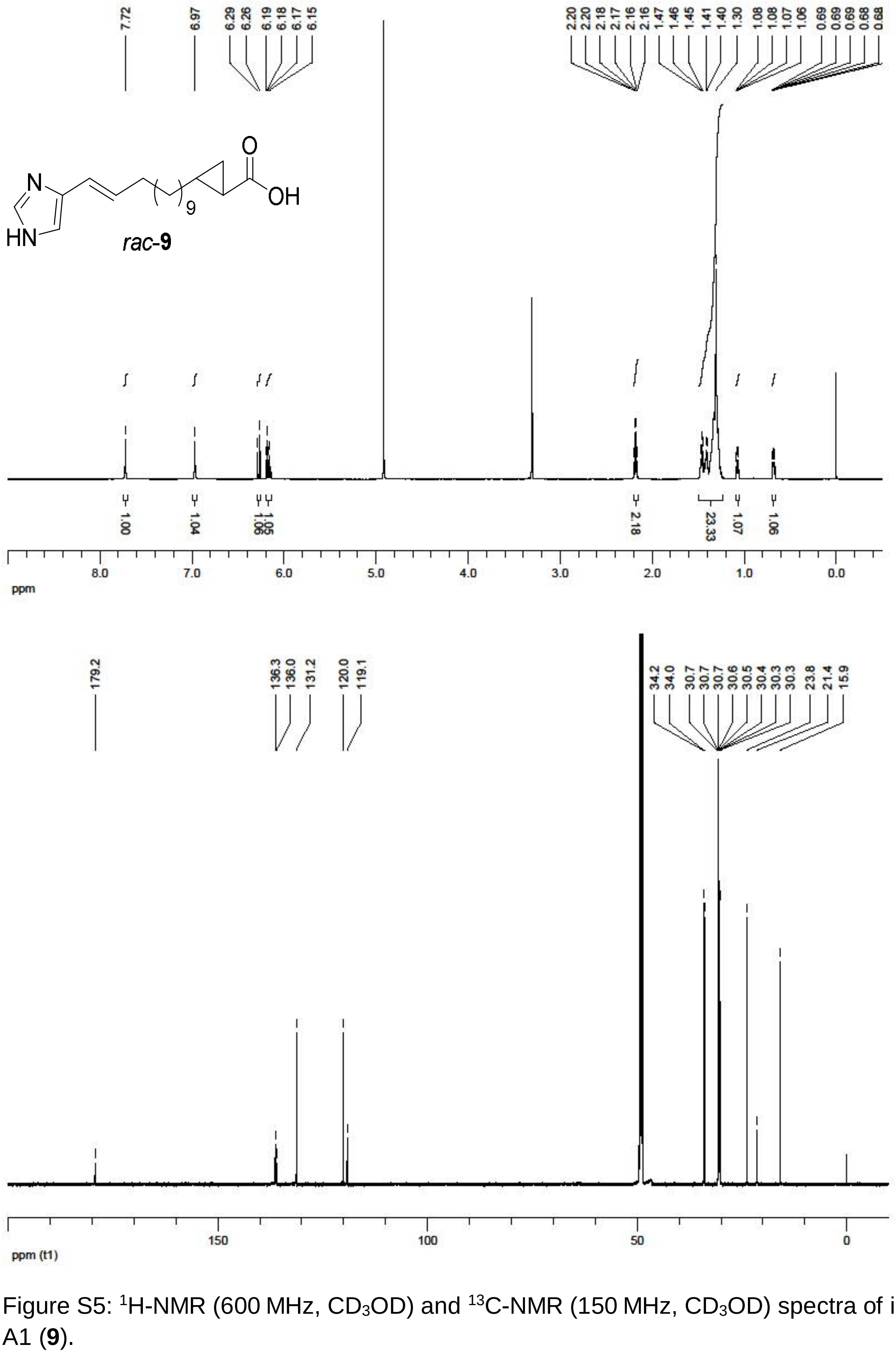

Figure S5: ^{1}H-NMR (600 MHz, CD_3OD) and ^{13}C-NMR (150 MHz, CD_3OD) spectra of imidacin A1 (**9**).

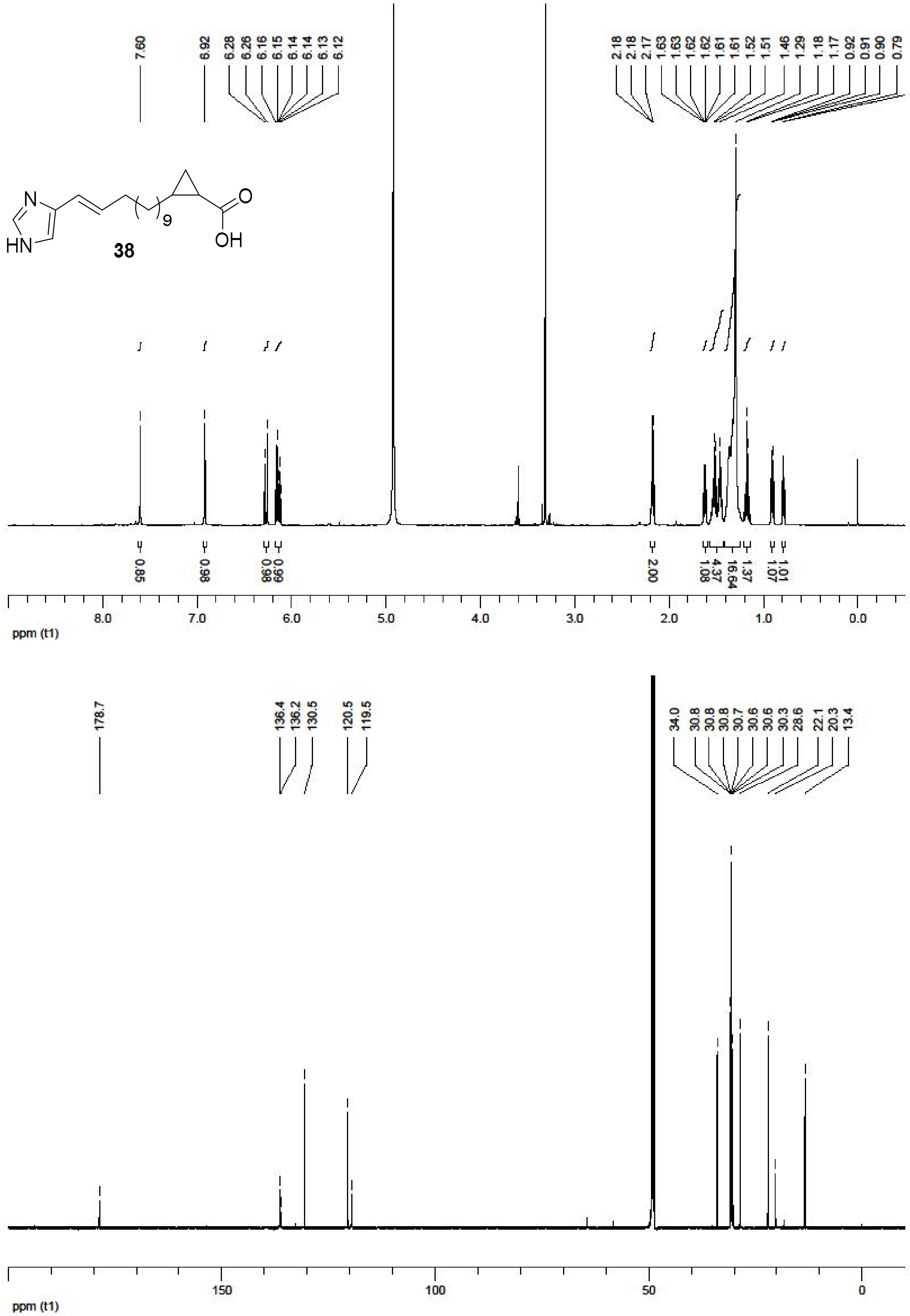

Figure S6: ^{1}H-NMR (600 MHz, CD_3OD) and ^{13}C-NMR (150 MHz, CD_3OD) spectra of *cis*-imidacin A1 (**38**).

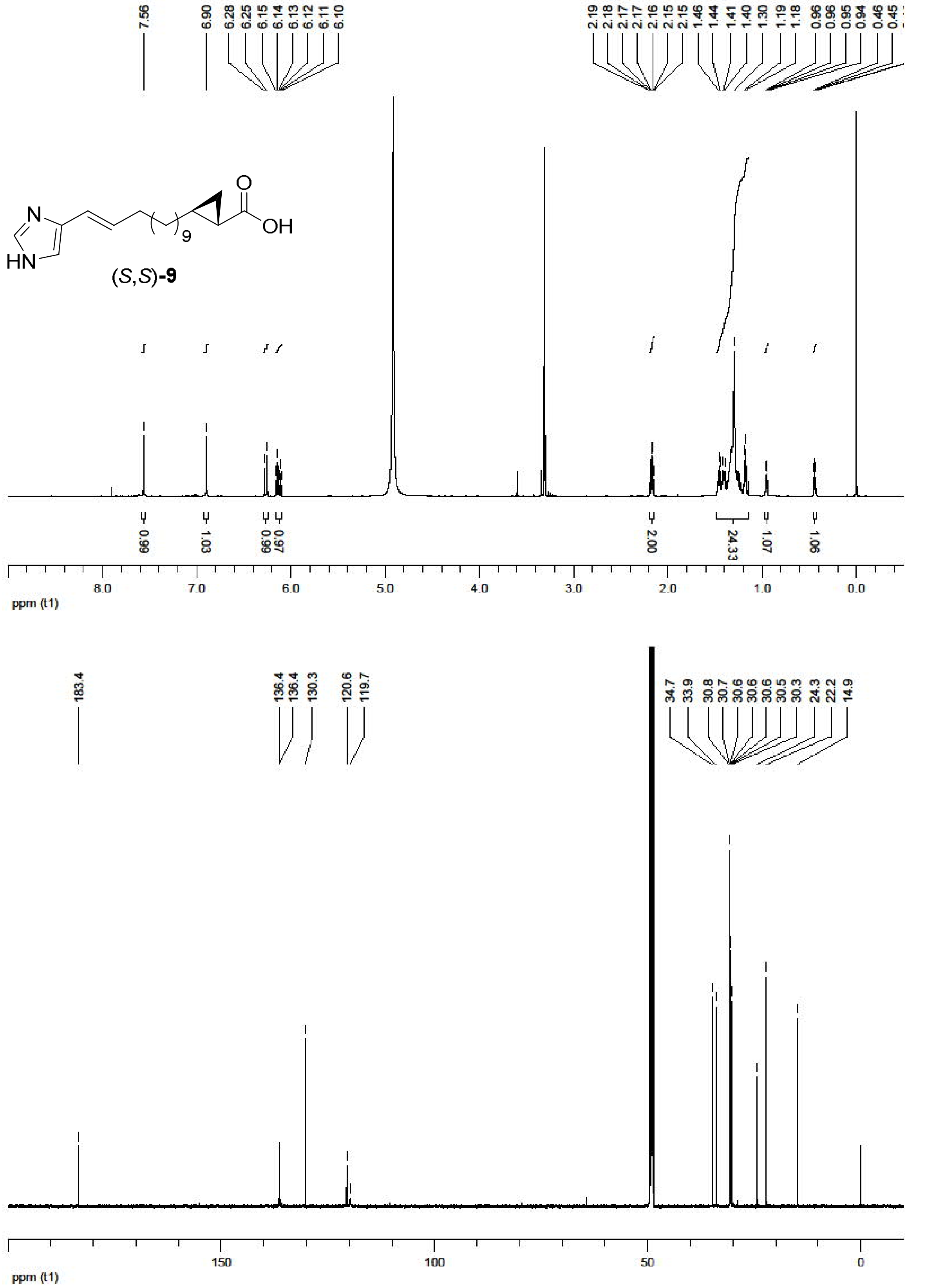

Figure S7: ^{1}H-NMR (600 MHz, CD_3OD + 4 µL NH_3) and ^{13}C-NMR (150 MHz, CD_3OD + 4 µL NH_3) spectra of imidacin (*S*,*S*)-A1 (*S*,*S*-**9**).

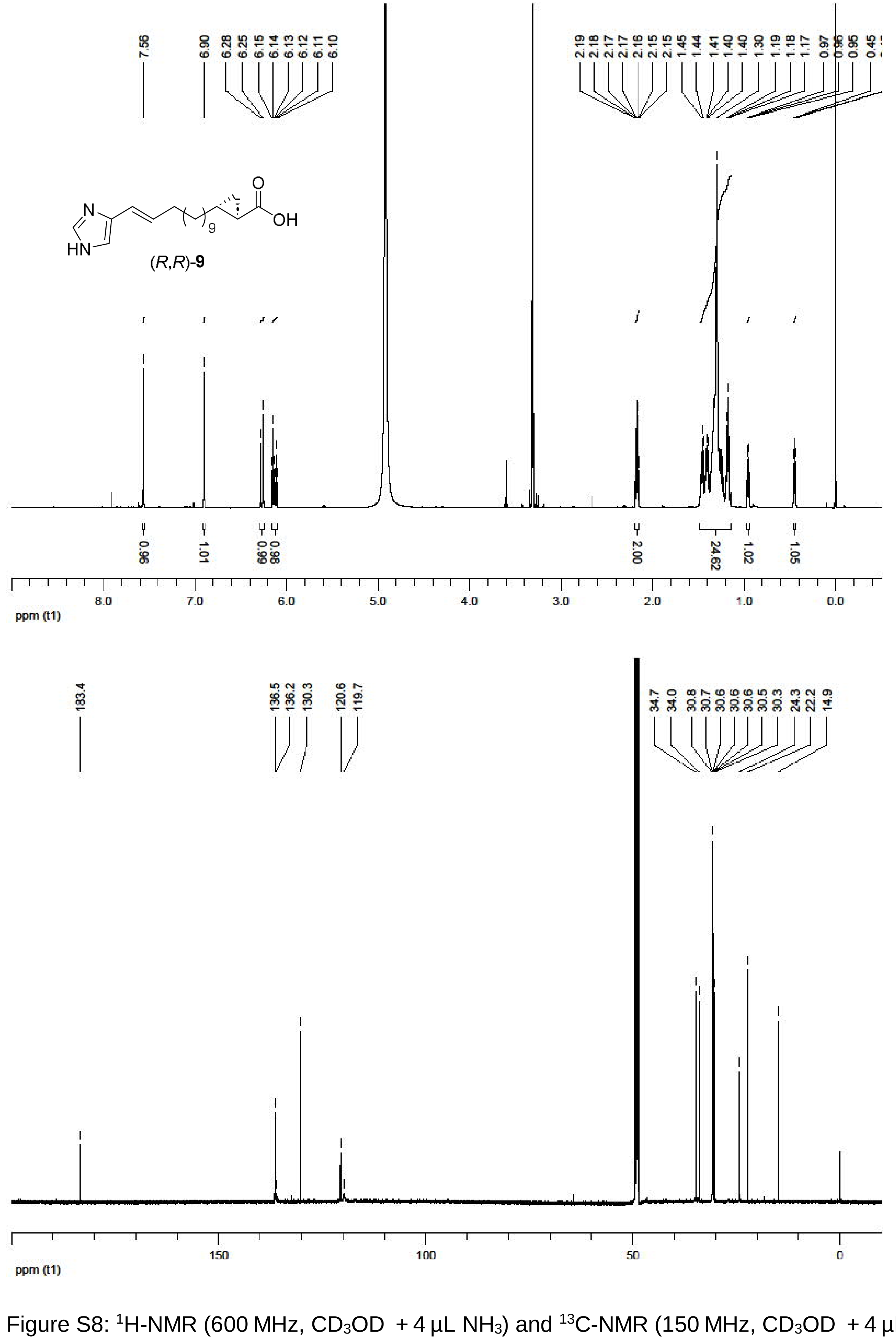

Figure S8: ^{1}H-NMR (600 MHz, CD_3OD + 4 µL NH_3) and ^{13}C-NMR (150 MHz, CD_3OD + 4 µL NH_3) spectra of imidacin (*R*,*R*)-A1 (*R*,*R*-**9**).

^{1}H-NMR (600 MHz, CD_3OD + 4 µL NH_3) of imidacin A2:

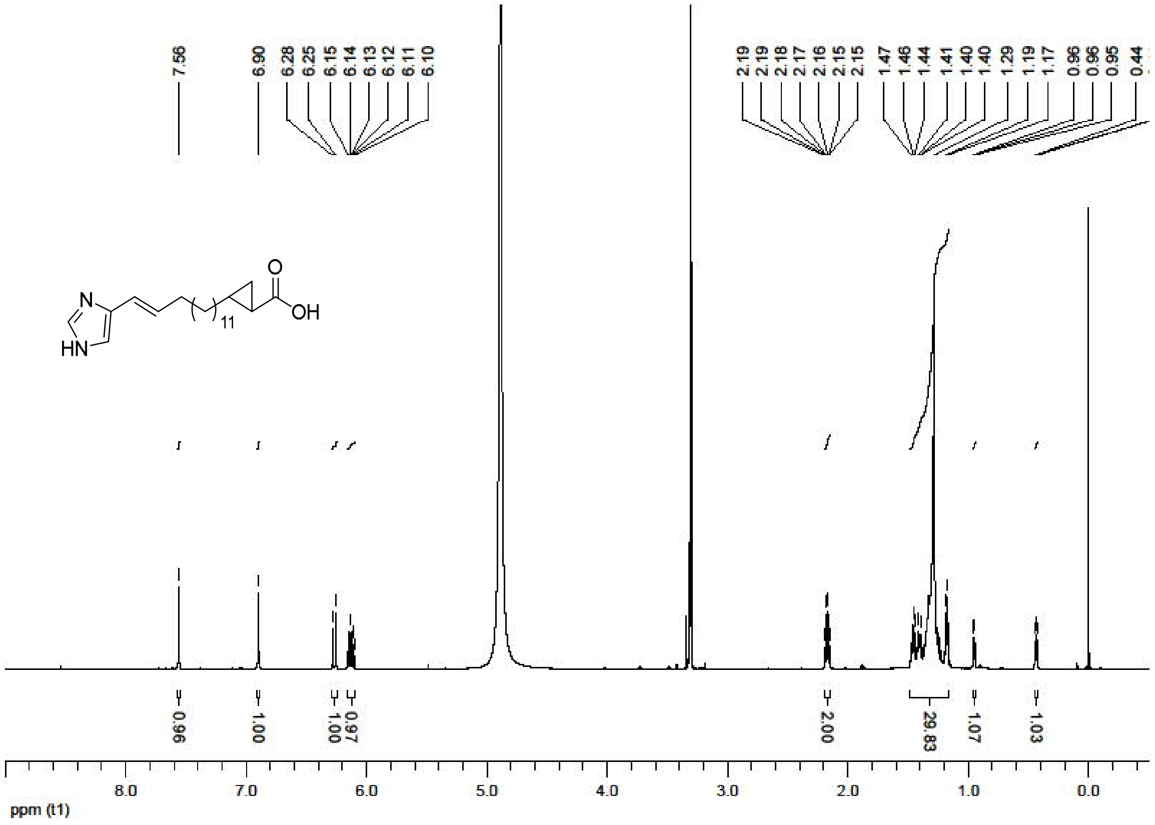

^{13}C-NMR (150 MHz, CD_3OD + 4 µL NH_3) of imidacin A2:

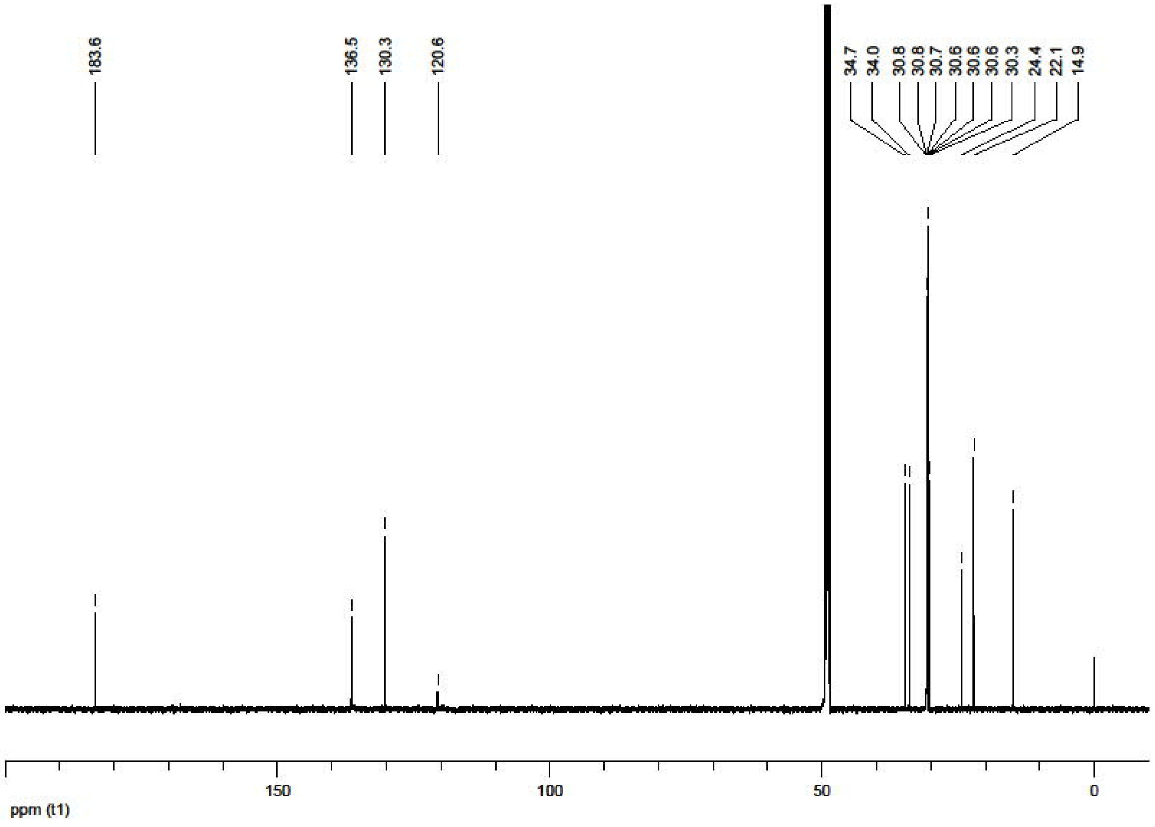

Figure S9: ^{1}H-NMR (600 MHz, CD_3OD + 4 µL NH_3) and ^{13}C-NMR (150 MHz, CD_3OD + 4 µL NH_3) spectra of imidacin A2 (**47**).

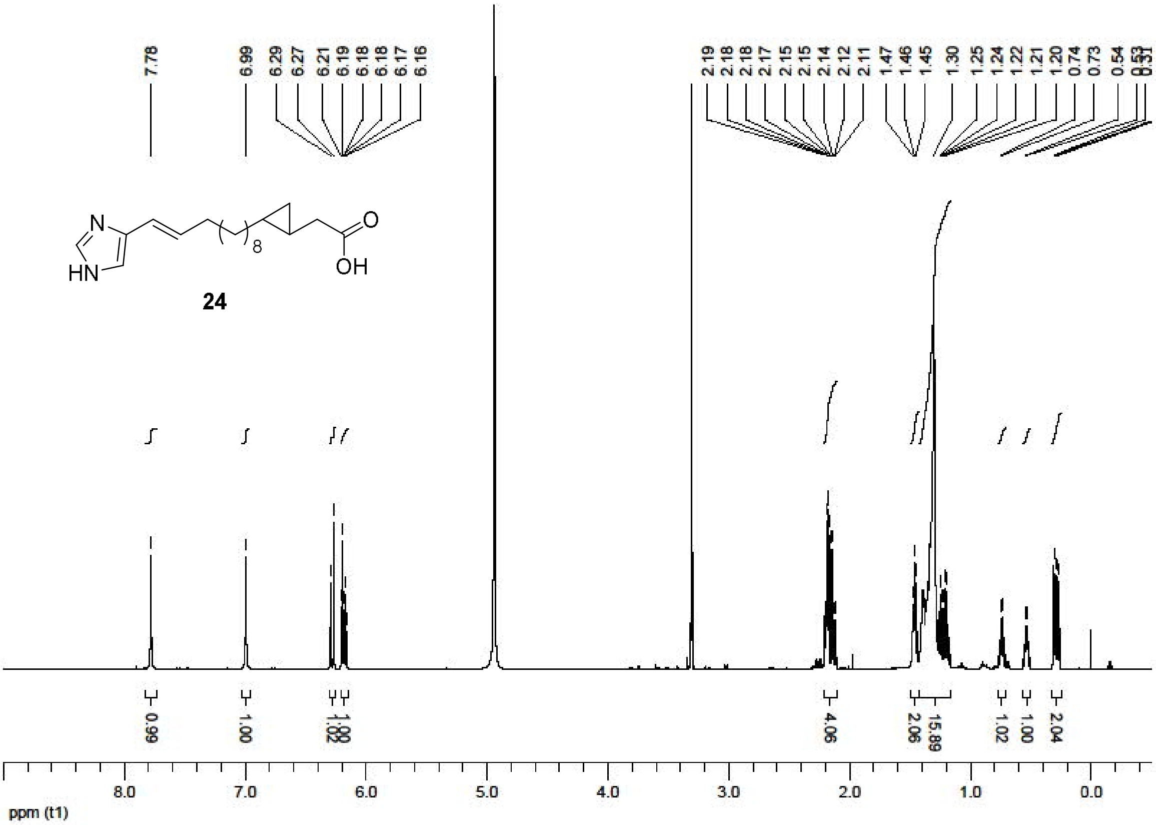

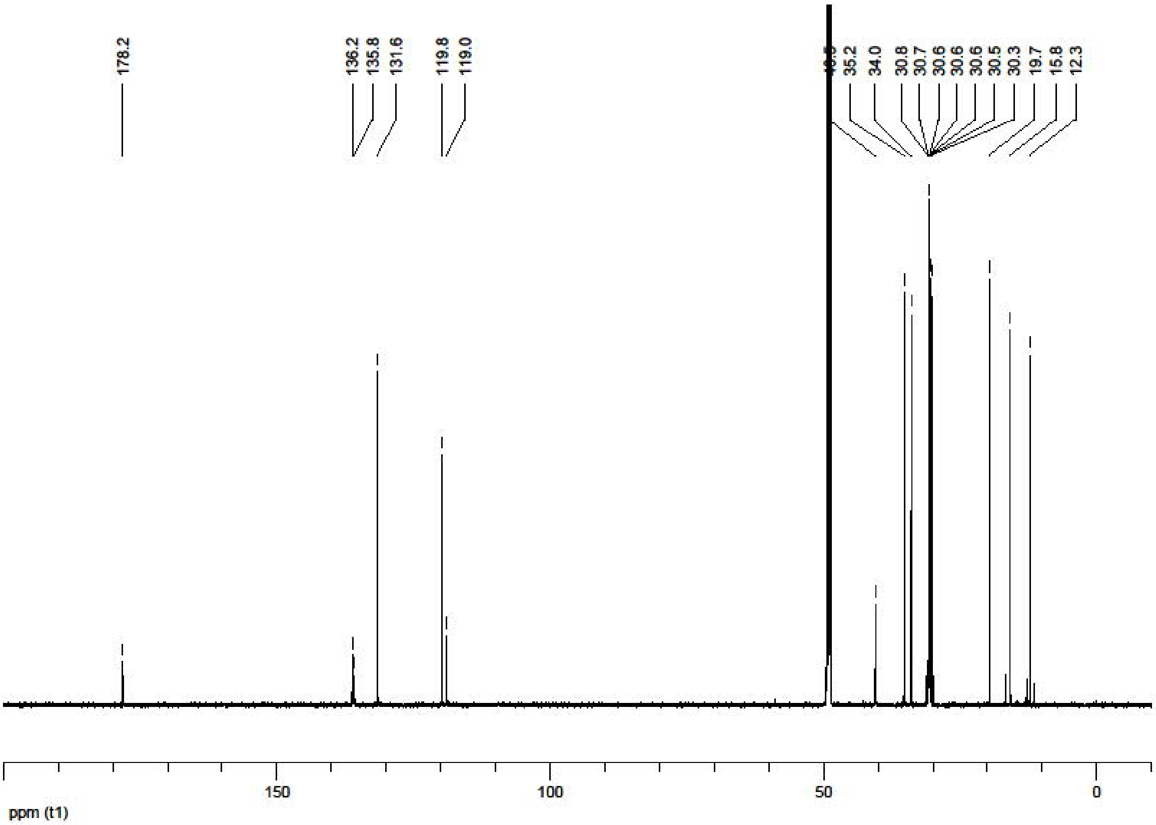

Figure S10: ¹H-NMR (600 MHz, CD_3OD) and ¹³C-NMR (150 MHz, CD_3OD) spectra of imidacin B1 (**24**).

Synthesis of imidacin A1

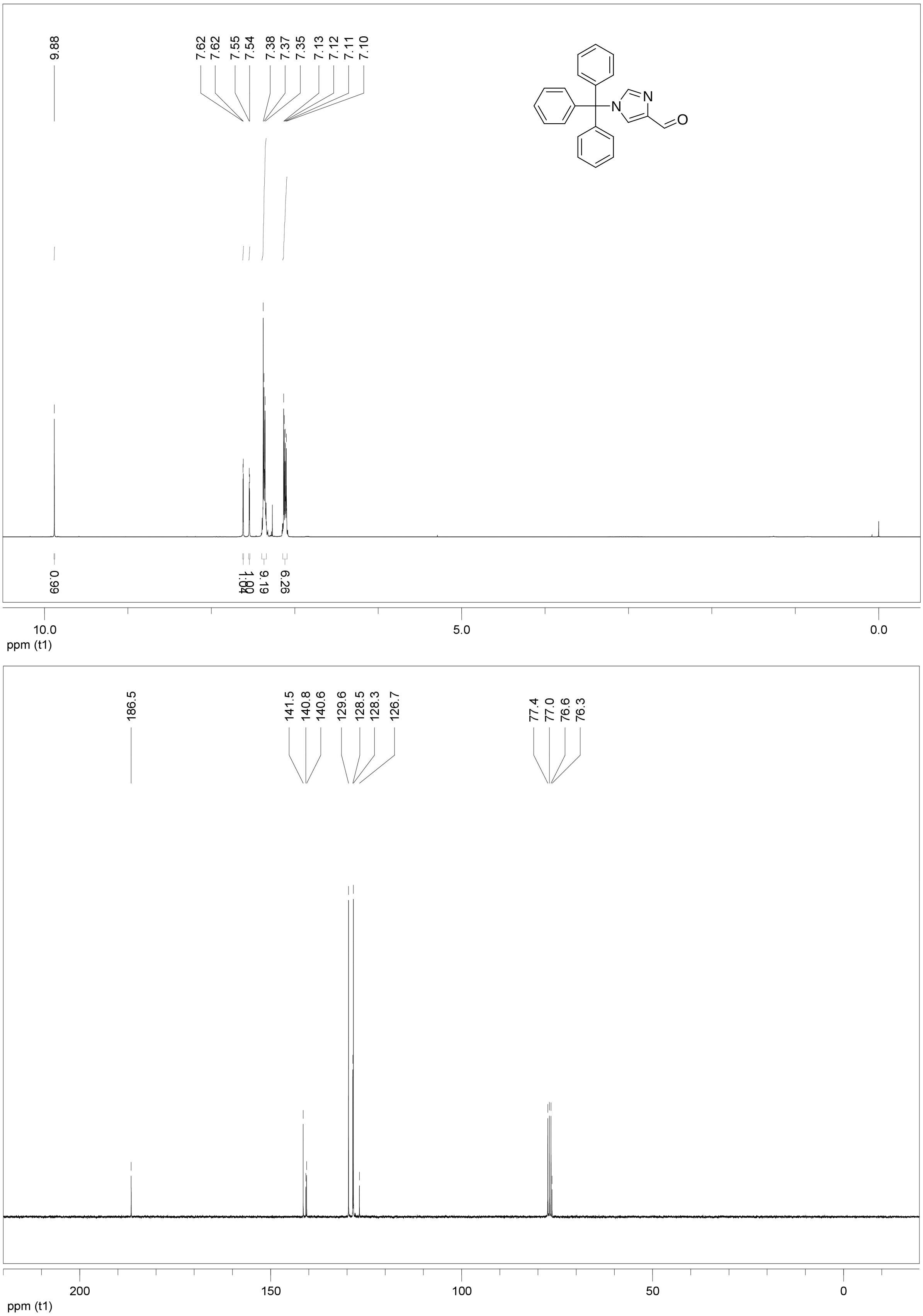

Figure S11: ^{1}H- (300 MHz) and ^{13}C-NMR (75 MHz) spectra of 1-trityl-1*H*-imidazole-4-carbaldehyde (**8**).

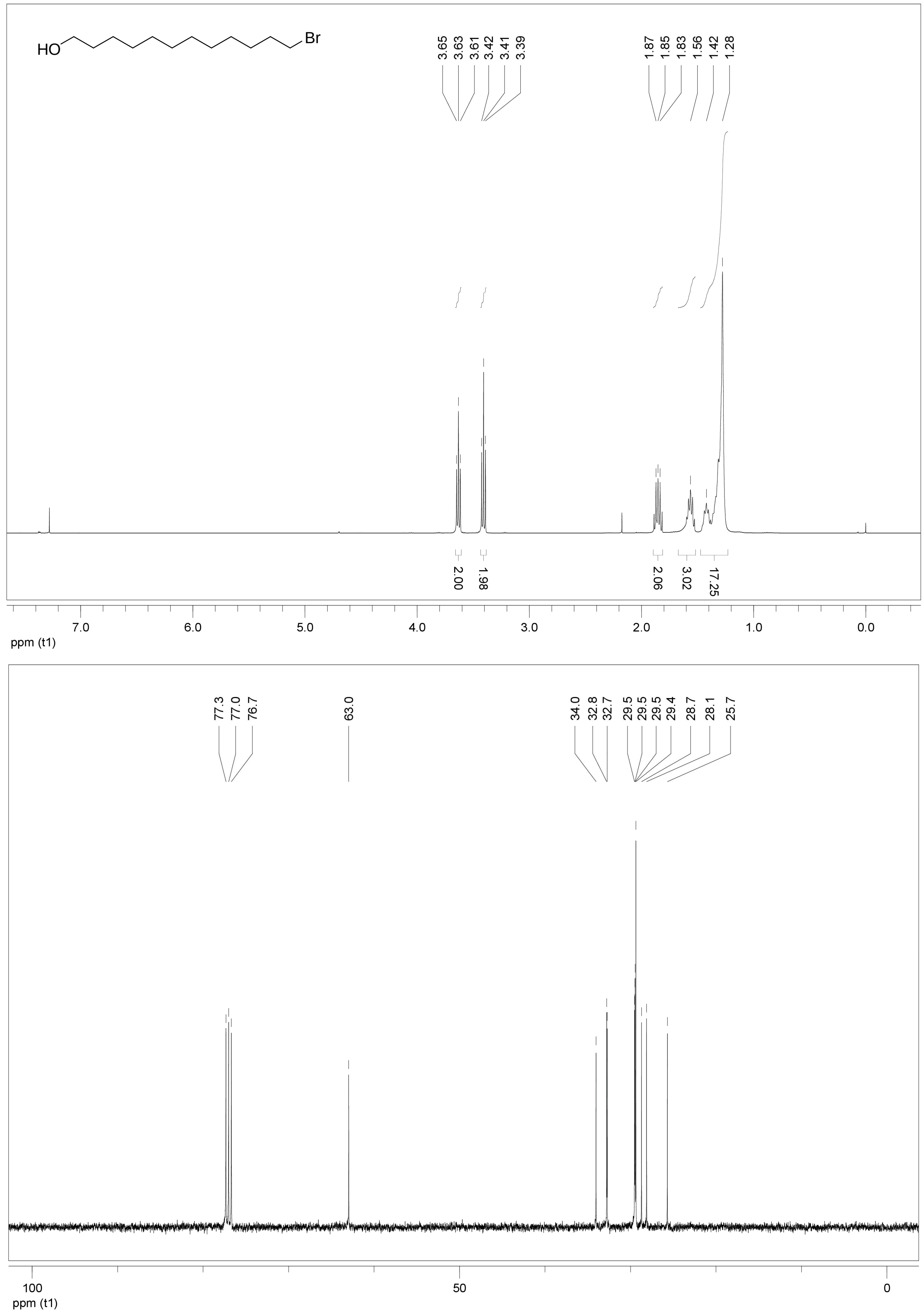

Figure S12: ^{1}H- (400 MHz) and ^{13}C-NMR (100 MHz) spectra of 12-bromododecan-1-ol.

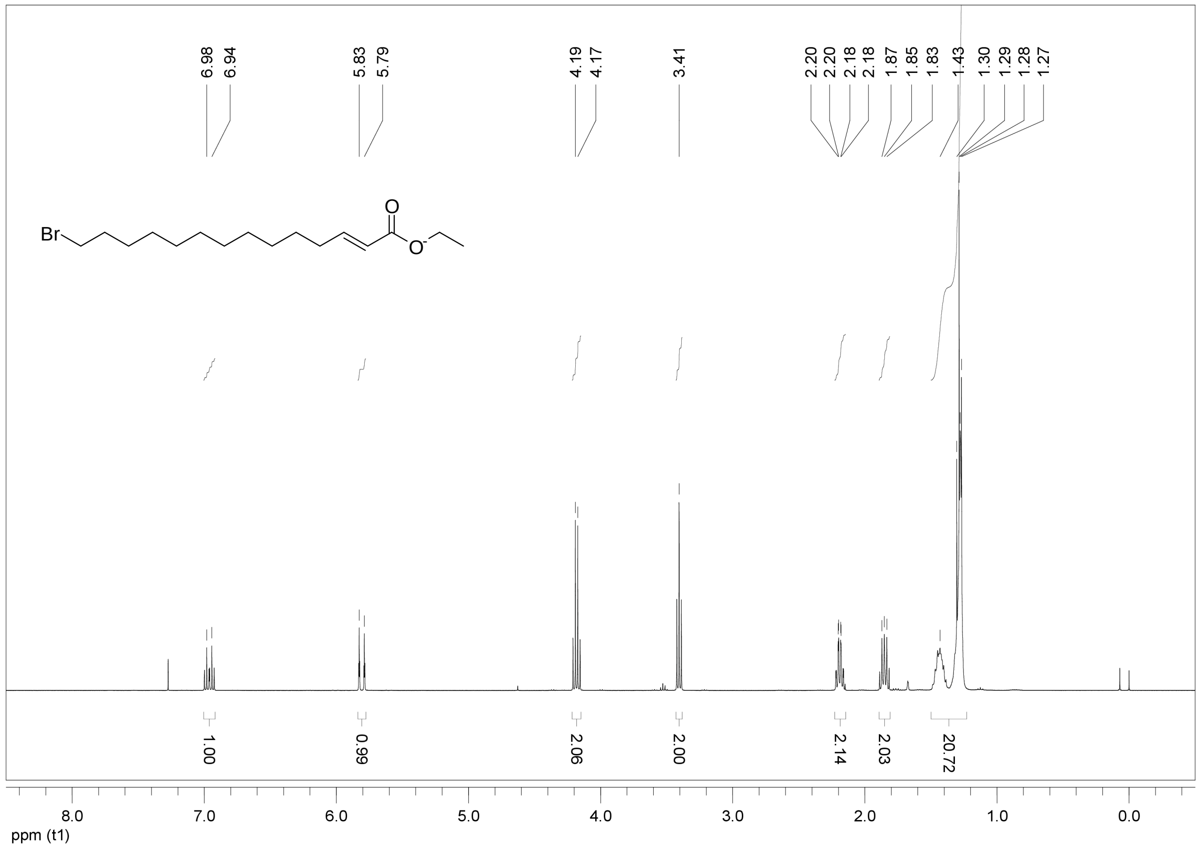

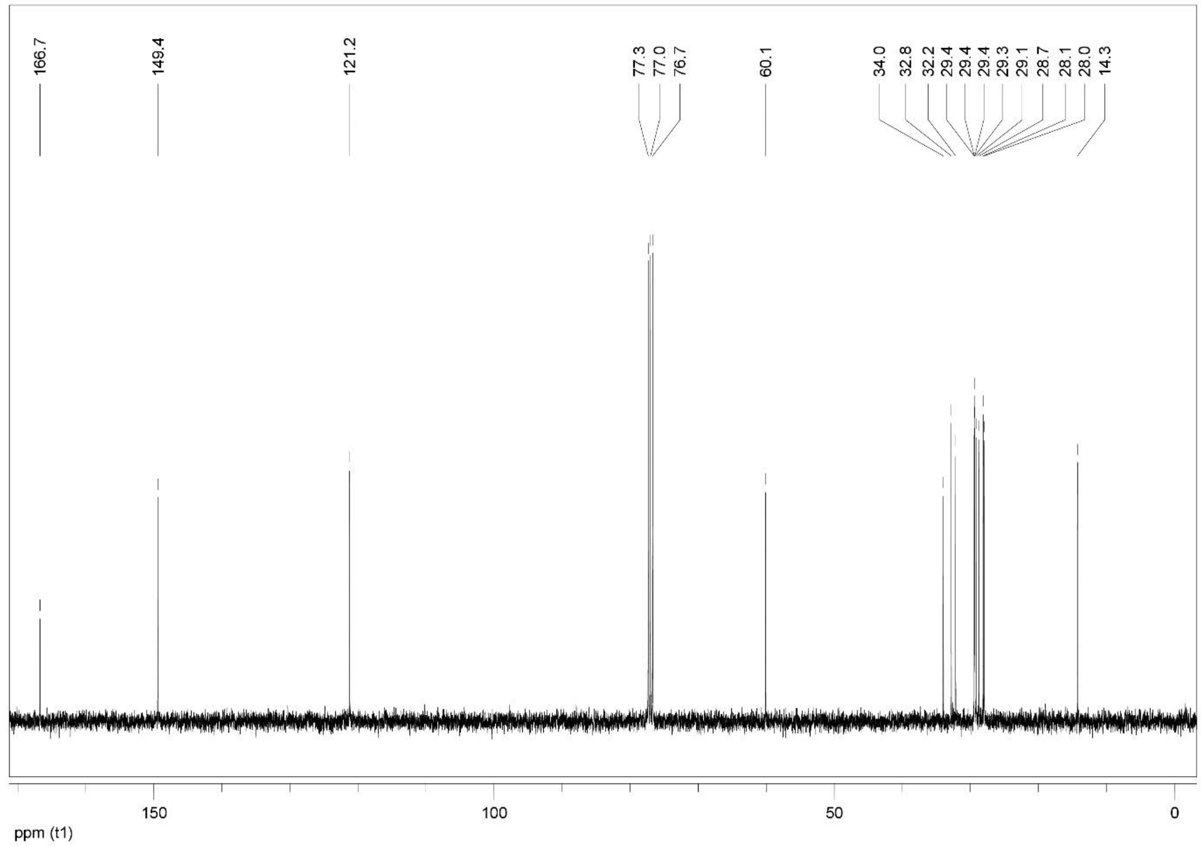

Figure S13: ^{1}H- (400 MHz) and ^{13}C-NMR (100 MHz) spectra of ethyl (*E*)-14-bromotetradec-2-enoate.

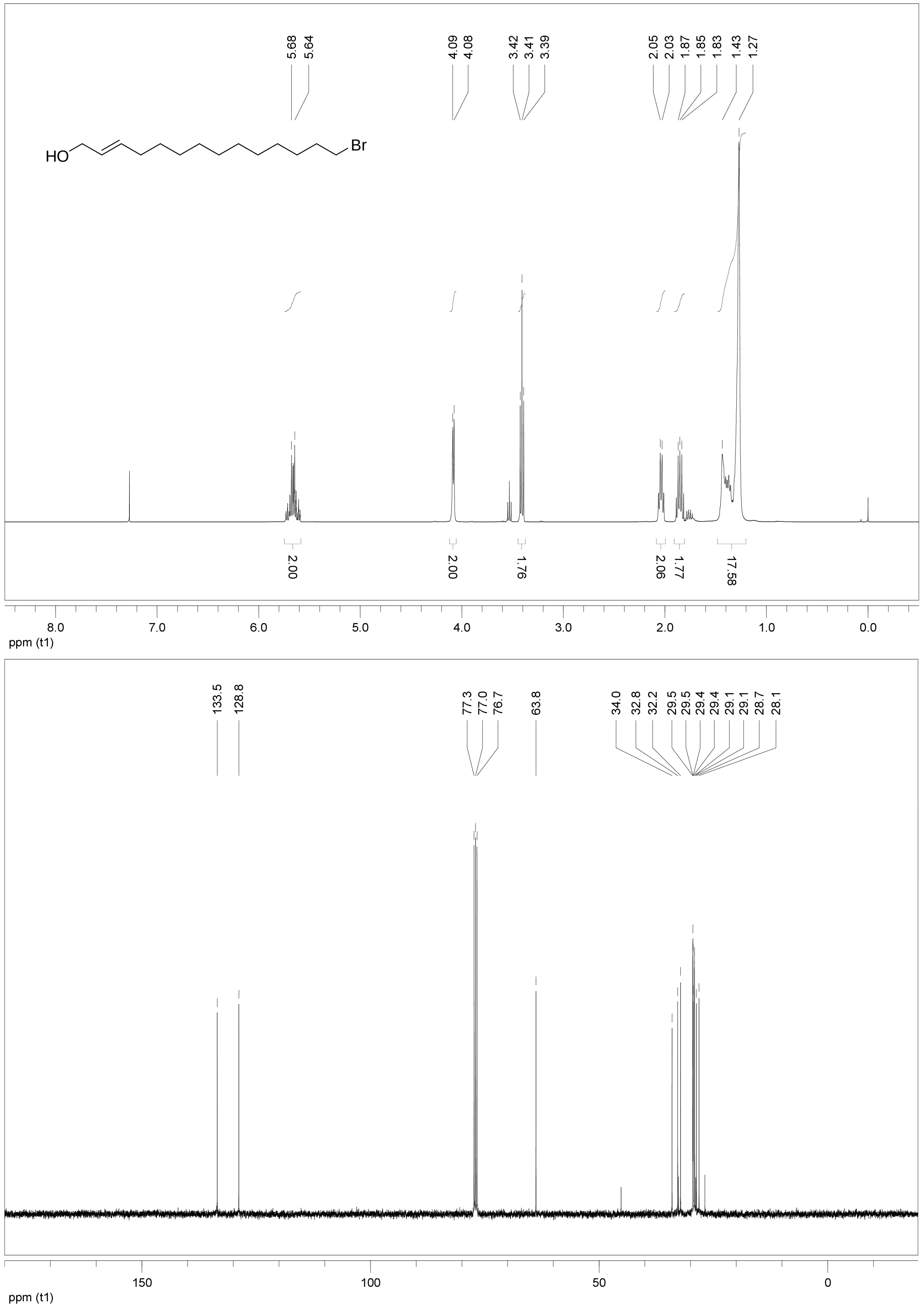

Figure S14: ^{1}H- (400 MHz) and ^{13}C-NMR (100 MHz) spectra of (*E*)-14-bromotetradec-2-en-1-ol (**3**).

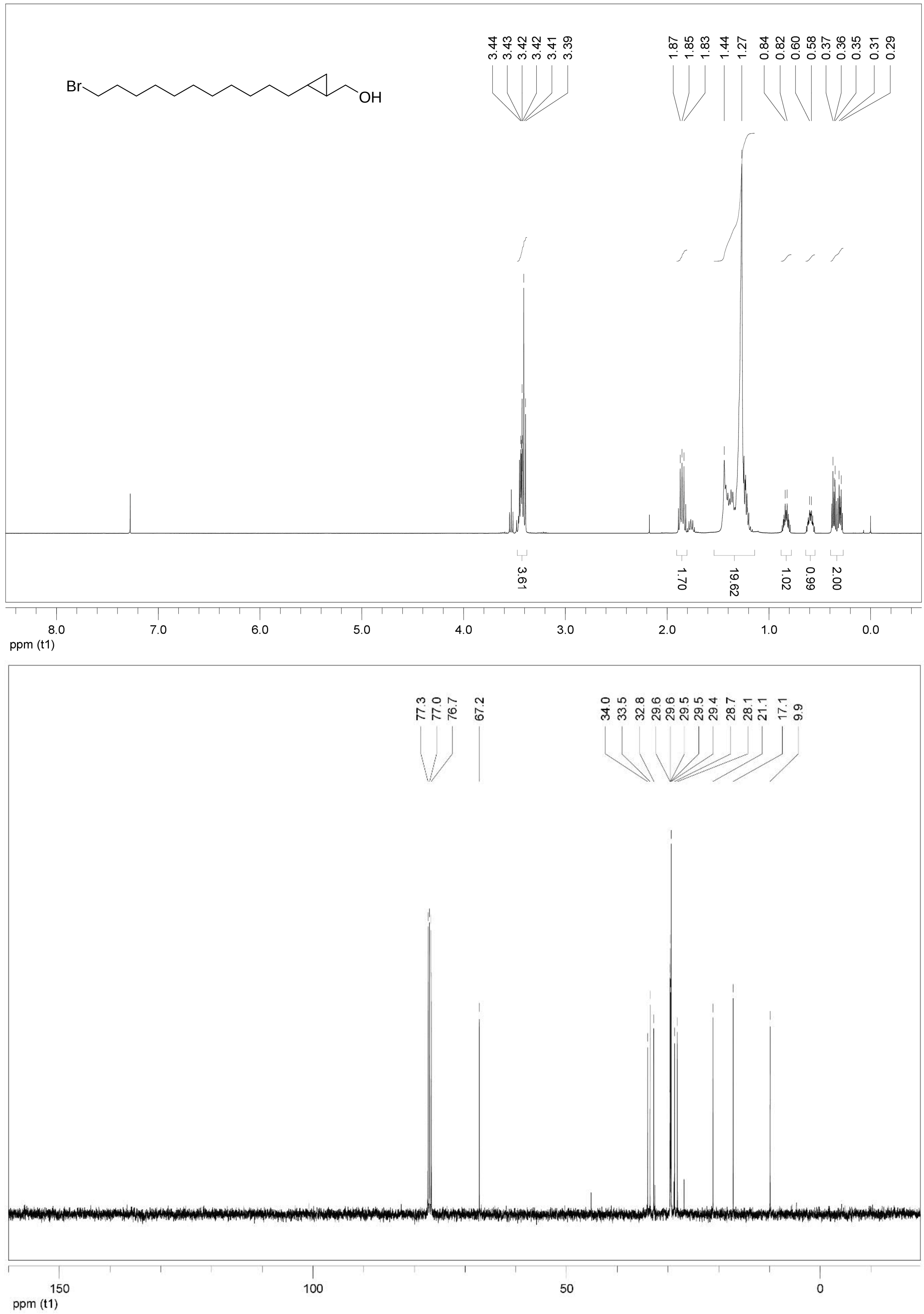

Figure S15: ^{1}H- (400 MHz) and ^{13}C-NMR (100 MHz) spectra of ((1*RS*,2*RS*)-2-(11-bromoundecyl)cyclopropyl)methanol (**4**).

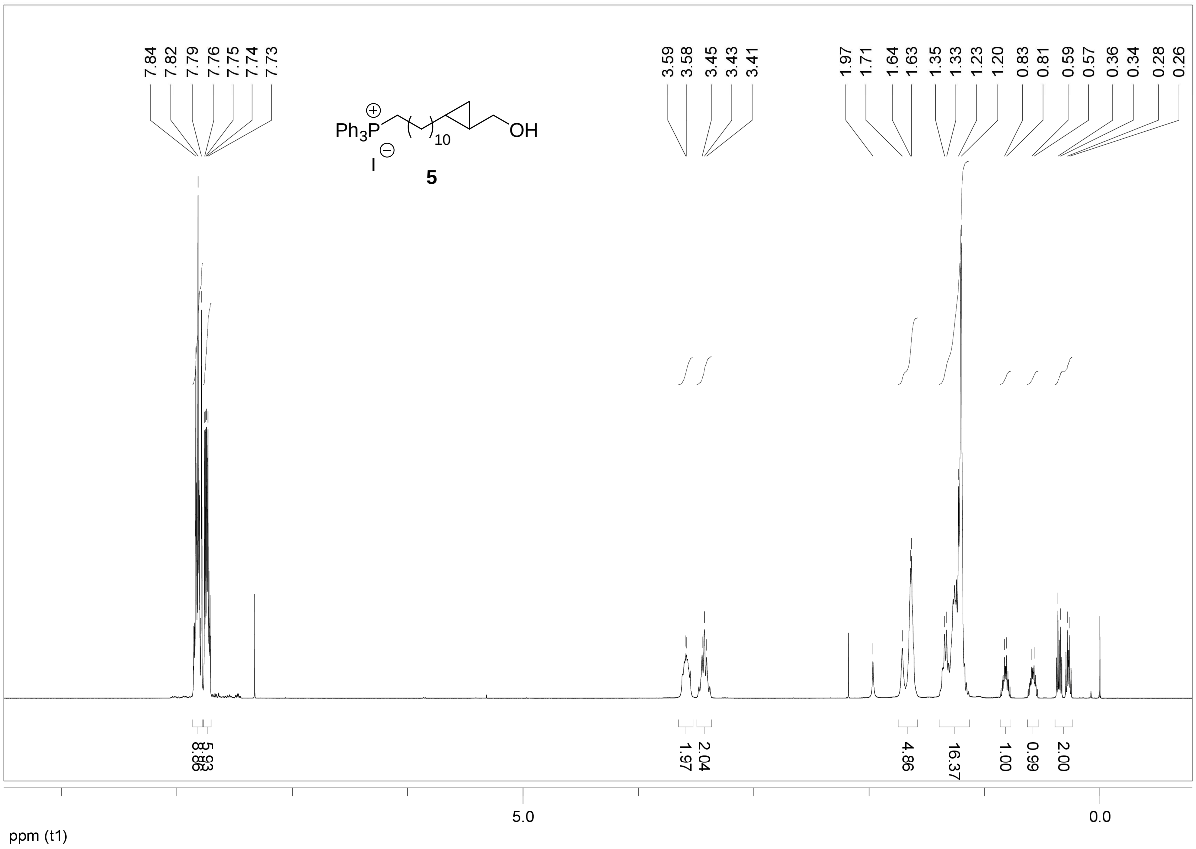

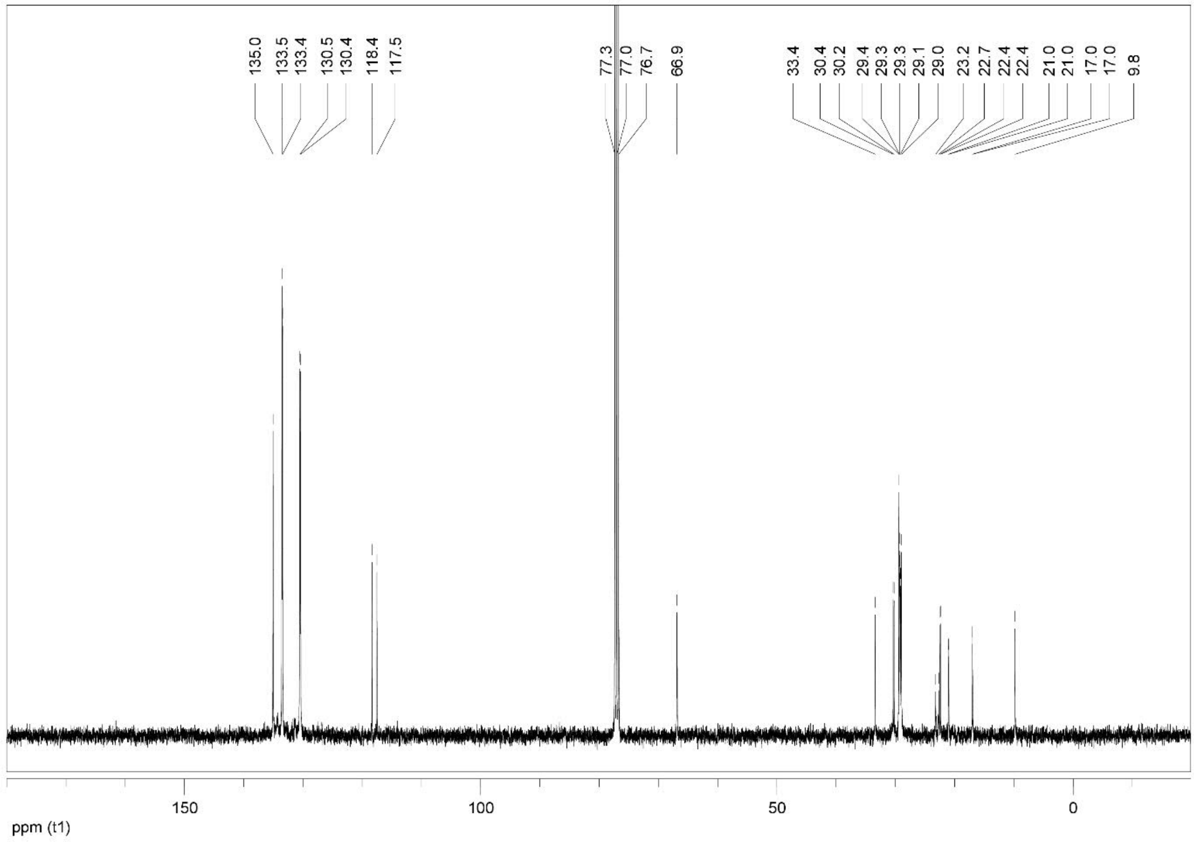

Figure S16: ^{1}H- (400 MHz) and ^{13}C-NMR (100 MHz) spectra of (11-((1*RS*,2*RS*)-2-(hydroxymethyl)cyclopropyl)undecyl)triphenylphosphonium iodide (**5**).

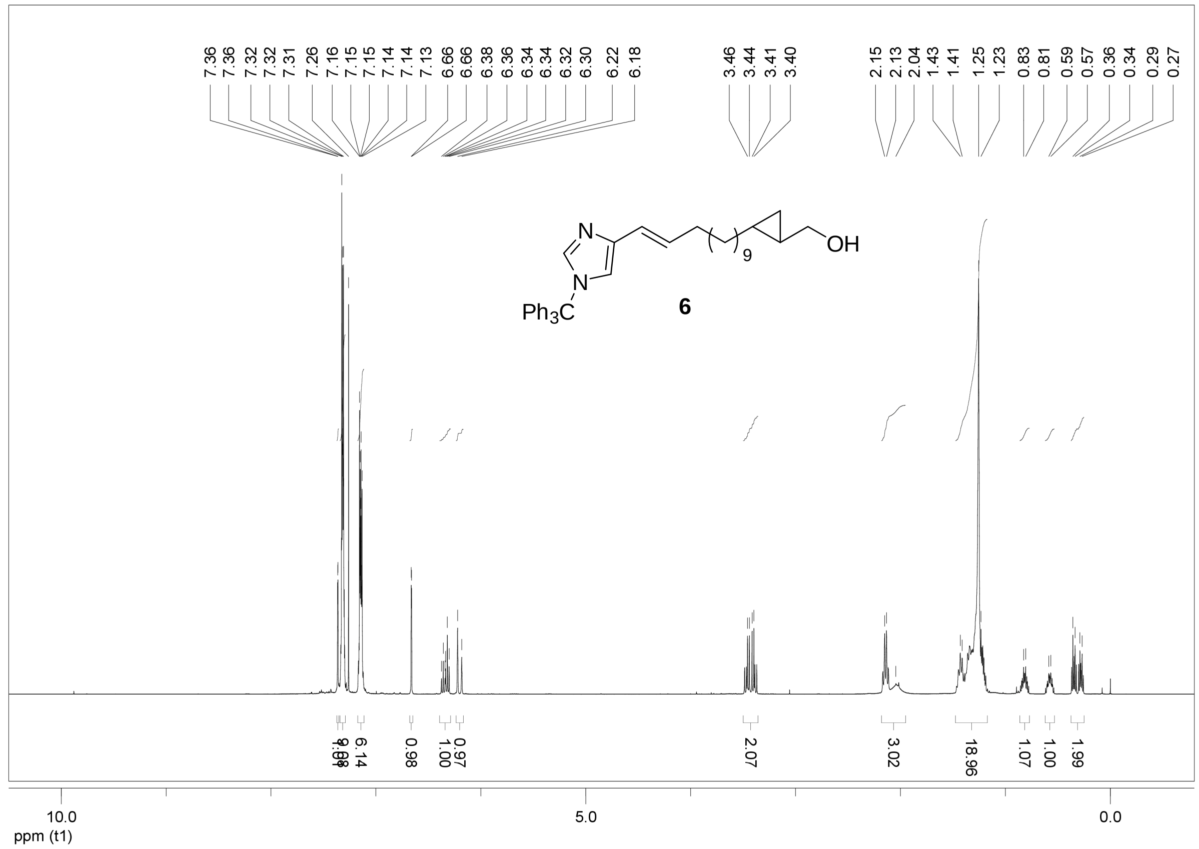

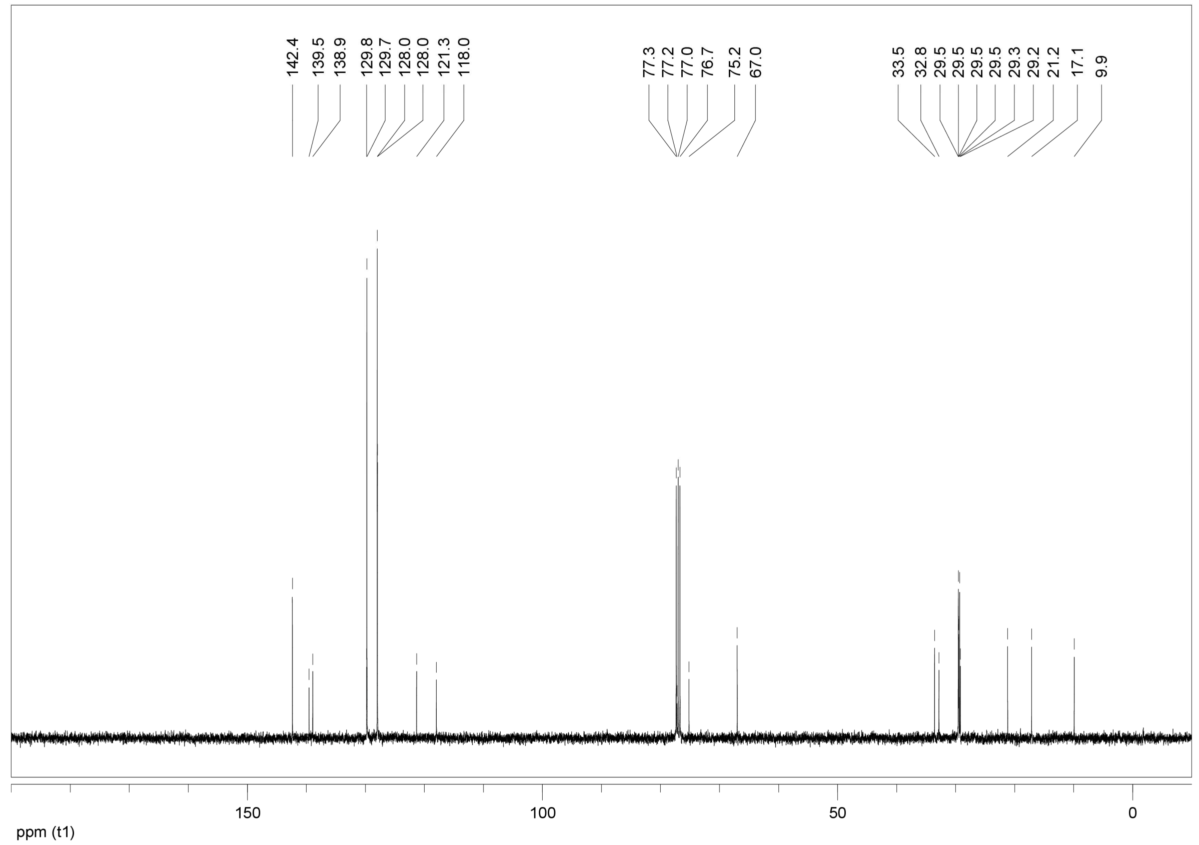

Figure S17: ^{1}H- (400 MHz) and ^{13}C-NMR (100 MHz) spectra of ((1*RS*,2*RS*)-2-((*E*)-12-(1-trityl-1*H*-imidazol-4-yl)dodec-11-en-1-yl)cyclopropyl)methanol (**6**).

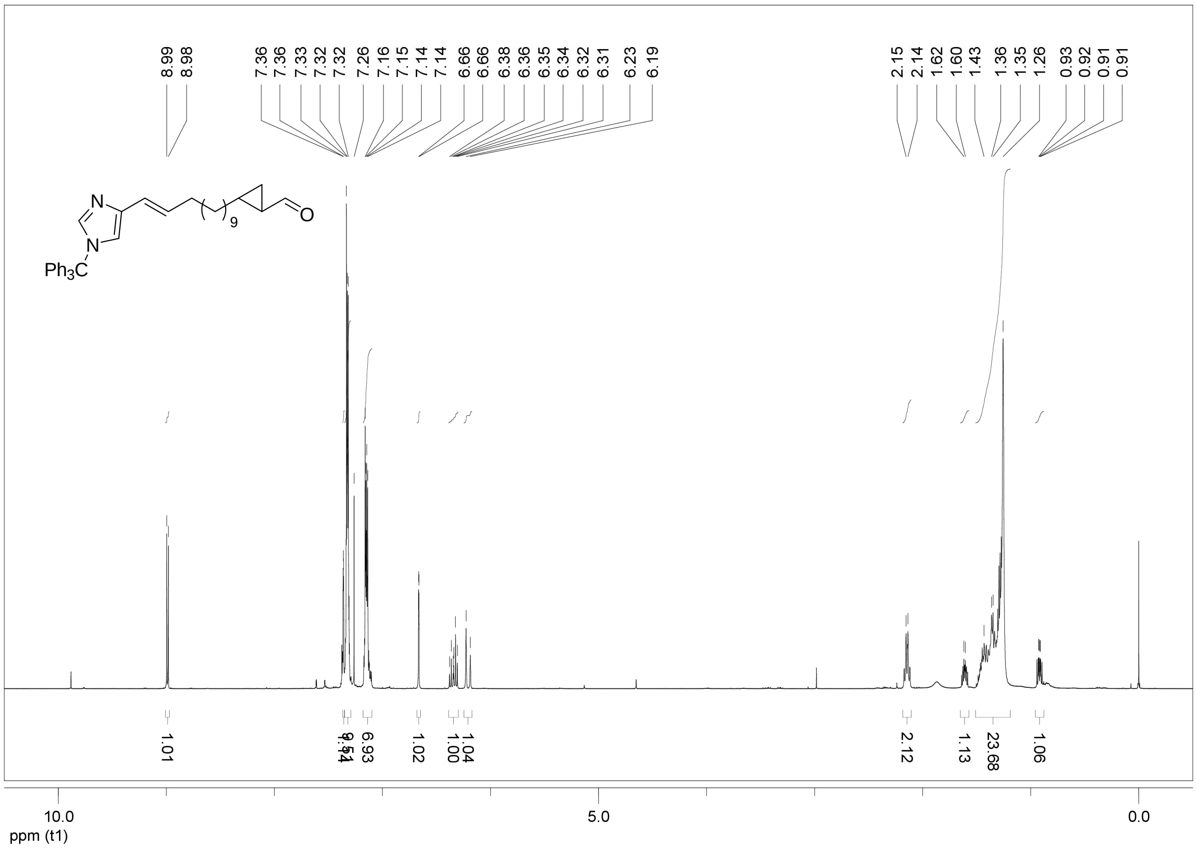

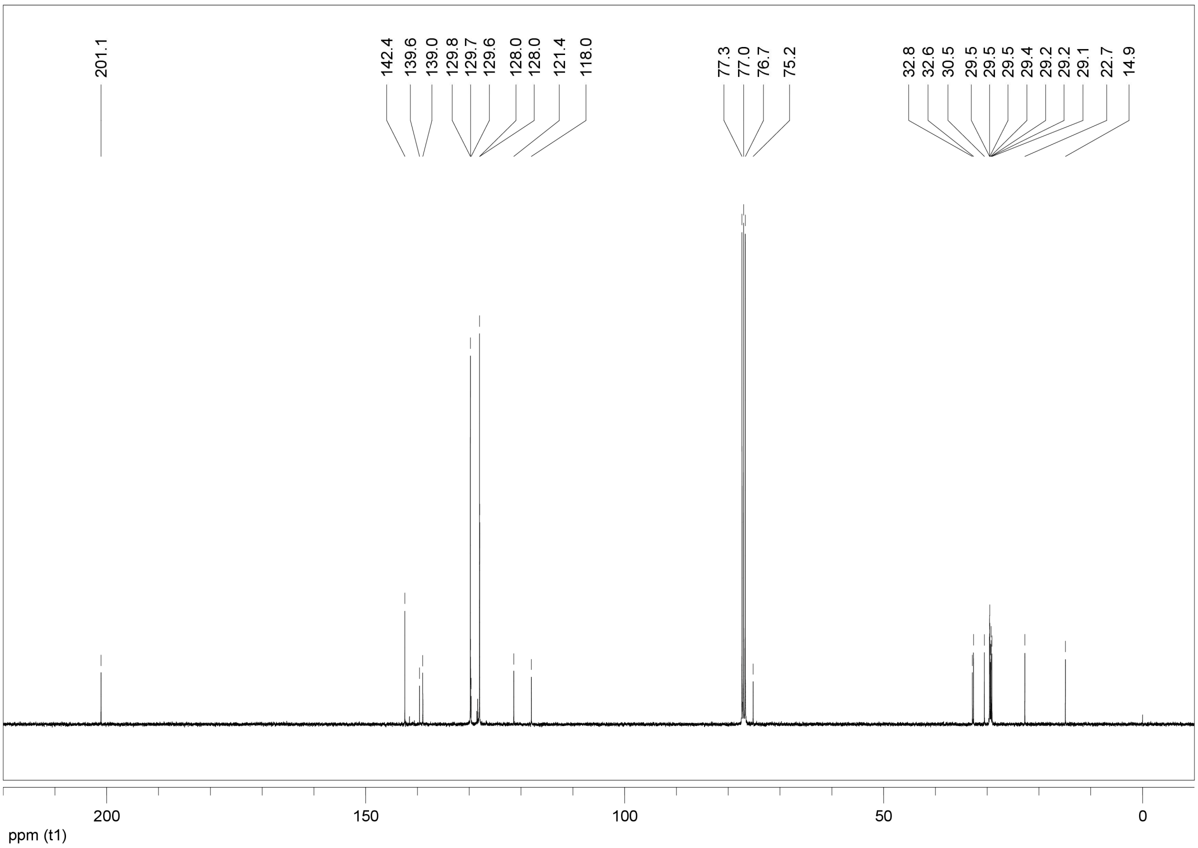

Figure S18: ^{1}H- (400 MHz) and ^{13}C-NMR (100 MHz) spectra of (1*RS*,2*RS*)-2-((*E*)-12-(1-trityl-1*H*-imidazol-4-yl)dodec-11-en-1-yl)cyclopropane-1-carbaldehyde.

Synthesis of imidacin *cis*-A1

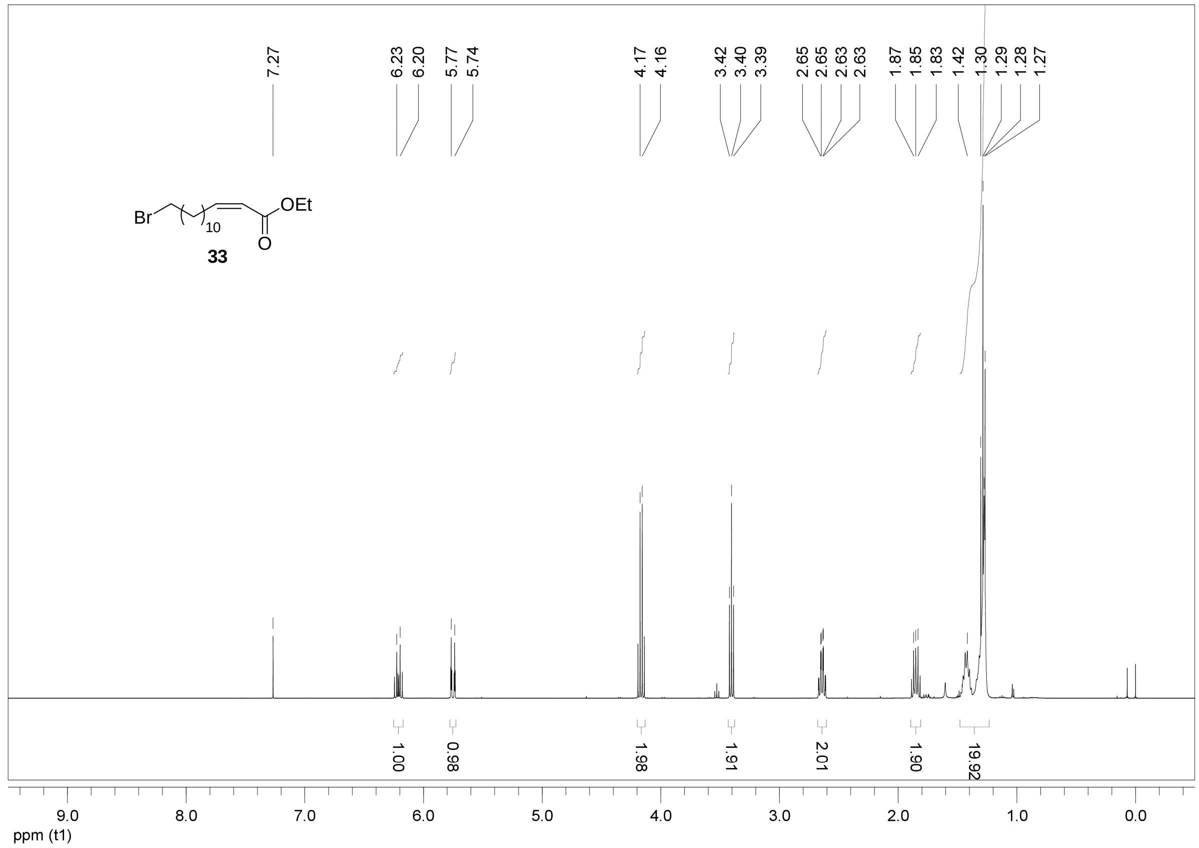

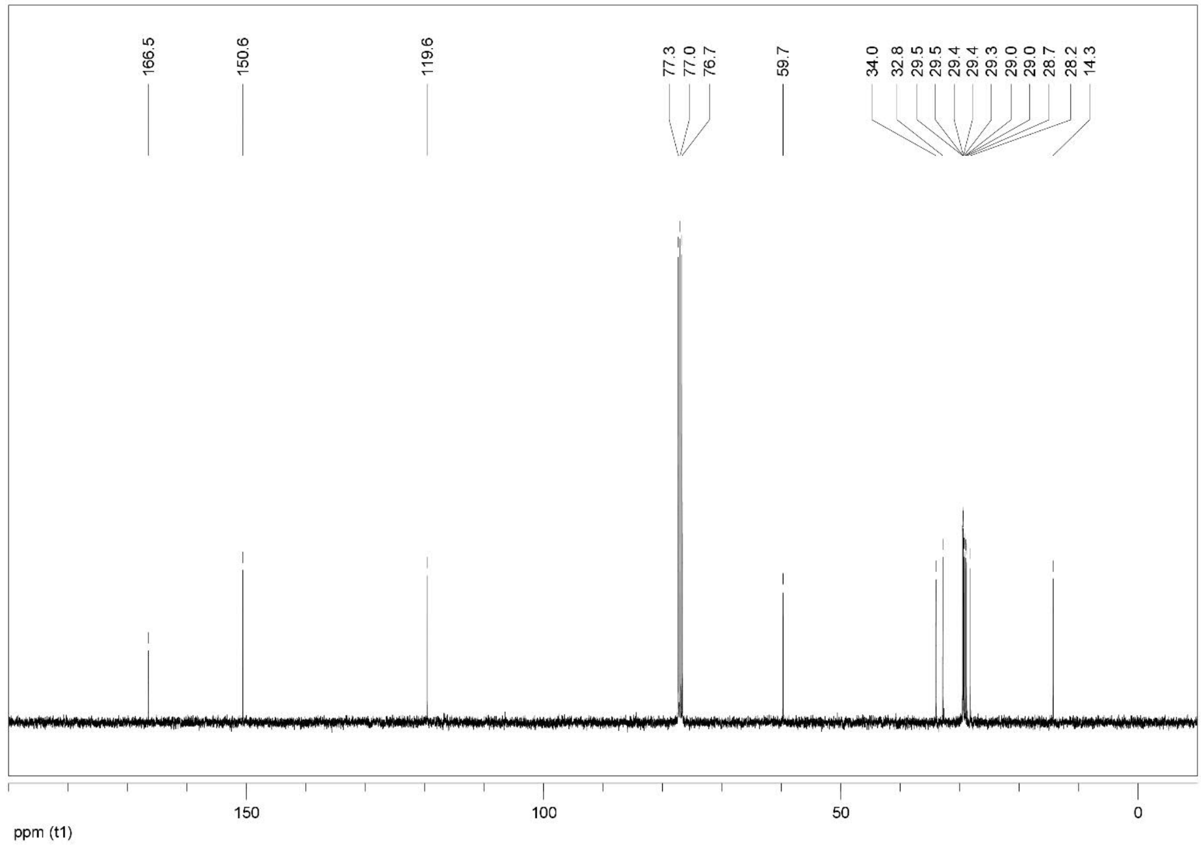

Figure S19: ^{1}H- (400 MHz) and ^{13}C-NMR (100 MHz) spectra of ethyl (*Z*)-14-bromotetradec-2-enoate (**33**).

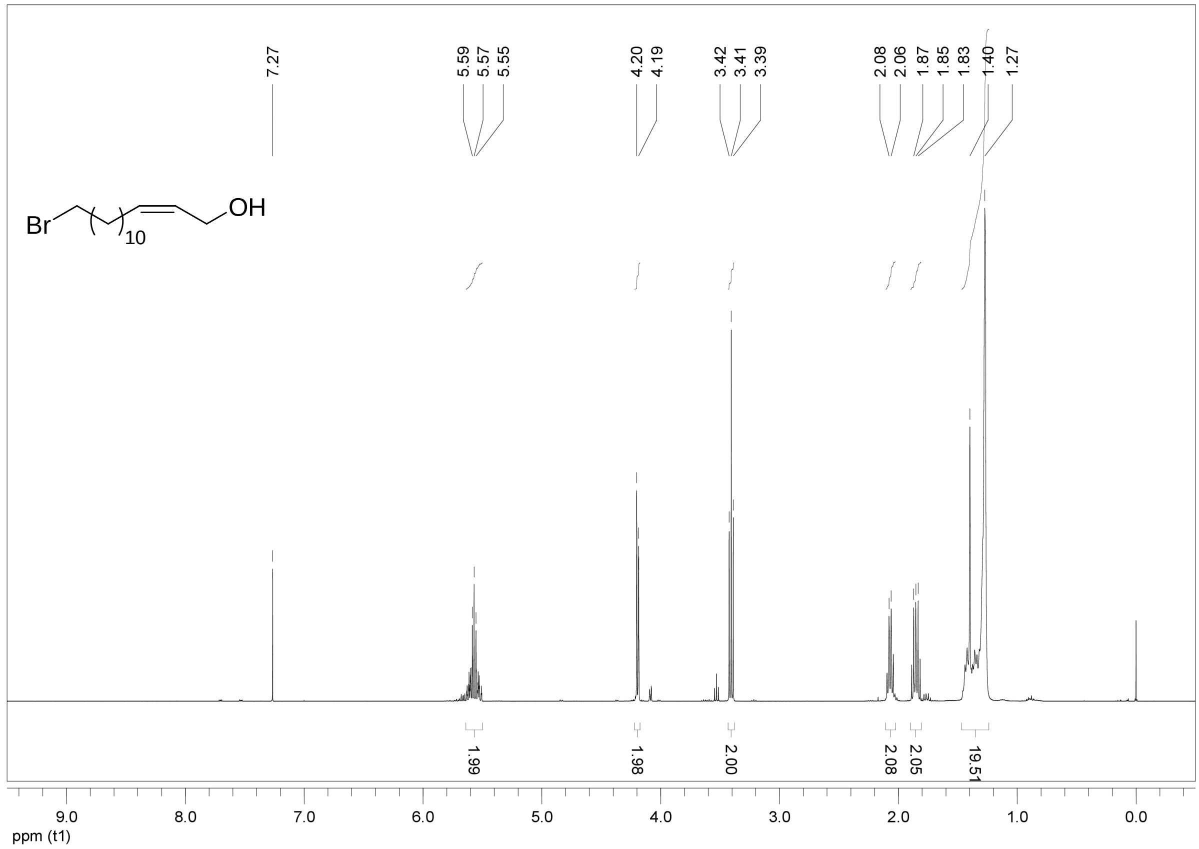

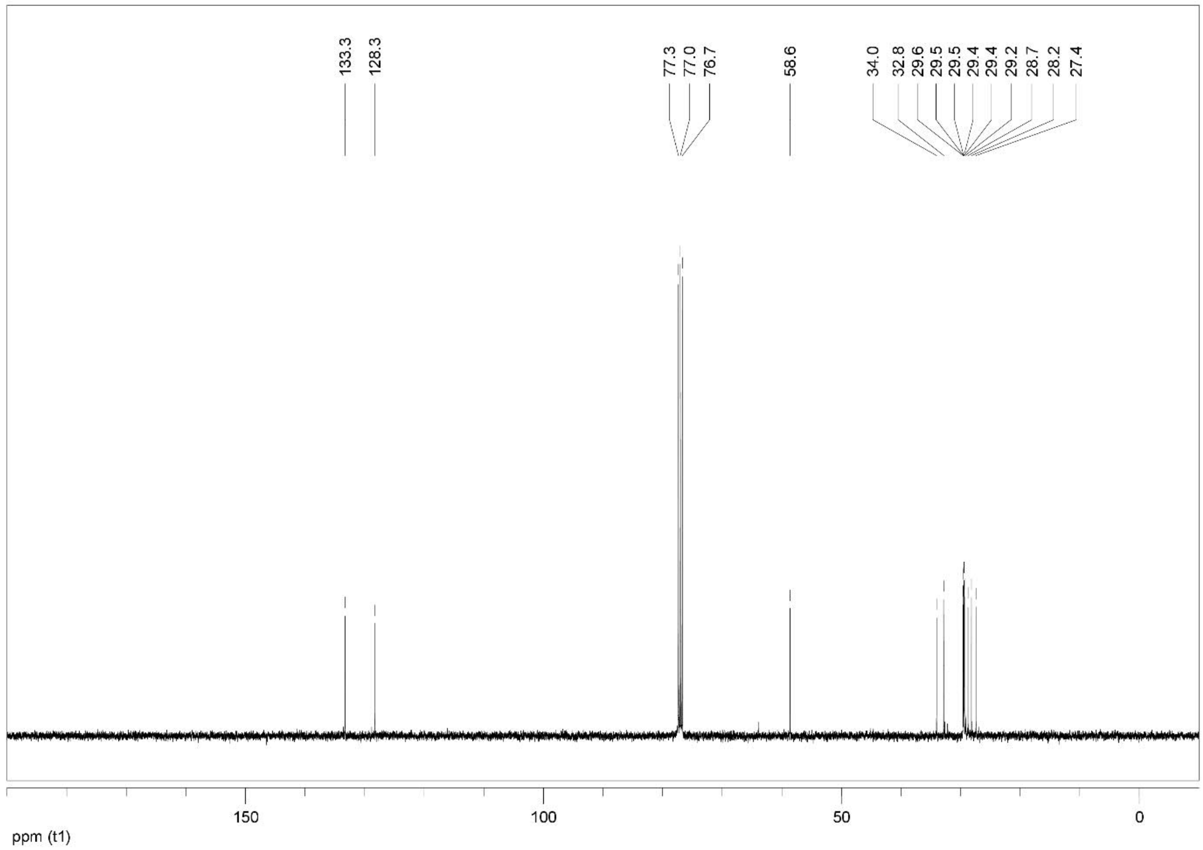

Figure S20: ^{1}H- (400 MHz) and ^{13}C-NMR (100 MHz) spectra of (*Z*)-14-bromotetradec-2-en-1-ol.

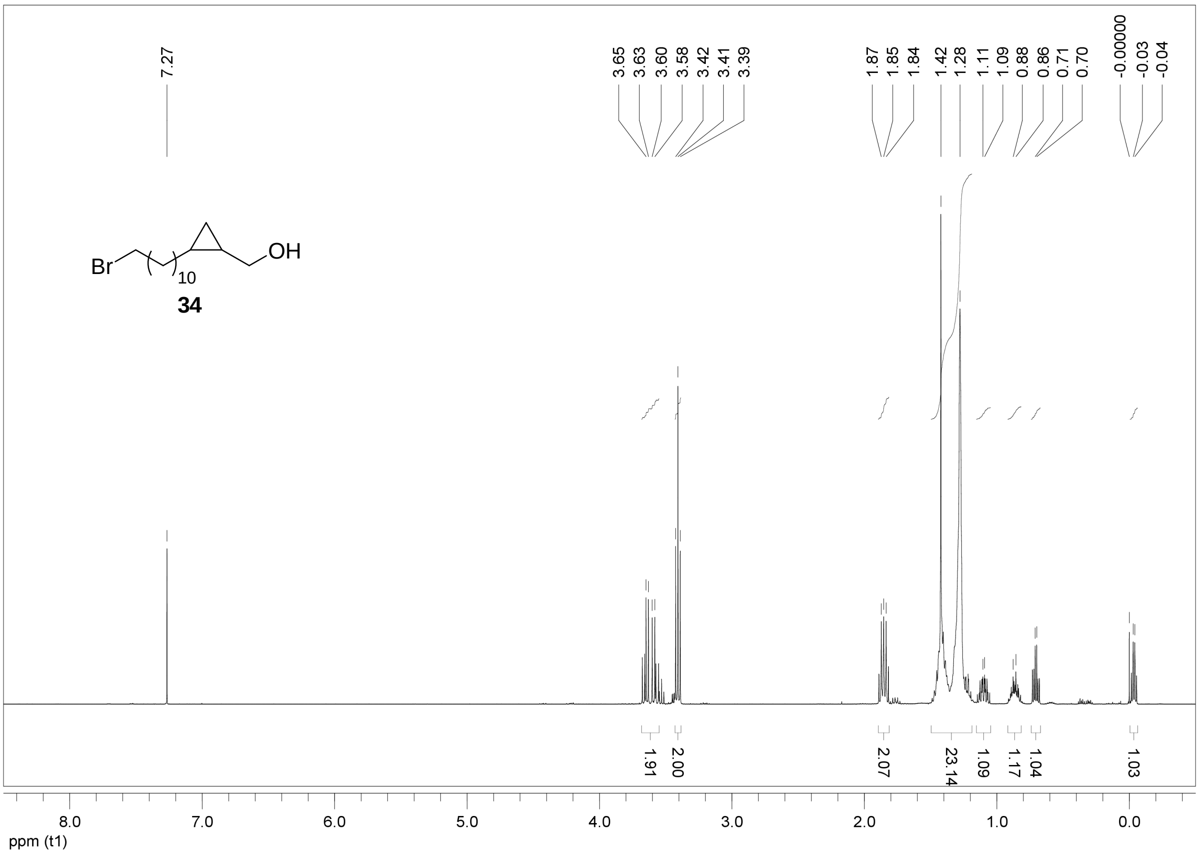

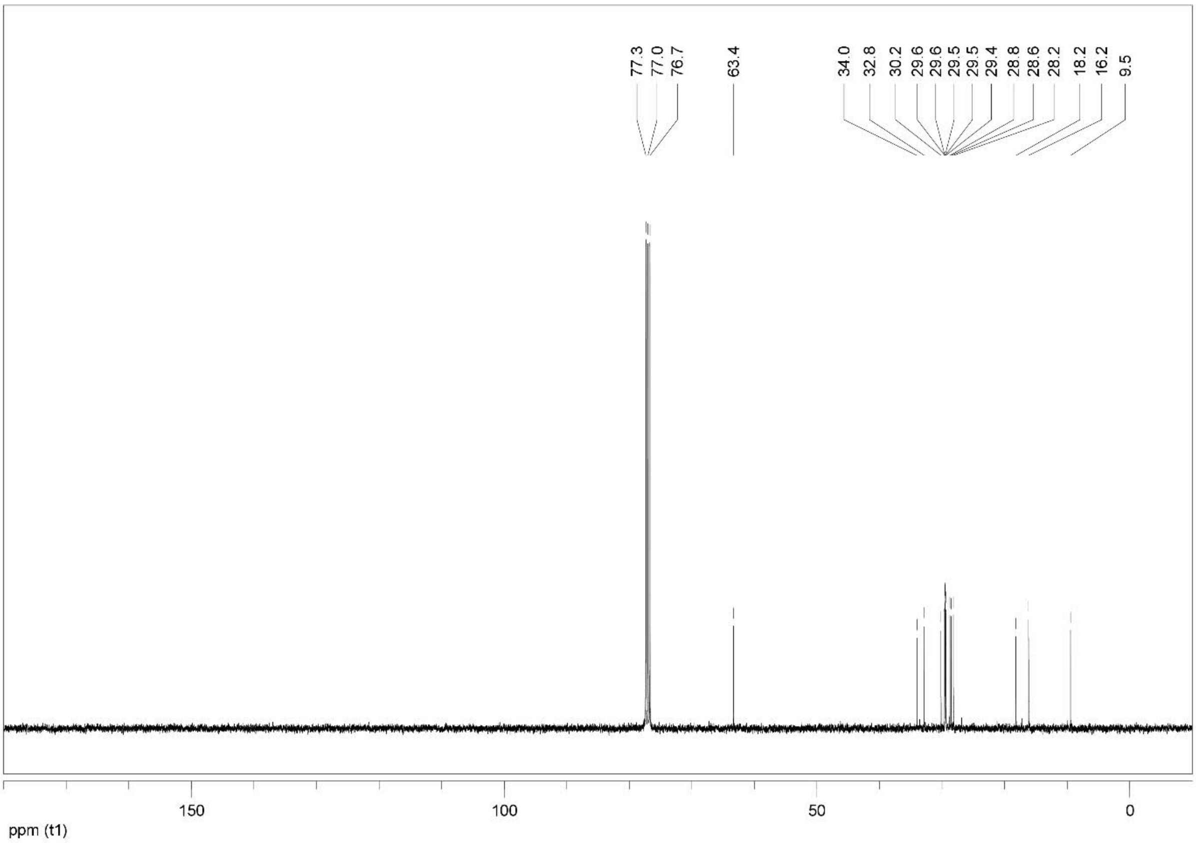

Figure S21: ^{1}H- (400 MHz) and ^{13}C-NMR (100 MHz) spectra of ((1*RS*,2*SR*)-2-(11-bromoundecyl)cyclopropyl)methanol (**34**).

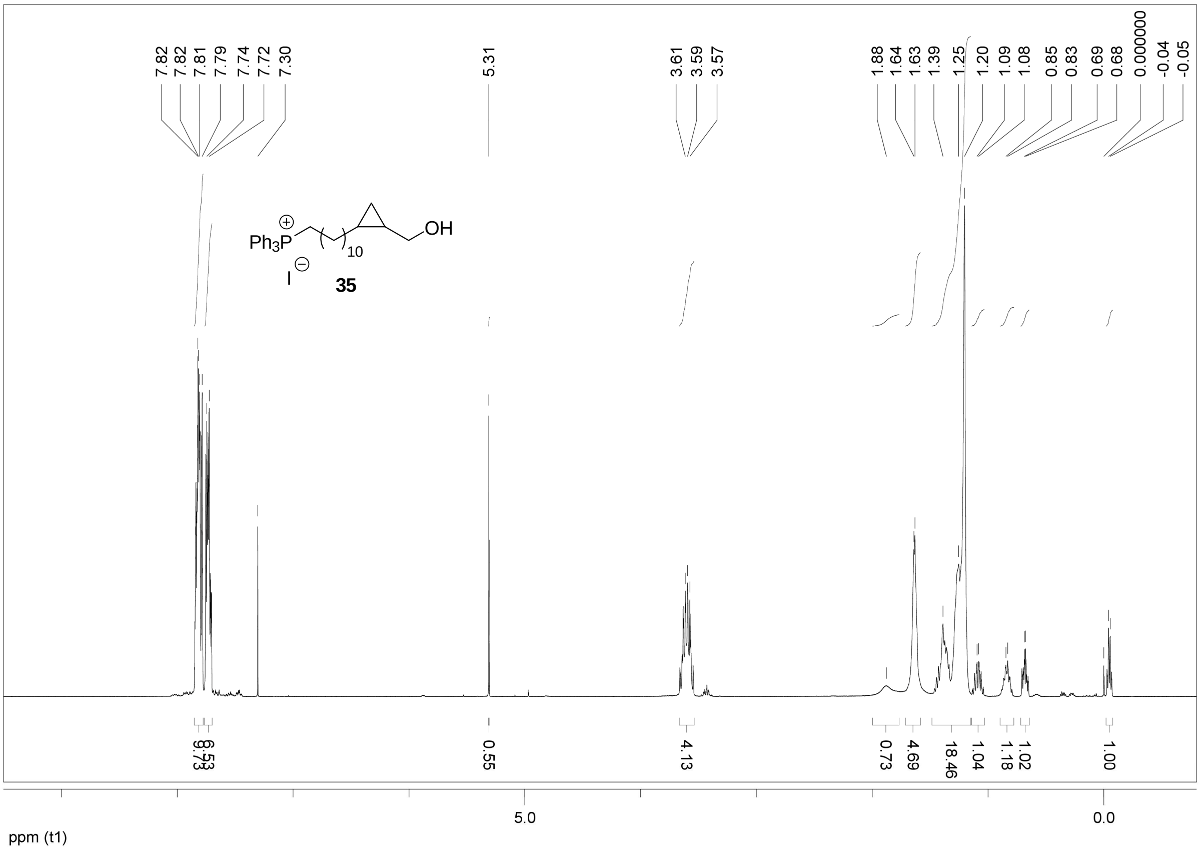

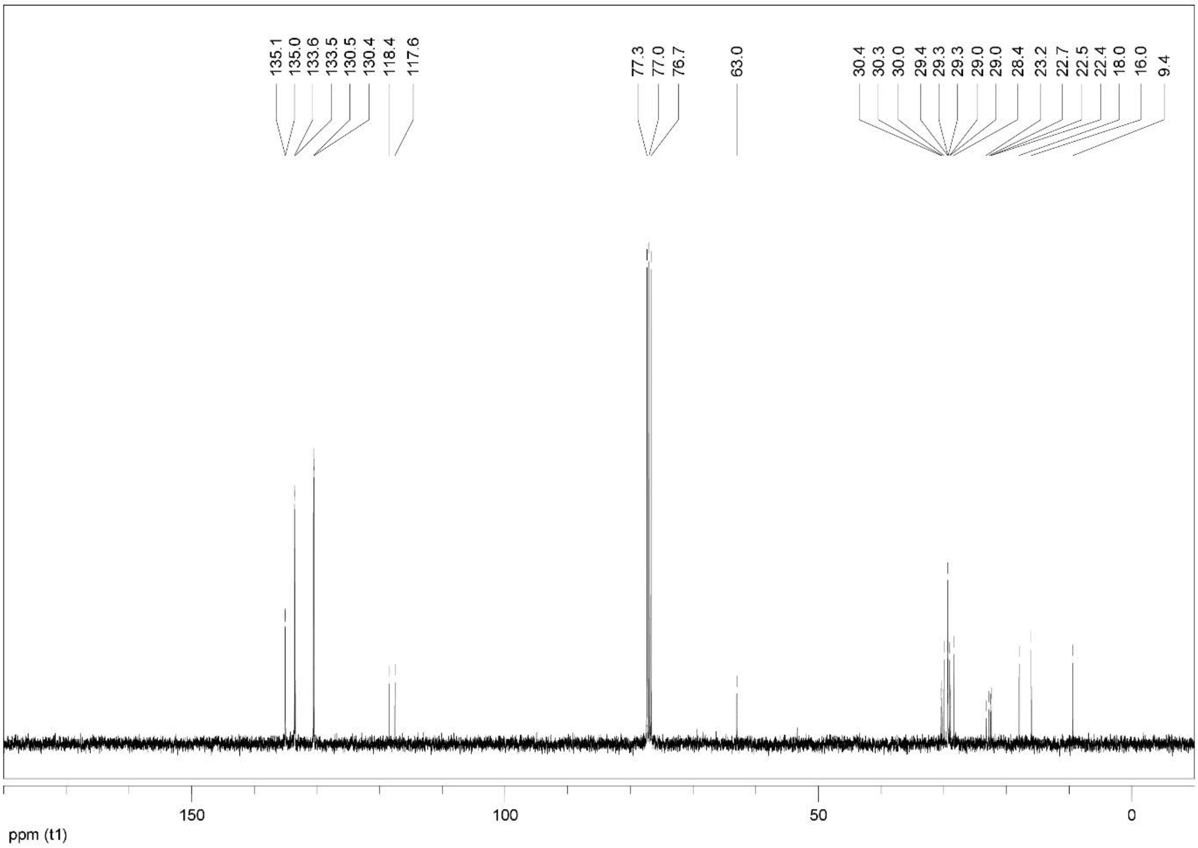

Figure S22: ^{1}H- (400 MHz) and ^{13}C-NMR (100 MHz) spectra of (11-((1*RS*,2*SR*)-2-(Hydroxymethyl)cyclopropyl)undecyl)triphenylphosphonium iodide (**35**).

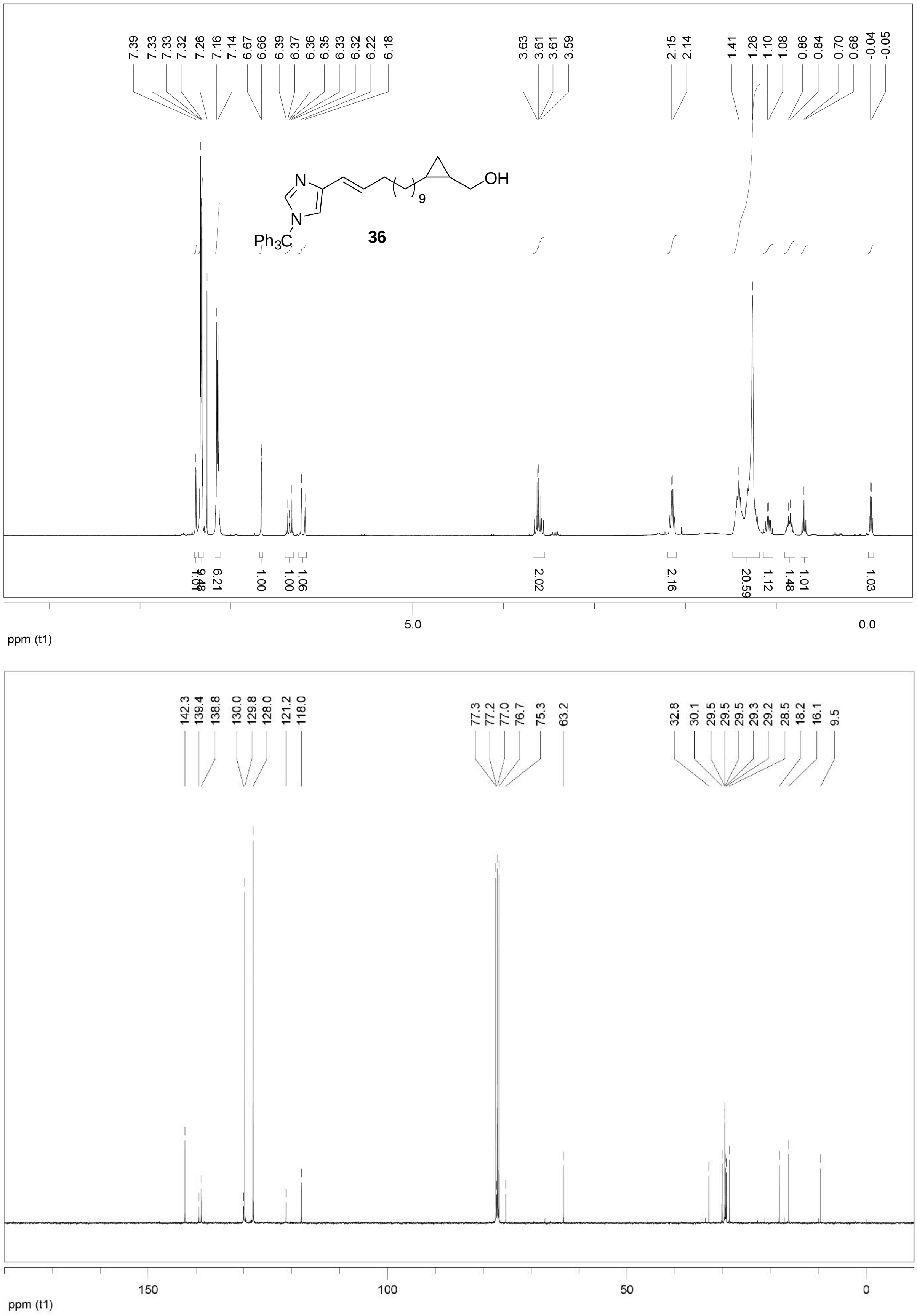

Figure S23: ^{1}H- (400 MHz) and ^{13}C-NMR (100 MHz) spectra of ((1*RS*,2*SR*)-2-((*E*)-12-(1-trityl-1*H*-imidazol-4-yl)dodec-11-en-1-yl)cyclopropyl)-methanol (**36**).

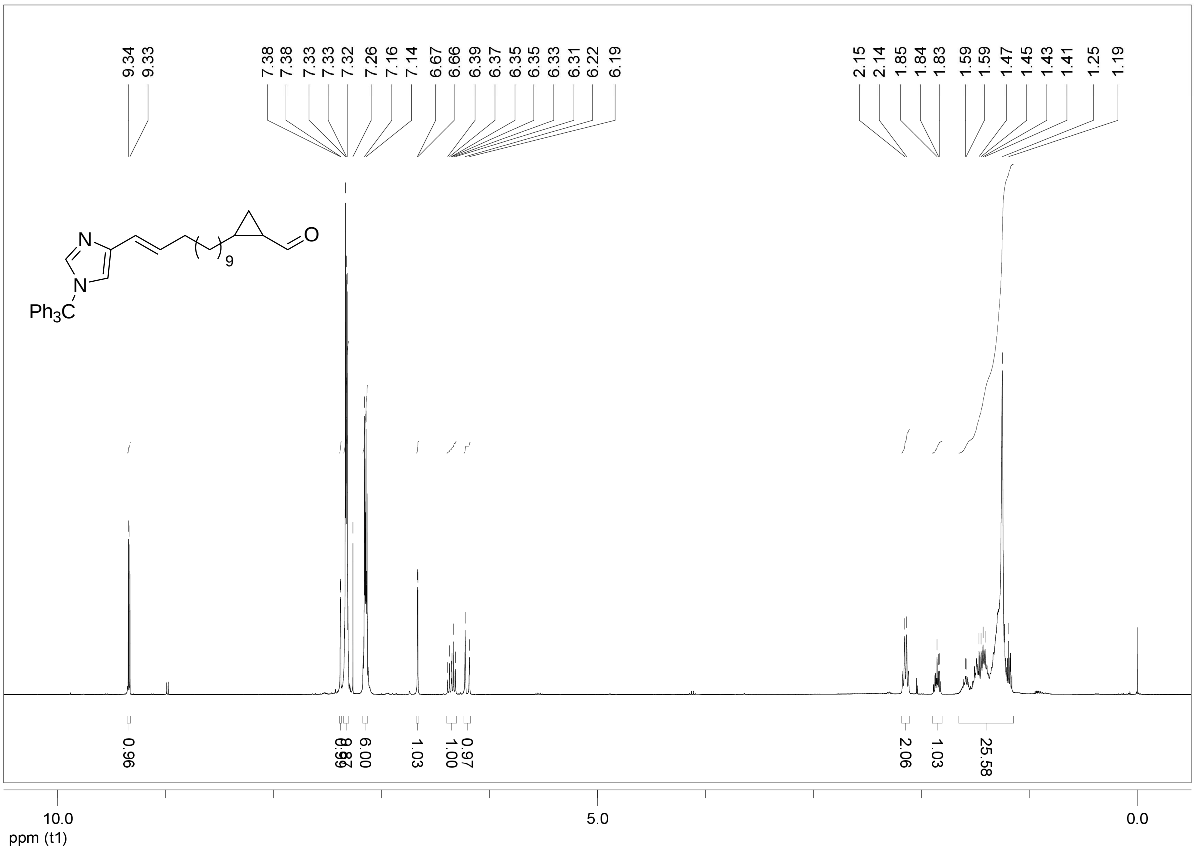

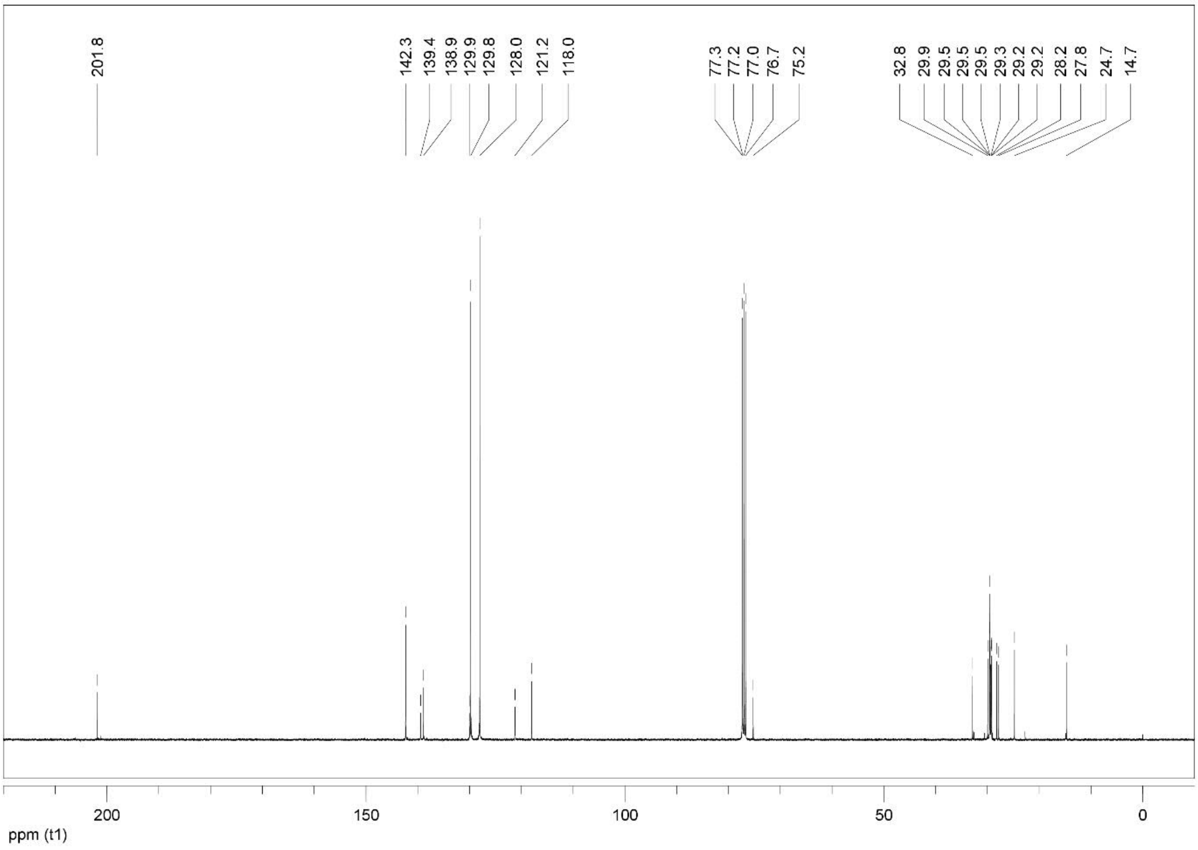

Figure S24: ^{1}H- (400 MHz) and ^{13}C-NMR (100 MHz) spectra of (1*RS*,2*SR*)-2-((*E*)-12-(1-trityl-1*H*-imidazol-4-yl)dodec-11-en-1-yl)cyclopropane-1-carbaldehyde.

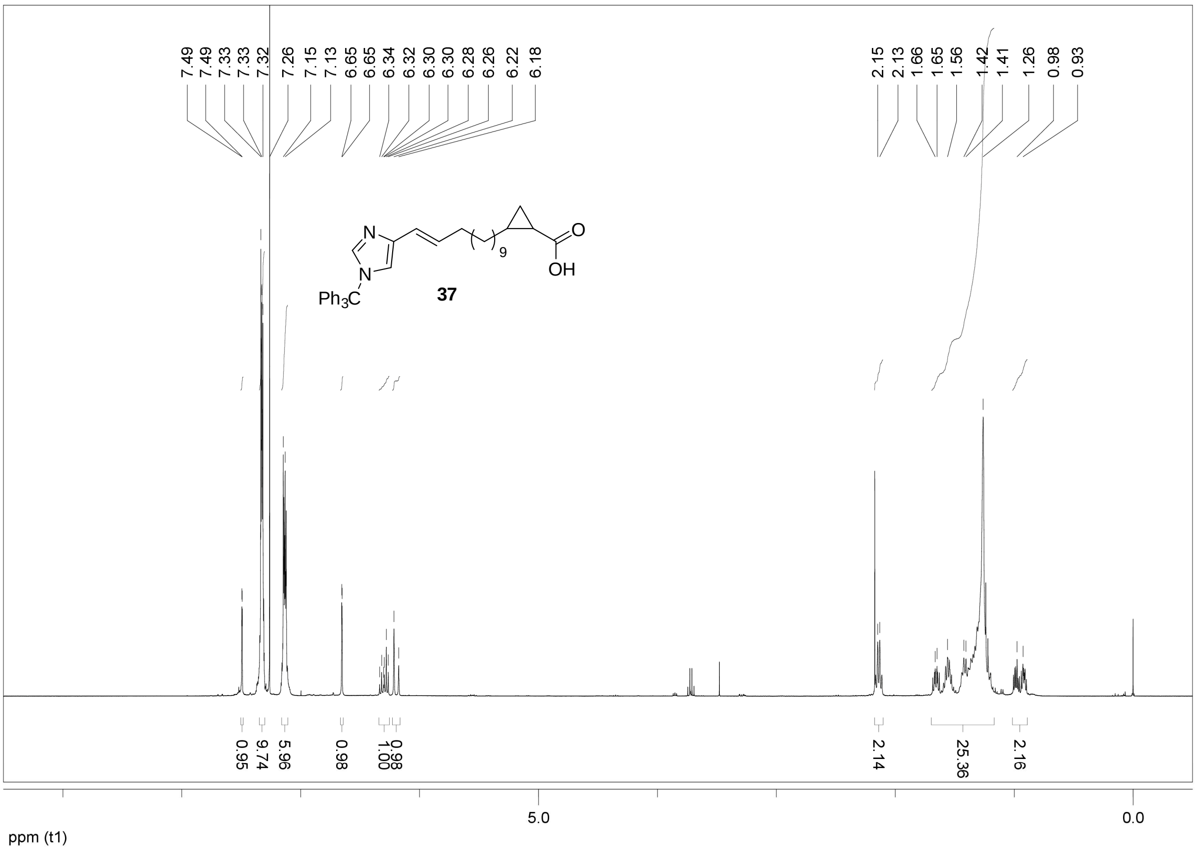

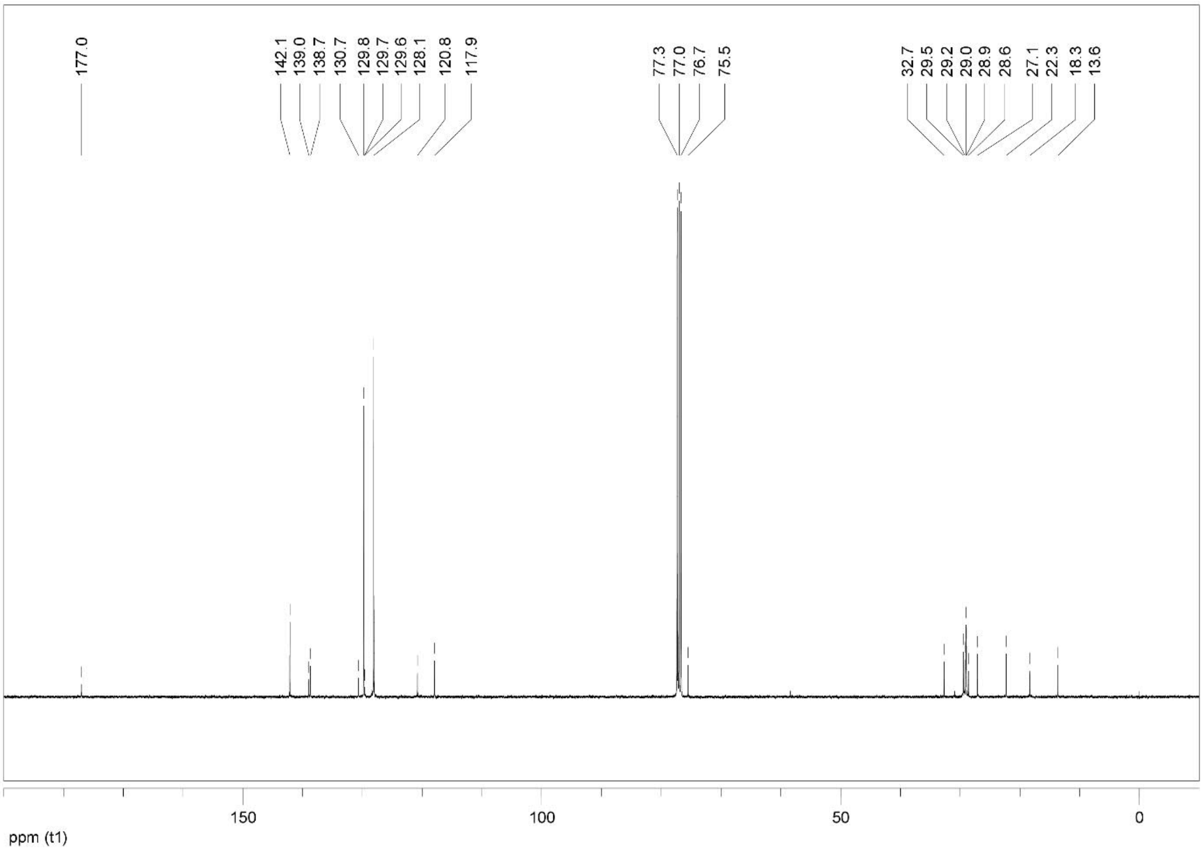

Figure S25: ^{1}H- (400 MHz) and ^{13}C-NMR (100 MHz) spectra of (1*RS*,2*SR*)-2-((*E*)-12-(1-trityl-1*H*-imidazol-4-yl)dodec-11-en-1-yl)cyclopropane-1-carboxylic acid (**37**).

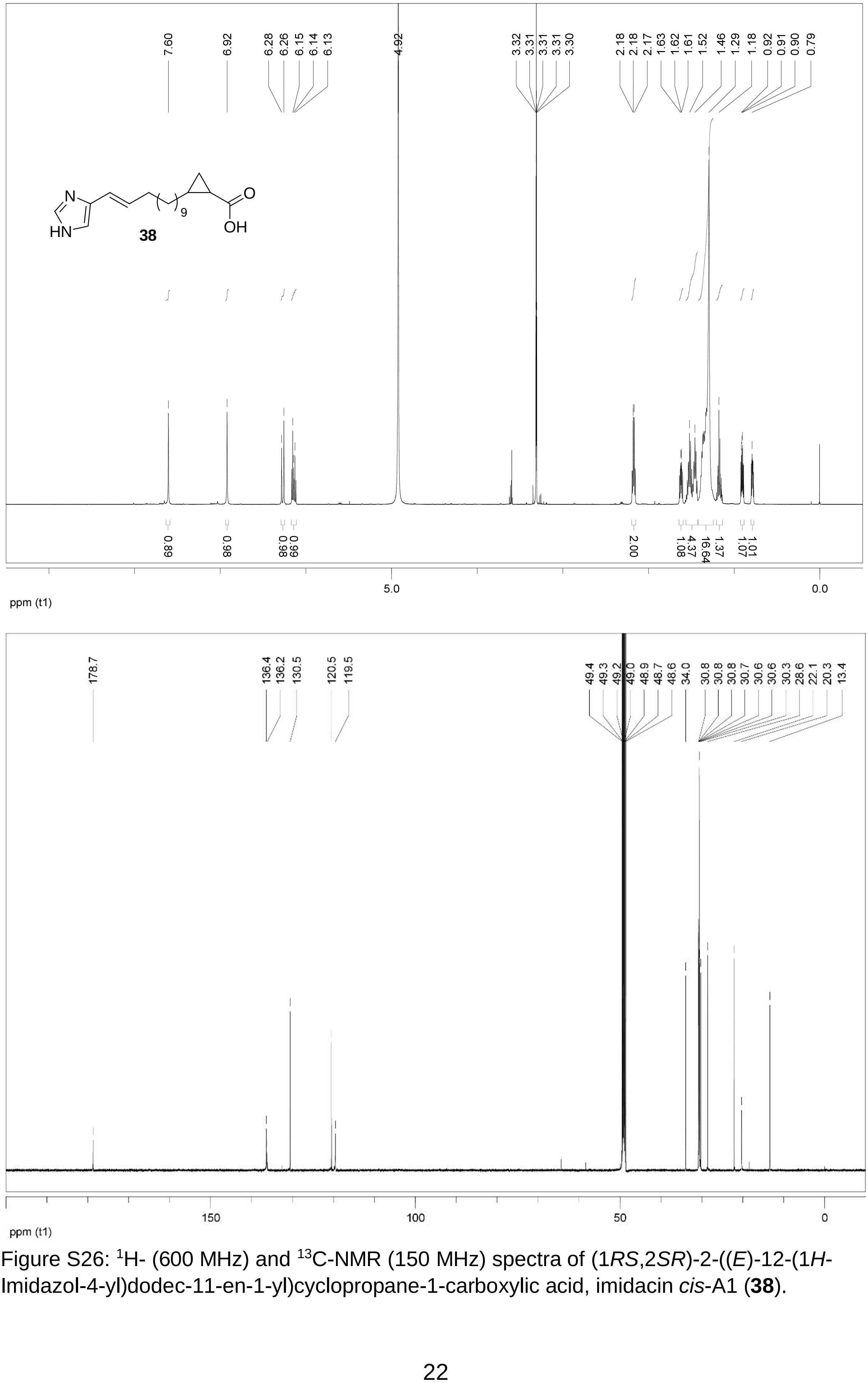

Figure S26: ^{1}H- (600 MHz) and ^{13}C-NMR (150 MHz) spectra of (1*RS*,2*SR*)-2-((*E*)-12-(1*H*-Imidazol-4-yl)dodec-11-en-1-yl)cyclopropane-1-carboxylic acid, imidacin *cis*-A1 (**38**).

Enantioselective synthesis of imidacin A1

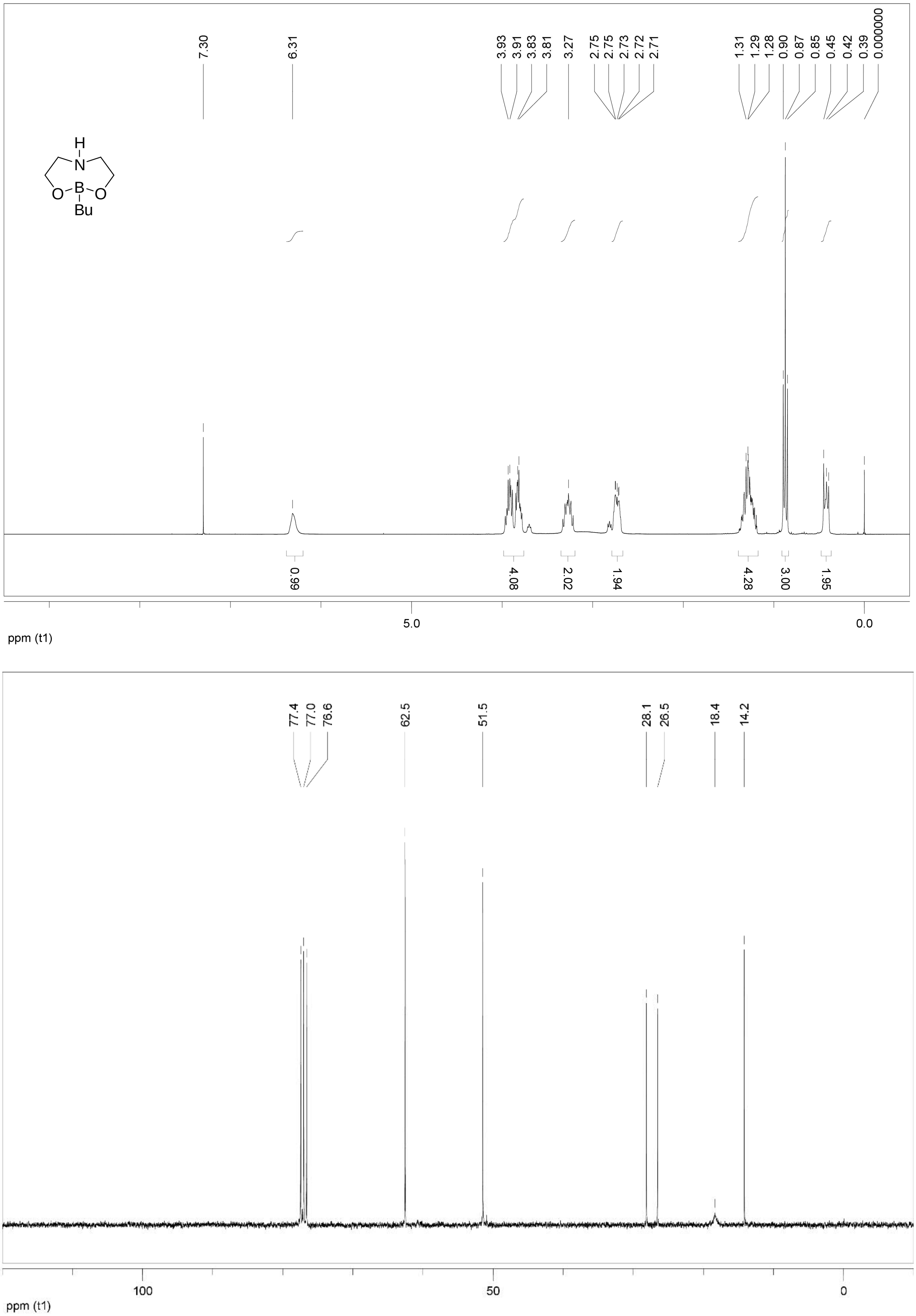

Figure S27: ^{1}H- (300 MHz) and ^{13}C-NMR (75 MHz) spectra of 2-butyl-1,3,6,2-dioxazaborocane.

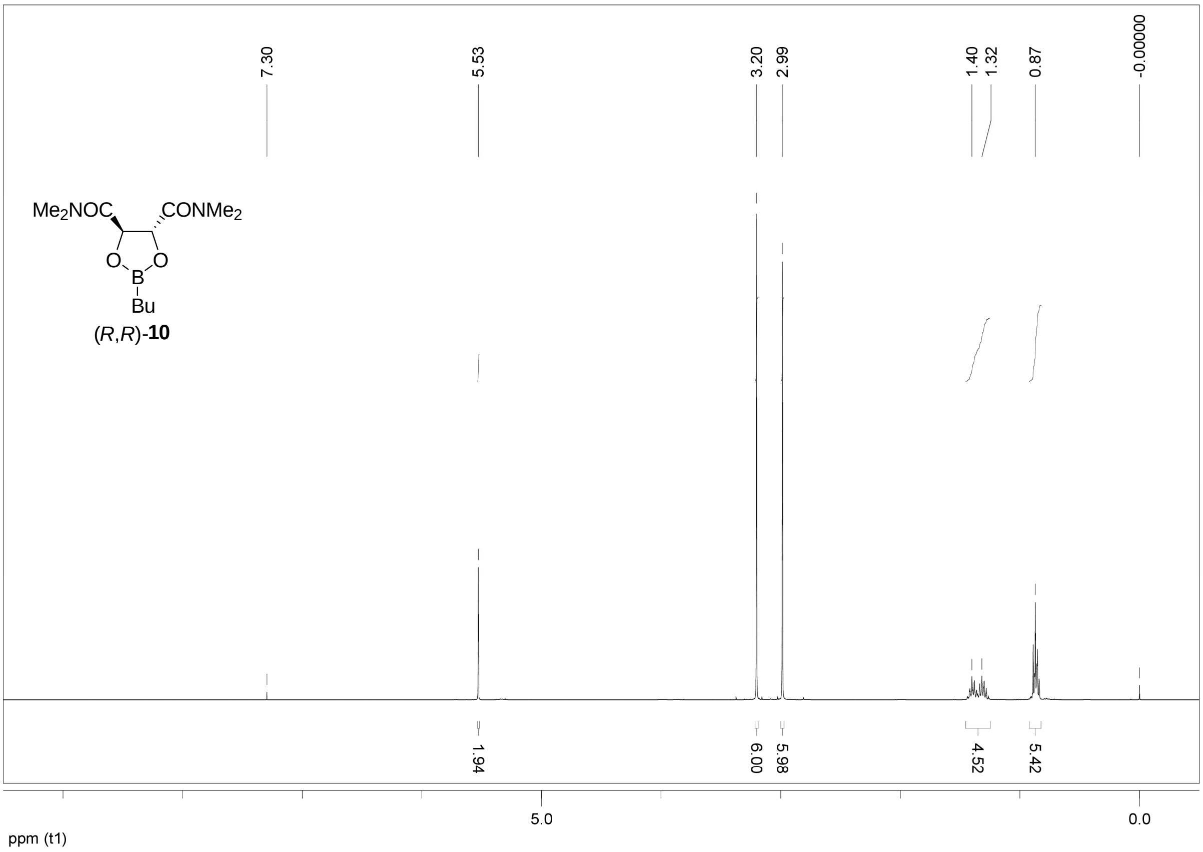

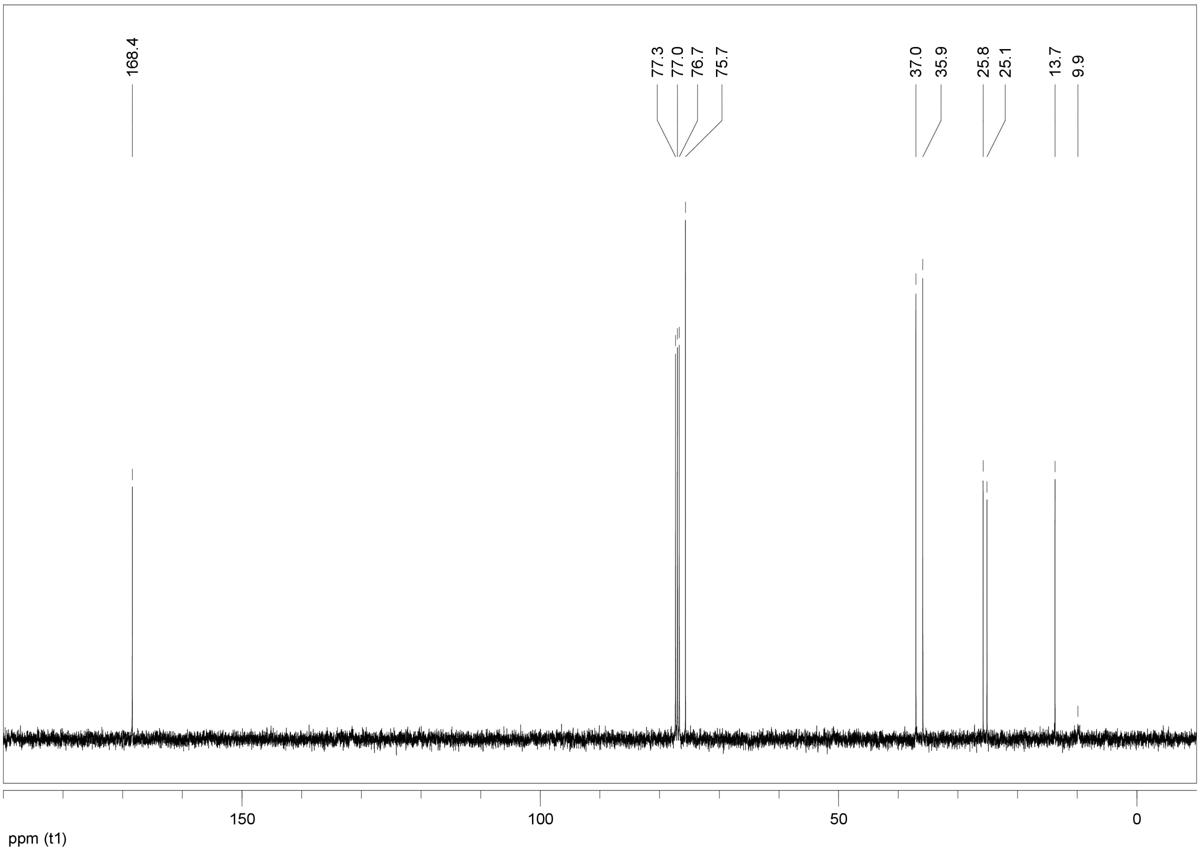

Figure S28: ^{1}H- (400 MHz) and ^{13}C-NMR (100 MHz) spectra of (4*R*,5*R*)-2-butyl-*N*[4],*N*[4],*N*[5],*N*[5]-tetramethyl-1,3,2-dioxaborolane-4,5-dicarboxamide ((*R*,*R*)-**10**).

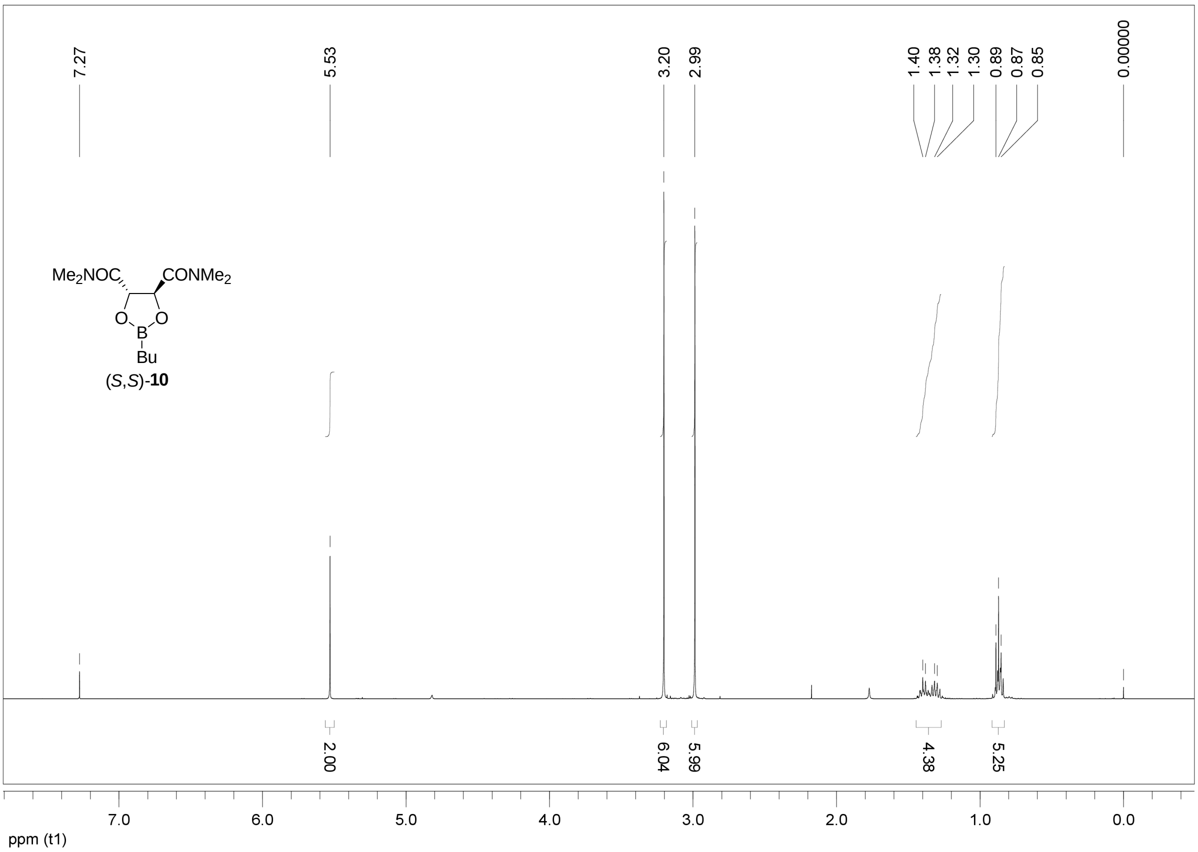

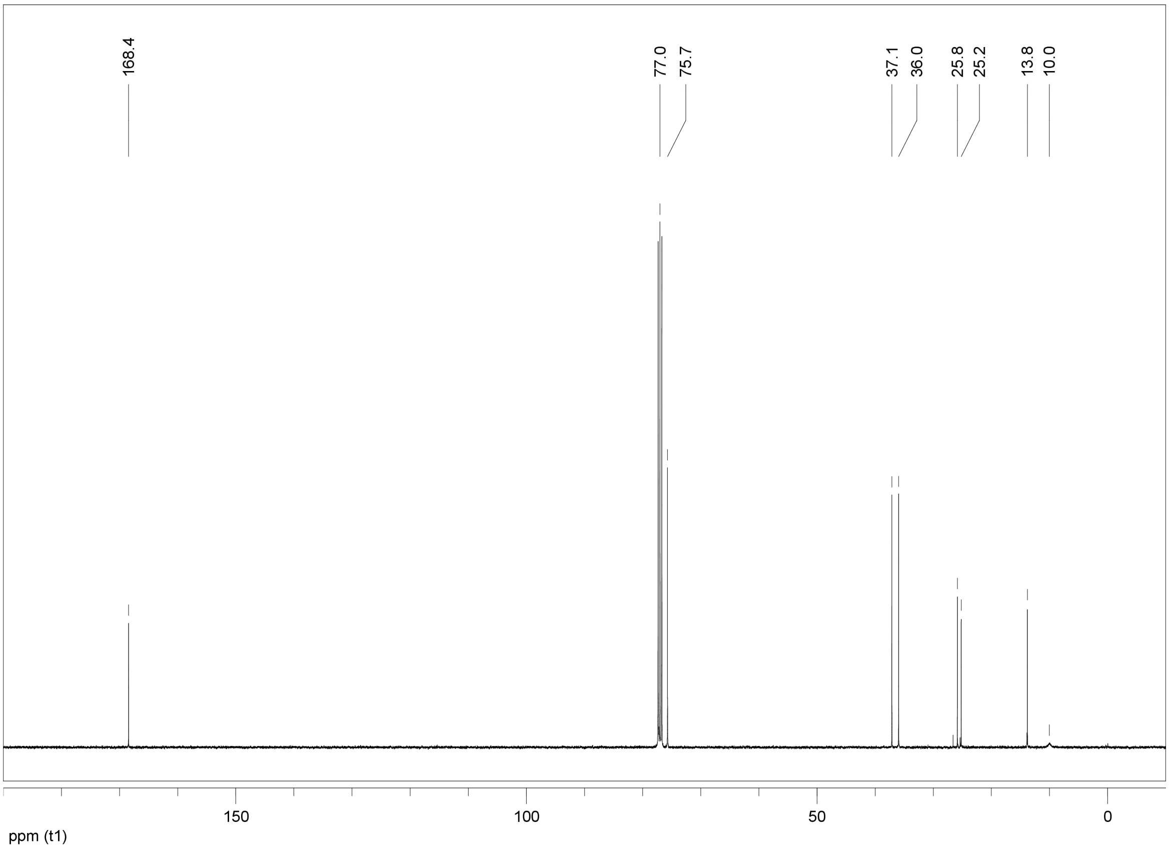

Figure S29: ^{1}H- (400 MHz) and ^{13}C-NMR (100 MHz) spectra of (4*S*,5*S*)-2-Butyl-*N*^{4},*N*^{4},*N*^{5},*N*^{5}-tetramethyl-1,3,2-dioxaborolane-4,5-dicarboxamide ((*S*,*S*)-**10**).

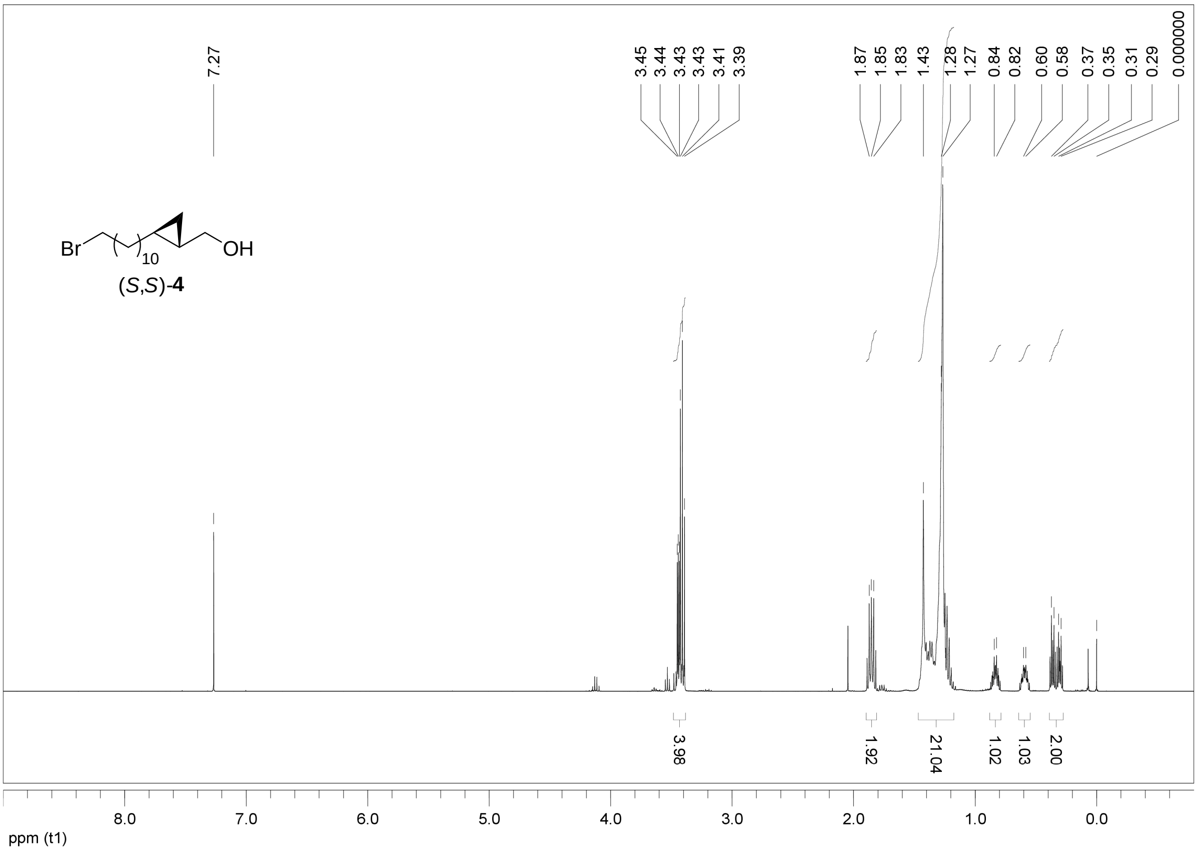

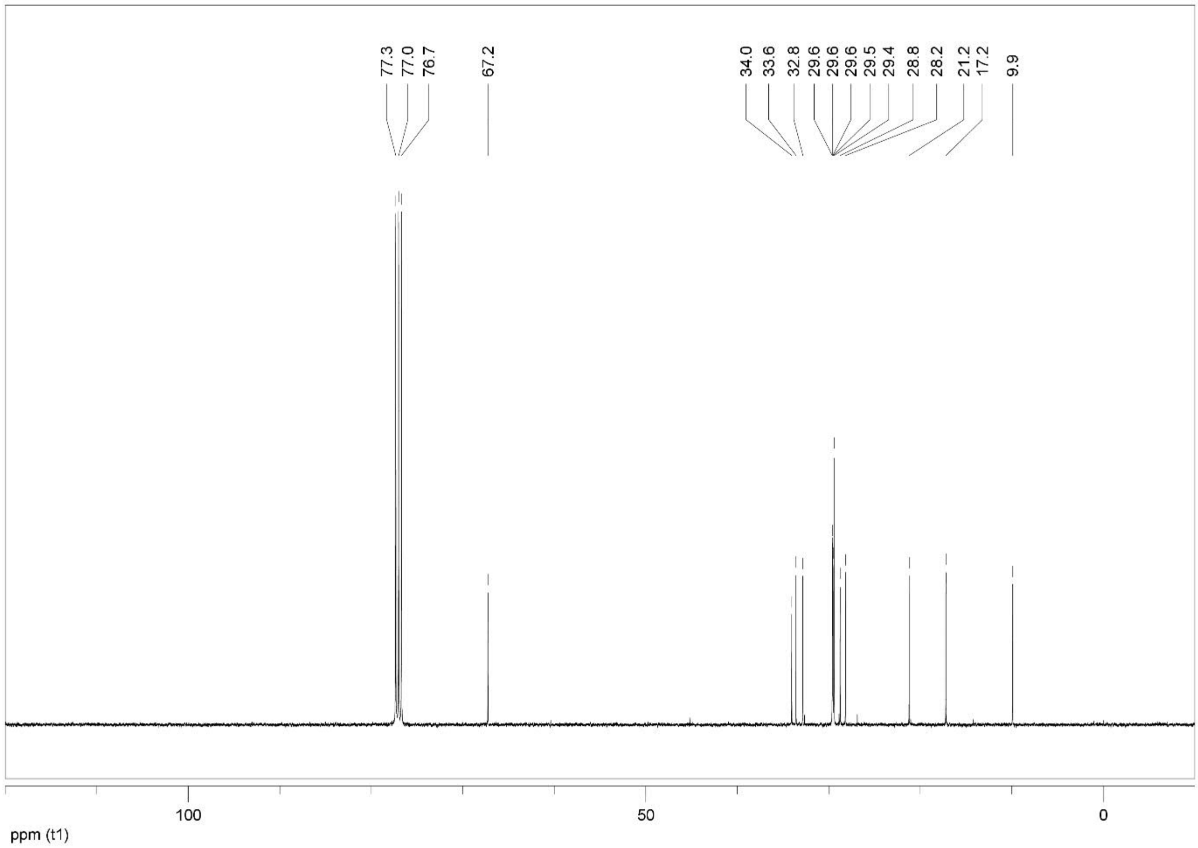

Figure S30: ^{1}H- (400 MHz) and ^{13}C-NMR (100 MHz) spectra of ((1*S*,2*S*)-2-(11-bromundecyl)cyclopropyl)methanol ((*S*,*S*)-**4**).

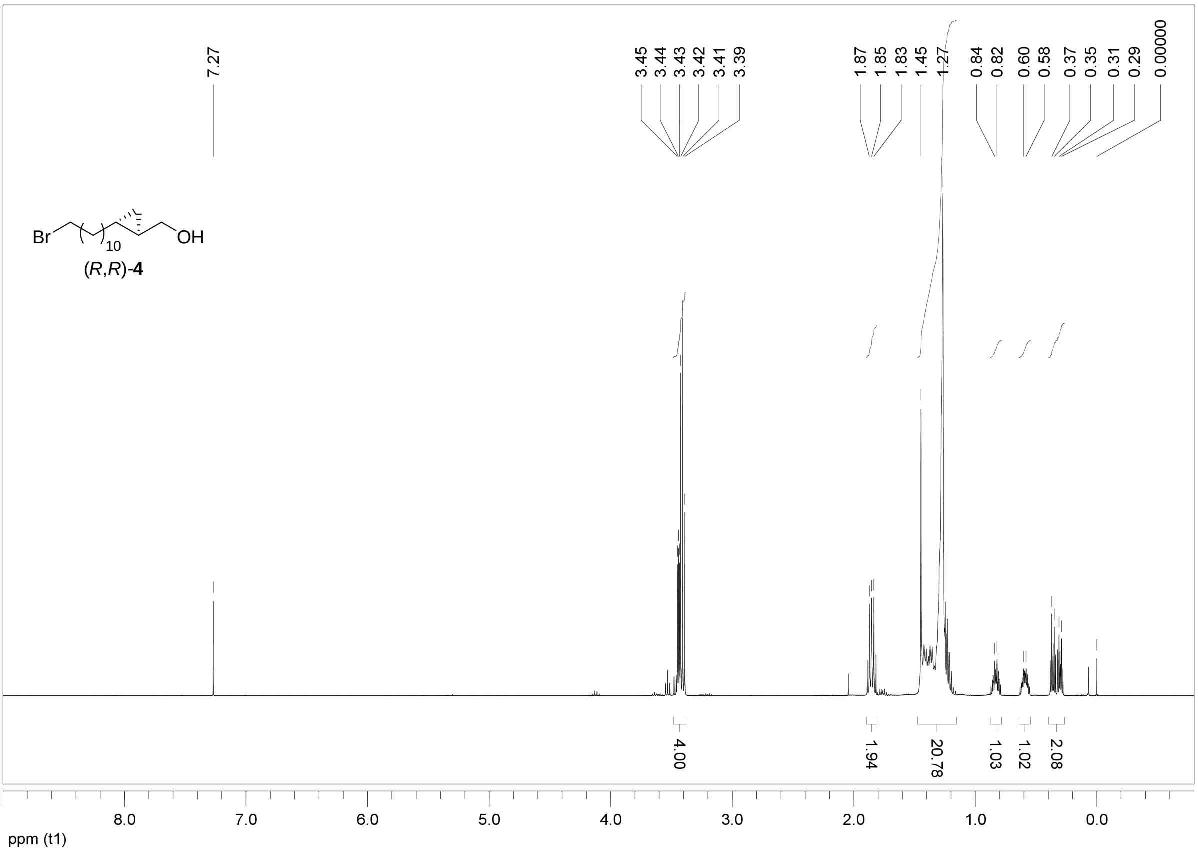

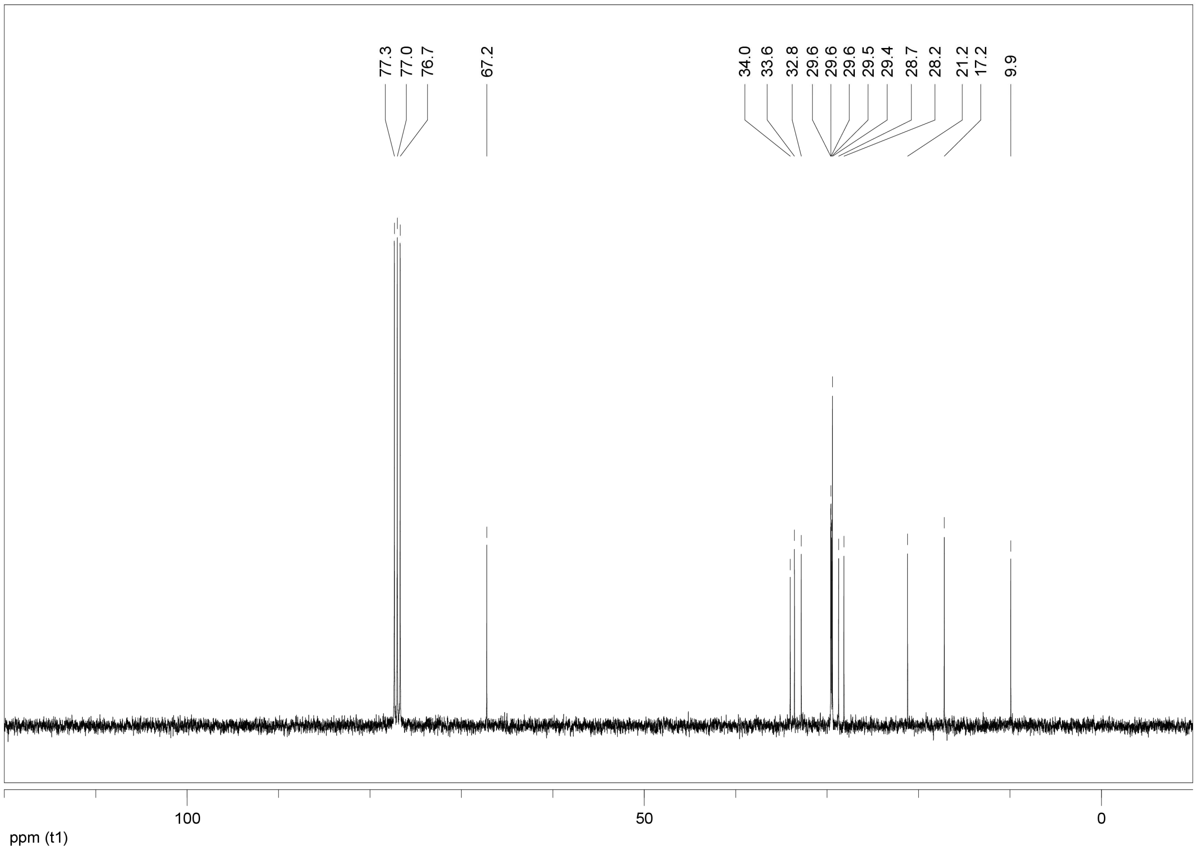

Figure S31: ^{1}H- (400 MHz) and ^{13}C-NMR (100 MHz) spectra of ((1*R*,2*R*)-2-(11-bromundecyl)cyclopropyl)methanol ((*R*,*R*)-**4**).

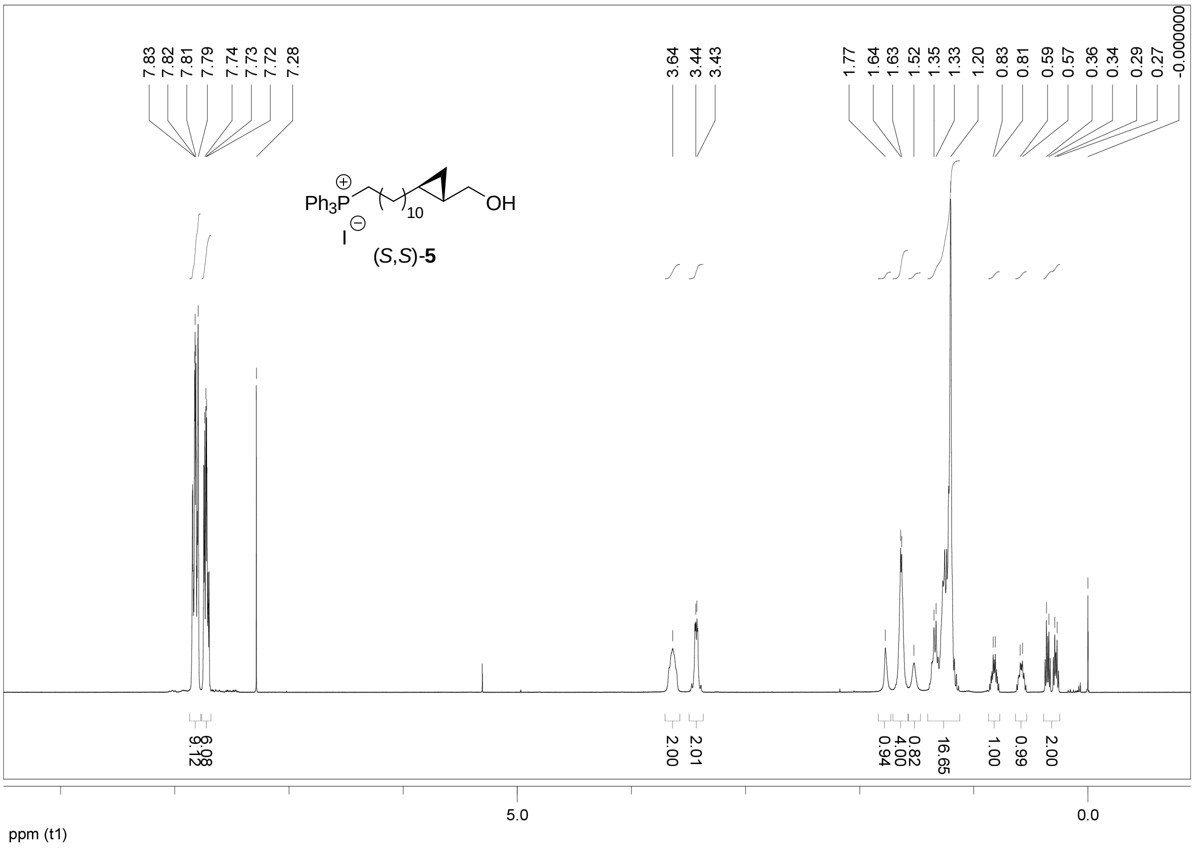

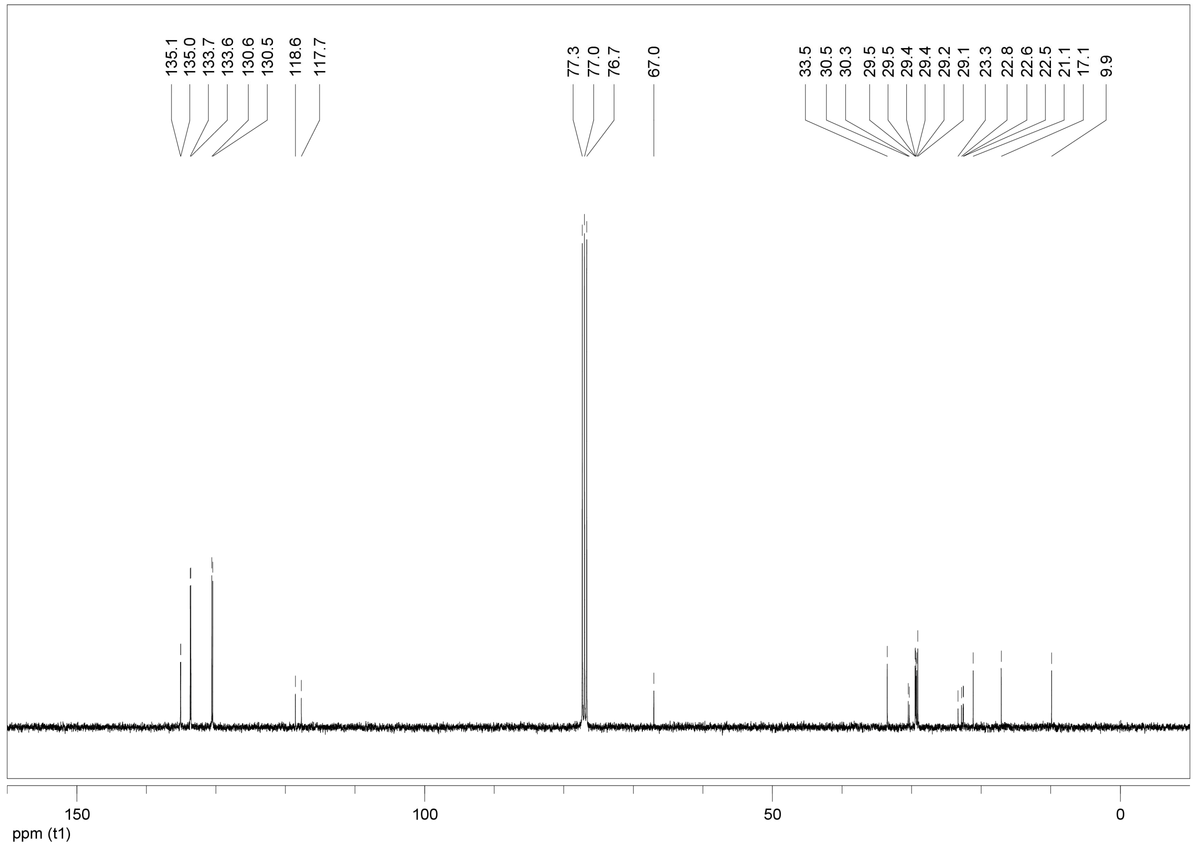

Figure S32: ^{1}H- (400 MHz) and ^{13}C-NMR (100 MHz) spectra of (11-((1*S*,2*S*)-2-(hydroxymethyl)cyclopropyl)undecyl)triphenylphosphonium iodide ((*S*,*S*)-**5**)

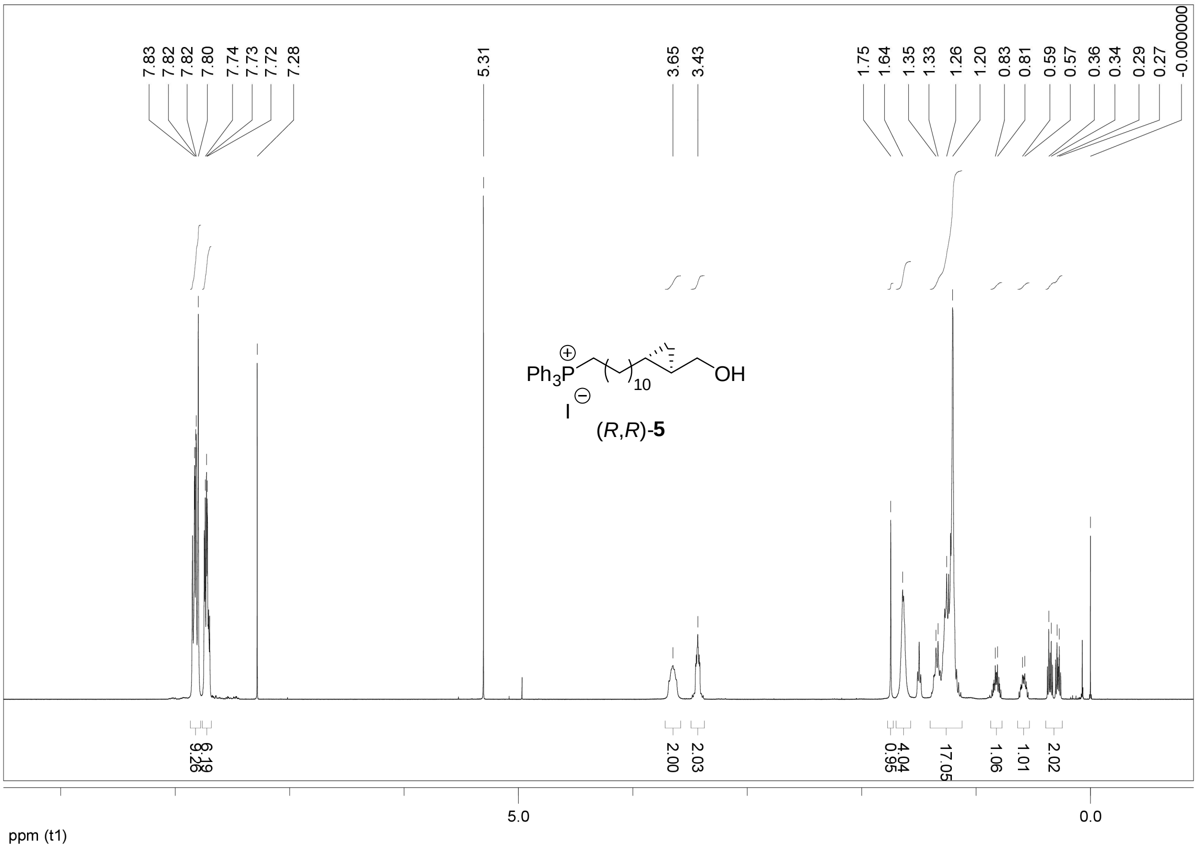

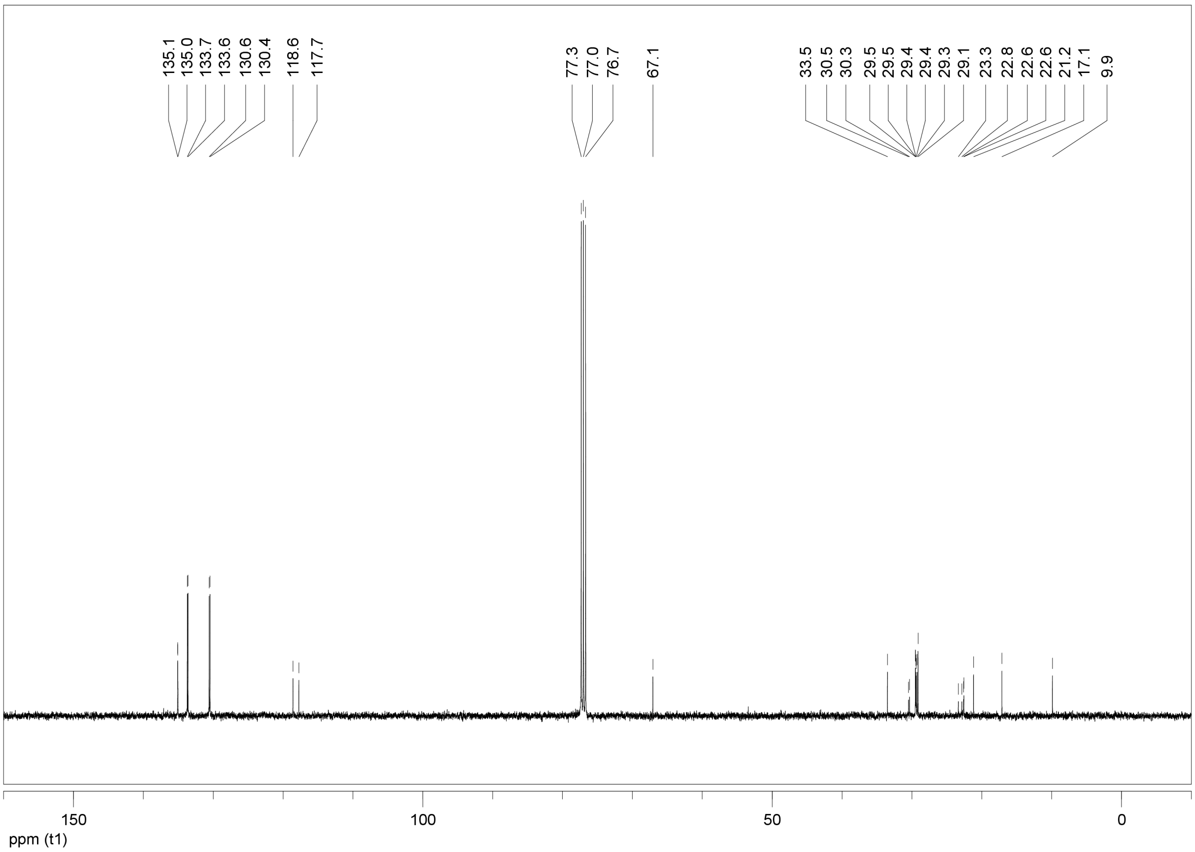

Figure S33: ^{1}H- (400 MHz) and ^{13}C-NMR (100 MHz) spectra of (11-((1*R*,2*R*)-2-(hydroxymethyl)cyclopropyl)undecyl)triphenylphosphonium iodide ((*R*,*R*)-**5**)

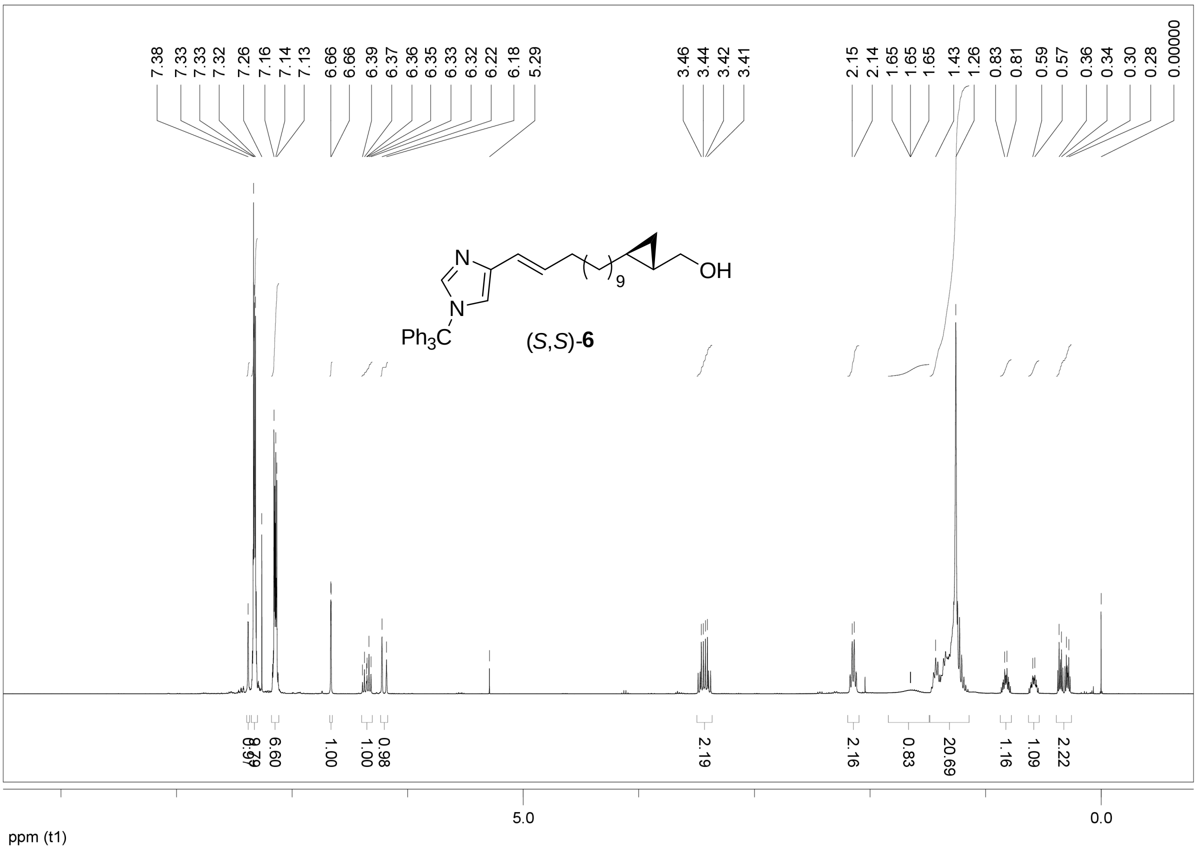

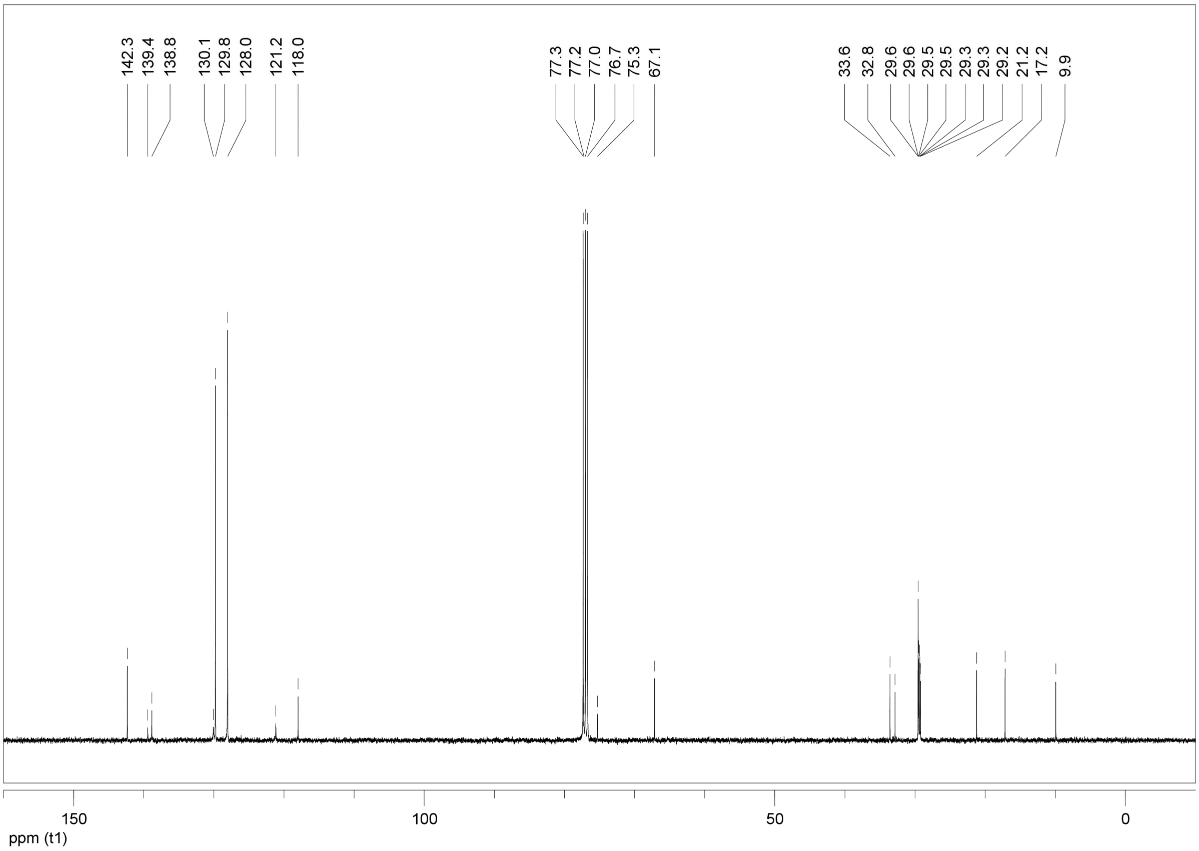

Figure S34: ^{1}H- (400 MHz) and ^{13}C-NMR (100 MHz) spectra of ((1*S*,2*S*)-2-((*E*)-12-(1-trityl-1*H*-imidazol-4-yl)dodec-11-en-1-yl)cyclopropyl)-methanol ((*S*,*S*)-**6**)

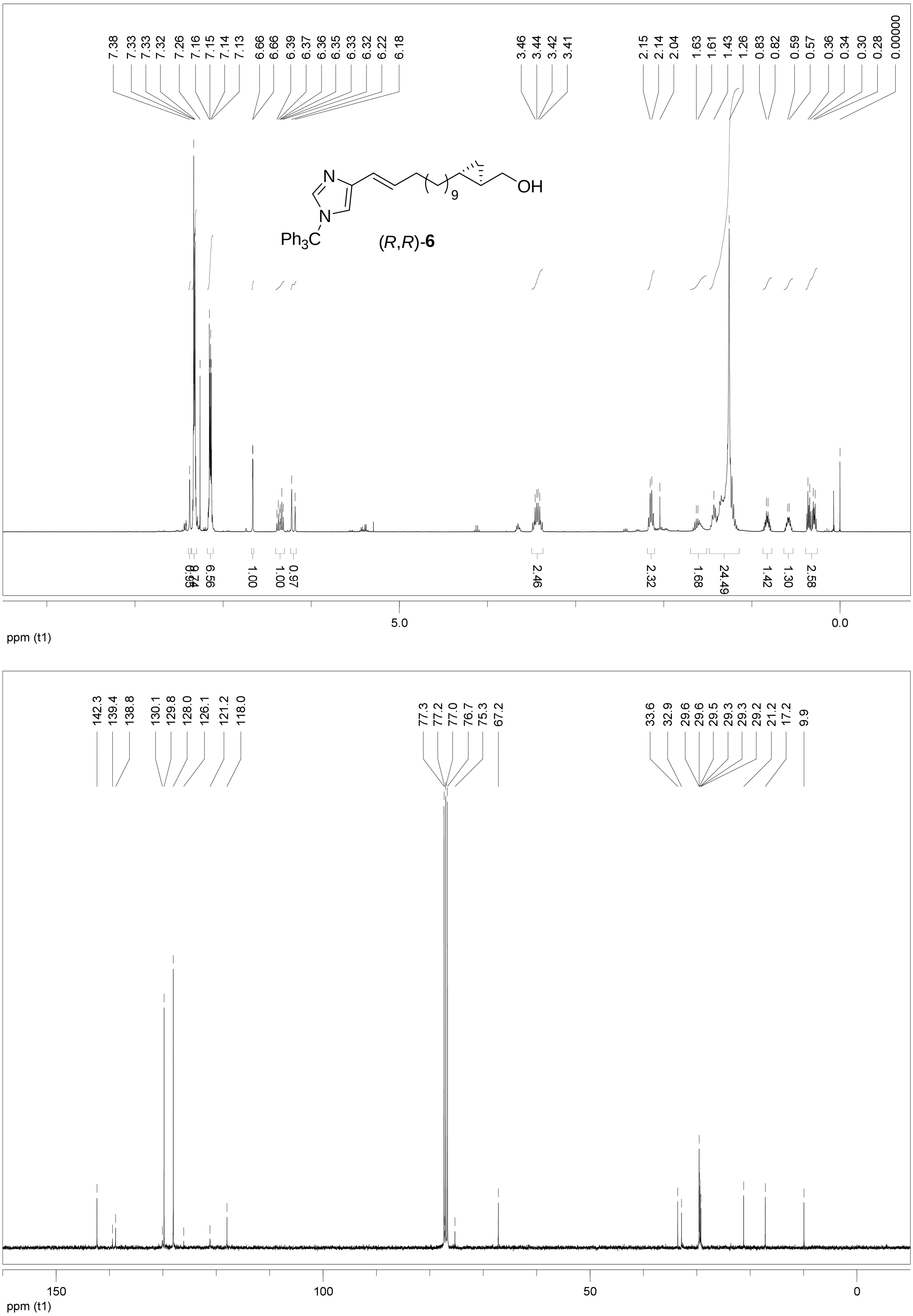

Figure S35: ^{1}H- (400 MHz) and ^{13}C-NMR (100 MHz) spectra of ((1*R*,2*R*)-2-((*E*)-12-(1-trityl-1*H*-imidazol-4-yl)dodec-11-en-1-yl)cyclopropyl)-methanol ((*R*,*R*)-**6**)

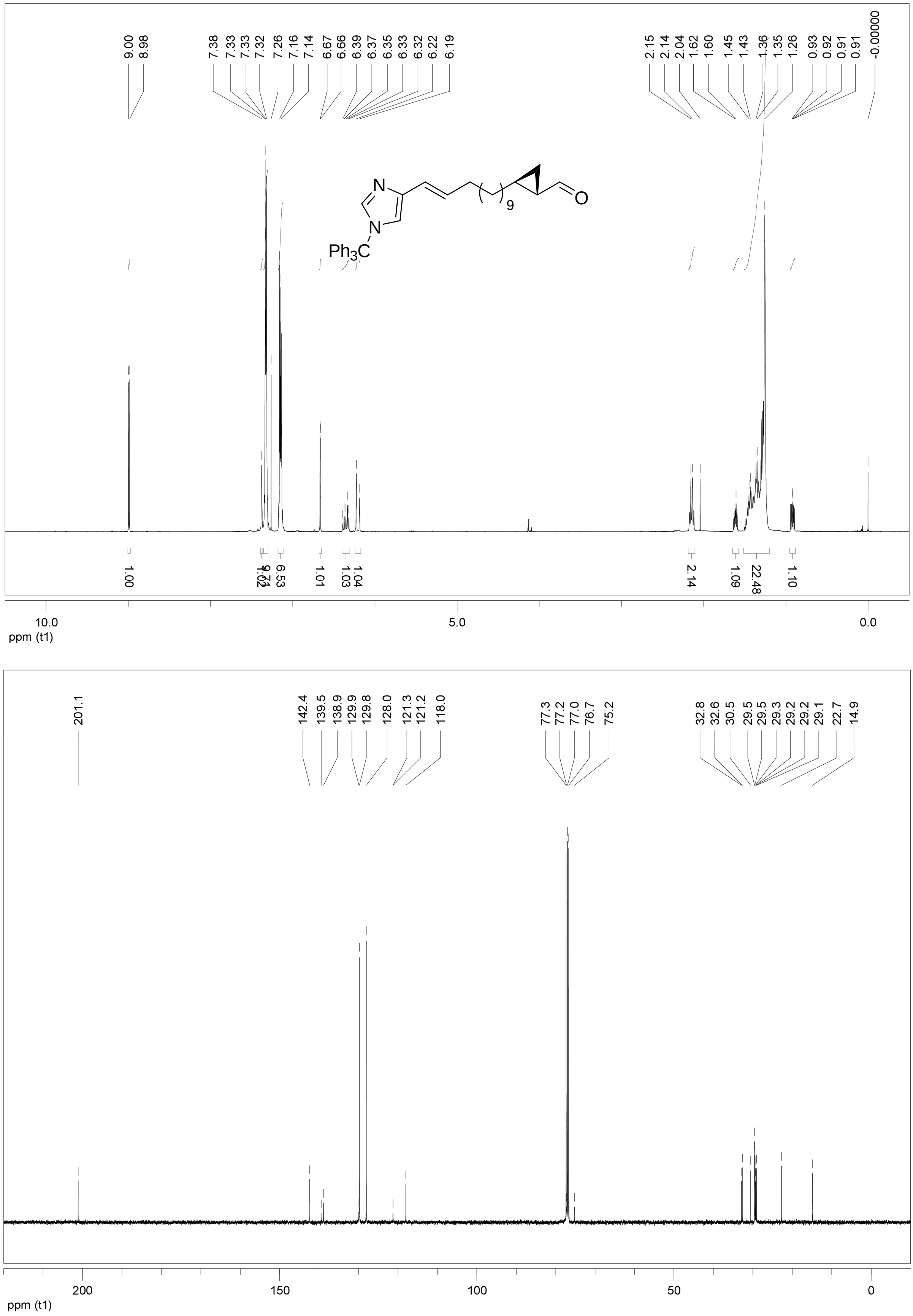

Figure S36: ^{1}H- (400 MHz) and ^{13}C-NMR (100 MHz) spectra of (1*S*,2*S*)-2-((*E*)-12-(1-trityl-1*H*-imidazol-4-yl)dodec-11-en-1-yl)cyclopropane-1-carbaldehyde.

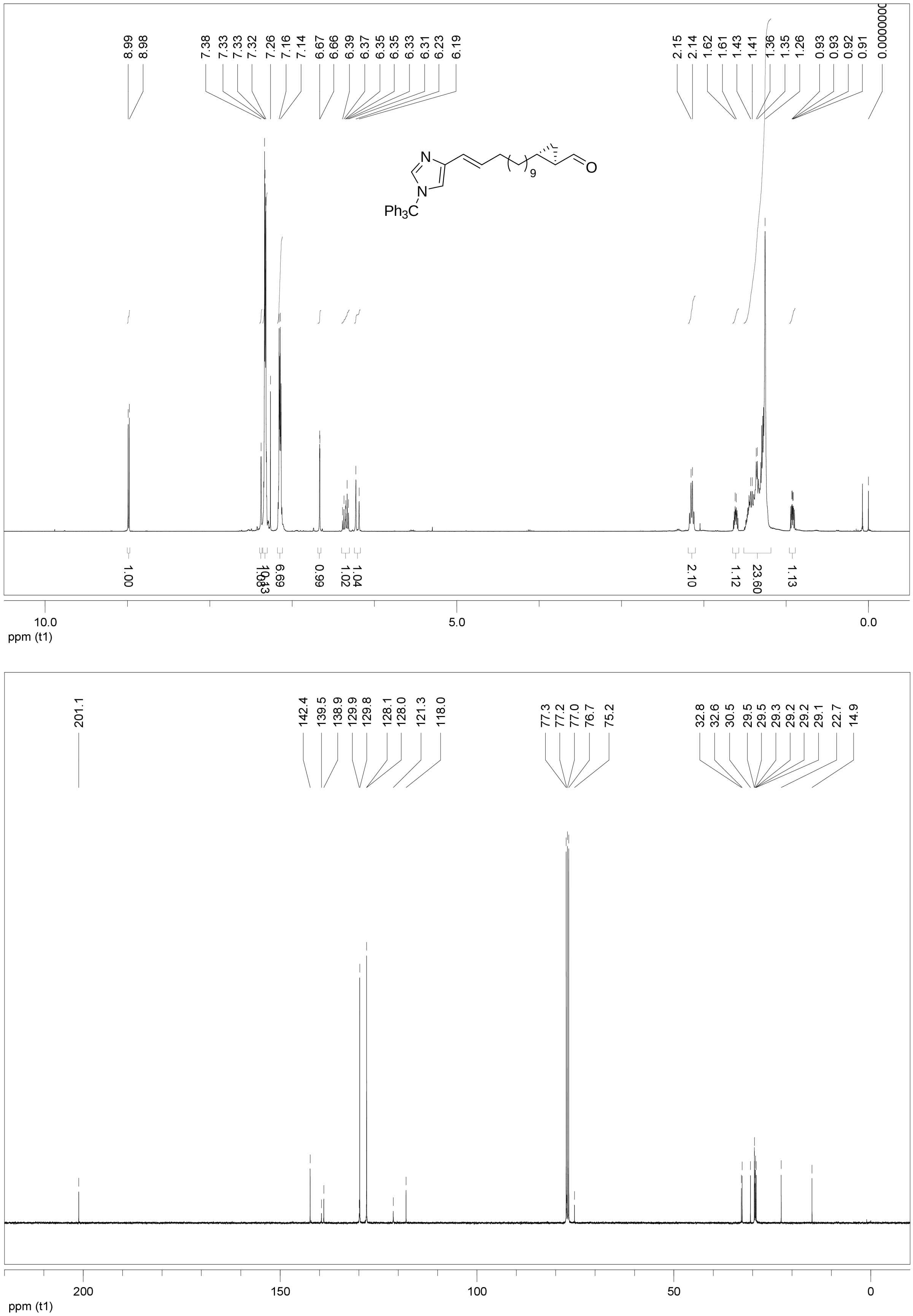

Figure S37: ^{1}H- (400 MHz) and ^{13}C-NMR (100 MHz) spectra of (1*R*,2*R*)-2-((*E*)-12-(1-trityl-1*H*-imidazol-4-yl)dodec-11-en-1-yl)cyclopropane-1-carbaldehyde.

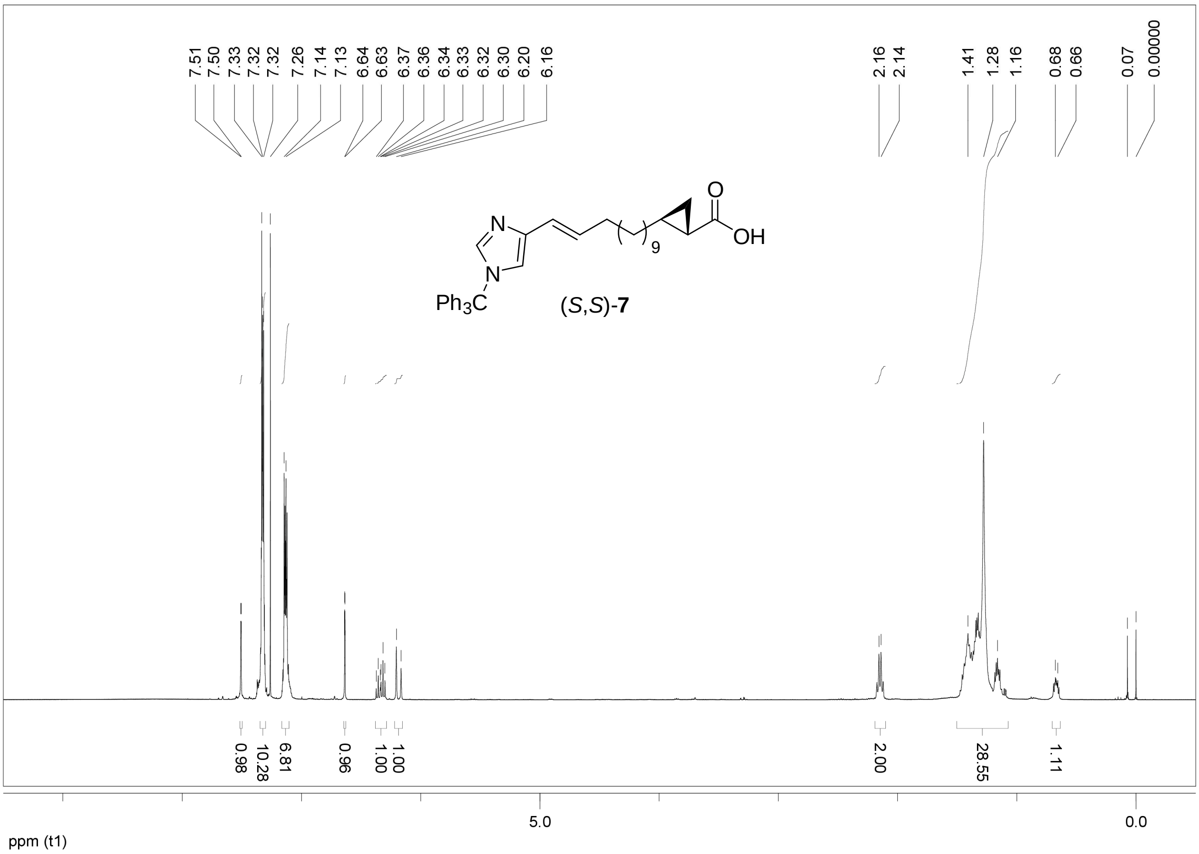

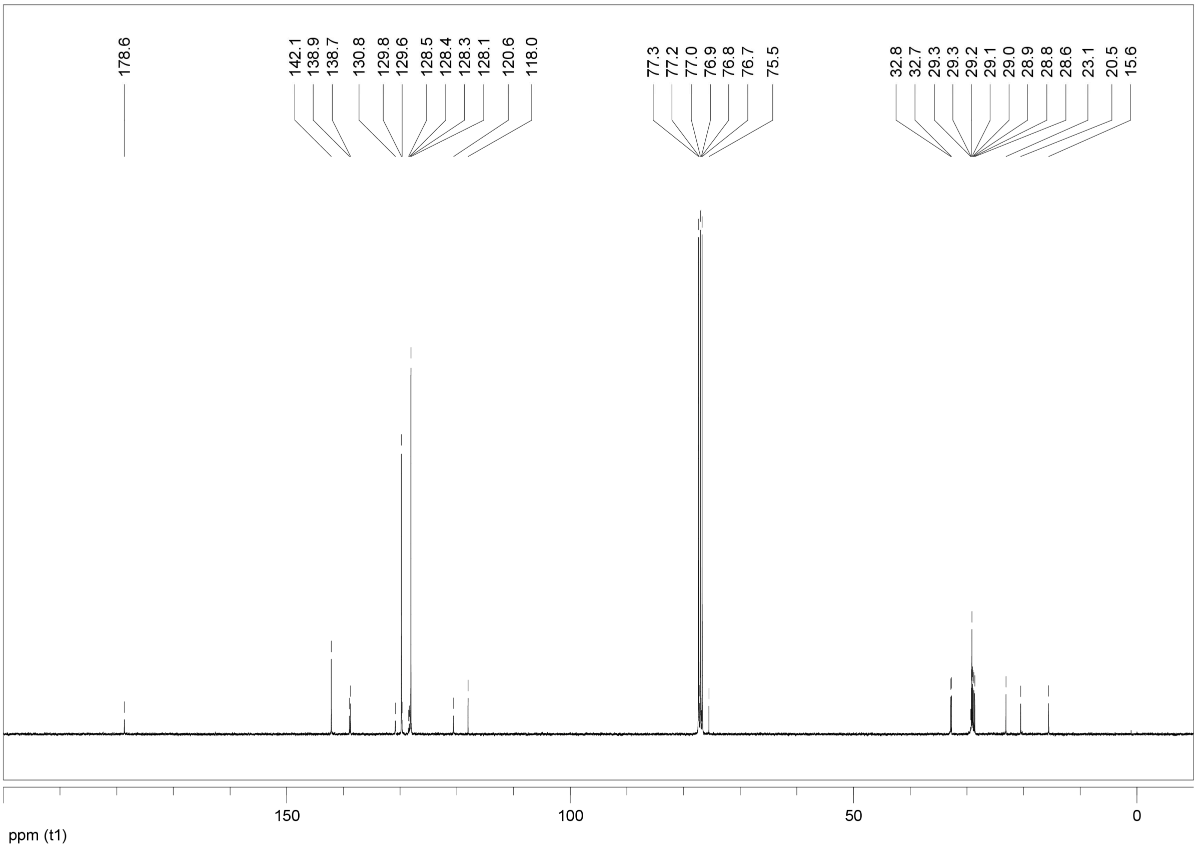

Figure S38: ^{1}H- (400 MHz) and ^{13}C-NMR (100 MHz) spectra of (1*S*,2*S*)-2-((*E*)-12-(1-trityl-1*H*-imidazol-4-yl)dodec-11-en-1-yl)cyclopropane-1-carboxylic acid ((*S*,*S*)-**7**)

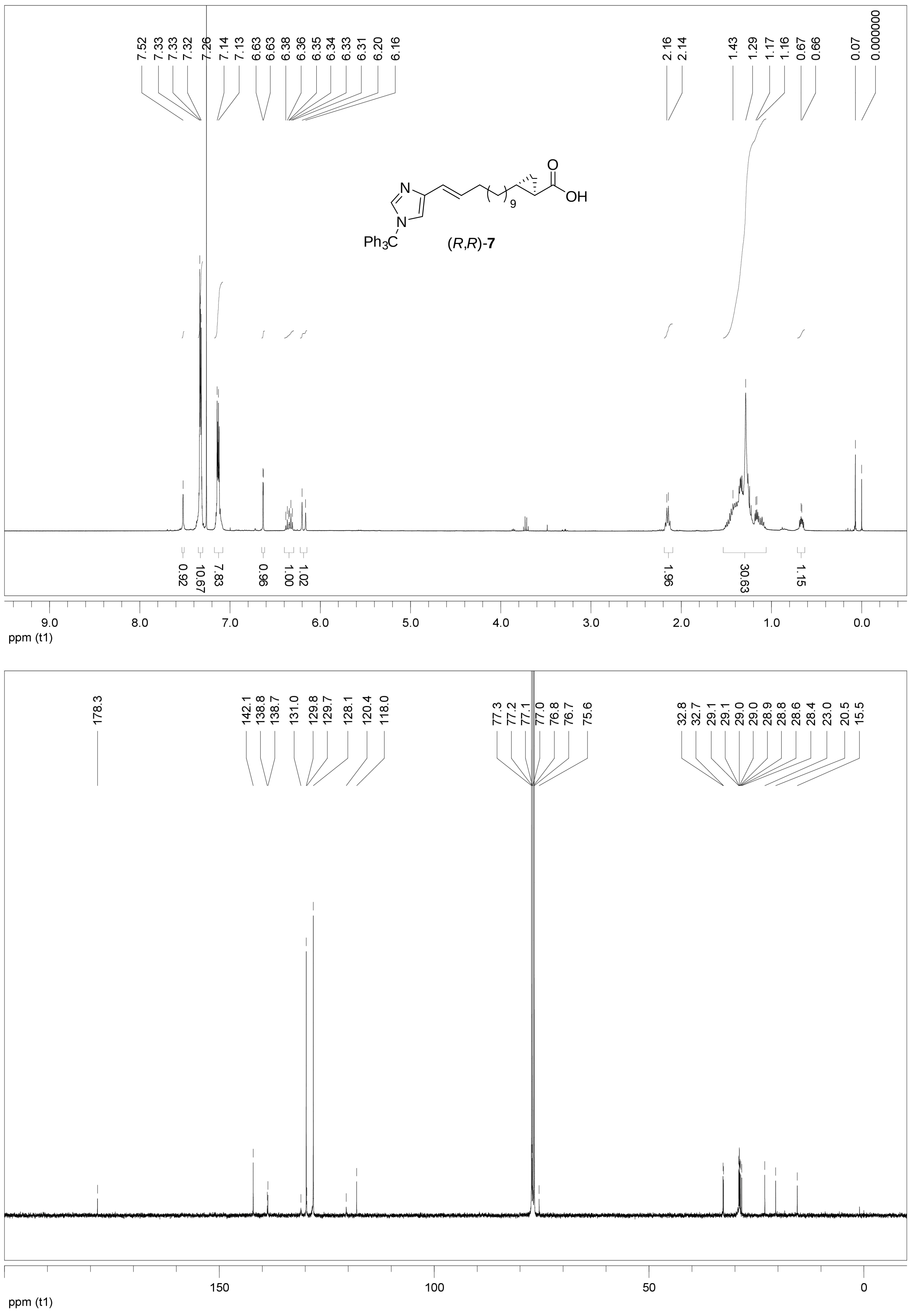

Figure S39: ^{1}H- (400 MHz) and ^{13}C-NMR (100 MHz) spectra of (1*R*,2*R*)-2-((*E*)-12-(1-trityl-1*H*-imidazol-4-yl)dodec-11-en-1-yl)cyclopropane-1-carboxylic acid ((*R*,*R*)-**7**)

Synthesis of imidacin B1

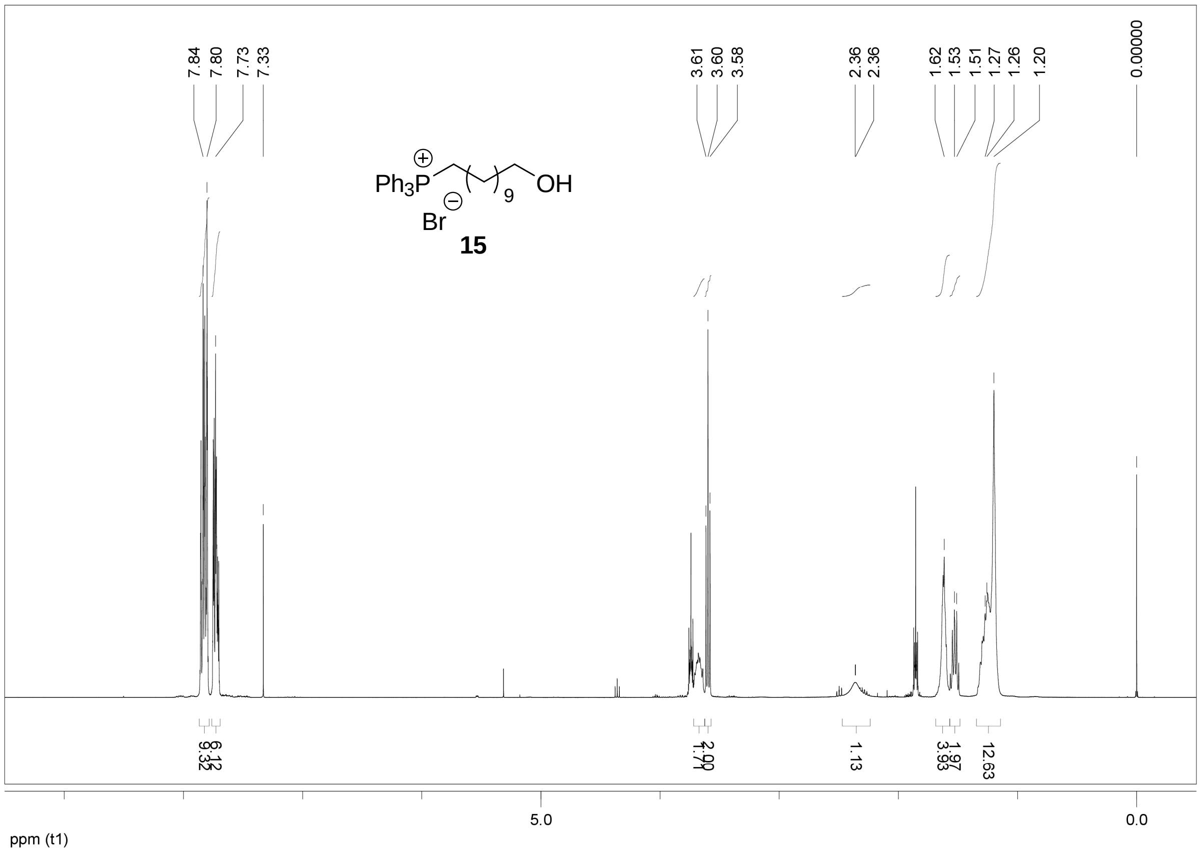

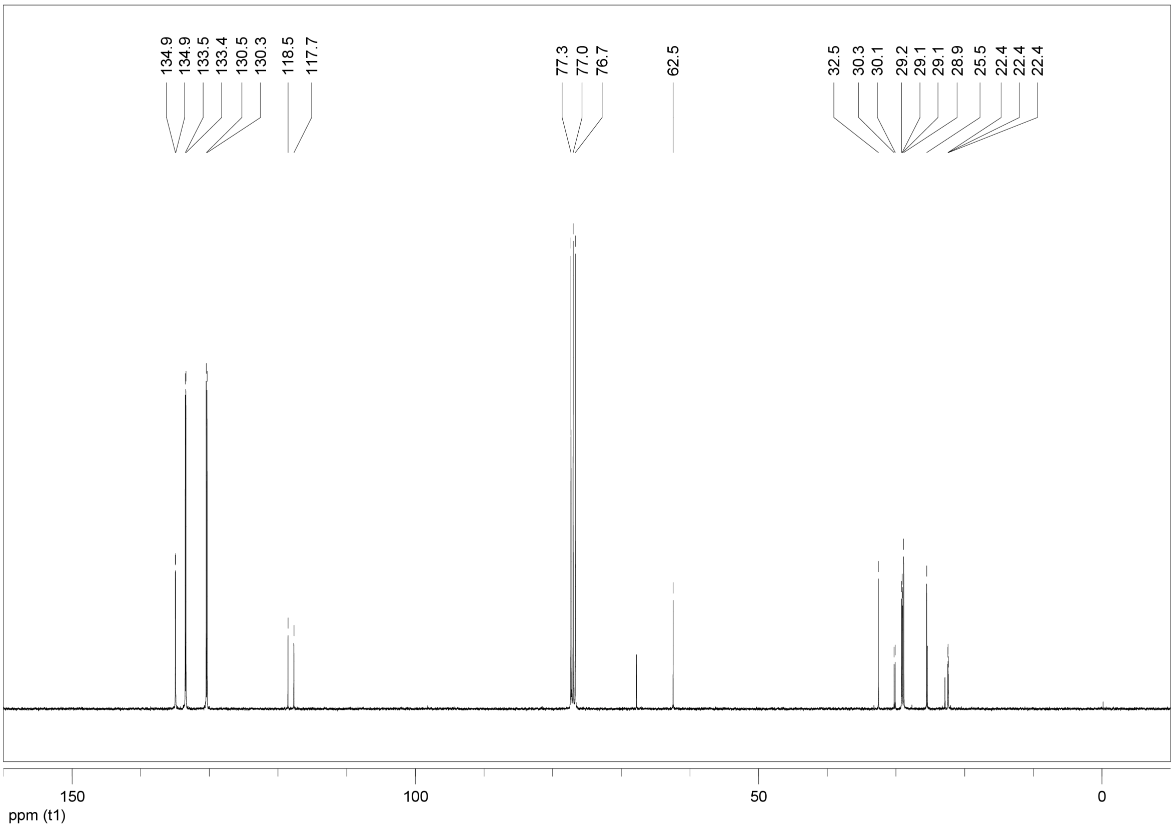

Figure S40: ^{1}H- (400 MHz) and ^{13}C-NMR (100 MHz) spectra of (11-hydroxyundecyl)triphenylphosphonium bromide (**15**)

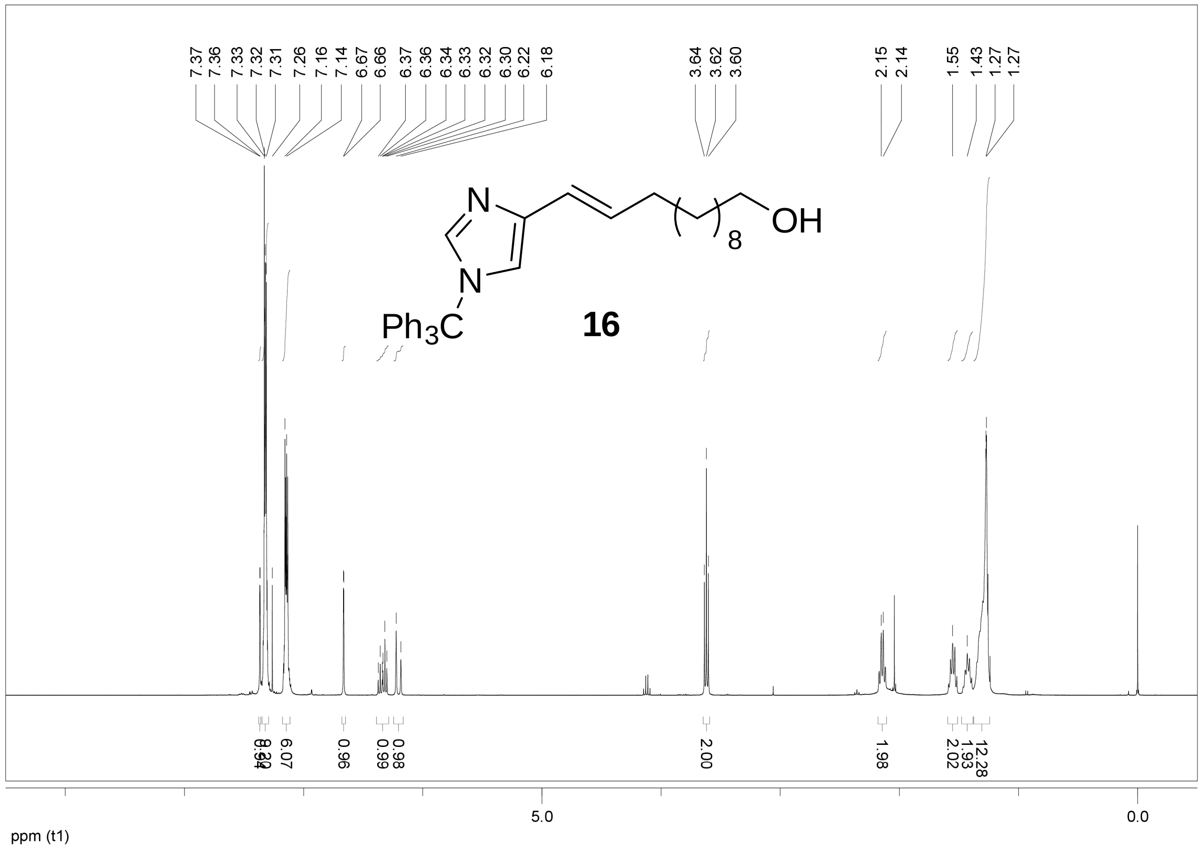

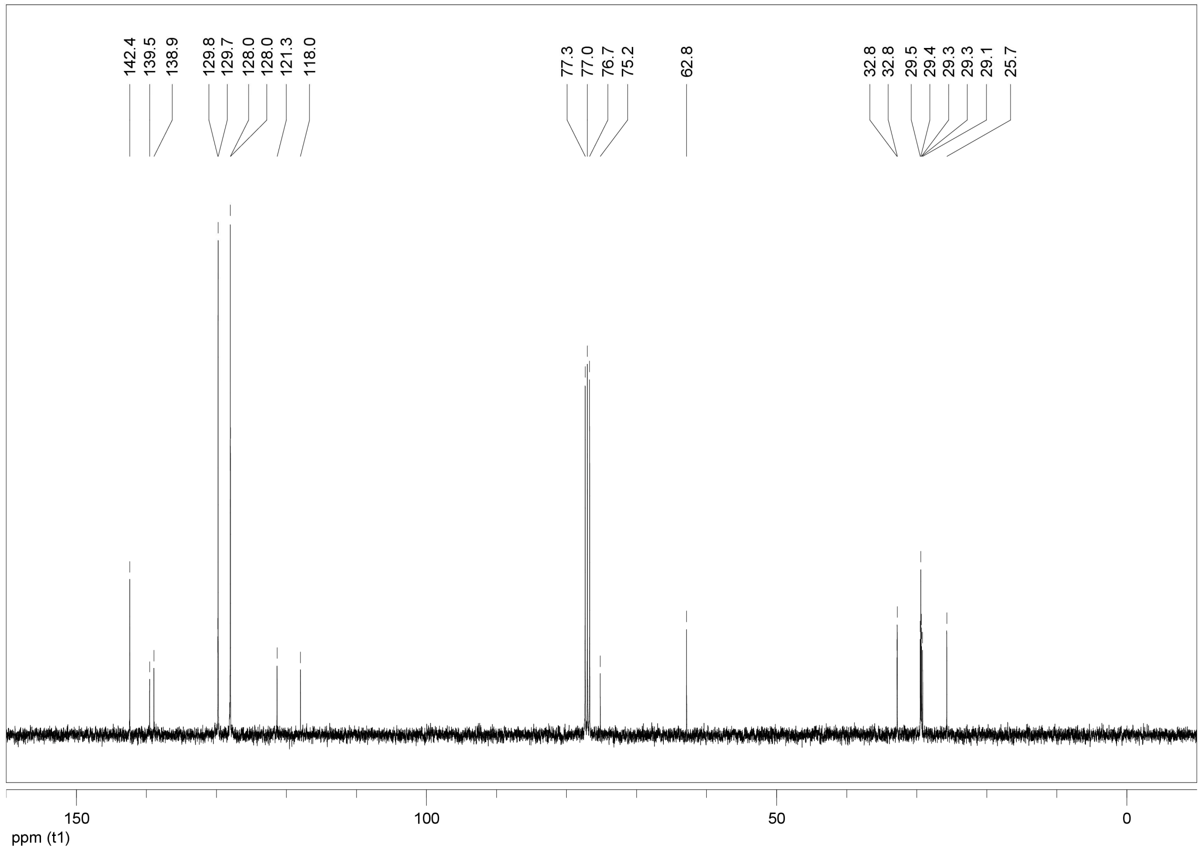

Figure S41: ^{1}H- (400 MHz) and ^{13}C-NMR (100 MHz) spectra of (*E*)-12-(1-trityl-1*H*-imidazol-4-yl)dodec-11-en-1-ol (**16**)

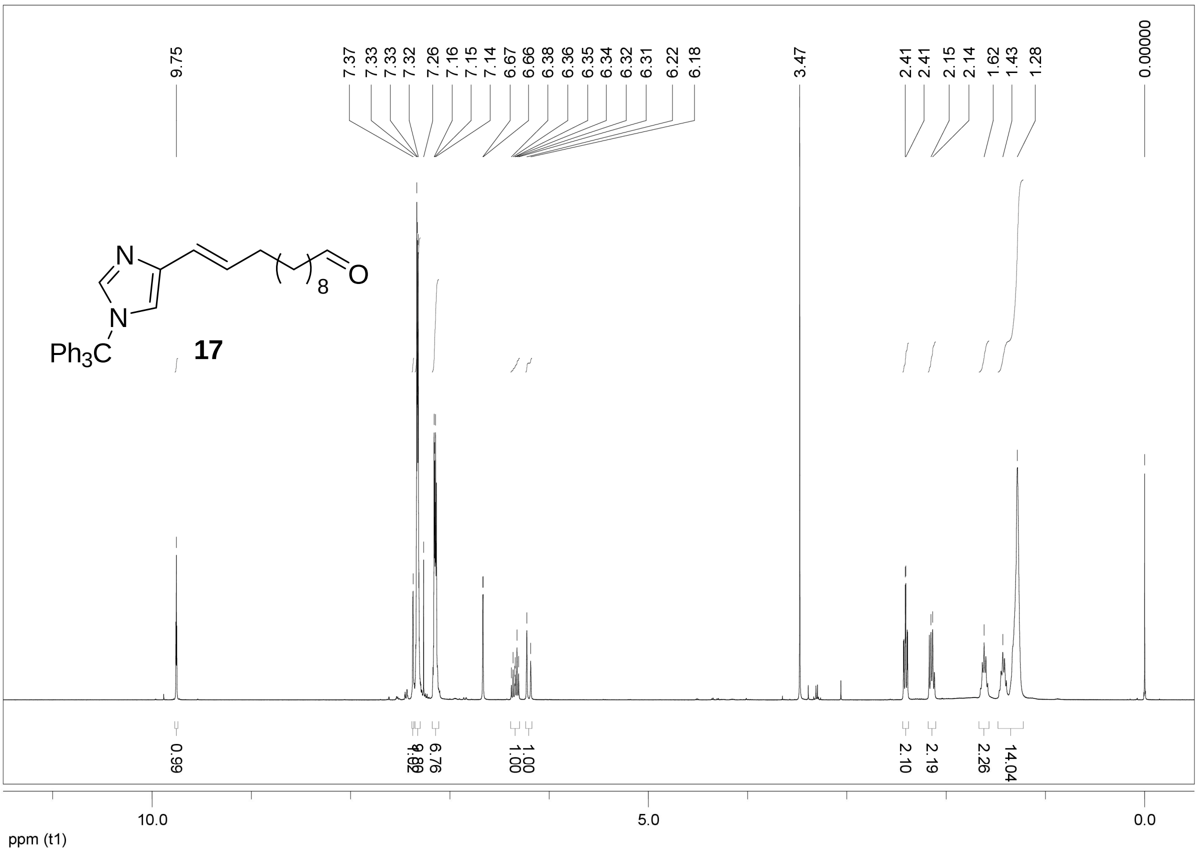

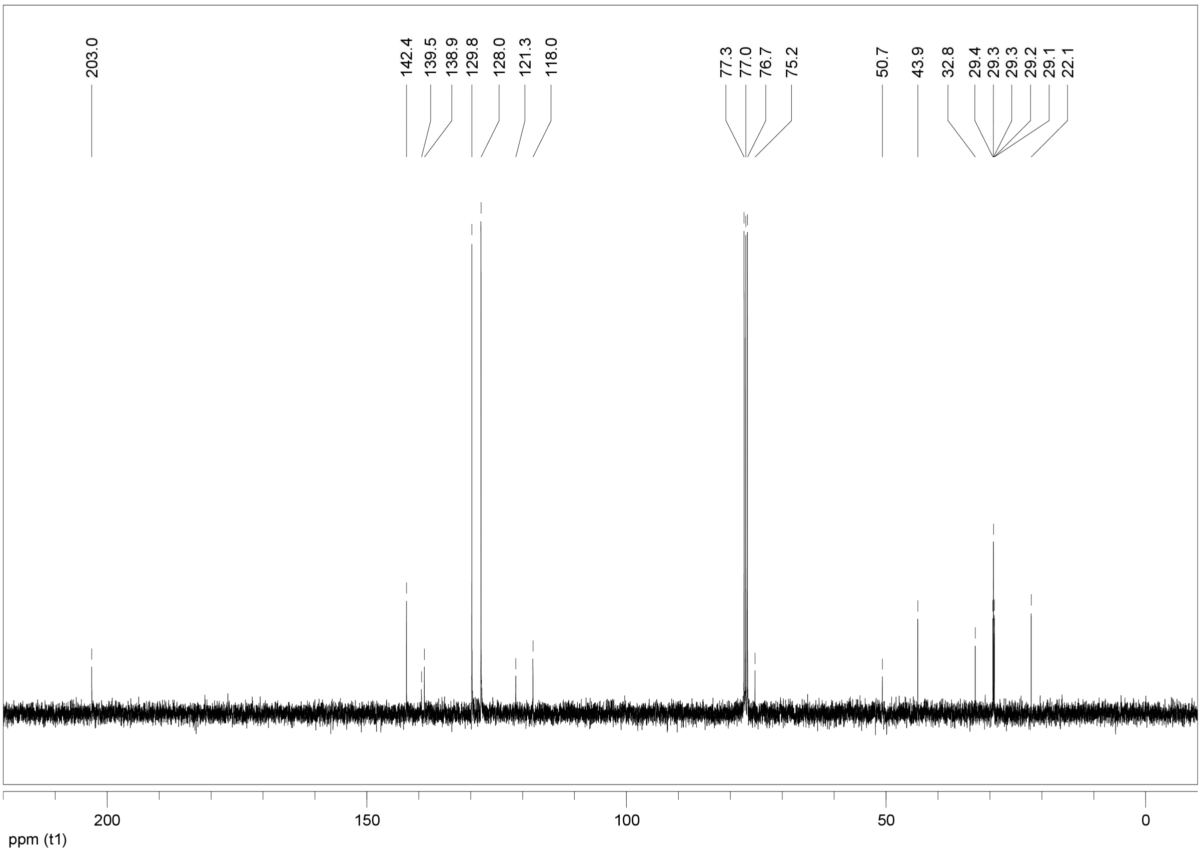

Figure S42: ^{1}H- (400 MHz) and ^{13}C-NMR (100 MHz) spectra of (*E*)-12-(1-trityl-1*H*-imidazol-4-yl)dodec-11-enal (**17**)

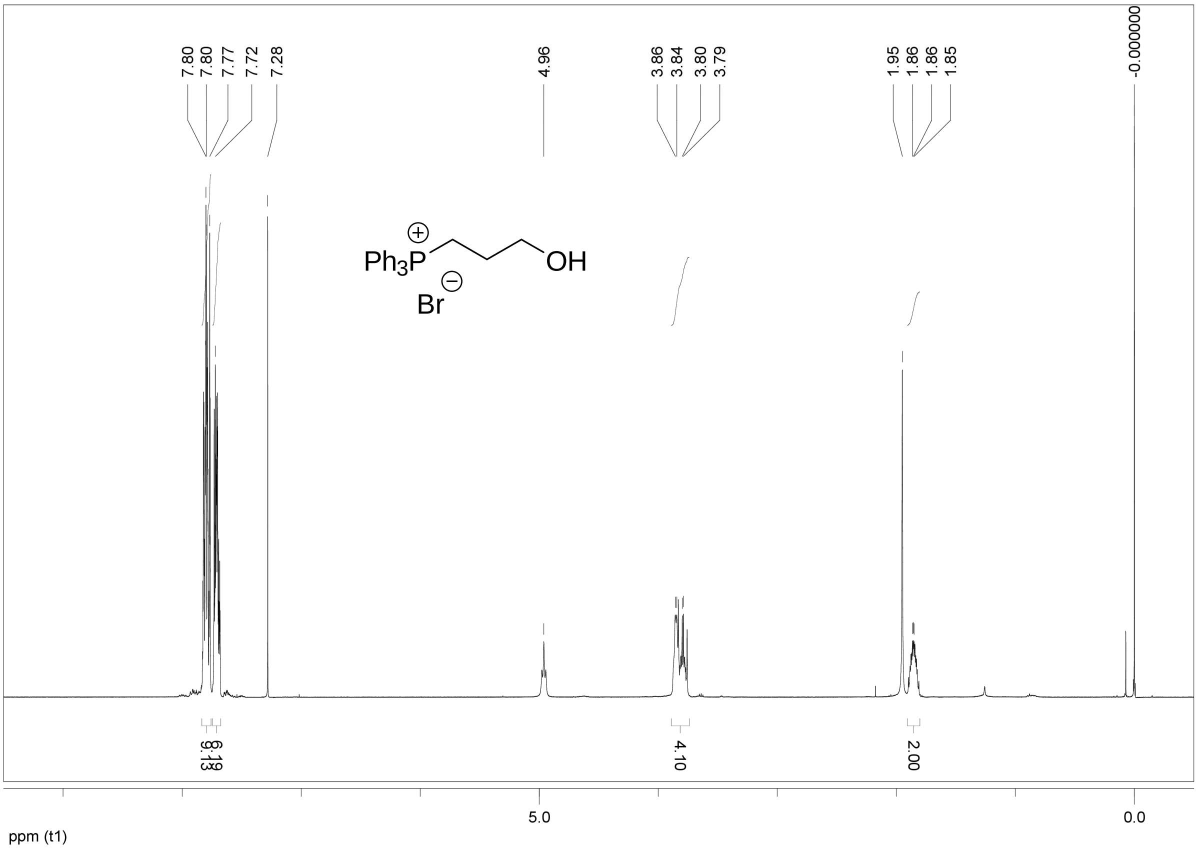

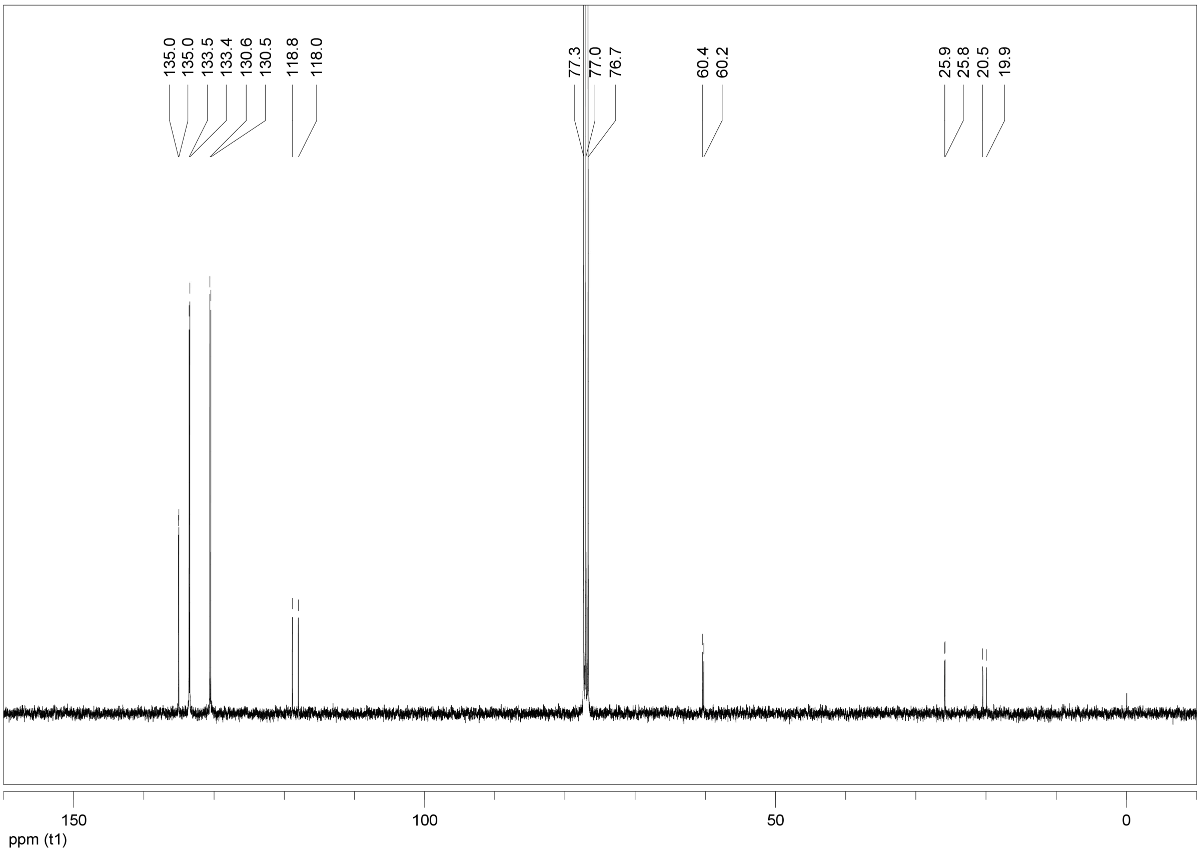

Figure S43: ^{1}H- (400 MHz) and ^{13}C-NMR (100 MHz) spectra of (3-hydroxypropyl)triphenylphosphonium bromide

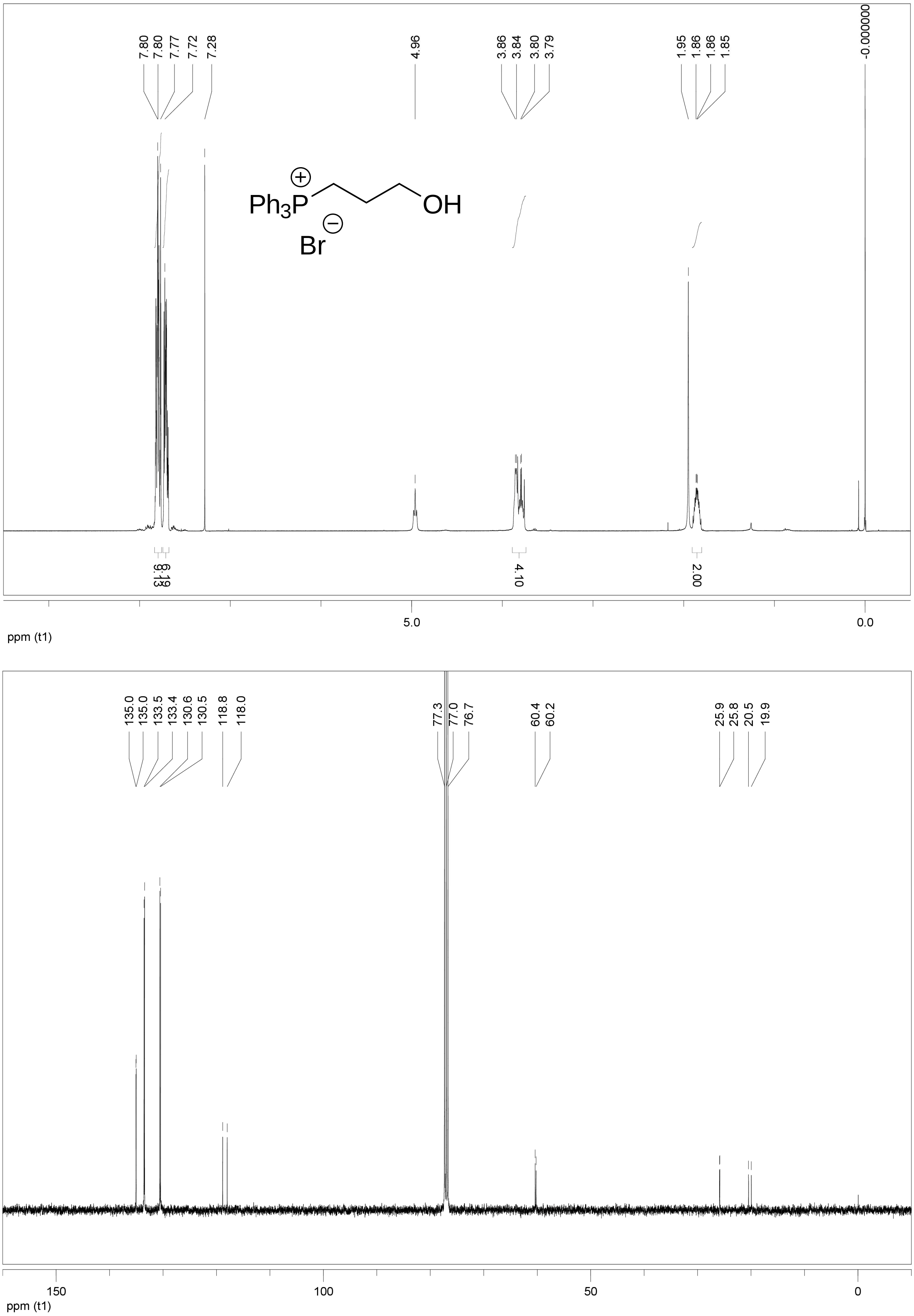

Figure S44: ^{1}H- (400 MHz) and ^{13}C-NMR (100 MHz) spectra of (3-hydroxypropyl)triphenylphosphonium bromide

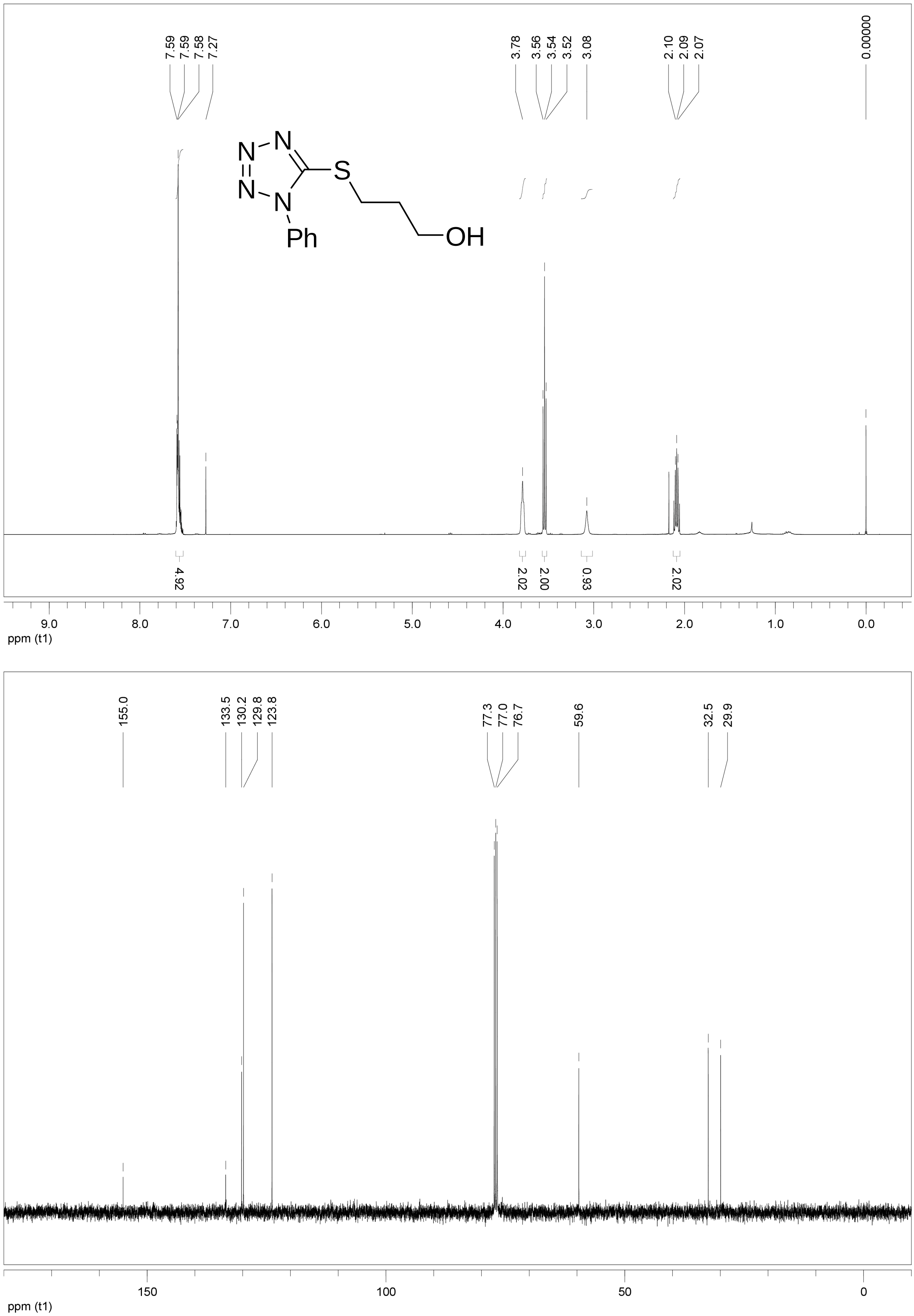

Figure S45: ^{1}H- (400 MHz) and ^{13}C-NMR (100 MHz) spectra of 3-((1-phenyl-1*H*-tetrazol-5-yl)thio)propan-1-ol

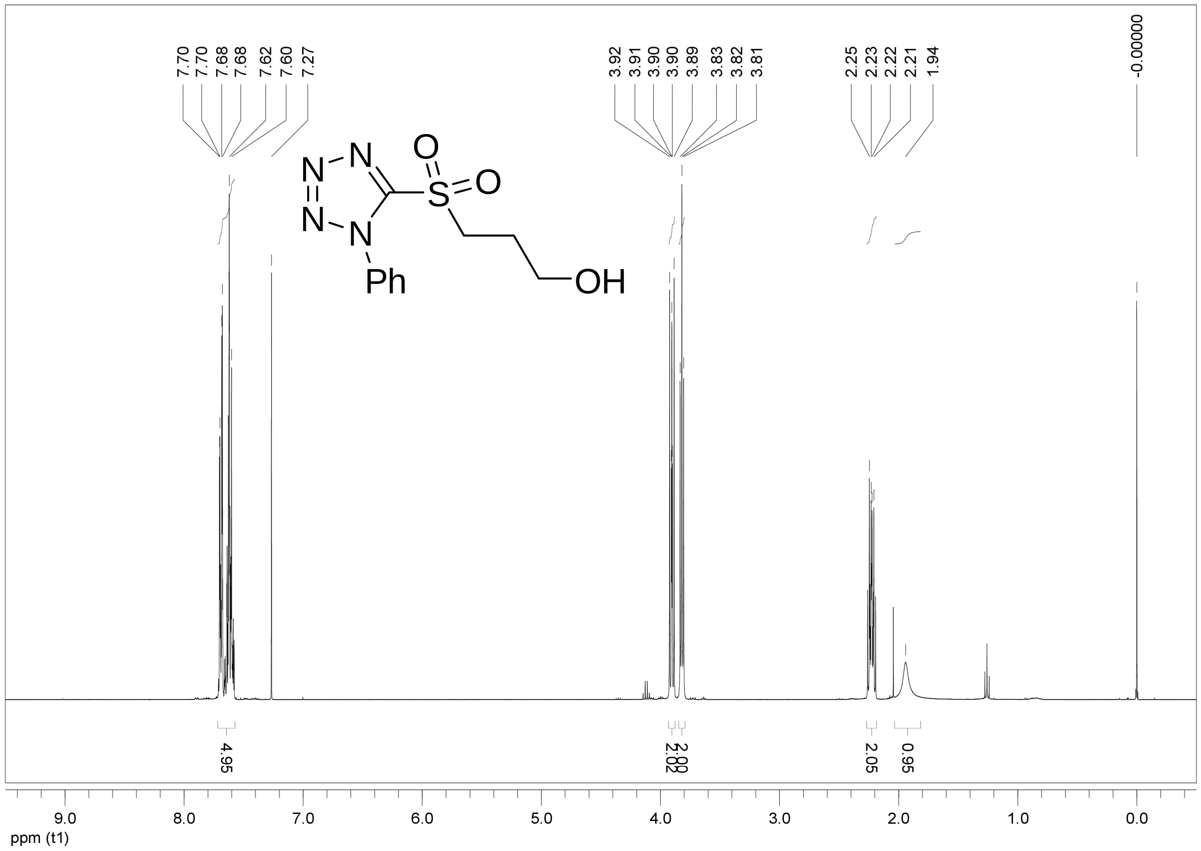

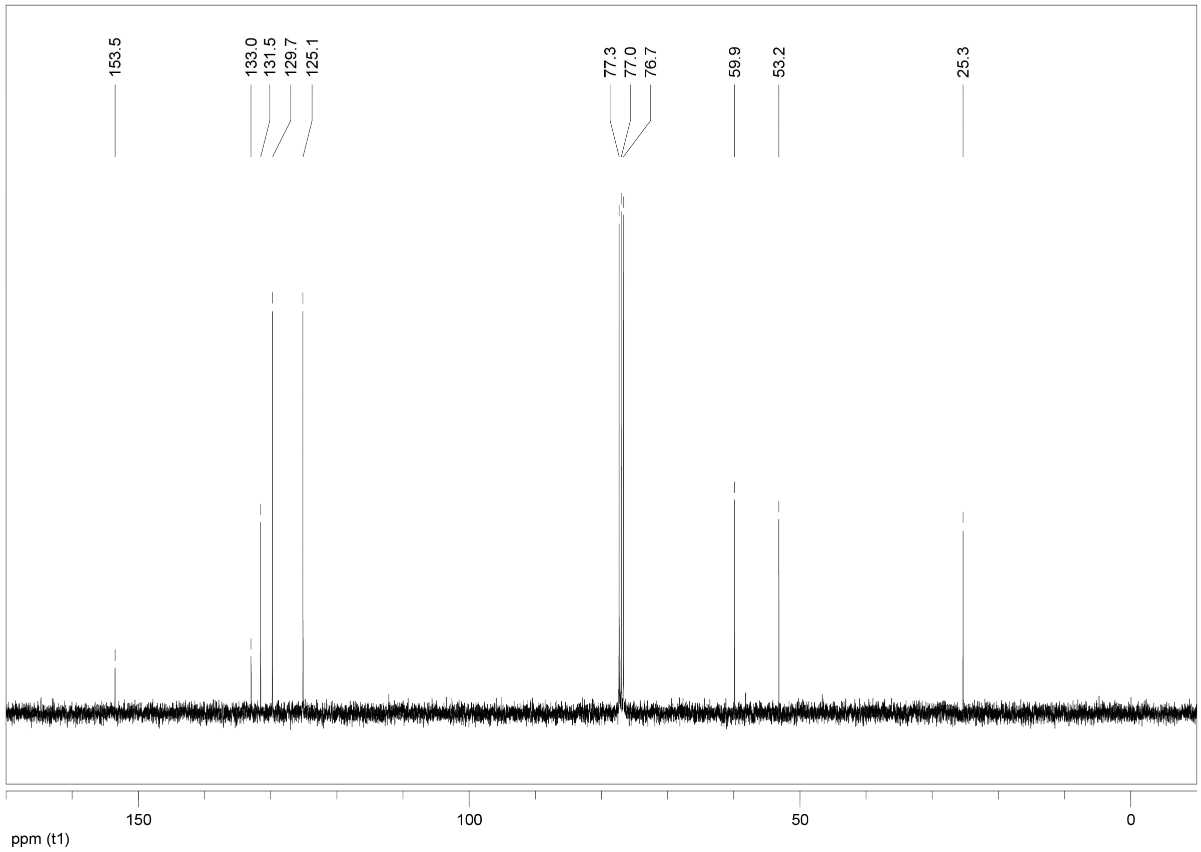

Figure S46: ^{1}H- (400 MHz) and ^{13}C-NMR (100 MHz) spectra of 3-((1-phenyl-1*H*-tetrazol-5-yl)sulfonyl)propan-1-ol

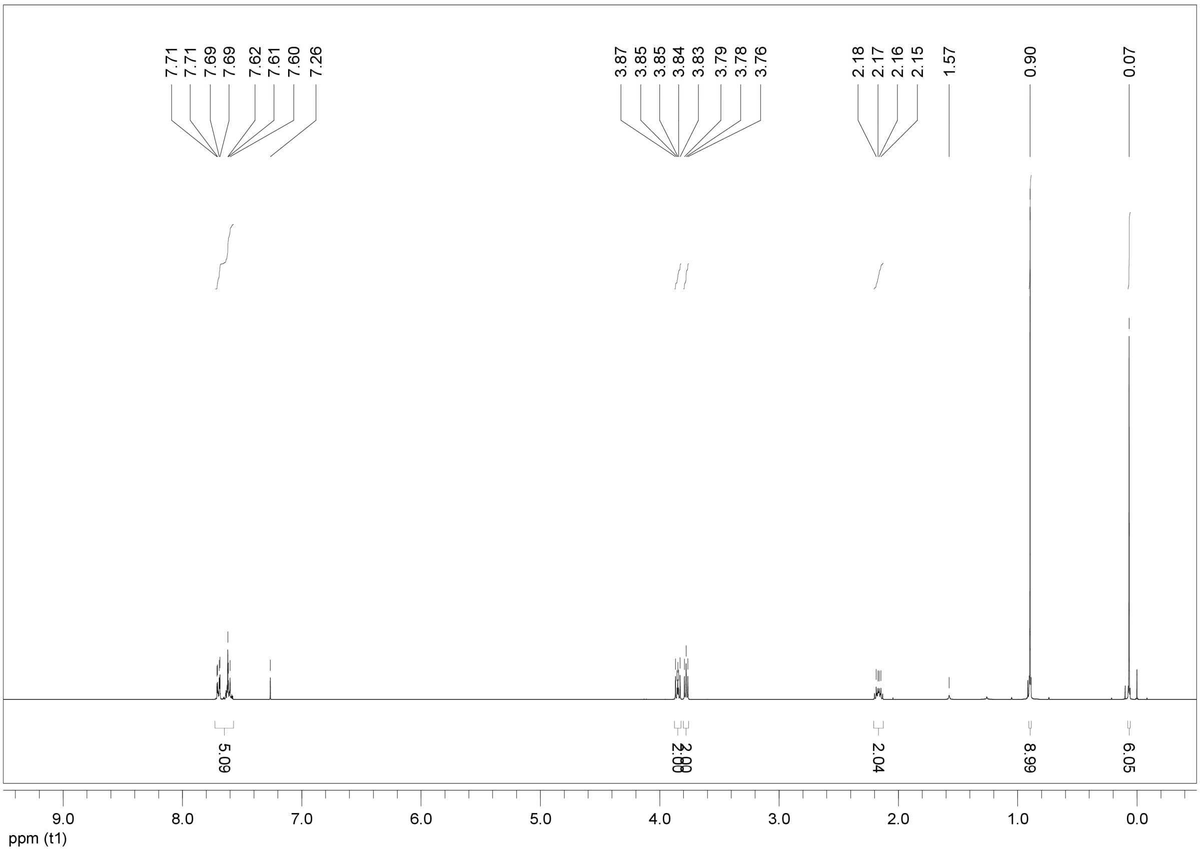

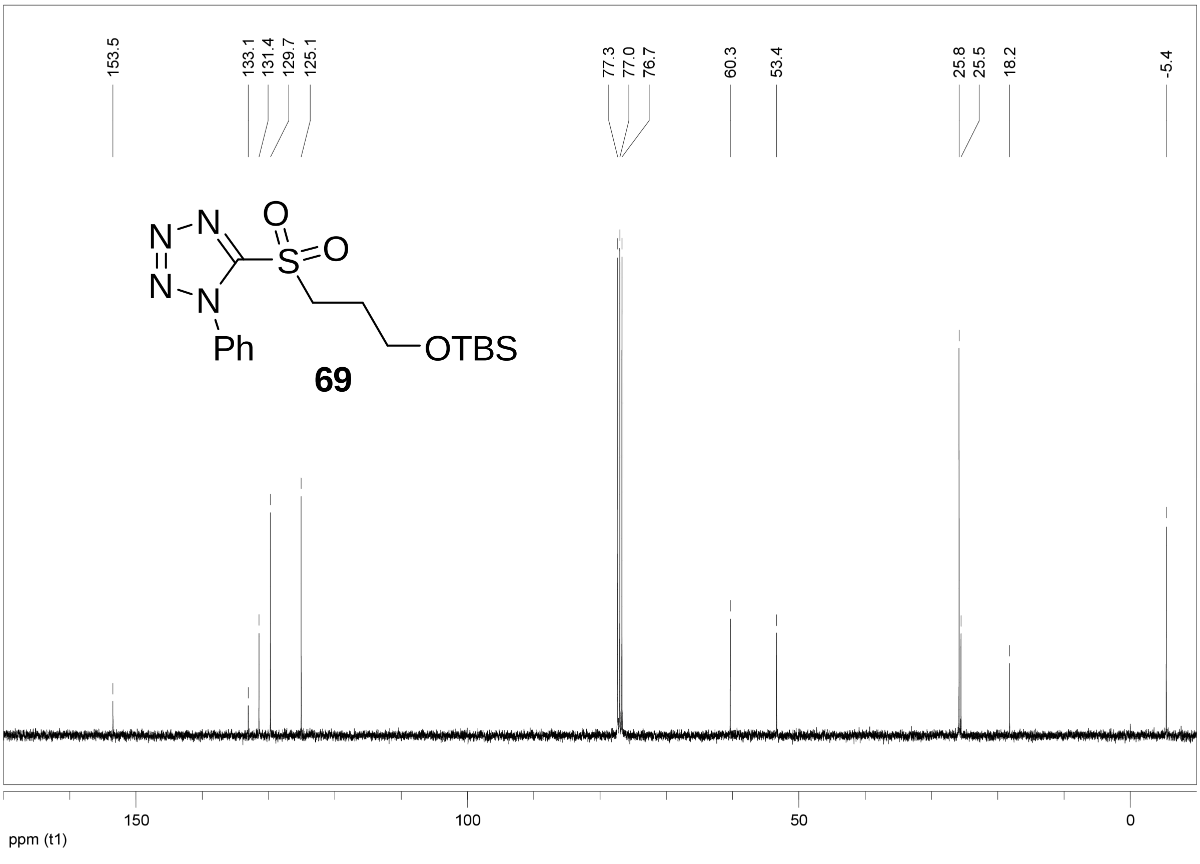

Figure S47: ^{1}H- (400 MHz) and ^{13}C-NMR (100 MHz) spectra of 3-((1-phenyl-1*H*-tetrazol-5-yl)sulfonyl)propan-1-ol tert-butyldimethylsilyl ether (**69**)

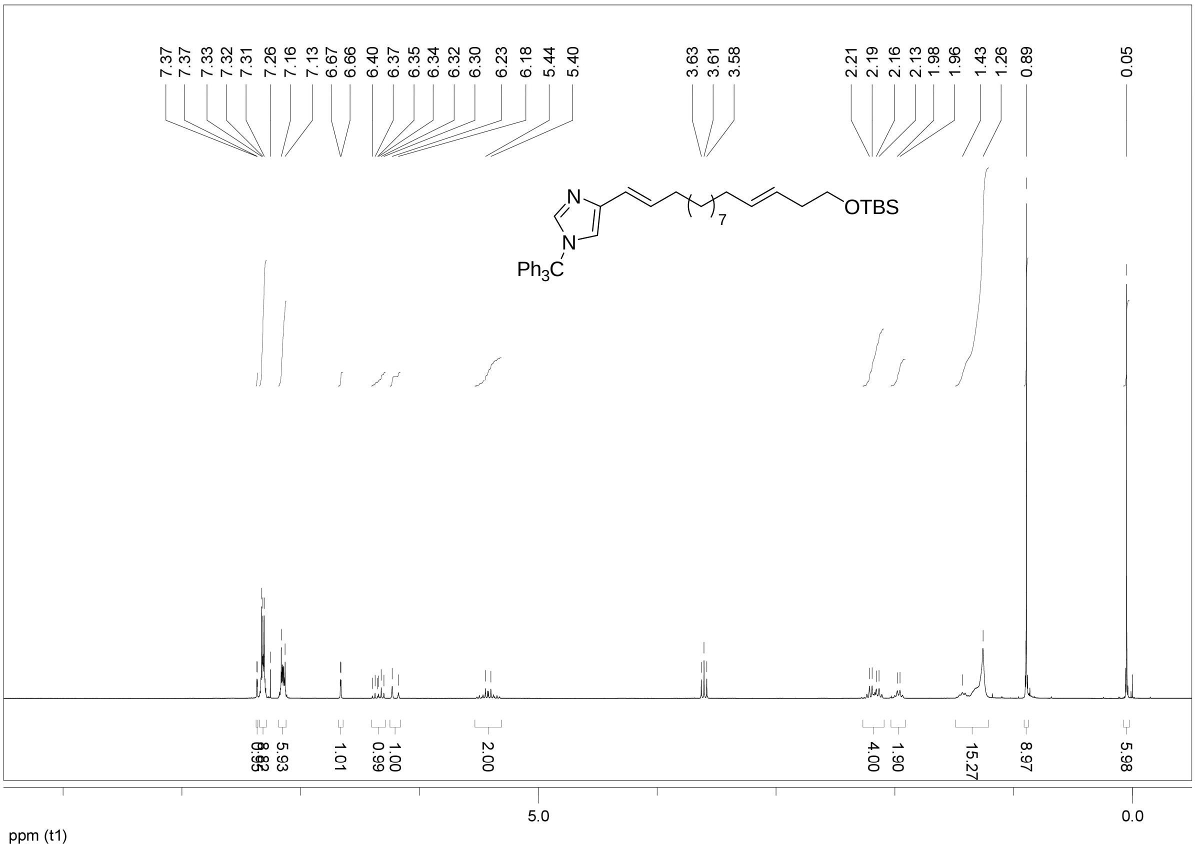

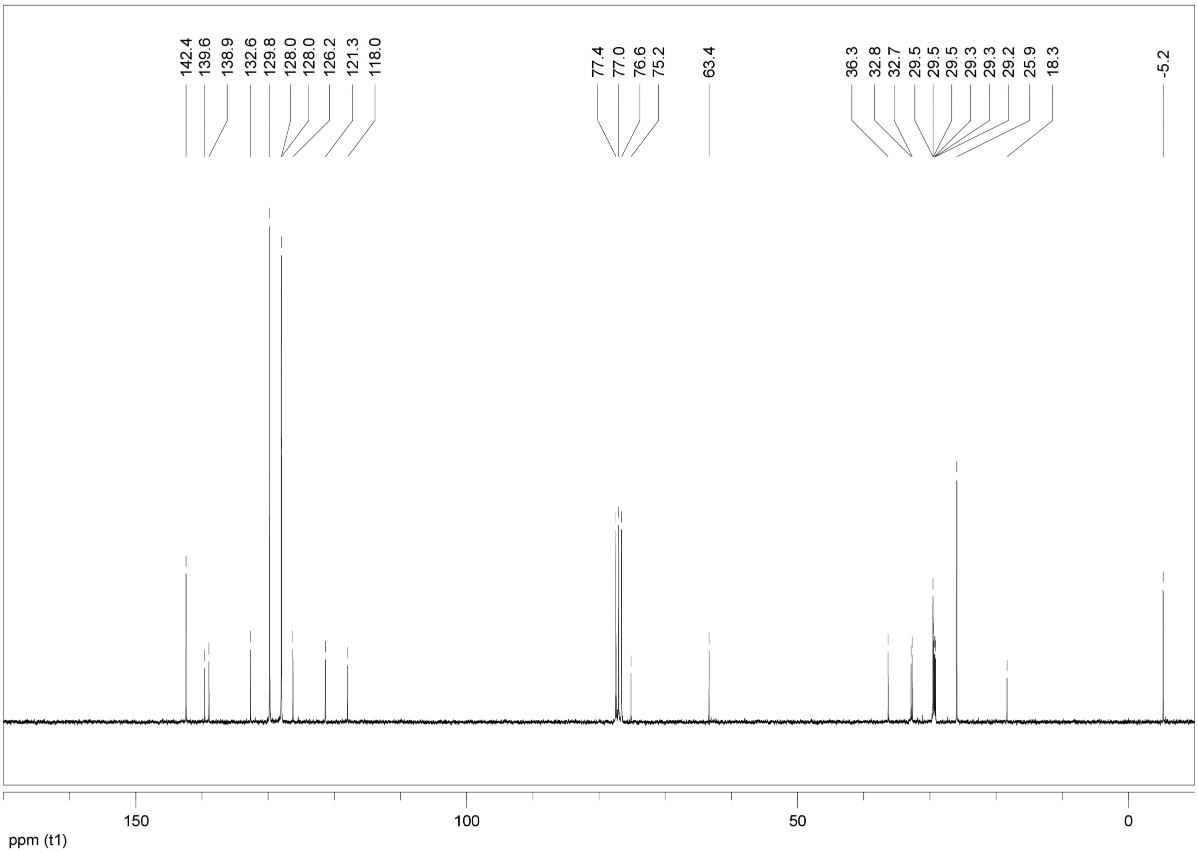

Figure S48: ^{1}H- (300 MHz) and ^{13}C-NMR (75 MHz) spectra of 4-((1*E*,12*E*)-15-((*tert*-Butyldimethylsilyl)oxy)pentadeca-1,12-dien-1-yl)-1-trityl-1*H*-imidazole

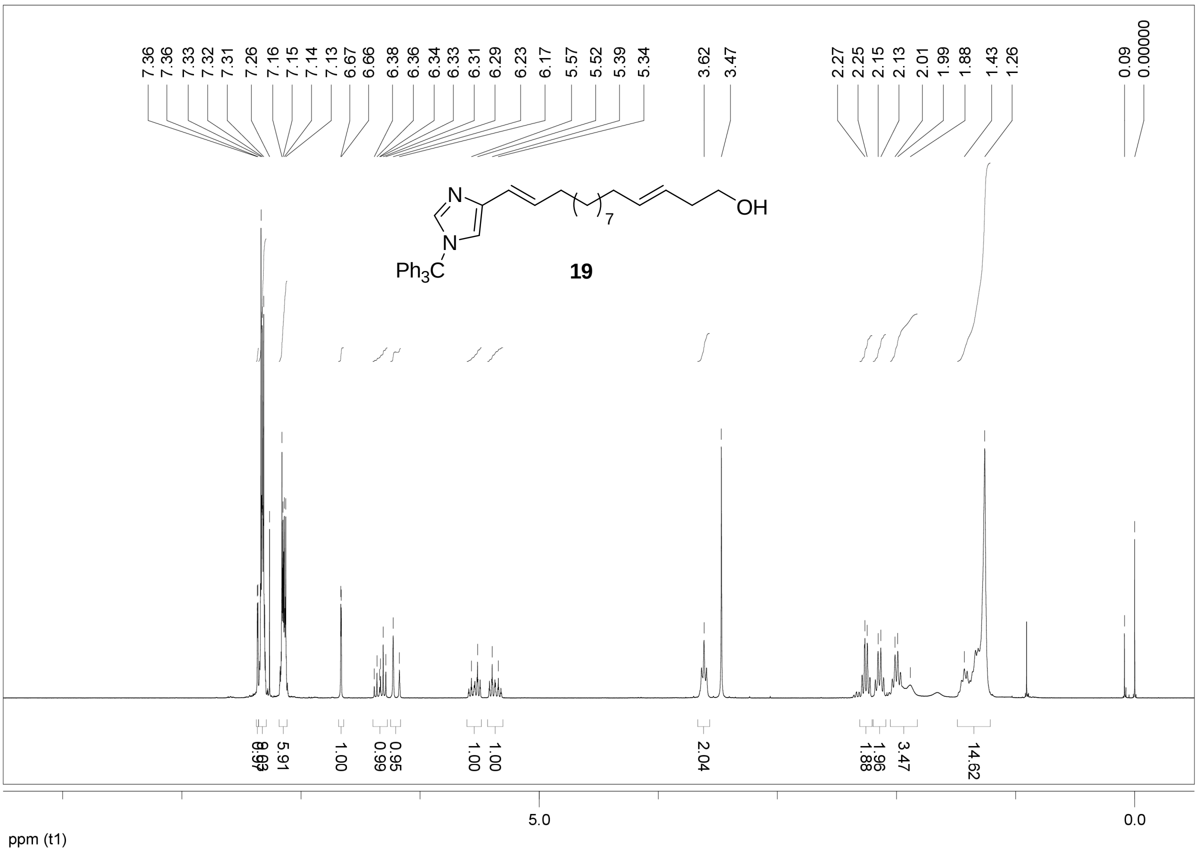

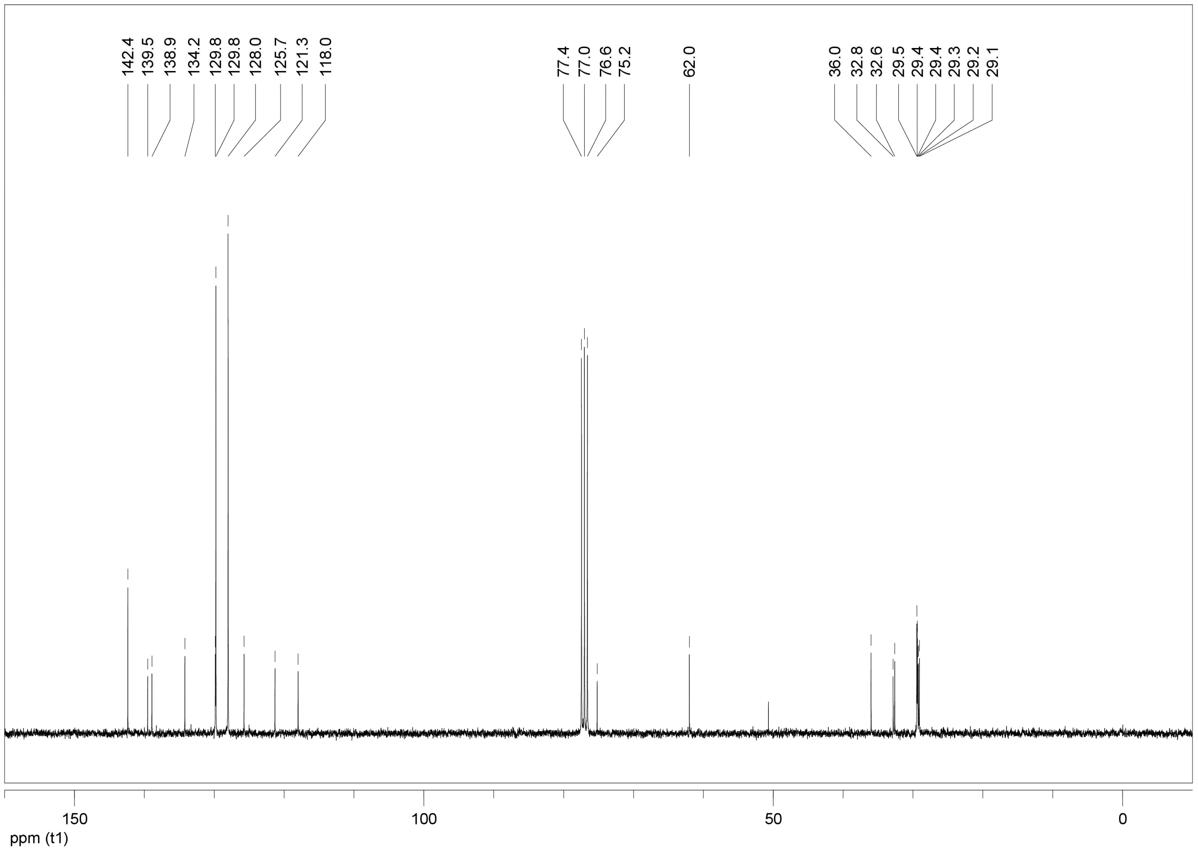

Figure S49: ^{1}H- (300 MHz) and ^{13}C-NMR (75 MHz) spectra of (3*E*,14*E*)-15-(1-trityl-1*H*-imidazol-4-yl)pentadeca-3,14-dien-1-ol (**19**)

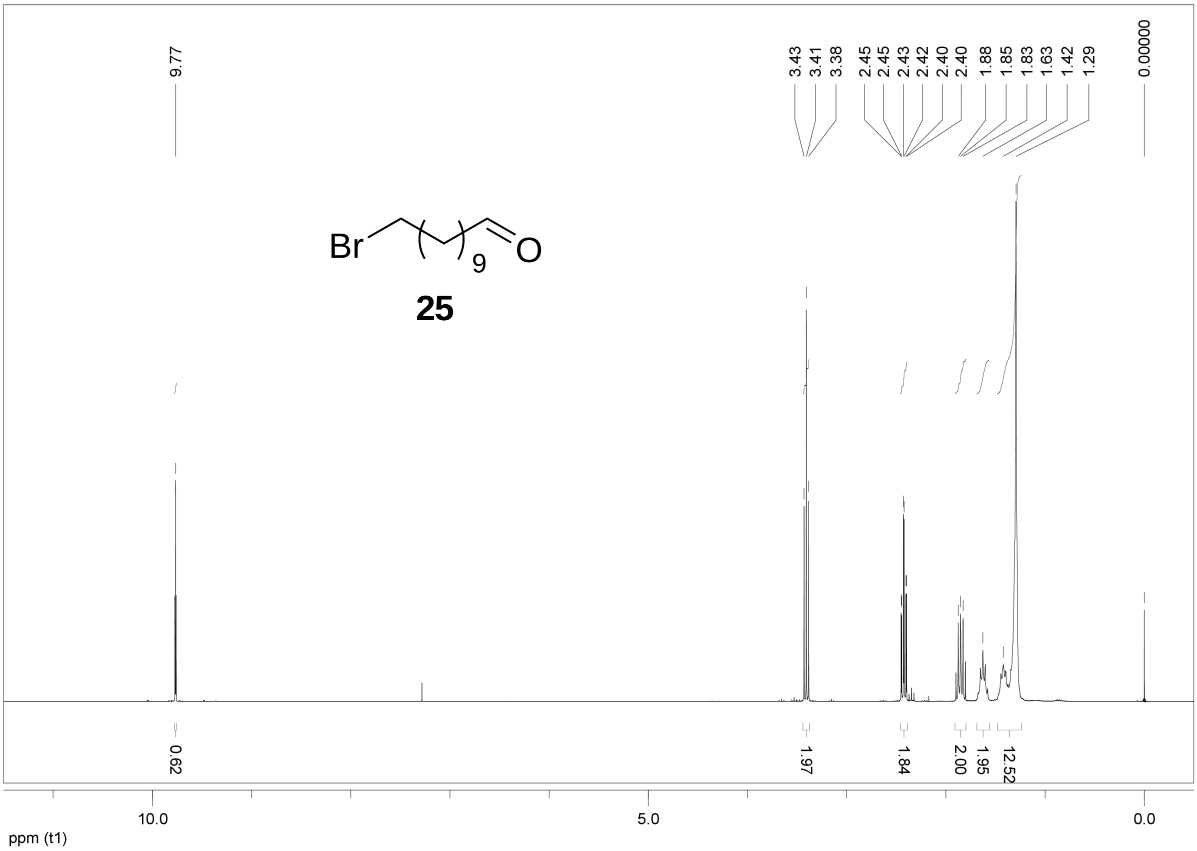

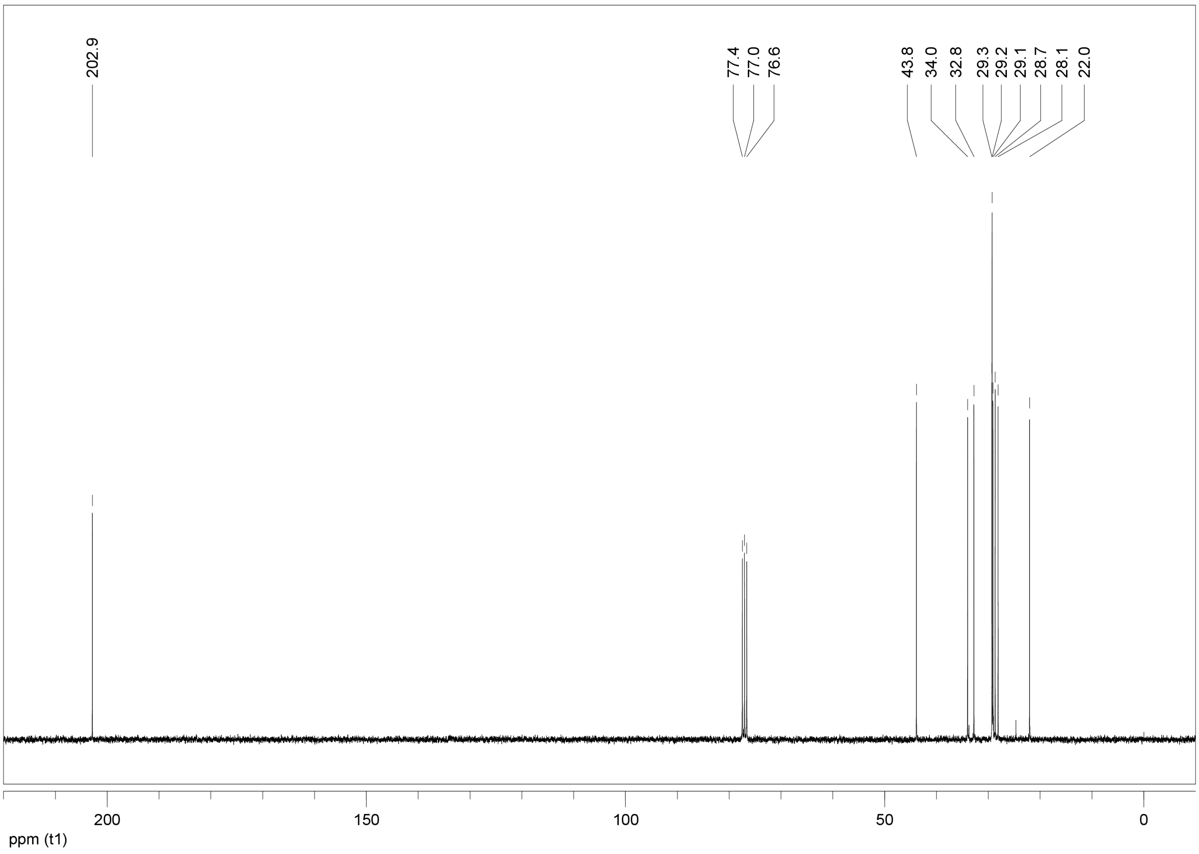

Figure S50: ^{1}H- (300 MHz) and ^{13}C-NMR (75 MHz) spectra of 11-bromoundecanal (**25**)

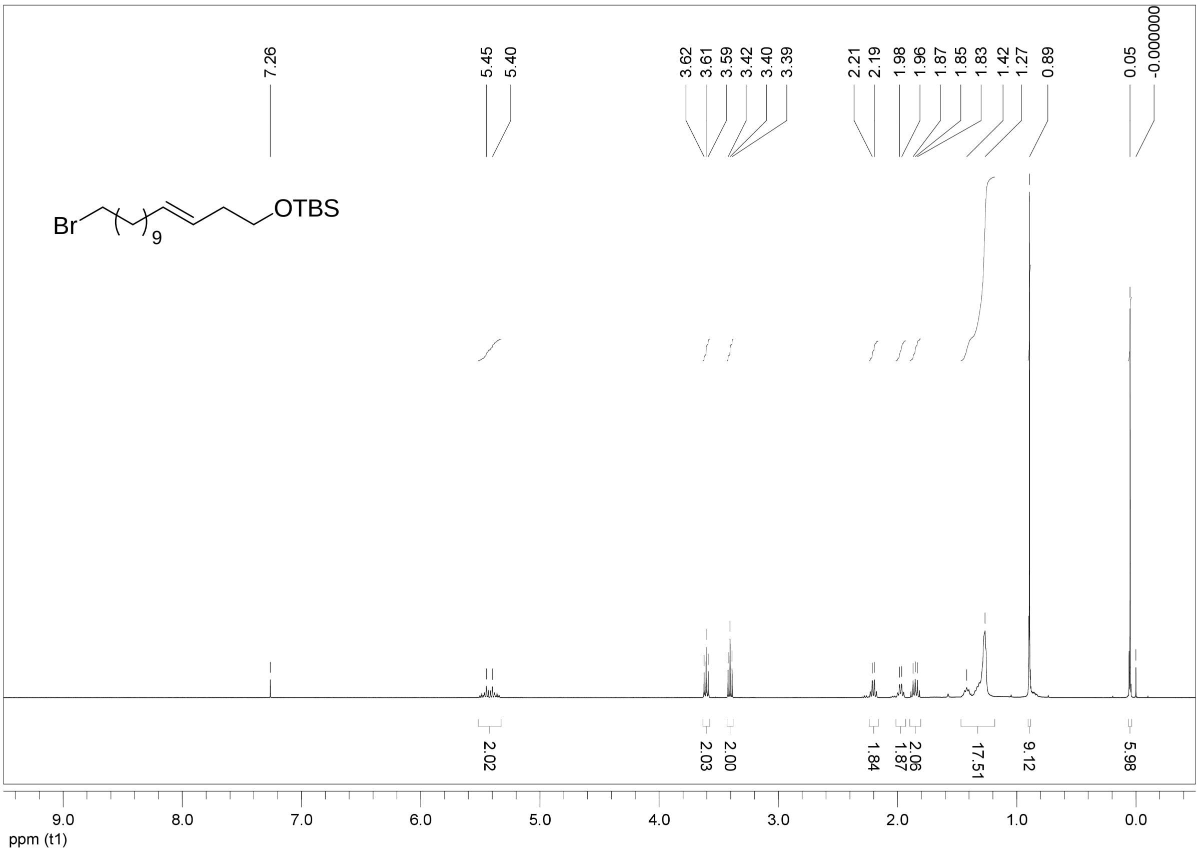

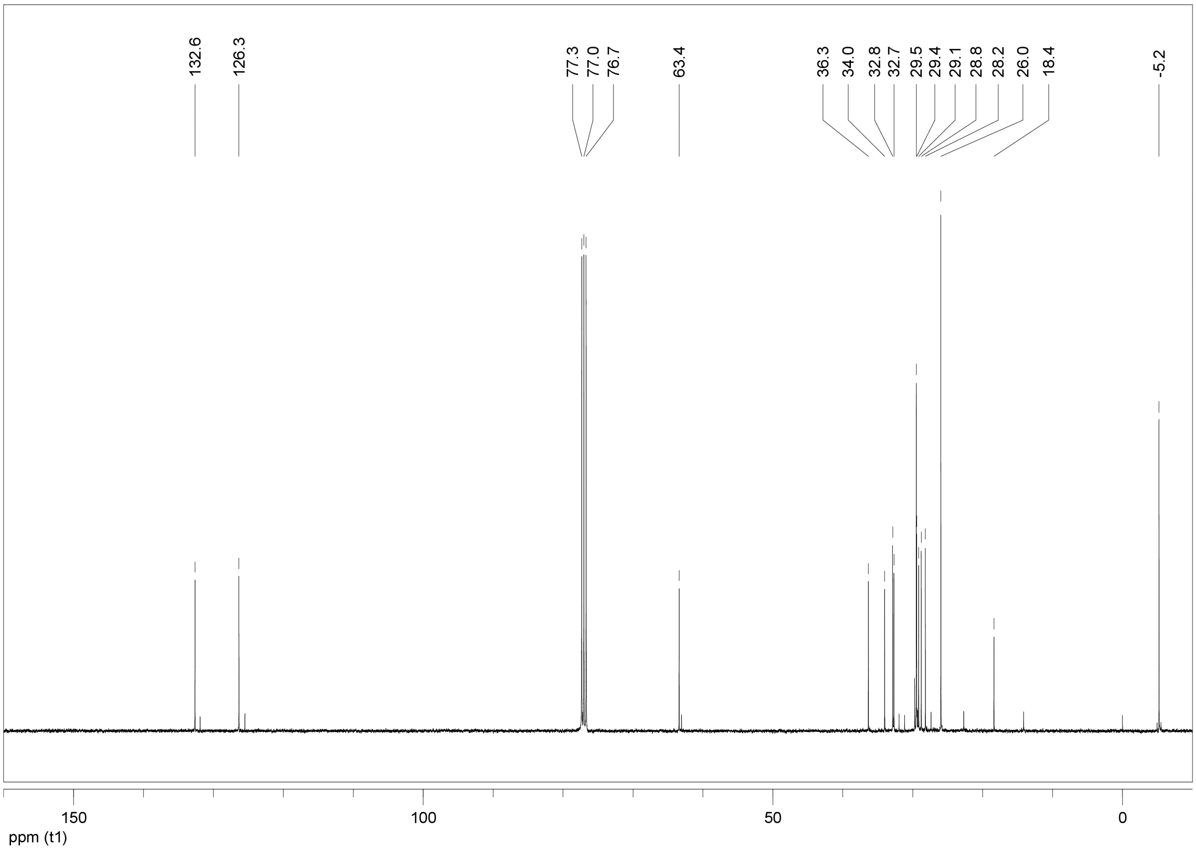

Figure S51: ^{1}H- (400 MHz) and ^{13}C-NMR (100 MHz) spectra of (*E*)-((14-Bromotetradec-3-en-1-yl)oxy)(tert-butyl)dimethylsilane

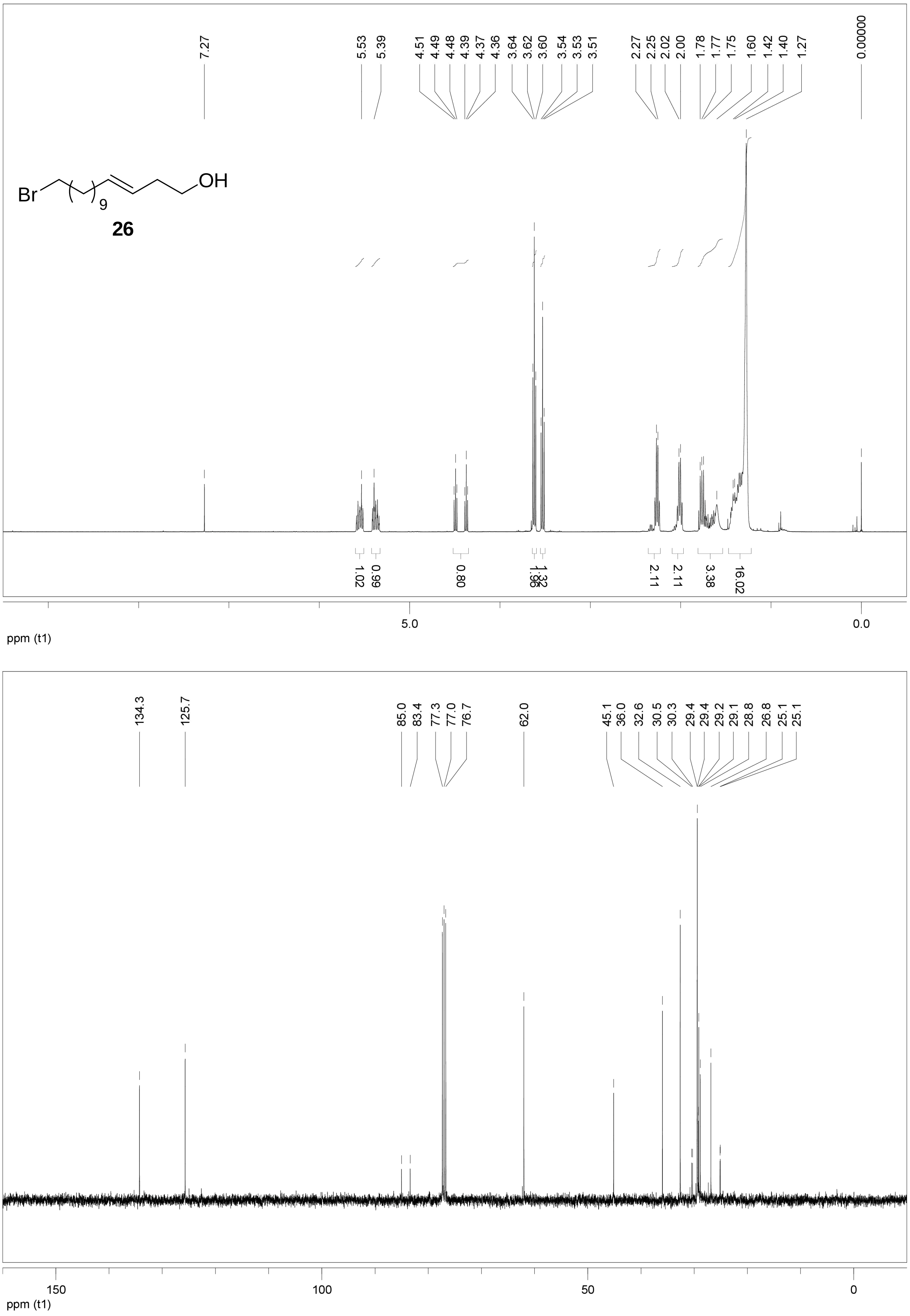

Figure S52: ^{1}H- (400 MHz) and ^{13}C-NMR (100 MHz) spectra of (*E*)-14-bromotetradec-3-en-1-ol (**26**)

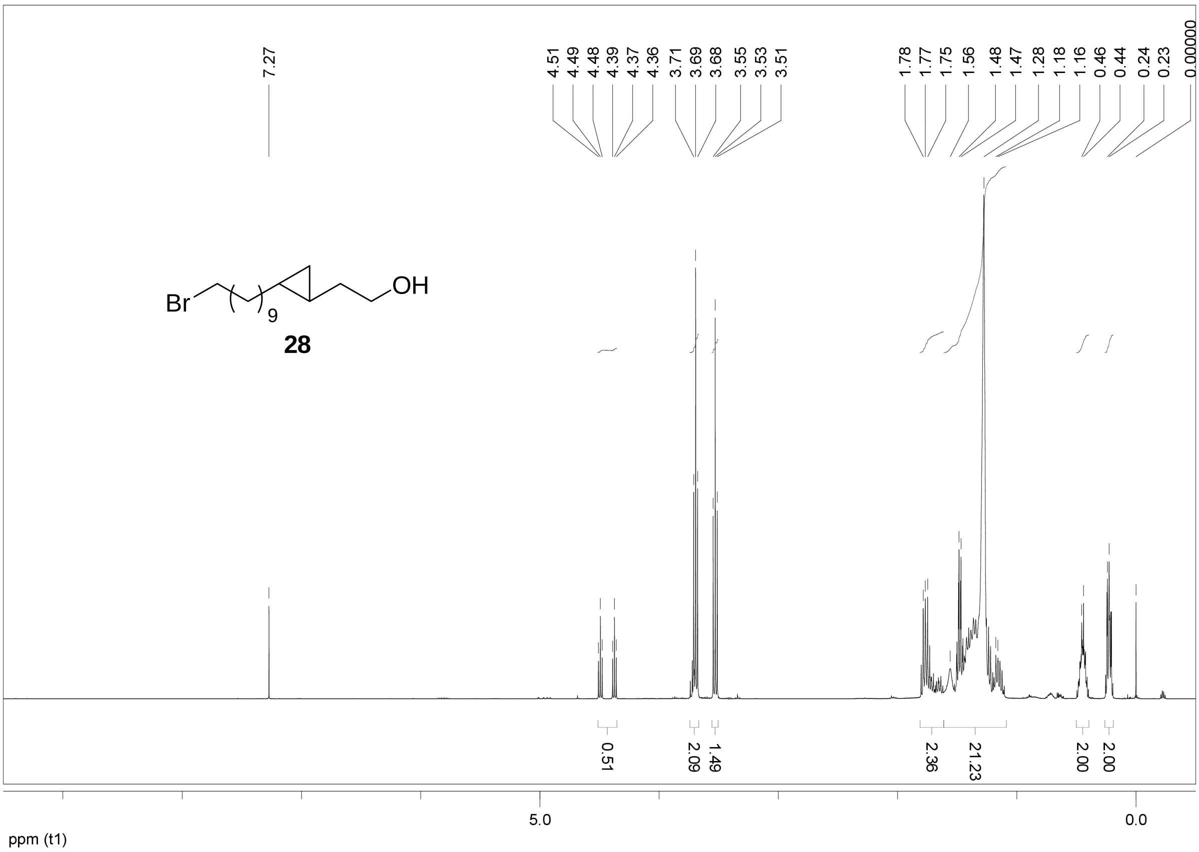

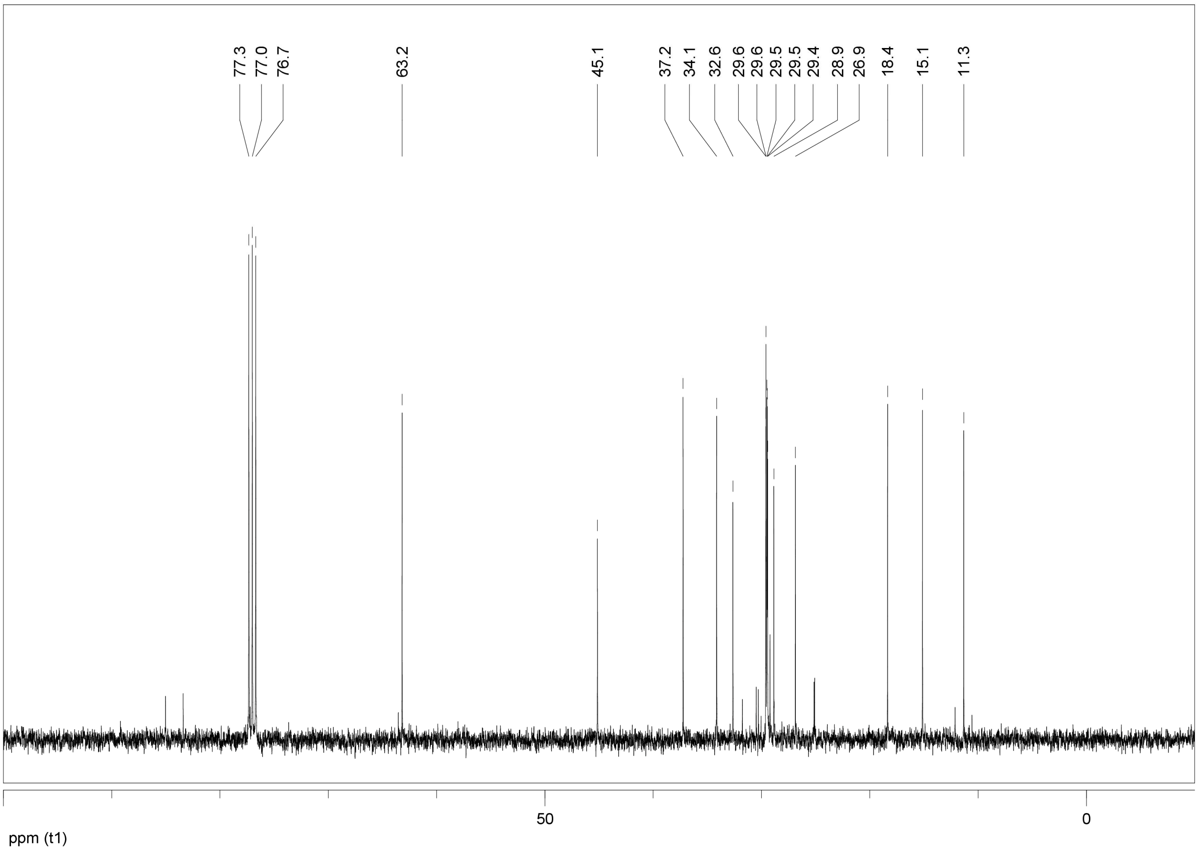

Figure S53: ^{1}H- (400 MHz) and ^{13}C-NMR (100 MHz) spectra of 2-((1*RS*,2*SR*)-(2-(10-bromodecyl)cyclopropyl)ethan-1-ol (**28**)

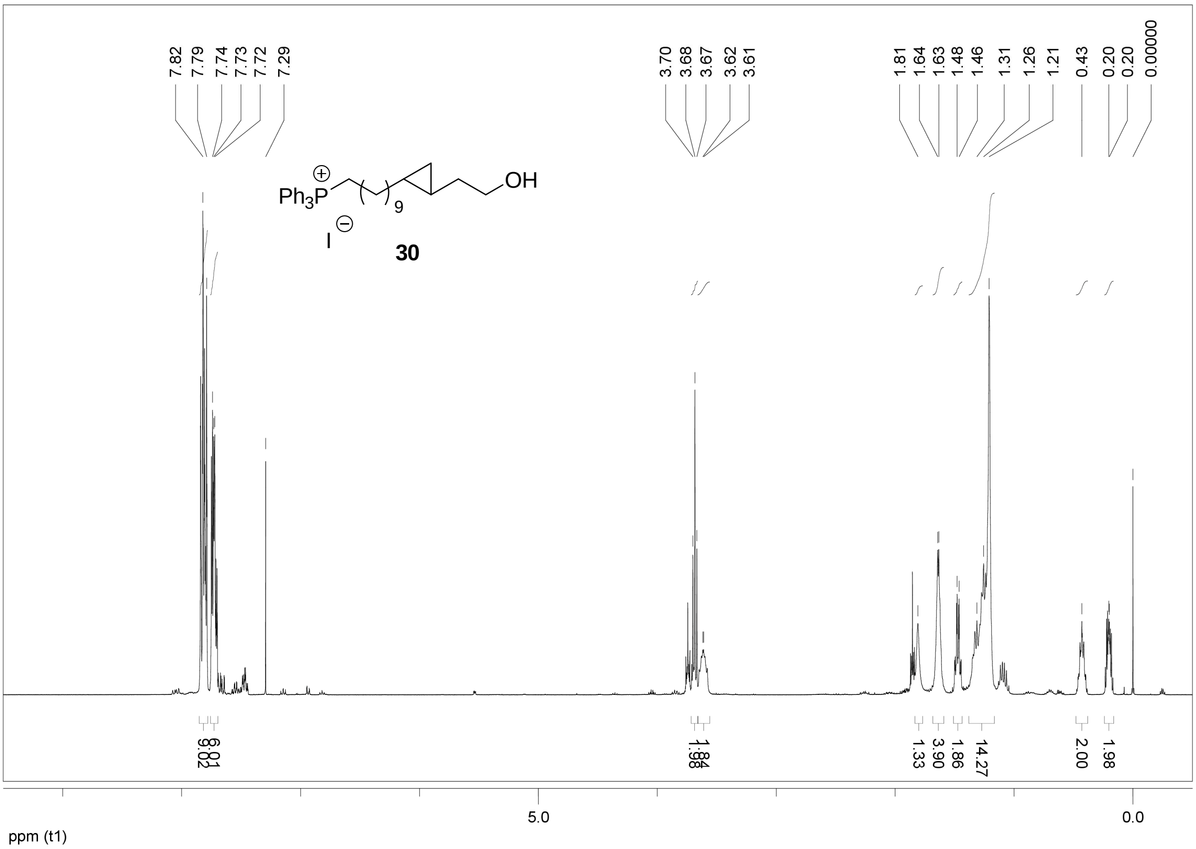

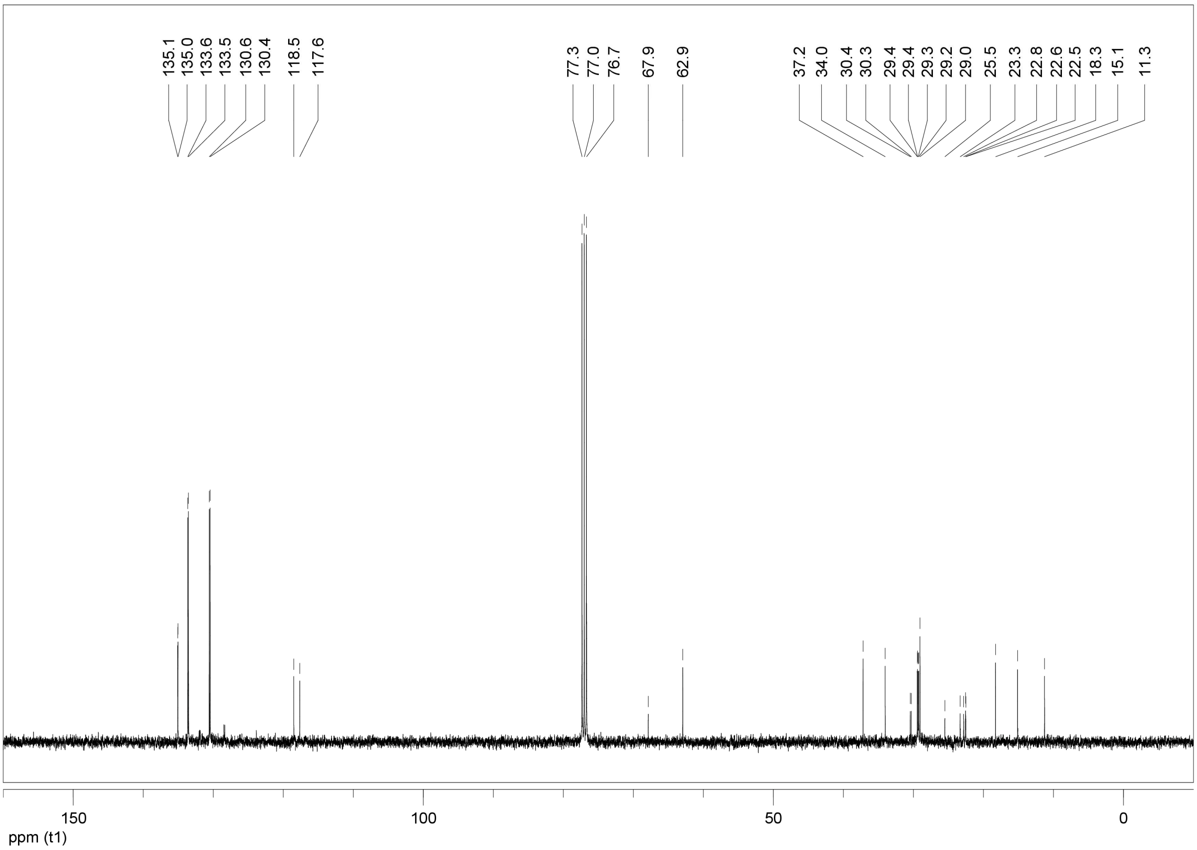

Figure S54: ^{1}H- (400 MHz) and ^{13}C-NMR (100 MHz) spectra of (10-((1*RS*,2*SR*)- 2-(2-hydroxyethyl)cyclopropyl)decyl)triphenylphosphonium iodide (**30**)

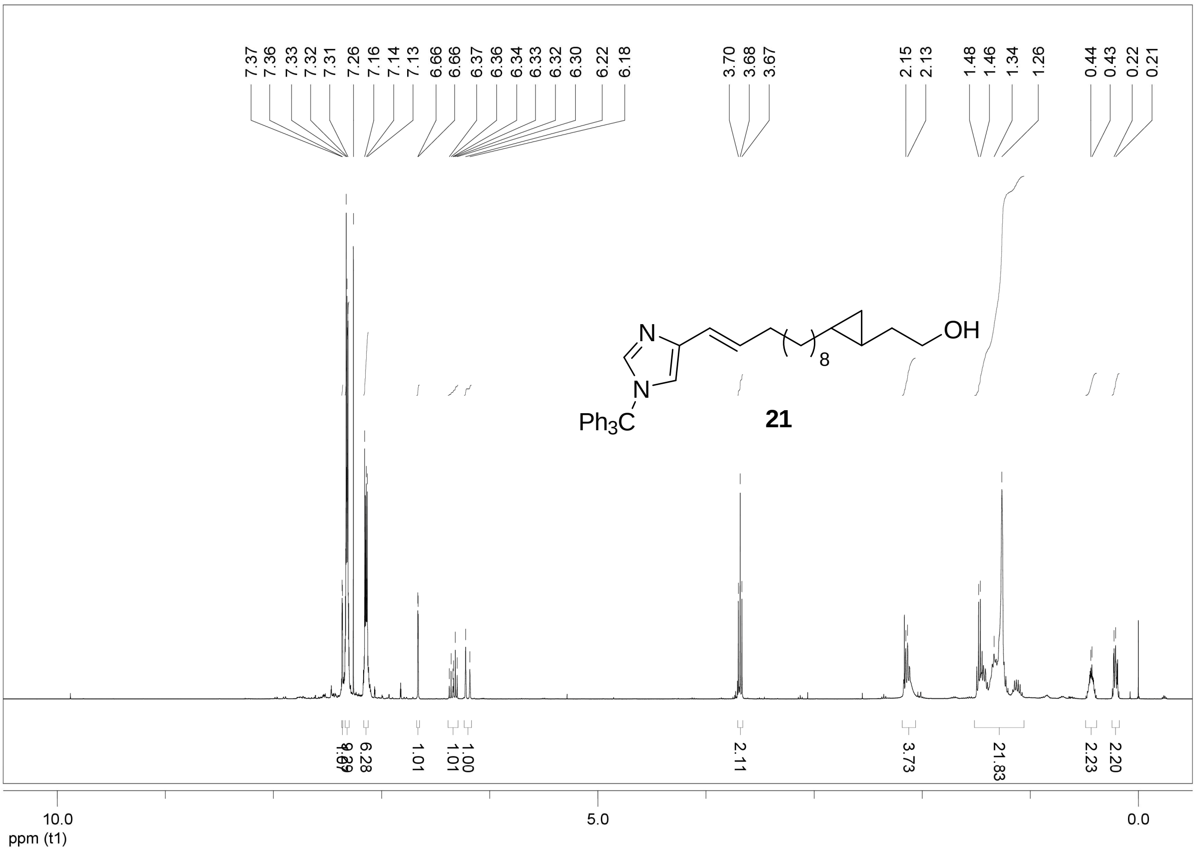

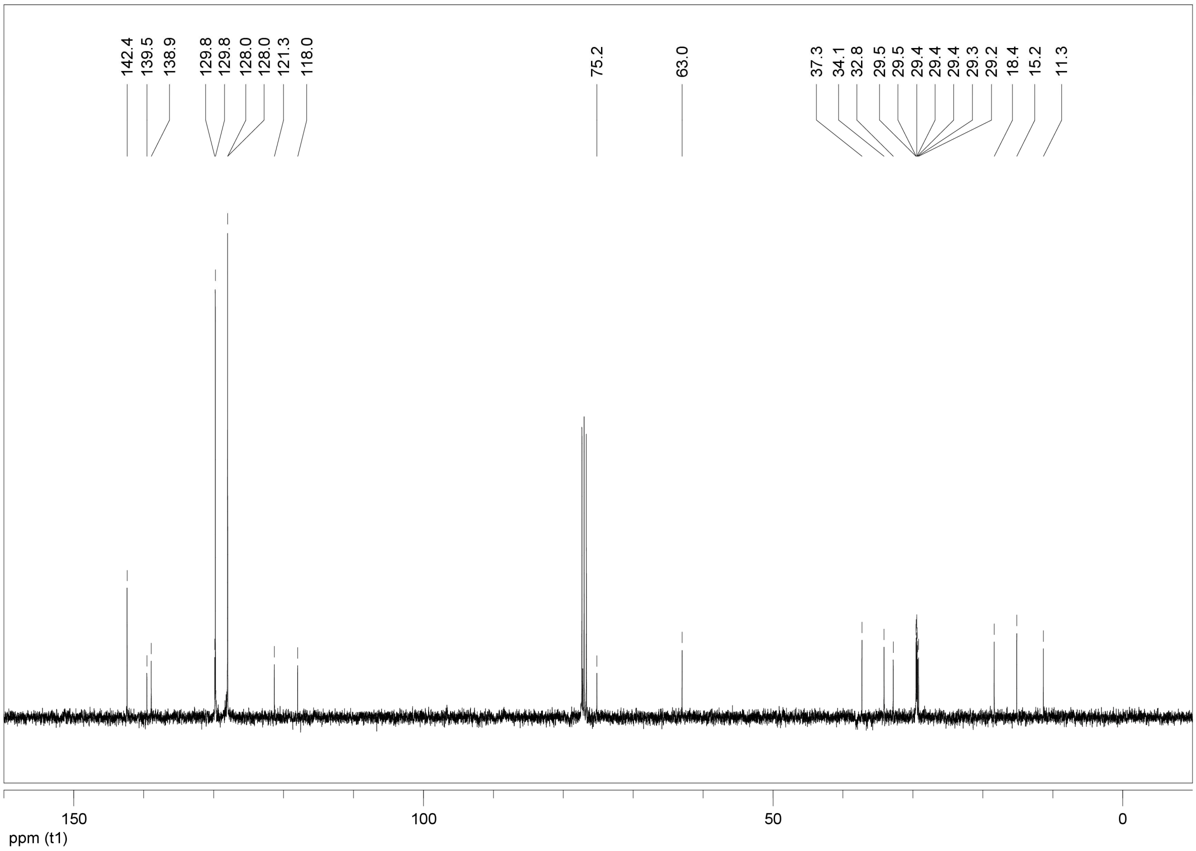

Figure S55: ^{1}H- (400 MHz) and ^{13}C-NMR (100 MHz) spectra of 2-((1*RS*,2*SR*)-2-((*E*)-11-(1-trityl-1*H*-imidazol-4-yl)undec-10-en-1-yl)cyclopropyl)-ethan-1-ol (**21**)

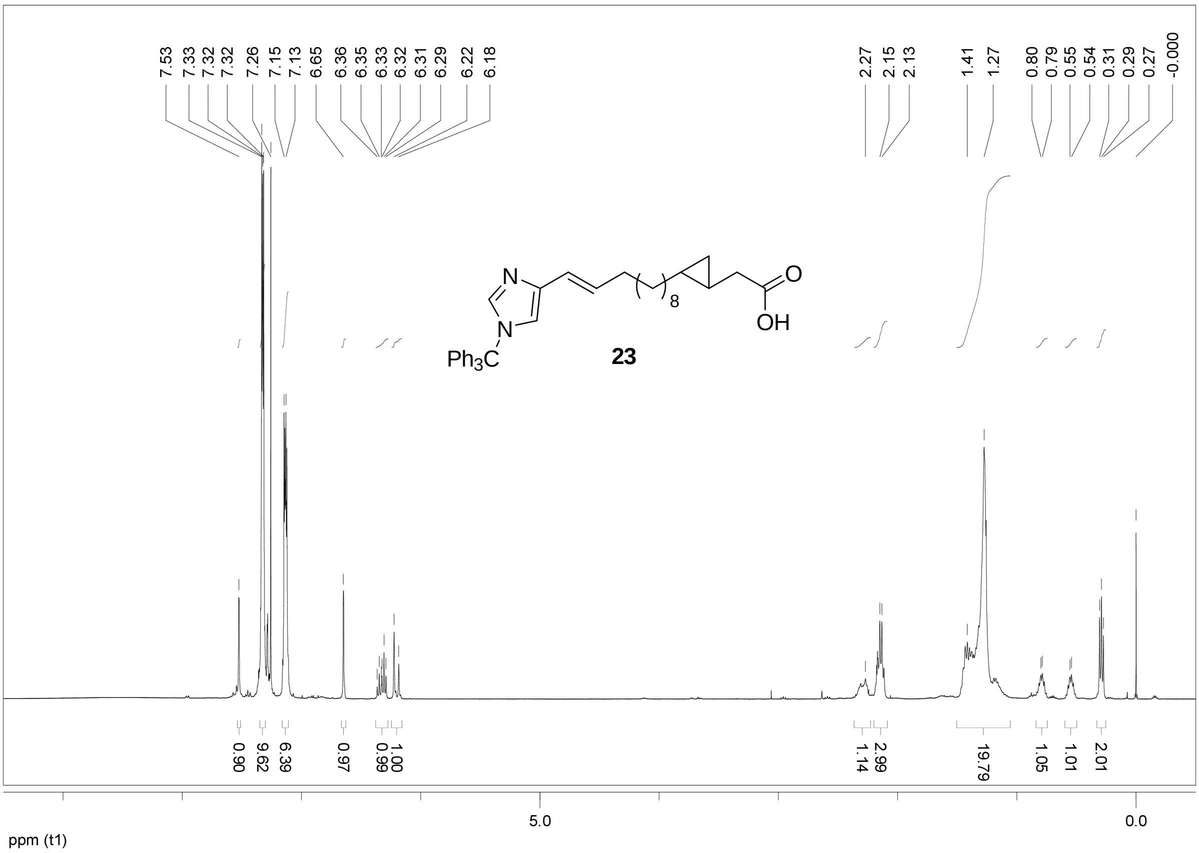

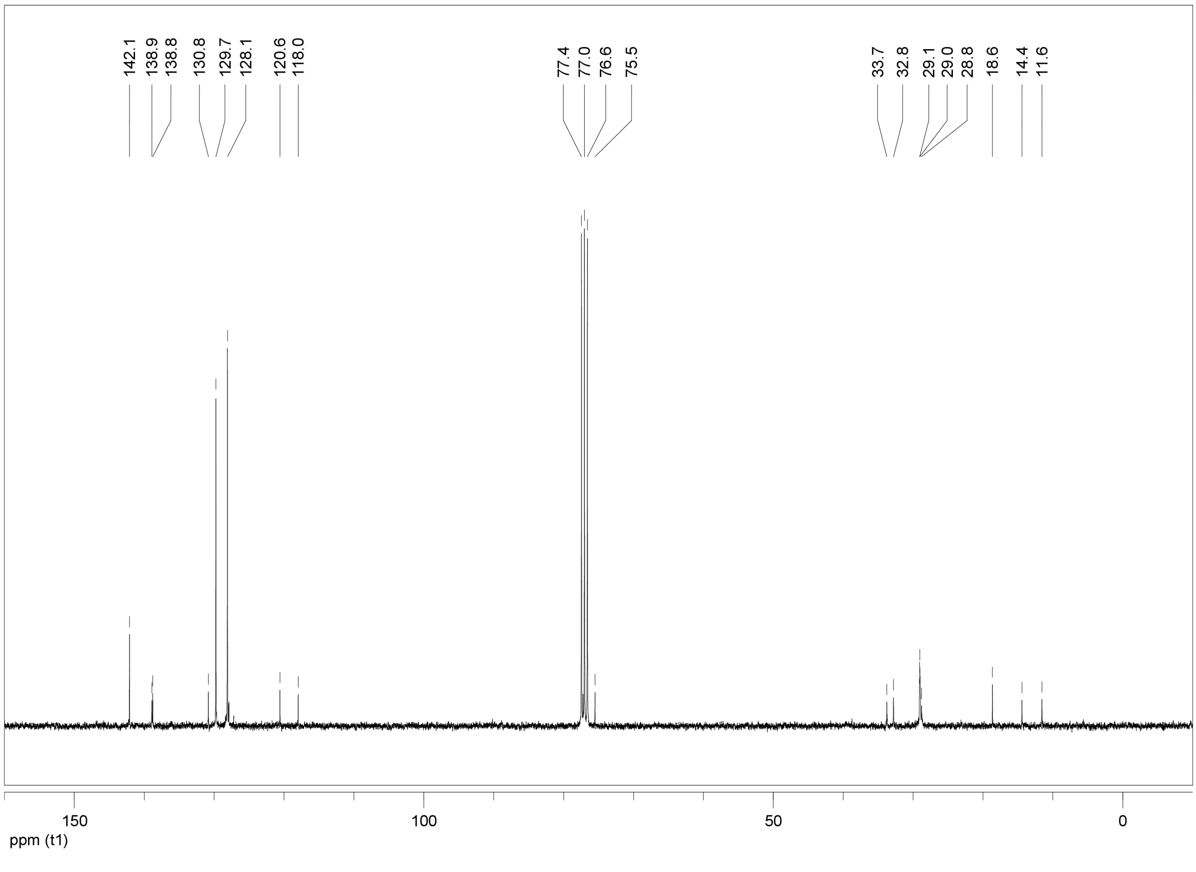

Figure S56: ^{1}H- (400 MHz) and ^{13}C-NMR (100 MHz) spectra of 2-((1*RS*,2*SR*)-2-((*E*)-11-(1-trityl-1*H*-imidazol-4-yl)undec-10-en-1-yl)cyclopropyl)-acetic acid (**23**)

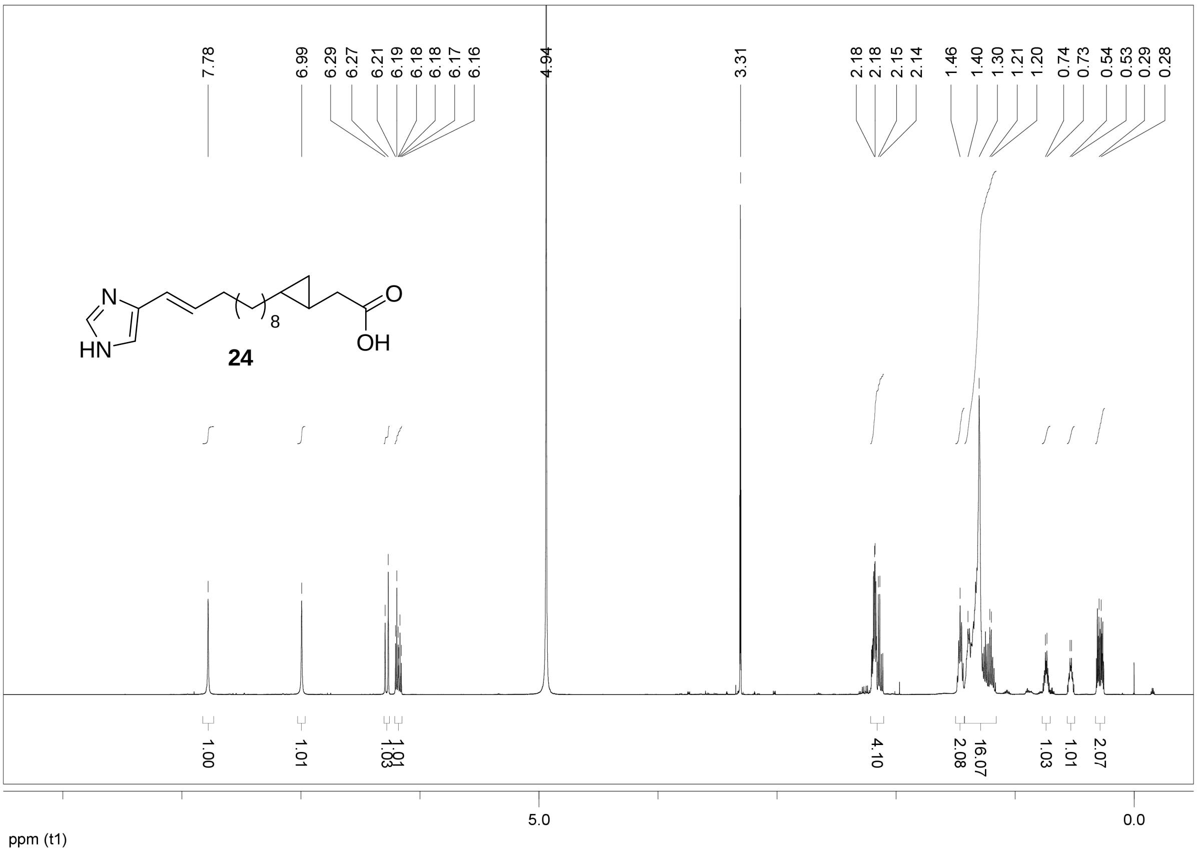

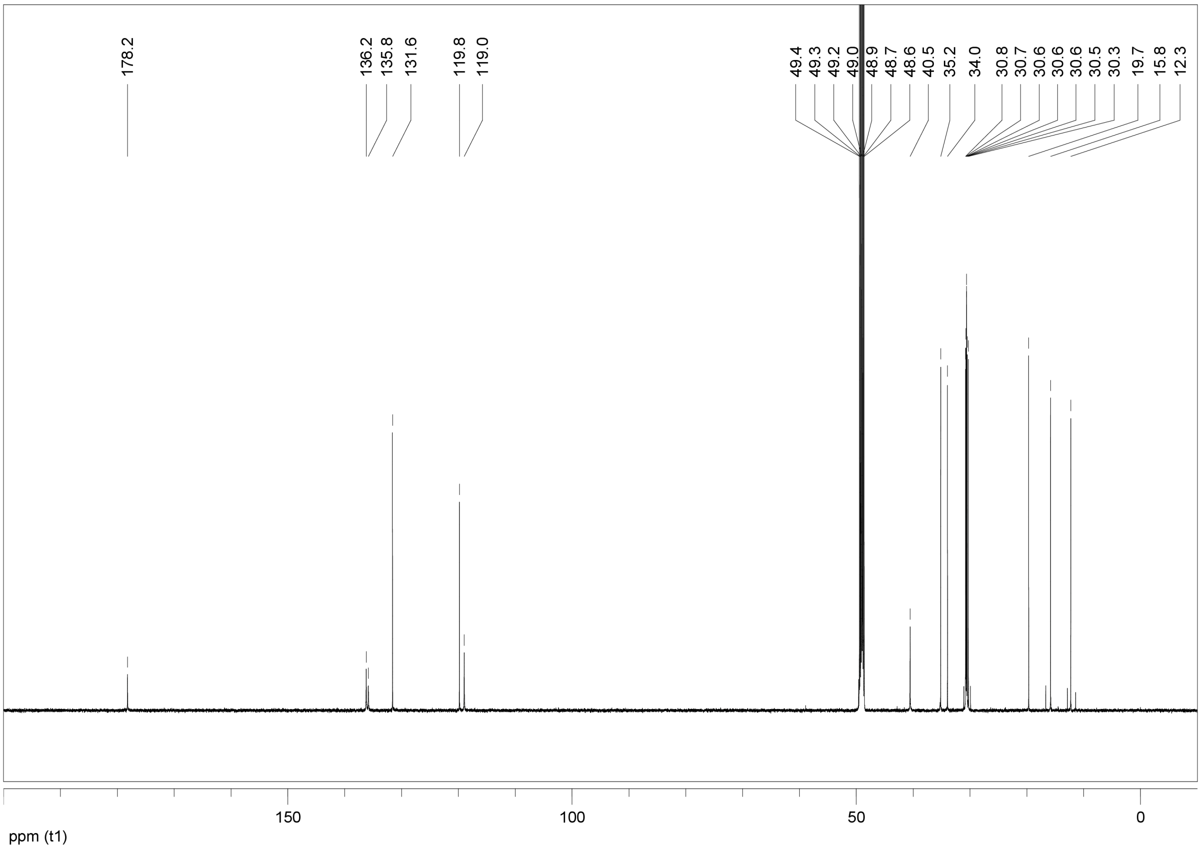

Figure S57: ^{1}H- (600 MHz) and ^{13}C-NMR (150 MHz) spectra of 2-((1*RS*,2*SR*)-2-((*E*)-11-(1*H*-imidazol-4-yl)undec-10-en-1-yl)cyclopropyl)acetic acid, imidacin B1 (**24**)

Synthesis of imidacin A2

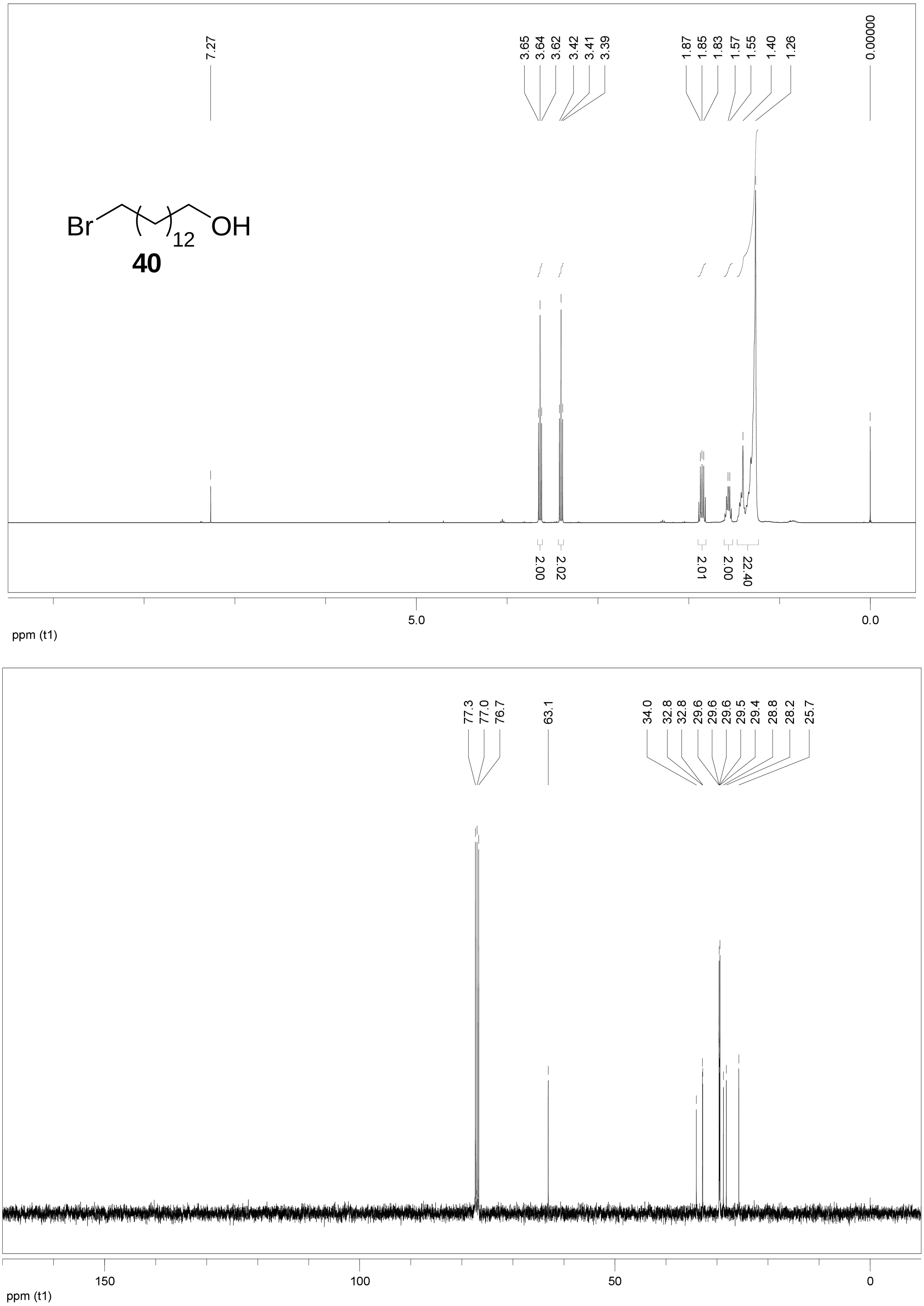

Figure S58: ^{1}H- (400 MHz) and ^{13}C-NMR (100 MHz) spectra of 14-Bromotetradecan-1-ol (**40**)

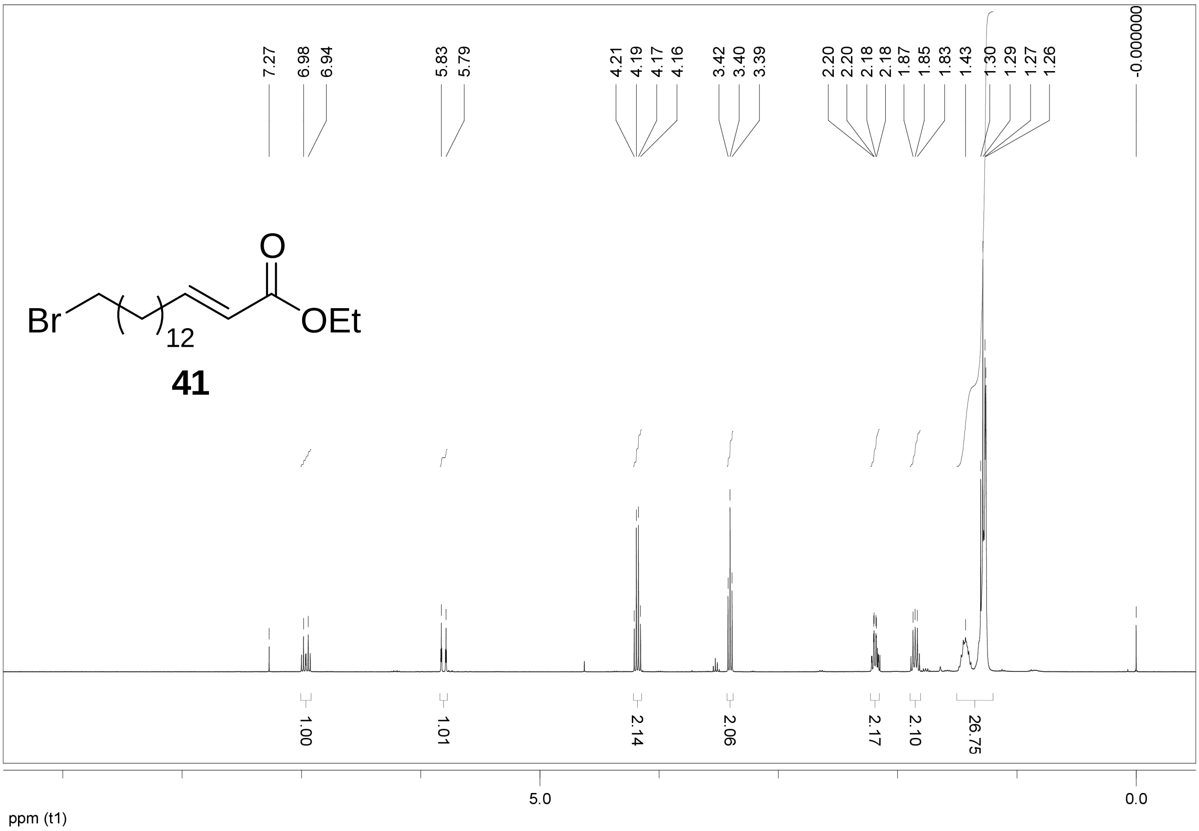

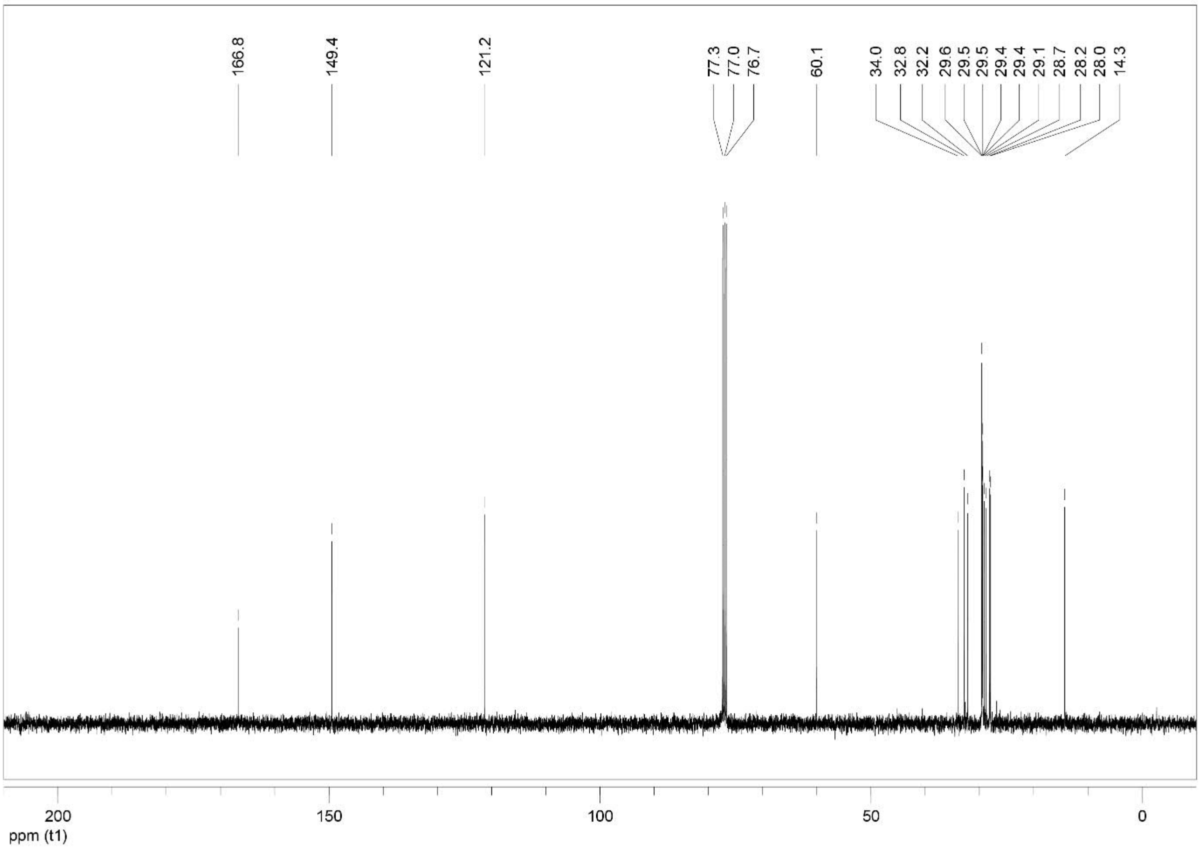

Figure S59: ^{1}H- (400 MHz) and ^{13}C-NMR (100 MHz) spectra of Ethyl (*E*)-16-bromehexadec-2-enoate (**41**)

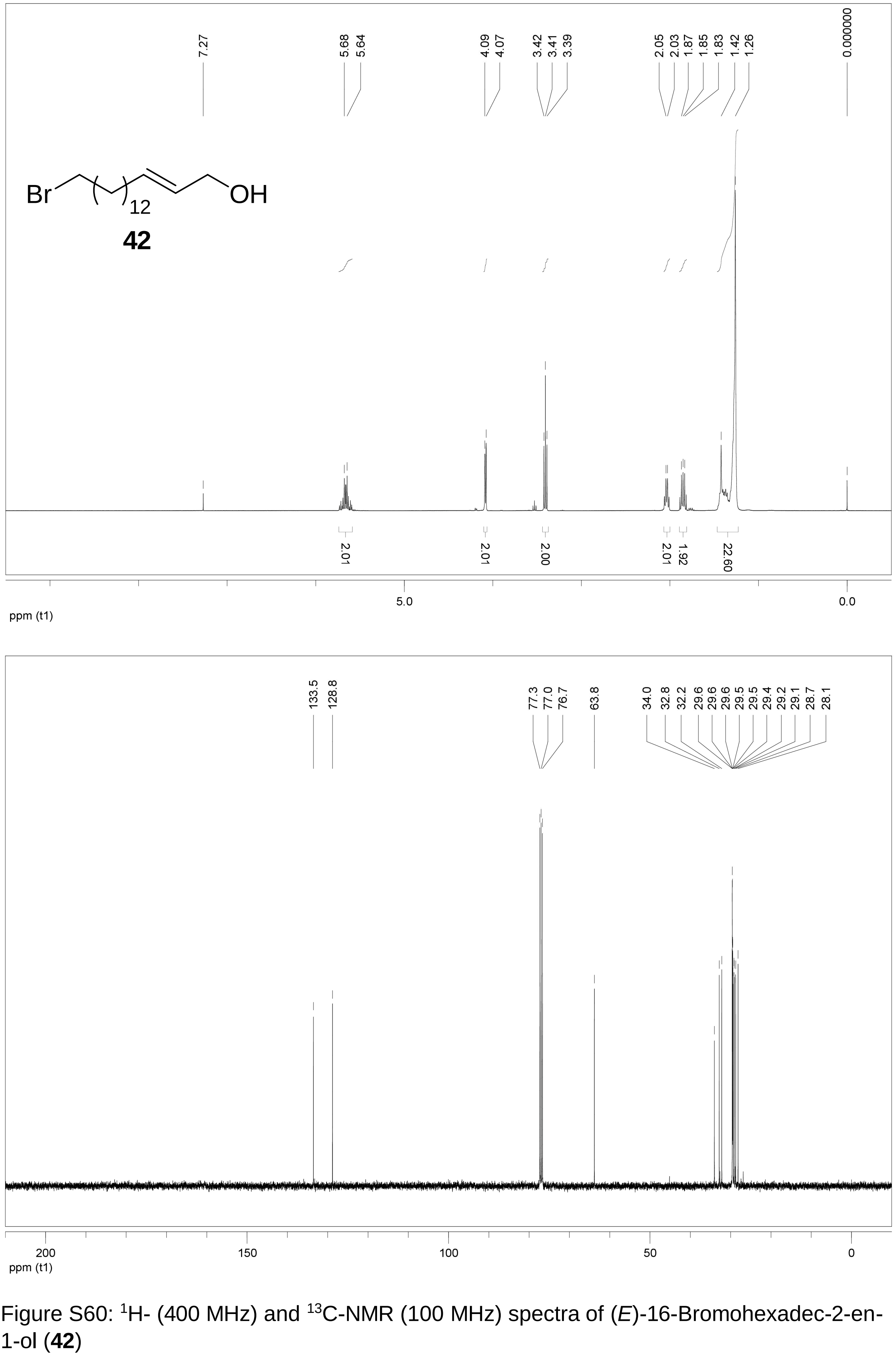

Figure S60: ^{1}H- (400 MHz) and ^{13}C-NMR (100 MHz) spectra of (*E*)-16-Bromohexadec-2-en-1-ol (**42**)

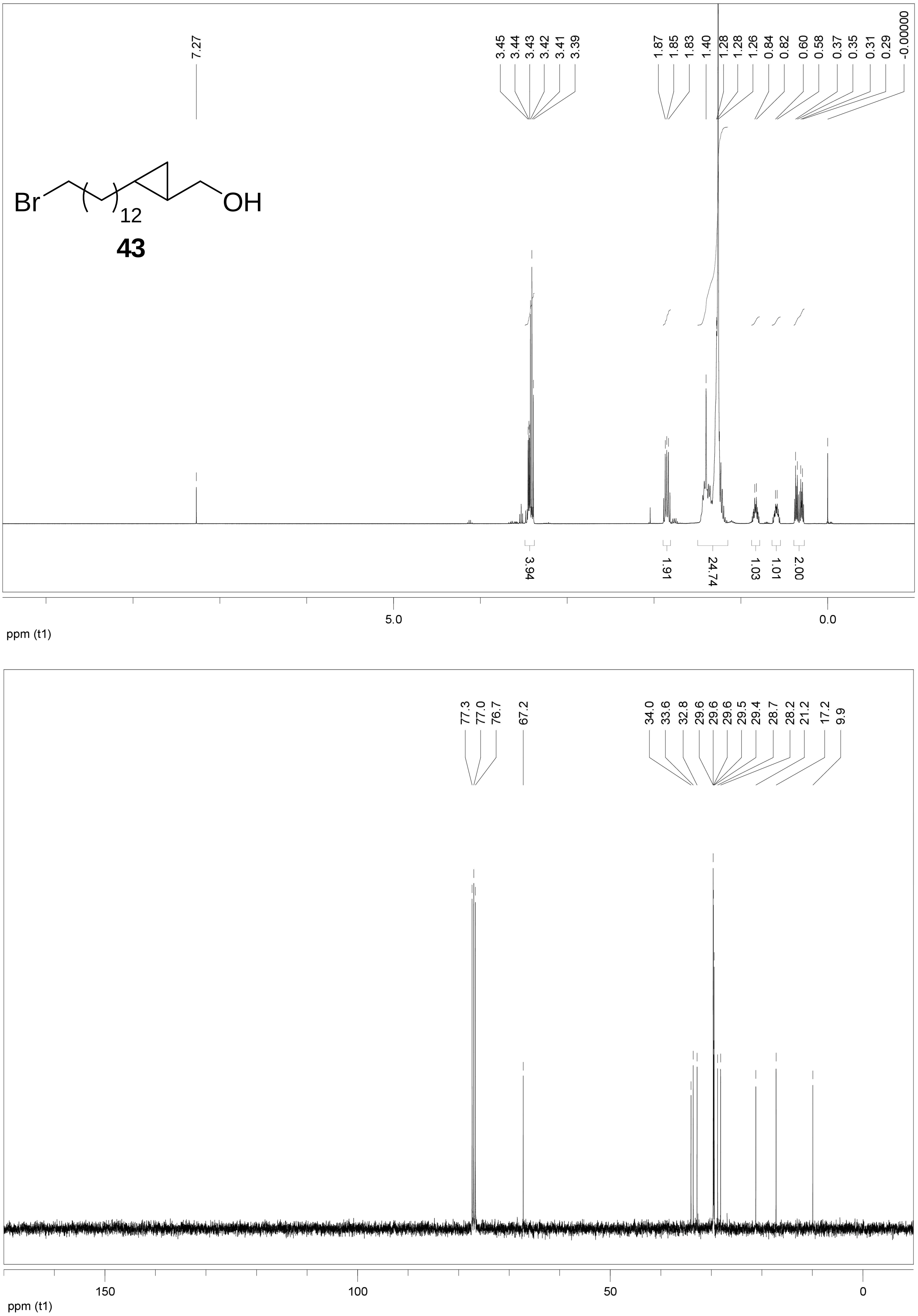

Figure S61: ^{1}H- (400 MHz) and ^{13}C-NMR (100 MHz) spectra of ((1*RS*,2*RS*)-2-(13-Bromotridecyl)cyclopropyl)methanol (**43**)

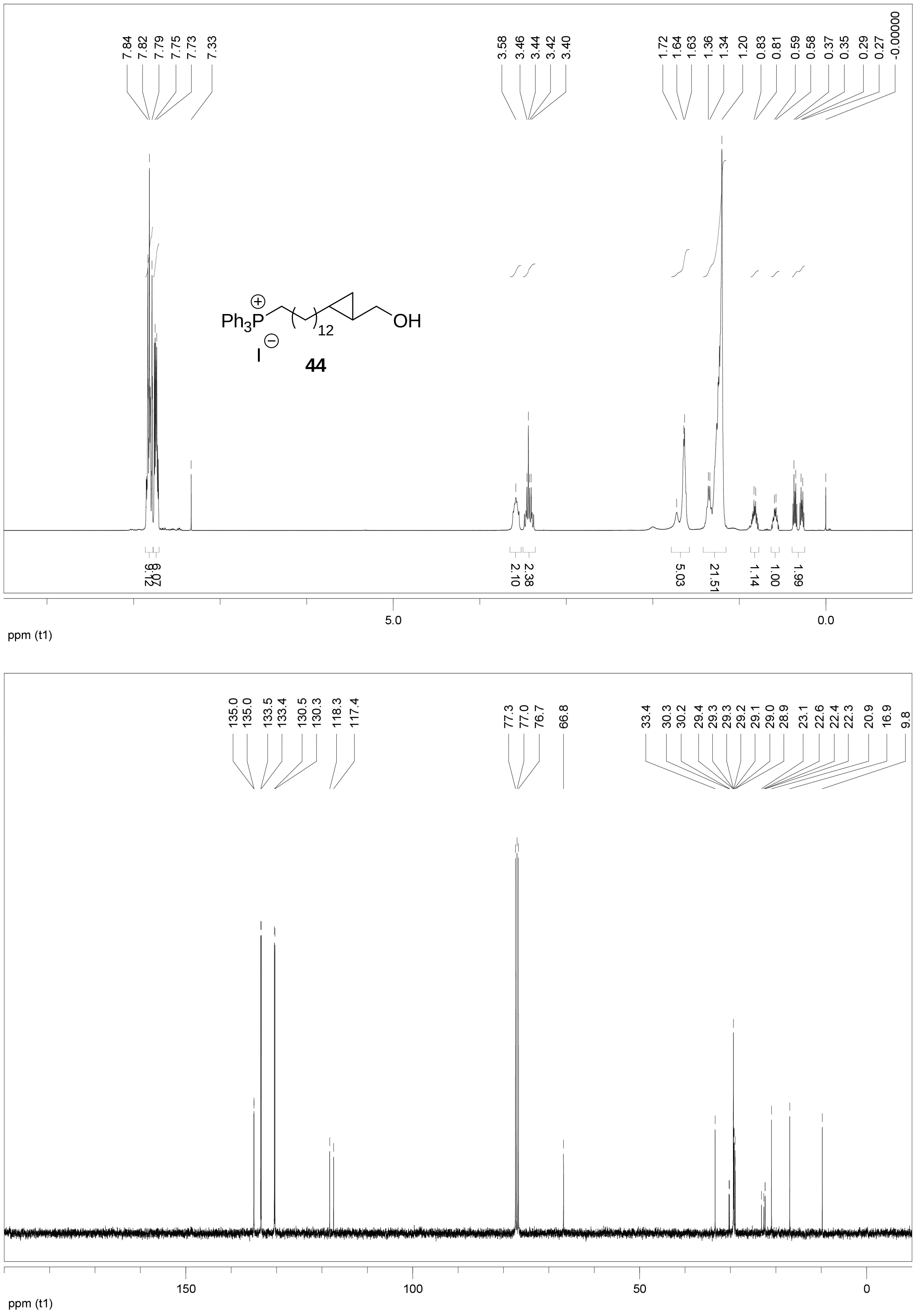

Figure S62: ^{1}H- (400 MHz) and ^{13}C-NMR (100 MHz) spectra of (13-((1*RS*,2*RS*)-2-(Hydroxymethyl)cyclopropyl)tridecyl)triphenylphosphonium iodide (**44**)

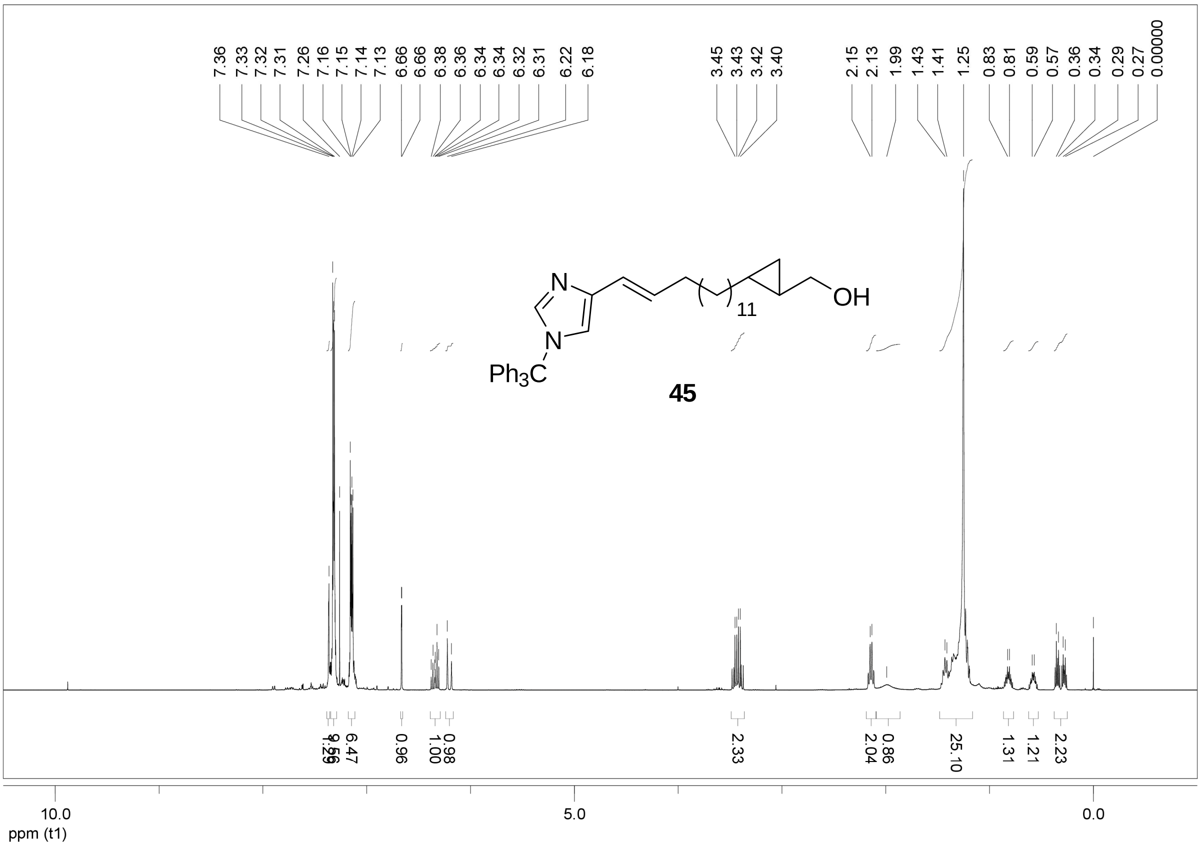

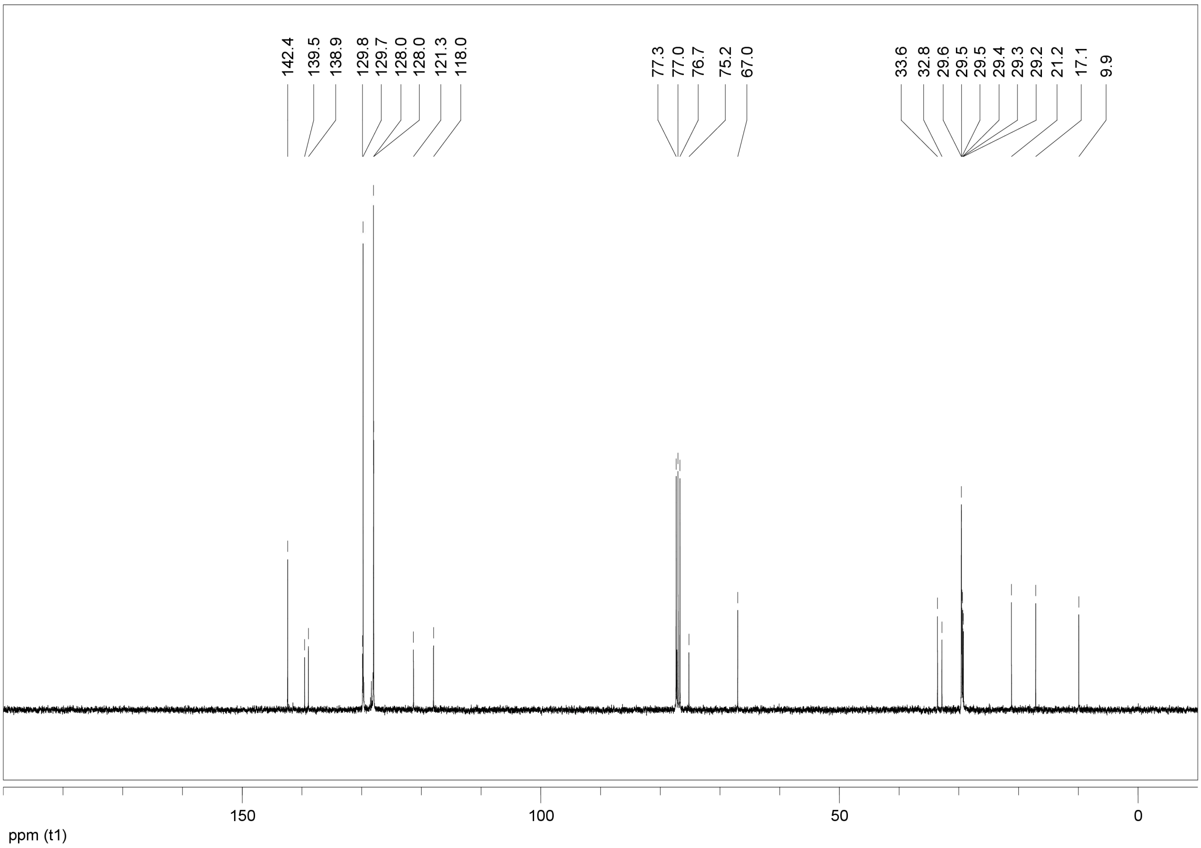

Figure S63: ^{1}H- (400 MHz) and ^{13}C-NMR (100 MHz) spectra of ((1*RS*,2*RS*)-2-((*E*)-14-(1-Trityl-1*H*-imidazol-4-yl)tetradec-13-en-1-yl)cyclopropyl)-methanol (**45**)

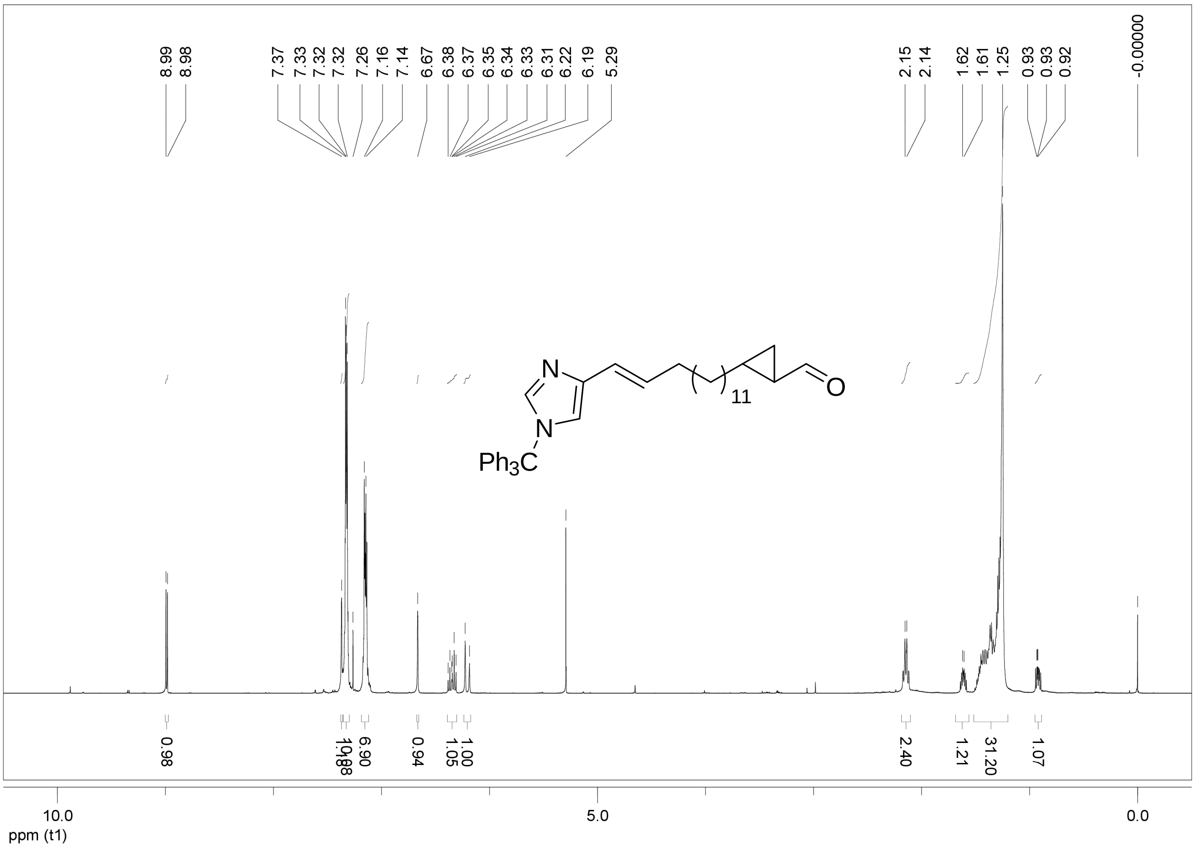

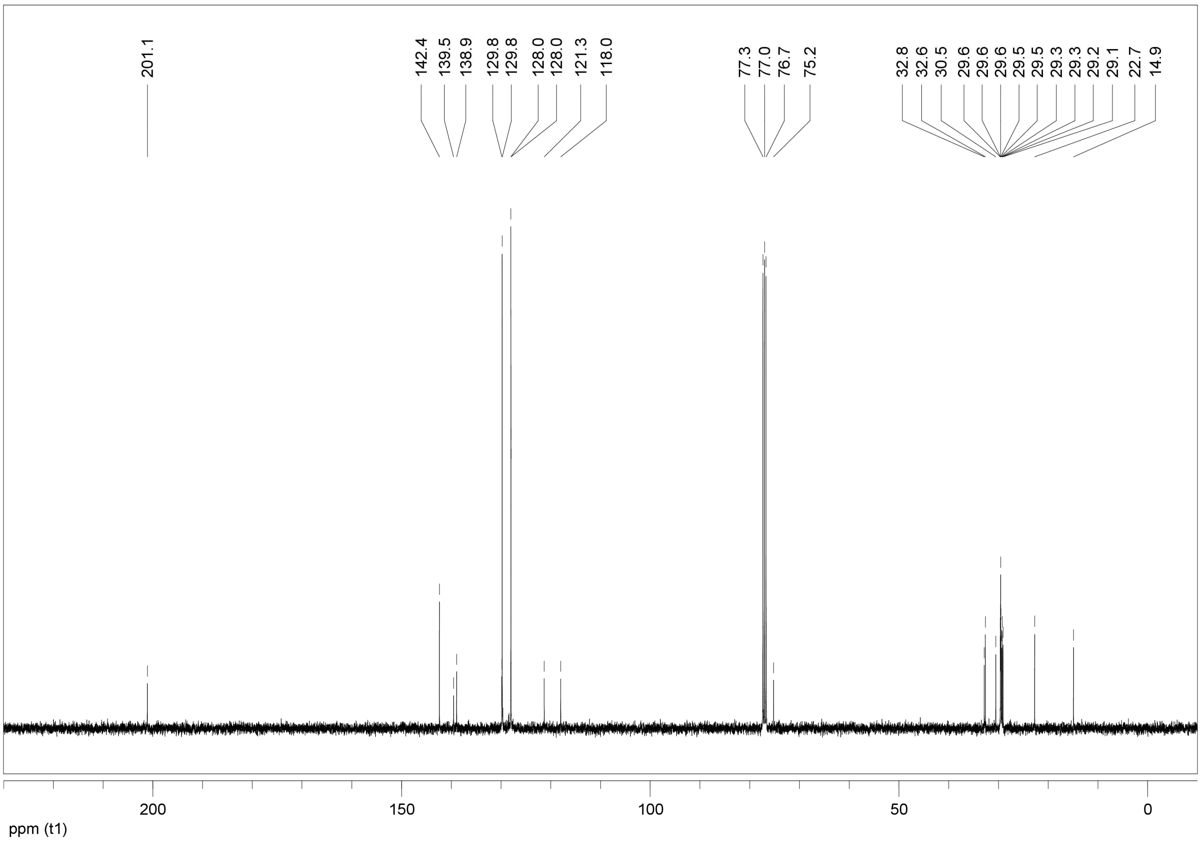

Figure S64: ^{1}H- (400 MHz) and ^{13}C-NMR (100 MHz) spectra of (1*RS*,2*RS*)-2-((*E*)-14-(1-Trityl-1*H*-imidazol-4-yl)tetradec-13-en-1-yl)cyclopropane-1-carbaldehyde

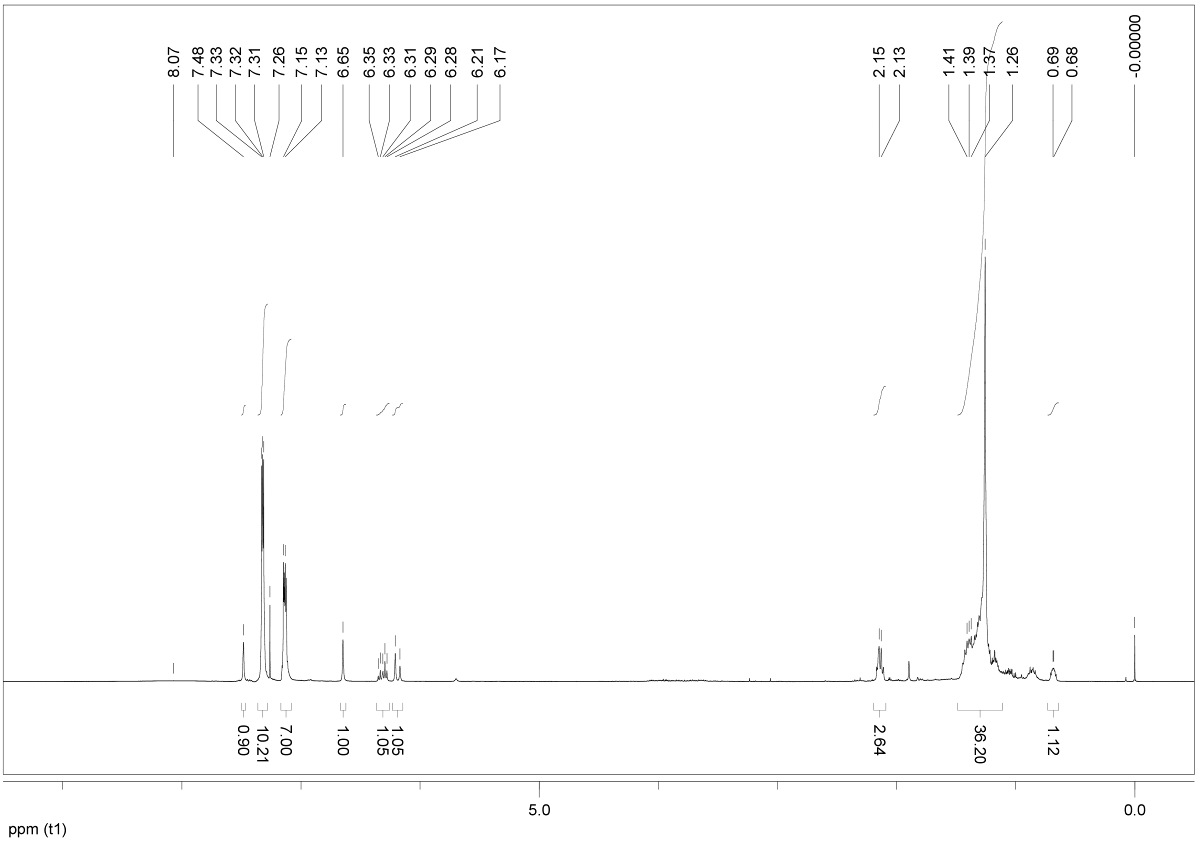

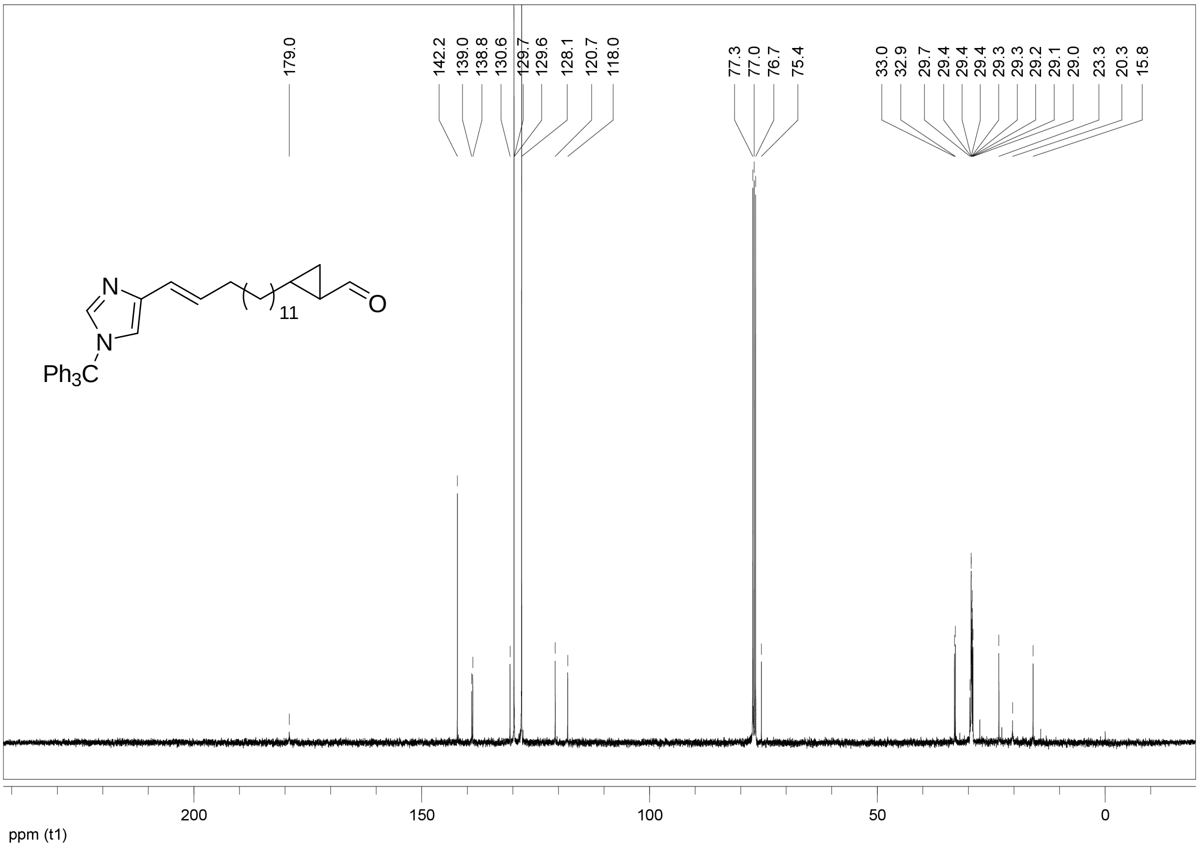

Figure S65: ^{1}H- (400 MHz) and ^{13}C-NMR (100 MHz) spectra of (1*RS*,2*RS*)-2-((*E*)-14-(1-Trityl-1H-imidazol-4-yl)tetradec-13-en-1-yl)cyclopropane-1-carboxylic acid (**46**)

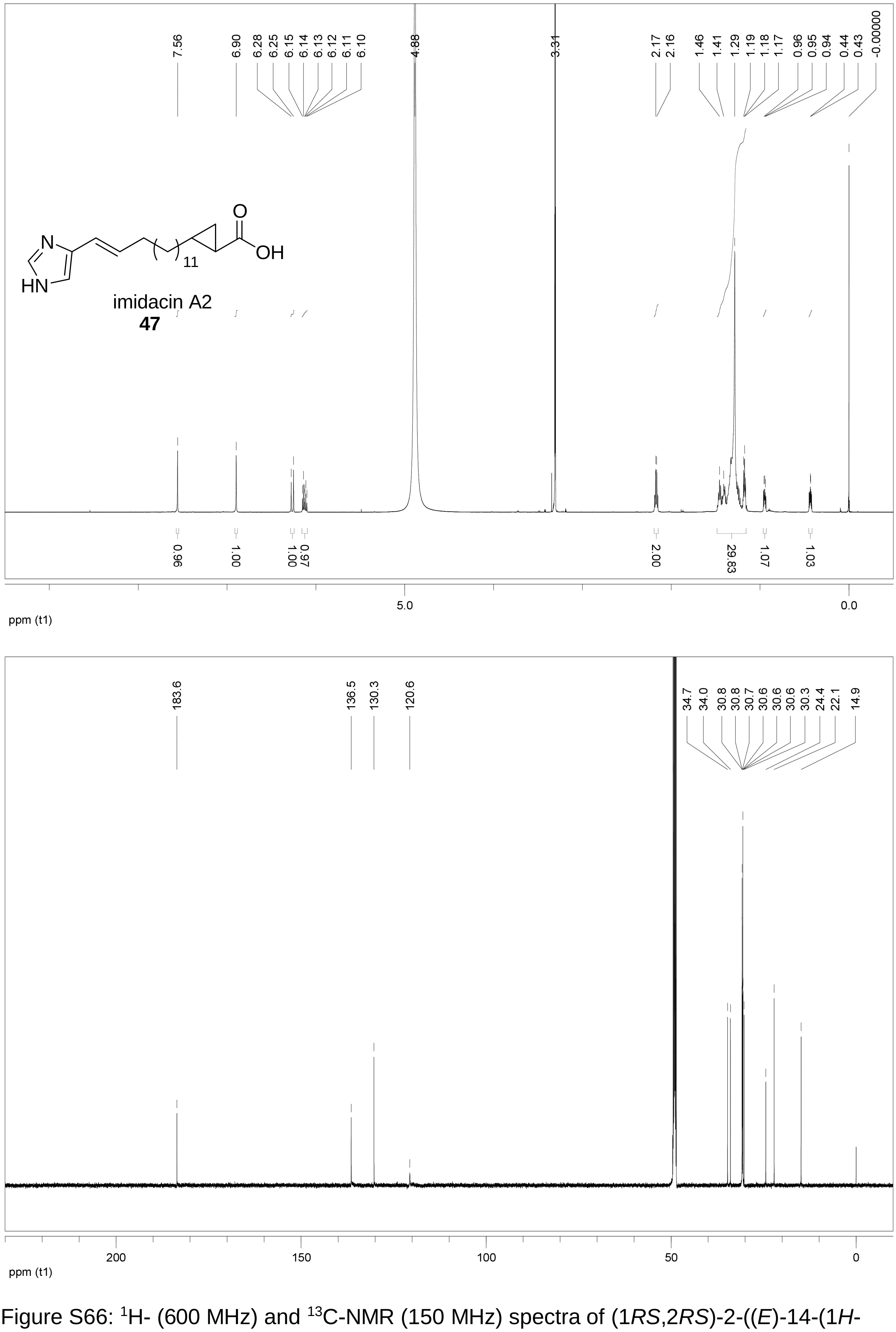

Figure S66: ^{1}H- (600 MHz) and ^{13}C-NMR (150 MHz) spectra of (1*RS*,2*RS*)-2-((*E*)-14-(1*H*-Imidazol-4-yl)tetradec-13-en-1-yl)cyclopropane-1-carboxylic acid (imidacin A2, **47**)
